# World Congress Integrative Medicine & Health 2017: part two: Berlin, Germany. 3–5 May 2017

**DOI:** 10.1186/s12906-017-1783-3

**Published:** 2017-06-30

**Authors:** Carolyn Ee, Sharmala Thuraisingam, Marie Pirotta, Simon French, Charlie Xue, Helena Teede, Agnete E. Kristoffersen, Fuschia Sirois, Trine Stub, Jennifer Engler, Stefanie Joos, Corina Güthlin, Jennifer Felenda, Christiane Beckmann, Florian Stintzing, Roni Evans, Gert Bronfort, Daniel Keefe, Anna Taberko, Linda Hanson, Alex Haley, Haiwei Ma, Joseph Jolton, Lana Yarosh, Francis Keefe, Jung Nam, Roni Evans, Liwanag Ojala, Mary J. Kreitzer, Linda Hanson, Careen Fink, Karin Kraft, Andrew Flower, George Lewith, Kim Harman, Beth Stuart, Felicity L. Bishop, Jane Frawley, Lilla Füleki, Eva Kiss, Tamas Vancsik, Tibor Krenacs, Martha Funabashi, Katherine A. Pohlman, Silvano Mior, Haymo Thiel, Michael D. Hill, David J. Cassidy, Michael Westaway, Jerome Yager, Eric Hurwitz, Gregory N. Kawchuk, Maeve O’Beirne, Sunita Vohra, Isabelle Gaboury, Chantal Morin, Katharina Gaertner, Loredana Torchetti, Martin Frei-Erb, Michael Kundi, Michael Frass, Eugenia Gallo, Valentina Maggini, Mattia Comite, Francesco Sofi, Sonia Baccetti, Alfredo Vannacci, Mariella Di Stefano, Maria V. Monechi, Luigi Gori, Elio Rossi, Fabio Firenzuoli, Rocco D. Mediati, Giovanna Ballerini, Paula Gardiner, Anna S. Lestoquoy, Lily Negash, Sarah Stillman, Prachi Shah, Jane Liebschutz, Pamela Adelstein, Christine Farrell-Riley, Ivy Brackup, Brian Penti, Robert Saper, Isabel Giralt Sampedro, Gilda Carvajal, Andreas Gleiss, Marie M. Gross, Dorothea Brendlin, Jonas Röttger, Wiebke Stritter, Georg Seifert, Noelle Grzanna, Rainer Stange, Peter W. Guendling, Wen Gu, Yan Lu, Jie Wang, Chengcheng Zhang, Hua Bai, Yuxi He, Xiaoxu Zhang, Zhengju Zhang, Dali Wang, Fengxian Meng, Alexander Hagel, Heinz Albrecht, Claudia Vollbracht, Wolfgang Dauth, Wolfgang Hagel, Francesco Vitali, Ingo Ganzleben, Hans Schultis, Peter Konturek, Jürgen Stein, Markus Neurath, Martin Raithel, Alexander Hagel, Claudia Vollbracht, Martin Raithel, Peter Konturek, Bianka Krick, Heidemarie Haller, Petra Klose, Gustav Dobos, Sherko Kümmel, Holger Cramer, Heidemarie Haller, Felix J. Saha, Anna Kowoll, Barbara Ebner, Bettina Berger, Gustav Dobos, Kyung-Eun Choi, Lisha He, Han Wang, X. He, C. Gu, Y. Zhang, Linhua Zhao, Xiaolin Tong, Lisha He, Han Wang, Xinhui He, Chengjuan Gu, Ying Zhang, Linhua Zhao, Xiaolin Tong, Lisha He, Han Wang, Xinhui He, Chengjuan Gu, Ying Zhang, Linhua Zhao, Xiaolin Tong, Robin S. T. Ho, Vincent C. H. Chung, Xinyin Wu, Charlene H. L. Wong, Justin C. Y. Wu, Samuel Y. S. Wong, Alexander Y. L. Lau, Regina W. S. Sit, Wendy Wong, Michelle Holmes, Felicity Bishop, Lynn Calman, Michelle Holmes, Felicity Bishop, George Lewith, Dave Newell, Jonathan Field, Win L. Htut, Dongwoon Han, Da I. Choi, Soo J. Choi, Ha Y. Kim, Jung H. Hwang, Ching W. Huang, Bo H. Jang, Fang P. Chen, Seong G. Ko, Wenjing Huang, De Jin, Fengmei Lian, Soobin Jang, Kyeong H. Kim, Eun K. Lee, Seung H. Sun, Ho Y. Go, Youme Ko, Sunju Park, Bo H. Jang, Yong C. Shin, Seong G. Ko, Hubert Janik, Natalie Greiffenhagen, Jürgen Bolte, Karin Kraft, Mariusz Jaworski, Miroslawa Adamus, Aleksandra Dobrzynska, Michael Jeitler, Jessica Jaspers, Christel von Scheidt, Barbara Koch, Andreas Michalsen, Nico Steckhan, Christian Kessler, De Jin, Wen-jing Huang, Bing Pang, Feng-Mei Lian, Miek Jong, Erik Baars, Anja Glockmann, Harald Hamre, Mosaburo Kainuma, Aya Murakami, Toshio Kubota, Daisuke Kobayashi, Yasuhiro Sumoto, Norihiro Furusyo, Shin-Ichi Ando, Takao Shimazoe, Olaf Kelber, S. Verjee, Eva Gorgus, Dieter Schrenk, Kathi Kemper, Ellie Hill, Kathi Kemper, Nisha Rao, Gregg Gascon, John Mahan, Gunver Kienle, Jörg Dietrich, Claudia Schmoor, Roman Huber, Weon H. Kim, Dongwoon Han, Mansoor Ahmed, Luzhu He, Jung Hye Hwang, Eva Kiss, Tamas Vancsik, Nora Meggyeshazi, Csaba Kovago, Tibor Krenacs, Anne K. Klaus, Roland Zerm, Danilo Pranga, Thomas Ostermann, Marcus Reif, Hans Broder von Laue, Benno Brinkhaus, Matthias Kröz, Anne K. Klaus, Roland Zerm, Danilo Pranga, Daniela Rodrigues Recchia, Thomas Ostermann, Marcus Reif, Hans B. von Laue, Benno Brinkhaus, Matthias Kröz, Christien T. Klein-Laansma, Mats Jong, Cornelia von Hagens, Jean P. Jansen, Herman van Wietmarschen, Miek C. Jong, Youme Ko, Seung-Ho Sun, Ho-Yeon Go, Chan-Yong Jeon, Yun-Kyung Song, Seong-Gyu Ko, Anna K. Koch, Sybille Rabsilber, Romy Lauche, Sherko Kümmel, Gustav Dobos, Jost Langhorst, Holger Cramer, Anna K. Koch, Milena Trifunovic-Koenig, Petra Klose, Holger Cramer, Gustav Dobos, Jost Langhorst, Evi Koster, Erik Baars, Diana Delnoij, Lena Kroll, Kathrin Weiss, Ai Kubo, Sarah Hendlish, Andrea Altschuler, Nancy Connolly, Andy Avins, Romy Lauche, Daniela Rodrigues Recchia, Holger Cramer, Jon Wardle, David Lee, David Sibbritt, Jon Adams, Thomas Ostermann, Romy Lauche, David Sibbritt, Crystal Park, Gita Mishra, Jon Adams, Holger Cramer, Johann Lechner, Inseon Lee, Younbyoung Chae, Jisu Lee, Seung H. Cho, Yujin Choi, Jee Y. Lee, Han S. Ryu, Sung S. Yoon, Hye K. Oh, Lyun K. Hyun, Jin O. Kim, Seong W. Yoon, Ju-Yeon Lee, Sang-Hoon Shin, Min Jang, Indra Müller, So-Hyun Janson Park, Anna S. Lestoquoy, Lance Laird, Lily Negash, Suzanne Mitchell, Paula Gardiner, Xiaofei Li, Yunhui Wang, Jianhua Zhen, He Yu, Tiegang Liu, Xiaohong Gu, Hui Liu, Weiguo Ma, Chengcheng Zhang, Xuezheng Shang, Yu Bai, Fengxian Meng, Wei Liu, Collin Rooney, Amos Smith, Shirlene Lopes, Marcelo Demarzo, Maria do Patrocínio Nunes, Peter Lorenz, Carsten Gründemann, Miriam Heinrich, Manuel Garcia-Käufer, Franziska Grunewald, Silke Messerschmidt, Anja Herrick, Kim Gruber, Christiane Beckmann, Matthias Knödler, Roman Huber, Carmen Steinborn, Florian Stintzing, Taoying Lu, Lixin Wang, Darong Wu, Christina M Luberto, Daniel L. Hall, Emma Chad-Friedman, Suzanne Lechner, Elyse R. Park, Christina M. Luberto, Elyse Park, Janice Goodman, Sonja Luer, Matthias Heri, Klaus von Ammon, Martin Frei-Erb, Weiguo Ma, Fengxian Meng, Valentina Maggini, Eugenia Gallo, Ida Landini, Andrea Lapucci, Stefania Nobili, Enrico Mini, Fabio Firenzuoli, Clare McDermott, George Lewith, Selwyn Richards, Diane Cox, Sarah Frossell, Geraldine Leydon, Caroline Eyles, Hilly Raphael, Rachael Rogers, Michelle Selby, Charlotte Adler, Jo Allam, Fengxian Meng, Wen Gu, Chengcheng Zhang, Hua Bai, Zhengju Zhang, Dali Wang, Xiangwei Bu, Honghong Zhang, Jianpeng Zhang, Hui Liu, Michael Mikolasek, Jonas Berg, Claudia Witt, Jürgen Barth, Ivan Miskulin, Zdenka Lalic, Maja Miskulin, Albina Dumic, Damir Sebo, Aleksandar Vcev, Nasr A. A. Mohammed, Dongwoon Han, Mansoor Ahmed, Soo Jeung Choi, Hyea Bin Im, Jung Hye Hwang, Anwesha Mukherjee, Amit Kandhare, Subhash Bodhankar, Anwesha Mukherjee, Amit Kandhare, Prasad Thakurdesai, Subhash Bodhankar, Niki Munk, Erica Evans, Amanda Froman, Matthew Kline, Matthew J. Bair, Frauke Musial, Agnete E. Kristoffersen, Terje Alræk, Harald J. Hamre, Trine Stub, Lars Björkman, Vinjar M. Fønnebø, Bing Pang, Feng-mei Lian, Qing Ni, Xiao-lin Tong, Xin-long Li, Wen-ke Liu, Shuo Feng, Xi-yan Zhao, Yu-jiao Zheng, Xue-min Zhao, Yi-qun Lin, Bing Pang, Feng-mei Lian, Xiao-lin Tong, Tian-yu Zhao, Xi-Yan Zhao, Hui Che Phd, Chen Zhang, Bing Pang, Feng Liu, Xiao-lin Tong, Lin-hua Zhao, Xue-min Zhao, Ru Ye, Cheng-juan Gu, Bing Pang, Qing Ni, Xiao-lin Tong, Feng-mei Lian, Xi-yan Zhao, De Jin, Xue-min Zhao, Yu-jiao Zheng, Yi-qun Lin, Wenbo Peng, Romy Lauche, David Sibbritt, Jon Adams, Wenbo Peng, Jon Wardle, Holger Cramer, Gita Mishra, Romy Lauche, Katherine A. Pohlman, Silvano Mior, Martha Funabashi, Diana De Carvalho, Mohamed El-Bayoumi, Bob Haig, Kimbalin Kelly, Darrell J. Wade, Maeve O’Beirne, Sunita Vohra, Emanuela Portalupi, Giampietro Gobo, Luigi Bellavita, Chiara Guglielmetti, Christa Raak, Myriam Teuber, Friedrich Molsberger, Ulrich von Rath, Ulrike Reichelt, Uta Schwanebeck, Sabine Zeil, Christian Vogelberg, Dolores Rodríguez Veintimilla, Claudia Vollbracht, Guerrero Tapia Mery, Marisol Maldonado Villavicencio, Sandra Herrera Moran, Christian Sachse, Peter W Gündlin, Rainer Stange, Monirsadat Sahebkarkhorasani, Hoda Azizi, Dania Schumann, Romy Lauche, Tobias Sundberg, Matthew J. Leach, Holger Cramer, Susana Seca, Henry Greten, Sugir Selliah, Anu Shakya, Dongwoon Han, Ha Yun Kim, Da I. Choi, Hyea B. Im, Soo J. Choi, Anna Sherbakova, Gudrun Ulrich-Merzenich, Olaf Kelber, Heba Abdel-Aziz, Erica Sibinga, Lindsey Webb, Jonathan Ellen, Kari Skrautvol, Dagfinn Nåden, Rhayun Song, Weronika Grabowska, Kamila Osypiuk, Gloria V. Diaz, Paolo Bonato, Moonkyoung Park, Jeffrey Hausdorff, Michael Fox, Lewis R. Sudarsky, Daniel Tarsy, James Novakowski, Eric A. Macklin, Peter M. Wayne, Rhayun Song, Inok Hwang, Sukhee Ahn, Myung-Ah Lee, Peter M. Wayne, Min K. Sohn, Oleg Sorokin, Nico Steckhan, Dagmar Heydeck, Astrid Borchert, Christoph-Daniel Hohmann, Harmut Kühn, Andreas Michalsen, Christian Kessler, Nico Steckhan, Christoph-Daniel Hohmann, Holger Cramer, Andreas Michalsen, Gustav Dobos, Christel von Scheidt, Clemens Kirschbaum, Tobias Stalder, Barbara Stöckigt, Michael Teut, Ralf Suhr, Daniela Sulmann, Benno Brinkhaus, Chris Streeter, Patrica Gerbarg, Marisa Silveri, Richard Brown, John Jensen, Wiebke Stritter, Britta Rutert, Angelika Eggert, Alfred Längler, Georg Seifert, Christine Holmberg, Jin Sun, Xin Deng, Wen-Yuan Li, Bin Wen, Nicola Robinson, Jian-Ping Liu, Hyun K. Sung, Narae Yang, Ho Y. Go, Seon M. Shin, Hee Jung, Young J. Kim, Woo S. Jung, Tae Y. Park, Kiyoshi Suzuki, Toshinori Ito, Seiya Uchida, Seika Kamohara, Naoya Ono, Mitsuyuki Takamura, Ayumu Yokochi, Kazuo Maruyama, Patricio Tapia, Katarzyna Thabaut, Benno Brinkhaus, Barbara Stöckigt, Anja Thronicke, Matthias Kröz, Megan Steele, Harald Matthes, Cornelia Herbstreit, Friedemann Schad, Jiaxing Tian, Fengmei Lian, Libo Yang, Xiaolin Tong, Tian Tian, Hewei Zhang, Xia Tian, CongCong Wang, Qian Yun Chai, Lijuan Zhang, Ruyu Xia, Na Huang, Yutong Fei, Jianpin Liu, Natalie Trent, Mindy Miraglia, Jeffrey Dusek, Edi Pasalis, Sat B. Khalsa, Milena Trifunovic-König, Petra Klose, Holger Cramer, Romy Lauche, Anna Koch, Gustav Dobos, Jost Langhorst, Lisa Uebelacker, Geoffrey Tremont, Lee Gillette, Gary Epstein-Lubow, David Strong, Ana Abrantes, Audrey Tyrka, Tanya Tran, Brandon Gaudiano, Ivan Miller, Gerhild Ullmann, Gerhild Ullmann, Yuhua Li, Sujata Vaidya, Vinod Marathe, Ana C. Vale, Jacquelyne Motta, Fabíola Donadão, Angela C. Valente, Luana C. Carvalho Valente, Ricardo Ghelman, Dusan Vesovic, Dragan Jevdic, Aleksandar Jevdic, Katarina Jevdic, Mihael Djacic, Dragica Letic, Drago Bozic, Marija Markovic, Slobodan Dunjic, Dusan Vesovic, Dragan Jevdic, Aleksandar Jevdic, Katarina Jevdic, Mihael Djacic, Dragica Letic, Drago Bozic, Marija Markovic, Gordana Ruscuklic, Dezire Baksa, Slobodan Dunjic, Dusan Vesovic, Dragan Jevdic, Aleksandar Jevdic, Katarina Jevdic, Mihael Djacic, Dragica Letic, Drago Bozic, Marija Markovic, Gordana Ruscuklic, Dezire Baksa, Slobodan Dunjic, Dusan Vesovic, Dragan Jevdic, Aleksandar Jevdic, Katarina Jevdic, Mihael Djacic, Dragica Letic, Drago Bozic, Marija Markovic, Gordana Ruscuklic, Dezire Baksa, Slobodan Dunjic, Dusan Vesovic, Dragan Jevdic, Aleksandar Jevdic, Katarina Jevdic, Mihael Djacic, Dragica Letic, Drago Bozic, Marija Markovic, Kenan Vrca, Slobodan Dunjic, Ann Vincent, Dietlind Wahner-Roedler, Mary Whipple, Maria M. Vogelius, Claudia Vollbracht, Iris Friesecke, Peter W. Gündling, Dietlind Wahner-Roedler, Saswati Mahapatra, Rebecca Hynes, Kimberly Van Rooy, Sherry Looker, Aditya Ghosh, Brent Bauer, Susanne Cutshall, Harald Walach, Ana Borges Flores, Harald Walach, Michael Ofner, Andreas Kastner, Gerhard Schwarzl, Hermann Schwameder, Nathalie Alexander, Gerda Strutzenberger, Jie Wang, Yan Lu, Wen Gu, Chengcheng Zhang, Xianwei Bu, Honghong Zhang, Jianping Zhang, Yuxi He, Xiaoxu Zhang, Fengxian Meng, Shang Wang, He Yu, Jinfeng Shi, Yu Hao, Tiegang Liu, Jun Wu, Zeji Qiu, Xiaohong Gu, Yuh-Hai Wang, Chi-Jung Lou, Sam Watts, Peter Wayne, Kamila Osypiuk, Gloria Vergara-Diaz, Paolo Bonato, Brian Gow, Jeffrey Hausdorff, Jose Miranda, Lewis Sudarsky, Daniel Tarsy, Michael Fox, Eric Macklin, Kathrin Wode, Jenny Bergqvist, Britt-Marie Bernhardsson, Johanna Hök Nordberg, Gunver Kienle, Lena Sharp, Roger Henriksson, Yeonju Woo, Min K. Hyun, Hao Wu, Tian-Fang Wang, Yan Zhao, Yu Wei, Lei Tian, Lei He, Xue Wang, Ruohan Wu, Shuo Feng, Mei Han, Patrina H. Y. Caldwell, Shigang Liu, Jing Zhang, Jianping Liu, Ruyu Xia, Qianyun Chai, Yutong Fei, Zhongning Guo, Congcong Wang, Zhijun Liu, Xun Li, Ying Zhang, Jianping Liu, I. J. Yang, V. Ruberio Lincha, S. H. Ahn, D. U. Lee, H. M. Shin, Lu Yang, David Sibbritt, Wenbo Peng, Jon Adams, N. Yang, H. Sung, S. M. Shin, H. Y. Go, H. Jung, Y. Kim, T. Y. Park, Angela Yap, Yu H. Kwan, Chuen S. Tan, Syed Ibrahim, Seng B. Ang, Alfred Yayi, Dongwoon Han, Hyea Bin Im, Jung Hye Hwang, Soo Jeung Choi, Jeong E. Yoo, Ho R. Yoo, Sae B. Jang, Hye L. Lee, Ala’a Youssef, Shahira Ezzat, Amira Abdel Motaal, Hesham El-Askary, Xiaotong Yu, Yashan Cui, Ying Zhang, Fengmei Lian, Younghee Yun, Youme Ko, Jin-Hyang Ahn, Bo-Hyung Jang, Kyu-Seok Kim, Seong-Gyu Ko, Inhwa Choi, Roland Zerm, Augustina Glinz, Danilo Pranga, Bettina Berger, Fadime ten Brink, Marcus Reif, Arnd Büssing, Christoph Gutenbrunner, Matthias Kröz, Roland Zerm, Bert Helbrecht, Danilo Pranga, Benno Brinkhaus, Andreas Michalsen, Matthias Kröz, Honghong Zhang, Tiesheng Fang, Jie Wang, Chengcheng Zhang, Yuxi He, Xiaoxu Zhang, Zhengju Zhang, Dali Wang, Fengxion Meng, Jianping Zhang, Chengcheng Zhang, Hua Bai, Zhiming Shen, Weiguo Ma, Hui Liu, Yu Bai, Xuezheng Shang, Fengxian Meng, Ruixin Zhang, Fan Wu, Ming Li, Xinyun Xuan, Xueyong Shen, Ke Ren, Brian Berman, Jianhua Zhen, Xiaofei Li, Xiaohong Gu, He Yu, Zian Zheng, Yuxiang Wan, Yunhui Wang, Xueyan Ma, Fei Dong, Tiegang Liu, Jianhua Zhen, Xiaofei Li, Xiaohong Gu, He Yu, Zian Zheng, Yuxiang Wan, Yunhui Wang, Xueyan Ma, Fei Dong, Tiegang Liu, Suzie Zick, Richard Harris, Go E. Bae, Jung N. Kwon, Hye Y. Lee, Jong K. Nam, Sang D. Lee, Dong H. Lee, Ji Y. Han, Young J. Yun, Ji H. Lee, Hye L. Park, Seong H. Park, Chiara Bocci, Giovanni B. Ivaldi, Ilaria Vietti, Ilaria Meaglia, Marta Guffi, Rubina Ruggiero, Marita Gualea, Emanuela Longa, Massimo Bonucci, Sarah Croke, Lourdes Diaz Rodriguez, Juan C. Caracuel-Martínez, Manuel F. Fajardo-Rodríguez, Angélica Ariza-García, Francisca García-De la Fuente, Manuel Arroyo-Morales, Maria S. Estrems, Vicente G. Gómez, Maria S. Estrems, Mónica Valero Sabater, Rosaria Ferreri, Simonetta Bernardini, Roberto Pulcri, Franco Cracolici, Massimo Rinaldi, Claudio Porciani, Fabio Firenzuoli, Sonia Baccetti, Mariella Di Stefano, Maria V. Monechi, Eugenia Gallo, Valentina Maggini, Luigi Gori, Elio Rossi, Peter Fisher, John Hughes, Ariadna Mendoza, Hugh MacPherson, Claudia Witt, Jacqueline Filshie, George Lewith, Antonia Di Francesco, Alberto Bernardini, Monica Messe, Vincenzo Primitivo, Piera A. Iasella, Ricardo Ghelman, Monica Taminato, Jaqueline Do Carmo Alcantara, Katia R. De Oliveira, Debora C. De Azevedo Rodrigues, Juliana R. Campana Mumme, Olga K. Matsumoto Sunakozawa, Vicente Odone Filho, Georg Seifert, Joshua Goldenberg, Andrew Day, Masa Sasagawa, Lesley Ward, Kieran Cooley, Thora Gunnarsdottir, Ingibjorg Hjaltadottir, Mahdie Hajimonfarednejad, Nicole Hannan, Rut Hellsing, Kathrin Wode, Johanna Hök Nordberg, Johanna Hök Nordberg, Susanne Andermo, Maria Arman, Iris von Hörsten, Patricia Vásquez Torrielo, Carmen L. Andrade Vilaró, Francisco Cerda Cabrera, Roman Huber, Henny Hui, Eric Ziea, Dora Tsui, Joyce Hsieh, Christine Lam, Edith Chan, Mark P. Jensen, Samuel L. Battalio, Joy Chan, Karlyn A. Edwards, Kevin J. Gertz, Melissa A. Day, Leslie H. Sherlin, Dawn M. Ehde, Kyeong H. Kim, Soobin Jang, Bo-Hyoung Jang, Ho-Yeon Go, Sunju Park, Seong-Gyu Ko, Karin Kraft, Hubert Janik, Anja Börner, Jihong Lee, Boram Lee, Gyu T. Chang, Alejandra Menassa, Yoshiharu Motoo, Jürgen Müller, Sabine Rabini, Bettina Vinson, Olaf Kelber, Martin Storr, Karin Kraft, Martin Niemeijer, Erik Baars, Joop Hoekman, Wied Ruijssenaaars, Faith C. Njoku, Petra Klose, Benno Brinkhaus, Andreas Michalsen, Gustav Dobos, Holger Cramer, Arne J. Norheim, Terje Alræk, Filiz Okumus, Halime Oncu-Celik

**Affiliations:** 1 National Institute of Complementary Medicine, Western Sydney University, Sydney, Australia; 2 Department of General Practice, University of Melbourne, Melbourne, Australia; 3 School of Rehabilitation Therapy, Queens University, Ontario, Canada; 4 School of Health and Biomedical Sciences, RMIT University, Melbourne, Australia; 5 School of Public Health and Preventive Medicine, Monash University, Melbourne, Australia; 6 NAFKAM, UiT, The Arctic University of Norway, Tromsø, 9019 Norway; 7 Department of Psychology, University of Sheffield, Sheffield, S1 1HD UK; 8 Department of Community Medicine, UiT The Arctic University of Norway, Tromsø, 9019 Norway; 9 Institue of General Practice, Goethe University Frankfurt, Frankfurt, 60590 Germany; 10 Department of General Practice, University of Tübingen, Tübingen, Germany; 11 Med.-pharm. Information/Klinische Forschung, WALA Heilmittel GmbH, Bad Boll/ Eckwälden, 73087 Germany; 12 Ressortleitung Wissenschaft, WALA Heilmittel GmbH, Bad Boll/Eckwälden, 73087 Germany; 13 University of Minnnesota, Minneapolis, 55455 MN USA; 14 Minneapolis College of Art and Design, Minneapolis, MN USA; 15 Duke University, Durham, NC USA; 16 University of Minnesota, Minneapolis, 55455 MN USA; 17 Caring Bridge, Minneapolis, MN USA; 18 Phytomedicines Supply and Development Center, Innovation & Development, Bayer Consumer Health Steigerwald Arzneimittelwerk GmbH, Darmstadt, 68526 Germany; 19 University Medicine of Rostock, Rostock, 18055 Germany; 20 Primary Care and Population Sciences, University of Southampton, Southampton, UK; 21 Psychology, University of Southampton, Southampton, UK; 22 Faculty of Health, University of Technology Sydney, Sydney, 2007 Australia; 23 1st Department of Pathology and Experimental Cancer Research, Semmelweis University, Budapest, 1085 Hungary; 24 University of Alberta, Edmonton, Canada; 25 Parker University, Dallas, TX USA; 26 Canadian Memorial Chiropractic College, Toronto, Canada; 27 Anglo-European College of Chiropractic, Bournemouth, UK; 28 University of Calgary, Calgary, Canada; 29 University of Toronto, Toronto, Canada; 30 University of Hawaii at Manoa, Honolulu, Hawai’I USA; 31 Family Medicine, Universite de Sherbrooke, Sherbrooke, J1H 5N4 Canada; 32 Institute for Complementary Medicine, University of Bern, Bern, 3008 Switzerland; 33 Institute of Environmental Health, Medical University of Vienna, Vienna, 1090 Austria; 34 Department of Medicine I, Clinical Division of Oncology, Medical University of Vienna, Vienna, 1090 Austria; 35 Department of Experimental and Clinical Medicine, University of Florence, Florence, Italy; 36 Center for Integrative Medicine, Careggi Universtary Hospital, Florence, Italy; 37 Referring Centre for Phytoterapy, Region Tuscany, Florence, Italy; 38 Tuscan Network of Integrative Medicine, Region of Tuscany, Florence, Italy; 39 Cure Palliative e Terapia Del Dolore, Careggi Universitary Hospital, Florence, Italy; 40 Family Medicine, Boston University Medical Center, Boston, 02118 MA USA; 41 Boston University School of Medicine, Boston, MA USA; 42 Codman Square Health Center, Dorchester, MA USA; 43 Dothouse Health Center, Dorchester, MA USA; 44 ENERGIMED, Barcelona, 08007 Spain; 45 Ginecology, Obstetrics and Reproduction, Hospital Universitari Quiron Dexeus, Barcelona, Spain; 46 Center for Medical Statistics, Informatics, and Intelligent Systems, Medical University of Vienna, Vienna, 1090 Austria; 47 AG für Integrative Medizin in der Pädiatrischen Onkologie, Charité Universitätsmedizin, Berlin, 13353 Germany; 48 Complementary Medicine, Hochschule Fresenius, München, 80798 Germany; 49 Naturheilkunde, Immanuel-Krankenhaus, Berlin, 14109 Germany; 50 Beijing Hospital of Traditional Chinese Medicine, Rheumatism, Beijing, China; 51 Emergency Department, Xiyuan Hospital, Beijing, China; 52 Dongfang Hospital, Beijing University of Chinese Medicine, Rheumatism, Beijing, China; 53 Dongfang Hospital, Beijing University of Chinese Medicine, Nephrology, Beijing, China; 54 Rehabilitation Department, Xiluoyuan Community Health Service Centre, Beijing, China; 55 University of Erlangen, Erlangen, 91054 Germany; 56 Pascoe Naturmedizin, Gießen, 35394 Germany; 57 Institute of Employment Research, Nuremberg, Germany; 58 Martha Maria, Nuremberg, Germany; 59 Synlab, Weiden, Germany; 60 Thuringia Clinic, Saalfeld, Germany; 61 Department of Nutritional Medicine, Sachsenhausen, Germany; 62 University of Erlangen, Erlangen, 91054 Germany; 63 Pascoe Naturmedizin, Gießen, 35394 Germany; 64 Thuringia Clinic, Saalfeld, Germany; 65 Department of Internal and Integrative Medicine, Faculty of Medicine, University of Duisburg-Essen, Essen, 45276 Germany; 66 Breast Unit, Kliniken Essen-Mitte, Essen, Germany; 67 Department of Internal and Integrative Medicine, Faculty of Medicine, University of Duisburg-Essen, Essen, 45276 Germany; 68 Department of Health, Institute for Integrative Medicine, Witten/Herdecke University, Herdecke, Germany; 69 Guang’anmen Hospital, China Acadamy of Chinese Medicine Sciences, Beijing, China; 70 Guang’anmen Hospital, China Acadamy of Chinese Medicine Sciences, Beijing, China; 71 Guang’anmen Hospital, China Acadamy of Chinese Medicine Sciences, Beijing, China; 72 Jockey Club School of Public Health and Primary Care, The Chinese University of Hong Kong, Hong Kong, 852 Hong Kong; 73 Hong Kong Institute of Integrative Medicine, The Chinese University of Hong Kong, Hong Kong, 852 Hong Kong; 74 Psychology, University of Southampton, Southampton, SO17 1BJ UK; 75 Faculty of Health Sciences, University of Southampton, Southampton, SO17 1BJ UK; 76 Psychology, University of Southampton, Southampton, SO17 1BJ UK; 77 Primary Medical Care, University of Southampton, Southampton, SO16 5ST UK; 78 Anglo-European College of Chiropractic, Bournemouth, BH5 2DF UK; 79 Back2Health, Southsea, PO4 0DW UK; 80 Global Health and Development, Hanyang University, College of Medicine, Seoul, 133-791 South Korea; 81 Department of Preventive Medicine, College of Korean Medicine, Kyung Hee University, Seoul, 02447 South Korea; 82 Institute of Safety, Efficacy and Effectiveness Evaluation for Korean Medicine, Kyung Hee University, Seoul, South Korea; 83 Taipei Veterans General Hospital, Taipei, Taiwan; 84 Department of Endocrinology, Guang’anmen Hospital China Academy of Chinese Medical Sciences, Beijing, 100053 China; 85 Kyung Hee University, Seoul, 02447 South Korea; 86 Sangji University Korean Medicine Hospital, Wonju, South Korea; 87 Semyung University, Jecheon, South Korea; 88 Daejeon University, Daejeon, South Korea; 89 Complementary Medicine, University of Medicine Rostock, Rostock, 18057 Germany; 90 Strandklinik Boltenhagen, Boltenhagen, Germany; 91 Department of Medical Psychology, Medical University of Warsaw, Warsaw, 02-091 Poland; 92 Department of Internal and Complementary Medicine, Immanuel Hospital Berlin, Berlin, 14109 Germany; 93 Charité - University Medical Center, Berlin, 10117 Germany; 94 Guang An Men Hospital of China Academy of Chinese Medical Sciences, Beijing, 100053 China; 95 Louis Bolk Institute, Driebergen, 3972LA Netherlands; 96 IFAEMM at the Witten/Herdecke University, Freiburg, Germany; 97 Community Medicine Education Unit, Graduate School Of Medical Science, Kyushu University, Fukuoka, 812-8582 Japan; 98 Department of Clinical Pharmacy and Pharmaceutical Care, Graduate School of Pharmaceutical Sciences, kyushu University, Fukuoka, Japan; 99 Department of General Internal Medicine, Kyushu University, Fukuoka, Japan; 100 Sleep Apnea Center, Kyushu University Hospital, Fukuoka, Japan; 101 Innovation & Development, Phytomedicines Supply and development Center, Steigerwald Arzneimittelwerk GmbH, Bayer Consumer Health, Darmstadt, 64295 Germany; 102 Food Chemistry and Toxicology, University of Kaiserslautern, Kaiserslautern, 67663 Germany; 103 OSU, Blacklick, 43004 OH USA; 104 OSU, Blacklick, 43004 OH USA; 105 College of Medicine, OSU, Columbus, 43210 OH USA; 106 Center for Complementary Medicine, Institute for Environmental Health Sciences and Hospital Infection Control, Medical Center – University of Freiburg, Freiburg, 79106 Germany; 107 Massachusetts General Hospital, Boston, MA USA; 108 Clinical Trials Unit Freiburg, Medical Center – University of Freiburg, Freiburg, Germany; 109 Graduate School of Public Policy, Hanyang University, Seoul, 133-791 South Korea; 110 Global Health and Development, Hanyang University, College of Medicine, Seoul, 04763 South Korea; 111 1st Department of Pathology and Experimental Cancer Research, Semmelweis University, Budapest, 1085 Hungary; 112 Department of Pharmacology and Toxicology, Szent Istvan University Faculty of Veterinary Science, Budapest, 1078 Hungary; 113 Research Institute Havelhöhe, Berlin, 14089 Germany; 114 Internal Medicine, Hospital Havelhöhe, Berlin, Germany; 115 Integrative Medicine, Witten/Herdecke University, Witten/Herdecke, Germany; 116 Methodology and Statistics in Psychology and Psychotherapy, Witten/Herdecke University, Witten/Herdecke, Germany; 117 Society for Clinical Research, Berlin, Germany; 118 Institute for Social Medicine, Epidemiology and Health Economics, Charité University, Berlin, 10117 Germany; 119 Research Institute Havelhöhe, Berlin, 14089 Germany; 120 Internal Medicine, Hospital Havelhöhe, Berlin, Germany; 121 Integrative Medicine, Witten/Herdecke University, Witten/Herdecke, Germany; 122 Methodology and Statistics in Psychology and Psychotherapy, Witten/Herdecke University, Witten/Herdecke, Germany; 123 Society for Clinical Research, Berlin, Germany; 124 Institute for Social Medicine, Epidemiology and Health Economics, Charité University, Berlin, Germany; 125 Health Care and Nutrition, Louis Bolk Institute, Driebergen, 3972LA Netherlands; 126 Nursing, Mid-Sweden University, Sundsvall, Sweden; 127 Naturopathy and Integrative Medicine, Department of Gynaecological Endocrinology and Reproductive Medicine, University Women’s Hospital Heidelberg, Heidelberg, Germany; 128 Preventive Medicine, Kyung Hee University, Seoul, 02447 South Korea; 129 Korean Internal Medicine, Sangji University, Wonju, South Korea; 130 Korean Internal Medicine, Semyung University, Jecheon, South Korea; 131 Korean Internal Medicine, Gachon University, Seongnam, South Korea; 132 Rehabilitation Medicine, Gachon University, Seongnam, South Korea; 133 Department of Internal and Integrative Medicine, Kliniken Essen-Mitte, Essen, 45276 Germany; 134 Centre of Integrative Gastroenterology, Kliniken Essen-Mitte, Essen, Germany; 135 Department of Gynecology, Malteser Hospital St. Anna, Duisburg, Germany; 136 Interdisclipinary Breast Cancer Center, Kliniken Essen-Mitte, Essen, Germany; 137 Australian Research Center in Complementary and Integrative Medicine, Faculty of Health, Sydney, Australia; 138 Department of Internal and Integrative Medicine, Kliniken Essen-Mitte, Essen, 45276 Germany; 139 Centre of Integrative Gastroenterology, Kliniken Essen-Mitte, Essen, Germany; 140 Australian Research Center in Complementary and Integrative Medicine, Faculty of Health, Sydney, Australia; 141 University of Applied Sciences Leiden, Leiden, 2333CK Netherlands; 142 Scientific Centre for Transformation in Care and Welfare (Tranzo), Tilburg University, Leiden, Netherlands; 143 Sport sciences, University of Augsburg, Augsburg, 86135 Germany; 144 Division of Research, Kaiser Permanente, Oakland, CA 94612 USA; 145 Department of Medicine, University of California, San Francisco, CA USA; 146 Australian Research Centre in Complementary and Integrative Medicine (ARCCIM), University of Technology Sydney, Ultimo, 2007 Australia; 147 Department of Psychology and Psychotherapy, University of Witten/Herdecke, Witten, Germany; 148 Department of Public Health Sciences, University of Miami, Miami, FL USA; 149 Australian Research Centre in Complementary and Integrative Medicine (ARCCIM), University of Technology Sydney, Ultimo, 2007 Australia; 150 Department of Psychology, University of Connecticut, Storrs, CT USA; 151 School of Public Health, University of Queensland, Herston, QLD Australia; 152 Department of Internal and Integrative Medicine, University of Duisburg-Essen, Essen, Germany; 153 Praxisklinik München, Munich, 81547 Germany; 154 Abt. Innere Medizin VI - Psychosomatische Medizin und Psychotherapie, Universitätsklinikum Tübingen, Tübingen, 72076 Germany; 155 Acupuncture & Meridian Science Research Center, Seoul, 130-701 South Korea; 156 Kyung Hee University, College of Korean Medicine, Seoul, South Korea; 157 Department of Clinical Korean Medicine, Kyung Hee University, Graduate School, Seoul, South Korea; 158 Kyung Hee University Hospital at Gangdong, Korean Internal Medicine, Seoul, South Korea; 159 Cardiovascular Engineering, Helmholtz Institute of RWTH Aachen University & Hospital, Aachen, 52074 Germany; 160 Oriental biomedical engineering, Sangji University, Wonju, 26339 South Korea; 161 Family Medicine, Boston University Medical Center, Boston, 02118 USA; 162 Boston University School of Medicine, Boston, USA; 163 Beijing University of Chinese Medicine, Beijing, 100029 China; 164 Pediatrics, First People’s Hospital of Pingyuan County, Shangdong, China; 165 Department of Pulmonary Diseases, China-Japan Friendship Hospital, Beijing, China; 166 Beijing University of Chinese Medicine Dong Fang Hospital, Chinese Medicine, Beijing, China; 167 Edward Via College of Osteopathic Medicine, Auburn, AL USA; 168 Northwestern University, Evanston, IL USA; 169 Auburn University, Auburn, AL USA; 170 Internal Medicine-General Internal Medicine and Semiology, University of São Paulo-School of Medicine, São Paulo, 01218011 Brazil; 171 Public Health, Brazilian Center for Mindfulness and Health Promotion – UNIFESP, São Paulo, 04753-060 Brazil; 172 Department of Analytical Development and Research, Wala Heilmittel GmbH, Bad Boll/Eckwaelden, 73087 Germany; 173 Center for Complementary Medicine, Institute for Environmental Health Sciences and Hospital Infection Control, University Medical Center, Freiburg, 79106 Germany; 174 Department of Pharmacology and Clinical Research, Wala Heilmittel GmbH, Bad Boll/Eckwaelden, 73087 Germany; 175 The Second Affiliated Hospital of Guangzhou University of Chinese Medicine, Guangzhou, 510120 China; 176 The Affiliated Hospital of Changchun University of Chinese Medicine, Changchun, China; 177 Harvard Medical School/ Massachusetts General Hospital, Boston, 02114 MA USA; 178 Benson-Henry Institute for Mind-Body Medicine, Massachusetts General Hospital, Boston, MA USA; 179 University of Miami, Miami, FL USA; 180 Harvard Medical School/Massachusetts General Hospital, Boston, 02114 MA USA; 181 Division of Pediatric Hematology/Oncology, University Children’s Hospital, Bern, Switzerland; 182 Institute of Complementary Medicine, University, Bern, Switzerland; 183 Dongfang Hospital of Beijing University of Traditional Medicine, Clinical, Beijing, China; 184 Department of Experimental and Clinical Medicine, University of Florence, Florence, viale Pieraccini 6, 50139 Florence, Italy; 185 Department of Health Sciences, University of Florence, viale Pieraccini 6, 50139 Florence, Italy; 186 Referring Center for Phytotherapy, Centre for Integrative Medicine - Careggi, Universitary Hospital, 50139 Florence, Italy; 187 Primary Care and Population Science, University of Southampton, Southampton, SO16 5ST UK; 188 University of Cumbria, Carlisle, UK; 190 Rebuilding Your Life Project, Oxford, UK; 191 Oxfordshire CFS/ME Service, Oxford, UK; 192 Department of Rheumatology, Dongfang Hospital, Beijing University of Chinese Medicine, Beijing, China; 193 Department of Rheumatology, Beijing Hospital of Traditional Chinese Medicine, Beijing, China; 194 Department of Nephrology, Dongfang Hospital, Beijing University of Chinese Medicine, Beijing, China; 195 Department of Traditional Chinese Medicine, Xiluoyuan Community Health Service Center of Fengtai Distict, Beijing, China; 196 Institute for Complementary and Integrative Medicine, UniversityHospital Zurich, Zurich, 8091 Switzerland; 197 Institute for Integrative Medicine, Faculty of Medicine Osijek, Josip Juraj Strossmayer University of Osijek, Osijek, 31000 Croatia; 198 APIPHARMA Ltd., Zagreb, Croatia; 199 Department of Public Health, Faculty of Medicine Osijek, Josip Juraj Strossmayer University of Osijek, Osijek, 31000 Croatia; 200 Department of Internal Medicine, Family Medicine and Medical History, Faculty of Medicine Osijek, Josip Juraj Strossmayer University of Osijek, Osijek, Croatia; 201 Global Health and Development, Hanyang University, College of Medicine, Seoul, 04763 South Korea; 202 Hanyang University, College of Medicine, Seoul, 133-791 South Korea; 522 Global Health and Development, Hanyang University, Seoul, South Korea; 203 Department of Pharmacology, Poona College of Pharmacy, BVDU, Pune, India; 204 Department of Pharmacology, Poona College of Pharmacy, BVDU, Pune, India; 205 Indus Biotech Private Limited, Pune, India; 206 Health Sciences, Indiana University School of Health and Rehabilitation Sciences, Indianapolis, 46202 IN USA; 207 Roudebush VA Medical Center, Indianapolis, IN USA; 208 Department of Community Medicine, National Research Center in Complementary and Alternative Medicine, NAFKAM, The Arctic University of Norway, UiT, Tromsø, 9037 Norway; 209 Institute for Applied Epistemology and Medical Methodology, University of Witten-Herdecke, Herdecke, Germany; 210 Dental Biomaterials Adverse Reaction Unit, Uni Health, Uni Research, Bergen, Norway; 211 Department of Endocrinology, Guang anmen Hospital of China Academy of Chinese Medical Sciences, Beijing, 100053 China; 212 Institute of Basic Research in Clinical Medicine, China Academy of Chinese Medical Sciences, Beijing, China; 213 Centre for Evidence-Based Chinese Medicine, Beijing University of Chinese Medicine, Beijing, China; 214 Department of Endocrinology, Guang anmen Hospital of China Academy of Chinese Medical Sciences, Beijing, 100053 China; 215 Endocrinology, First Teaching Hospital of Tianjin University of Traditional Chinese Medicine, Tianjin, China; 216 Digestion, Xiyuan Hospital of China Academy of Chinese Medical Sciences, Beijing, China; 217 Department of Endocrinology, Guang anmen Hospital of China Academy of Chinese Medical Sciences, Beijing, 100053 China; 218 Obesity Research Center, Beijing Wuguanda Biological Technology co., LTD., Beijing, 100053 China; 219 Traditional Chinese Medicine, Centre for Evidence-Based Chinese Medicine, Beijing University of Chinese Medicine, Beijing, Beijing, China; 220 Department of Endocrinology, Guang anmen Hospital of China Academy of Chinese Medical Sciences, Beijing, 100053 China; 221 Faculty of Health, University of Technology Sydney, Sydney, 2007 Australia; 222 Faculty of Health, University of Technology Sydney, Sydney, 2007 Australia; 223 Faculty of Medicine, University of Duisburg-Essen, Essen, Germany; 224 School of Public Health, University of Queensland, Brisbane, Australia; 225 University of Alberta, Edmonton, T6G 2C8 Canada; 226 Parker University, Dallas, USA; 227 Canadian Memorial Chiropractic College, Toronto, Canada; 228 Memorial University of Newfoundland, St. John’s, Canada; 229 New Brunswick Chiropractors’ Association, Fredericton, Canada; 230 Ontario Chiropractic Association, Toronto, Canada; 231 Newfoundland & Labrador Chiropractic Association, St. John’s, Canada; 232 University of Calgary, Calgary, Canada; 233 ARESMA, Milano, Italy; 234 ARTOI, Roma, Italy; 235 Department of Philosophy, University of Milano, Milano, Italy; 236 Politecnico of Milano, School of Design, Milano, Italy; 237 Department of Economics, Management and Quantitative Methods, University of Milano, DEMM, Milano, Italy; 238 Institute for Integrative Medicine, Witten/Herdecke University, Herdecke, 58313 Germany; 239 H:G - Hochschule für Gesundheit und Sport GmbH, Berlin, 10367 Germany; 240 Acupuncture Research Group, Düsseldorf, Germany; 241 Naturopathy and Acupuncture, Medical Office, Berlin, 14197 Germany; 242 Institute of Family Medicine, University Hospital Schleswig-Holstein, Campus Luebeck, Luebeck, 23570 Germany; 243 Technische Universität Dresden, Medizinische Fakultät Carl Gustav Carus, Dresden, Germany; 244 Koordinierungszentrum für klinische Studien, Dresden, Germany; 245 Universitätsklinikum Carl Gustav Carus, Klinik und Poliklinik für Kinder- und Jugendmedizin, Dresden, Germany; 246 Clinical Nutrition and Dietetics, SOLCA Cancer Hospital, Guayaquil, 000 Ecuador; 247 Pascoe Naturmedizin, Gießen, 35394 Germany; 248 Department of Natural Medicine, Immanuel Krankenhaus Berlin-Wannsee, Berlin, 14109 Germany; 249 Naturheilkunde und Komplementäre Medizin, Hochschule Fresenius, Idstein (Taunus), Germany; 250 Iranian Traditional Medicine, Mashhad Medical University, Mashhad, 9172314 Islamic Republic of Iran; 251 Department of Complementary Medicine, Mashhad University of Medical Sciences, Mashhad, 9135913556 Islamic Republic of Iran; 252 Department of Internal and Integrative Medicine, Kliniken Essen-Mitte, Faculty of Medicine, University of Duisburg-Essen, Essen, 45276 Germany; 253 Australian Research Centre in Complementary and Integrative Medicine (ARCCIM), Faculty of Health, University of Technology Sydney, Sydney, New South Wales Australia; 254 Department of Neurobiology, Care Sciences and Society (NVS/OMV), Karolinska Institutet, Stockholm, Sweden; 255 Chinese Medicine, Heidelberg School of Chinese Medicine, Heidelberg, Germany; 256 Faculty of Medicine, University of Coimbra, Coimbra, Portugal; 257 Traditional Chinese Medicine, Institute of Biomedical Sciences Abel Salazar, University of Porto, Porto, Portugal; 258 Department of Systematic and Evolutionary Botany, University of Zurich, Zürich, 8008 Switzerland; 259 Global Health and Development, Hanyang University, College of Medicine, Seoul, 04763 South Korea; 260 Healthcare Administration, Hanyang University, Seoul, 133-791 South Korea; 523 Global Health and Development, Hanyang University, Seoul, South Korea; 261 Medizinische Klinik III, UKB, Friedrich Wilhelms-Universität Bonn, Bonn, 53127 Germany; 262 Department of Forestry, Volga State Technical University, Yoshkar-Ola, Russian Federation; 263 Phytomeidines Supply and Development Center, Steigerwald Arzneimittelwerk GmbH, Bayer Consumer Health, Darmstadt, 64295 Germany; 264 Bayer Consumer Health, Medicial Affairs, Phytomedicines Supply and Development Center, Steigerwald Arzneimittelwerk GmbH, Darmstadt, Germany; 265 Pediatrics, Johns Hopkins School of Medicine, Baltimore, 21224 MD USA; 266 Mental Health, Johns Hopkins School of Public Health, Baltimore, MD USA; 267 Johns Hopkins School of Public Health, Baltimore, MD USA; 268 Faculty of Health Sciences, Oslo and Akershus University College of Applied Sciences, Oslo, 0130 Norway; 269 Department of Nursing, Chungnam National University, Daejeon, 301747 South Korea; 270 Osher Center for Integrative Medicine, Harvard Medical School and Brigham and Women’s Hospital, Boston, MA 02215 USA; 271 Physical Medicine & Rehabilitation, Harvard Medical School, Boston, MA USA; 272 Nursing, Woosong College, Daejeon, South Korea; 273 Sackler Faculty of Medicine, Tel Aviv University, Tel Aviv-Yafo, Israel; 274 Neurology, Harvard Medical School, Boston, MA USA; 275 Neurology, Brigham and Women’s Hospital, Boston, MA USA; 276 Massachusetts General Hospital, Boston, MA USA; 277 Department of Nursing, Chungnam National University, Daejeon, 301747 South Korea; 278 Chungnam National University Hospital, Daejeon, South Korea; 279 Kinesiology, Recreation and Sport, Indiana State University, Terre Haute, IN USA; 280 Harvard Medical School, Boston, MA 02215 USA; 281 National Ayurvedic Medical Association of Russia, Novosibirsk, 630108 Russian Federation; 282 Clinical Naturopathy, Charité Institute of Social Medicine, Epidemiologiy and Health Economics, Berlin, 14109 Germany; 283 Charité Institute of Biochemistry, Berlin, 10117 Germany; 284 Institute of Social medicine, Epidemiologiy and Health Economics, Charité University Hospital, Berlin, 14109 Germany; 285 Institute for Social Medicine, Epidemiology and Health Economics, Charité - Universitätsmedizin Berlin, Berlin, 10117 Germany; 286 Psychiatry, Boston University School of Mediicine, Boston, 02118 MA USA; 287 Psychiatry, Harvard University, Belmont, CA USA; 288 Psychiatry, New York Medical College, Valhalla, 10955 NY USA; 289 Psychiatry, Columbia College of Surgeon, New York, NY USA; 290 Department of Pediatrics, Division of Oncology and Hematology, Charité Universitätsmedizin Berlin, Berlin, 13353 Germany; 291 Institute of Public Health, Charité Universitätsmedizin Berlin, Berlin, Germany; 292 Institute for Integrative Medicine, Gemeinschaftskrankenhaus Herdecke, Witten/Herdecke University, Witten/Herdecke, Germany; 293 Center for Evidence-Based Chinese Medicine, Beijing University of Chinese Medicine, Beijing, 100029 China; 294 Guangxi university of Chinese Medicine Affiliated Hospital, Guangxi, China; 295 Faculty of Health and Social Care, London South Bank University, London, UK; 296 Institute for Integrative Medicine, Catholic Kwandong University International St. Mary’s Hospital, Incheon, South Korea; 297 Internal Medicine, Semyung University, Chungju, South Korea; 298 Internal Medicine, Semyung University, Jecheon, South Korea; 299 Korean Medicine, Korea Health Industry Development Institute, Osong, South Korea; 300 Internal Medicine, Kyunghee University, Seoul, South Korea; 301 MOA Health Science Foundation, President, Tokyo, Japan; 302 Osaka University School of Medicine, Integrative Medicine, Osaka, Japan; 303 MOA Health Science Foundation, Tokyo, Japan; 304 Japan Society of Integrative Medicine, Tokyo, Japan; 305 Kampo medicine outpatient clinic, Mie University Hospital, Tsu, 24201 Japan; 306 Pain Clinic, Mie University Hospital, Tsu, 24201 Japan; 307 Department of Family Medicine, School of Medicine, Pontifica Cniversidad Católica de Chile, Santiago, 8330077 Chile; 308 Institute for Social Medicine, Epidemiology and Health Economics, Charité - Universitätsmedizin Berlin, Berlin, Germany; 309 Research Institute Havelhöhe, Berlin, 14089 Germany; 310 Hospital Havelhöhe, Berlin, Germany; 311 Institute for Social Medicine, Epidemiology and Health Economics, Charité University Hospital, Berlin, Germany; 312 Guang’anmen Hospital, China Academy of Chinese Medical Sciences, Beijing, 100053 China; 313 Hebei Medical University Pharmaceutical Research Institute, Shijiazhuang, 050017 China; 314 Beijing University of Chinese Medicine, The Preclinical medicine Institute, Beijing, China; 315 Beijing University of Chinese Medicine, Beijing, 100029 China; 316 Acupuncture, Xiyuan Hospital CACMS, Beijing, China; 317 Brigham and Women’s Hospital, Medicine, Boston, MA USA; 318 Harvard Medical School, Boston, MA USA; 319 Kripalu Center for Yoga & Health, Stockbridge, MA USA; 320 Allina Health, Minneapolis, MN USA; 321 Department of Internal and Integrative Medicine, Centre of Integrative Gastroenterology, Kliniken Essen-Mitte, Faculty of Medicine, University of Duisburg-Essen, Essen, Germany; 322 Department of Internal and Integrative Medicine, Kliniken Essen-Mitte, Faculty of Medicine, University of Duisburg-Essen, Essen, Germany; 323 Australian Research Centre in Complementary and Integrative Medicine (ARCCIM), University of Technology Sydney, Sydney, Australia; 324 Psychosocial Research, Butler Hospital, Providence, 02906 RL USA; 325 Brown University, Providence, RL USA; 326 Rhode Island Hospital, Providence, RL USA; 327 Independent Yoga Consultant, Providence, RL USA; 328 UCSD, San Diego, CA USA; 329 Social and Behavioral Sciences, School of Public Health, University of Memphis, Memphis, 38152 TN USA; 330 Social and Behavioral Sciences, University of Memphis, School of Public Health, Memphis, 38152 TN USA; 331 Exercise, Sport and Movement Science, University of Memphis, School of Health Studies, Memphis, TN USA; 332 Health Solutions, Pune, India; 333 Sharp Health Care, HealthCare, Pune, India; 334 Clinic, Vila Velha, 29102010 Brazil; 335 Instituto do Tratamento do Câncer Infantil – Department of Pediatric Oncology, Faculdade de Medicina da Universidade de São Paulo, Sao Paulo, 05410-03 Brazil; 336 VISAN - Sanitary Medical School of Applied Sciences, Belgrade, 11000 Serbia; 337 Private Practice “Astrocit”, Vrsac, Serbia; 338 Private Practice “Dr Jevdic”, Vrsac, Serbia; 339 Special Neuropsychiatric Hospital “Dr S. Bakalovic”, Vrsac, Serbia; 340 Health care Center “1. Oktobar”, Plandiste, Serbia; 341 Center for Integrative Procedures and Supplements “Dr Dunjic”, Belgrade, Serbia; 342 VISAN - Sanitary Medical School of Applied Sciences, Belgrade, 11000 Serbia; 343 Private Practice “Astrocit”, Vrsac, Serbia; 344 Private Practice “Dr Jevdic”, Vrsac, Serbia; 345 Special Neuropsychiatric Hospital “Dr S. Bakalovic”, Vrsac, Serbia; 346 Health Care Center “1. Oktobar”, Plandiste, Serbia; 347 “Planet Zdravja”, Ljubljana, Slovenia; 348 Center for Integrative Procedures and Supplements “Dr Dunjic”, Belgrade, Serbia; 349 VISAN - Sanitary Medical School of Applied Sciences, Belgrade, 11000 Serbia; 350 Private Practice “Astrocit”, Vrsac, Serbia; 351 Private Practice “Dr Jevdic”, Vrsac, Serbia; 352 Special Neuropsychiatric Hospital “Dr S. Bakalovic”, Vrsac, Serbia; 353 Health Care Center “1. Oktobar”, Plandiste, Serbia; 354 “Planet Zdravja”, Ljubljana, Slovenia; 355 Center for Integrative Procedures and Supplements “Dr Dunjic”, Belgrade, Serbia; 356 VISAN - Sanitary Medical School of Applied Sciences, Belgrade, 11000 Serbia; 357 Private Practice “Astrocit”, Vrsac, Serbia; 358 Private Practice “Dr Jevdic”, Vrsac, Serbia; 359 Special Neuropsychiatric Hospital “Dr S. Bakalovic”, Vrsac, Serbia; 360 Health Care Center “1. Oktobar”, Plandiste, Serbia; 361 “Planet Zdravja”, Ljubljana, Slovenia; 362 Center for Integrative Procedures and Supplements “Dr Dunjic”, Belgrade, Serbia; 363 VISAN - Sanitary Medical School of Applied Sciences, Belgrade, 11000 Serbia; 364 Private Practice “Astrocit”, Vrsac, Serbia; 365 Private Practice “Dr Jevdic”, Vrsac, Serbia; 366 Special Neuropsychiatric Hospital “Dr S. Bakalovic”, Vrsac, Serbia; 367 Health Care Center “1. Oktobar”, Plandiste, Serbia; 368 “Planet Zdravja”, Sarajevo, Bosnia and Herzegovina; 369 Center for Integrative Procedures and Supplements “Dr Dunjic”, Belgrade, Serbia; 370 Mayo Clinic, Rochester, 55905 NY USA; 371 Institute of Public Health, University of Copenhagen, Copenhagen, 1353 Denmark; 372 Naturopathy & Complementary Medicine, Carl Remigius Medical School, Idstein, 65510 Germany; 373 Palliativstation, Warnow-Klinik Bützow, Bützow, 18246 Germany; 374 Mayo Clinic, General Internal Medicine, Rochester, NY USA; 375 Pediatric Gastroenterology, Poznan Medical University, Poznan, 60-572 Poland; 376 Department of Psychology, University of Edinburgh, Edinburgh, UK; 377 Pediatric Gastroenterology, Poznan Medical University, Poznan, 60-572 Poland; 378 Institute of Pathophysiology, Medical University Graz, Graz, Austria; 379 Department of Traumatology, University of Linz, Linz, Austria; 380 Department of Radiology, University of Linz, Linz, Austria; 381 Sports Science, University of Salzburg, Salzburg, Austria; 382 Department of Rheumatology, Dongfang Hospital, Beijing University of Chinese Medicine, Beijing, China; 383 Emergency Department, Xiyuan Hospital, China Academy of Chinese Medical Science, Beijing, China; 384 Department of Rheumatology, Beijing Hospital of Traditional Chinese Medicine, Beijing, China; 385 Department of Nephrology, Dongfang Hospital, Beijing University of Chinese Medicine, Beijing, China; 386 Beijing University of Chinese Medicine, Beijing, 100029 China; 387 Natural Biotechnology, Nanhua University, Dalin, 622 Taiwan; 388 Primary Care, University of Southampton, Southhampton, SO16 5ST UK; 389 Osher Center for Integrative Medicine, BWH/HMS, Boston, MA USA; 390 Physical Medicine & Rehabilitation, Spaulding Rehabilitation Hospital, Harvard Medical School, Boston, MA USA; 391 Sackler Faculty of Medicine, Tel Aviv University, Tel Aviv, Israel; 392 Spaulding Rehabilitation Hospital, Physical Medicine & Rehabilitation, Boston, MA USA; 393 Neurology, Brigham and Women’s Hospital, Boston, MA USA; 394 Beth Israel Deaconess Medical Center, Boston, MA USA; 395 Laboratory of Brain Network Imaging and Modulation, Beth Israel Deaconess Medical Center, Boston, MA USA; 396 Massachusetts General Hospital, Boston, MA USA; 397 Regional Cancer Center Stockholm Gotland, Stockholm, Sweden; 398 St Görans Hospital, Stockholm, Sweden; 399 Karolinska Institutet, Stockholm, Sweden; 400 Center for Complementary Medicine, Institute for Environmental Health Sciences and Hospital Infection Control, Medical Center – University of Freiburg, Freiburg, Germany; 401 North Sweden University Hospital, Umeå, Sweden; 402 Preventive Medicine, College of Korean Medicine, Dongguk University Graduate School, Seoul, South Korea; 403 Preventive medicine, College of Korean Medicine, Dongguk University, Gyeongju-si, South Korea; 404 Beijing University of Chinese Medicine, TCM Diagnostics, Beijing, China; 405 Beijing University of Chinese Medicine, Beijing, 100029 China; 406 The Children’s Hospital at Westmead and Discipline of Paediatrics and Child Health, University of Sydney, Sydney, Australia; 407 Guang’anmen Hospital, China Academy of Chinese Medical Sciences, Beijing, China; 408 Department of Biostatistics and Epidemiology, University of Pennsylvania, Philadelphia, USA; 409 Centre for Evidence-Based Chinese Medicine, Beijing University of Chinese Medicine, Beijing, China; 410 Journal of Traditional Chinese Medicine, Beijing, China; 411 Beijing University of Chinese Medicine, Beijing, China; 412 Department of Oncology, Xiyuan Hospital, China Academy of Chinese Medical Sciences, Beijing, China; 413 China Association of Traditional Chinese Medicine, Beijing, China; 414 Beijing Kawin Technology Share-holding CO., Ltd, Beijing, China; 415 Dongguk University College of Korean Medicine, Physiology, Gyeongju-si, South Korea; 416 Dongguk University College of Korean Medicine, Anatomy, Gyeongju-si, South Korea; 417 Dongguk University, Division of Bioscience, Gyeongju-si, South Korea; 418 National Development Institute of Korean Medicine, Gyeongju-si, South Korea; 419 University of Technology Sydney, Ultimo, 2007 Australia; 420 Catholic Kwandong University Int St.Mary’s Hospital, Integrative Medicine, Incheon, South Korea; 421 College of Korean Medicine, Semyung University, Korean Internal Medicine, Chungju, South Korea; 422 College of Korean Medicine, Semyung University, Korean Internal Medicine, Jecheon, South Korea; 423 Korea Health Industry Development Institute, Korean Medicine, Osong, South Korea; 424 Catholic Kwandong University Int St.Mary’s hospital, Incheon, South Korea; 425 Duke-NUS Medical School, Singapore, Singapore; 426 National University of Singapore, Singapore, Singapore; 427 OneHeartBeat Percussions, Singapore, Singapore; 428 KK Women’s and Children’s Hospital, Singapore, Singapore; 429 Global Health and Development, Hanyang University, College of Medicine, Seoul, South Korea; 430 Daejeon University, Obstetrics and Gynecology, Daejeon, South Korea; 431 Daejeon University, Daejeon, South Korea; 432 Gachon University, College of Korean Medicine, Seongnam, South Korea; 433 Heliopolis University for Sustainable Development, Medicinal Plants, Nasr City, Egypt; 434 Cairo University, Medicinal Plants, Cairo, Egypt; 435 Guang’anmen Hospital, China Academy of Chinese Medical Sciences, Beijing, China; 436 Beijing University of Chinese Medicine, Beijing, China; 437 Department of Korean Medicine Dermatology, Kyung Hee University Hospital at Gangdong, Seoul, South Korea; 438 Department of Preventive Medicine, Graduate School, Kyung Hee University, Seoul, South Korea; 439 Department of Dermatology of Korean Medicine, Kyung Hee University, Seoul, South Korea; 440 FIH-Berlin, Berlin, Germany; 441 Internal Medicine, Gemeinschaftskrankenhaus Havelhöne, Berlin, Germany; 442 Institute for Integrative Medicine, University Witten/Herdecke, Witten, Germany; 443 Hannover Medical School, Clinic for Rehabilitative Medicine, Hannover, Germany; 444 Society for Clinical Research, Berlin, Germany; 445 Institute for Social Medicine, Epidemiology and Health Economics, Charité University Hospital, Berlin, Germany; 446 FIH-Berlin, Berlin, Germany; 447 Internal Medicine, Gemeinschaftskrankenhaus Havelhöne, Berlin, Germany; 448 Institute for Social Medicine, Epidemiology and Health Economics, Charité University Hospital, Berlin, Germany; 449 Institute for Integrative Medicine, University Witten/Herdecke, Witten, Germany; 450 Dongfang Hospital, Beijing University of Chinese Medicine, Rheumatology, Beijing, China; 451 Community Health Service Center of Yangsong Town, Traditional Chinese Medicine, Beijing, China; 452 Dongfang Hospital, Beijing University of Chinese Medicine, Nephrology, Beijing, China; 453 Dongfang Hospital, Beijing University of Chinese Medicine, Beijing China, Rheumatology, Beijing, China; 454 Dongfang Hospital, Beijing University of Chinese Medicine, Rheumatology, Beijing, China; 455 Xiluoyuan Community Health Service Center, Fengtai District, Beijing, China, Traditional Chinese Medicin, Beijing, China; 456 Jingzhou Central Hospital of Hubei Province, China, Hubei, China; 457 Dongfang Hospital, Beijing University of Chinese Medicine, Beijing China, Endocrinology, Beijing, China; 458 Center for Integrative Medicine, University of Maryland, Baltimore, MD USA; 459 Shanghai University of Traditional Chinese Medicine, Shanghai, China; 460 Department of Neural and Pain Sciences, Dental School, University of Maryland, Baltimore, MD USA; 461 School of Basic Medical, Beijing University of Chinese Medicine, Beijing, 100029 China; 462 School of Basic Medical, Beijing University of Chinese Medicine, Beijing, 100029 China; 463 Family Medicine, University of Michigan, Ann Arbor, MI 48105 USA; 464 Pusan National University Korean Medicine Hospital, Internal Medicine, Yangsan-si, South Korea; 465 Department of Urology, Pusan National University Yangsan Hospital, Yangsan, South Korea; 466 S. Maugeri Fondation, Pavia, 27100 Italy; 467 S. Feliciano Hospital, Outpatient Oncology, Rome, Italy; 468 School of Heathcare, University of Leeds Alumnus, Rochdale, OL12 7RX UK; 469 Nursing, Faculty of Health Sciences/University of Granada, Granada, 18971 Spain; 470 Physiotherapy Unit, University Hospital San Cecilio/Health Andalusian Service, Granada, Spain; 471 Fundació Sànitaria de Mollet, Surgery, Barcelona, Spain; 472 Hospital de Viladecans, Barcelona, Spain; 473 Fundació Sànitaria de Mollet, Surgery, Barcelona, Spain; 474 Hospital Royo Villanova, Zaragoza, Spain; 475 Hospital Centre of Integrated Medicine, Hospital of Pitigliano ASL Sudest Toscana, Grosseto, 58100 Italy; 476 Referring Centre for Phytotherapy, Center for Integrative Medicine, Careggi Universitary Hospital, Florence, 50139 Italy; 477 Tuscan Network of Integrative Medicine, Florence, Italy; 478 Royal London Hospital for Integrated Medicine, UCLH NHS Trust, London, WC1N 3HR UK; 479 UCH Cancer Clinical Trials Unit, London, UK; 480 University of York, York, UK; 481 University Hospital Zurich, Zurich, Switzerland; 482 Royal Marsden Hospital, London, UK; 483 University of Southampton, Southampton, UK; 484 IOMED, Lecce, Italy; 485 Instituto do Tratamento do Câncer Infantil – Department of Pediatric Oncology, Faculdade de Medicina da Universidade de São Paulo, Sao Paulo, 05410-030 Brazil; 486 Department of Pediatric Oncology, Charité University Medicine, Berlin, Germany; 487 Bastyr University, Kenmore, WA 98028-4966 USA; 488 Australian Research Centre in Complementary and Integrative Medicine, University of Technology, Sydney, Australia; 489 Nuffield Dept of Orthopaedics, Rheumatology & Musculoskeletal Sciences, University of Oxford, Oxford, UK; 490 The Canadian College of Naturopathic Medicine, Toronto, Canada; 491 Faculty of Nursing, University of Iceland, Reykjavik, 105 Iceland; 492 Shiraz University of Medical Sciences, Shiraz, 71747637 Islamic Republic of Iran; 493 Alchemy Health & Wellbeing, Miami, 4220 Australia; 494 Regional Cancer Center Stockholm/Gotland, Stockholm, SE-102 39 Sweden; 495 Department of Neurobiology, Caring Sciences & Society, Karolinska Institutet, Huddinge, SE-14183 Sweden; 496 Department of Neurobiology, Caring Sciences & Society, Karolinska Institute, Huddinge, SE-14183 Sweden; 497 Hospital San Juan de Dios, Internal Medicine, Santiago de Chile, Chile; 498 Centro de Estudios e Investigación Terapéutica, Santiago de Chile, Chile; 499 Center for Complementary Medicine, University Medical Center Freiburg, Freiburg, 79106 Germany; 500 Chinese Medicine Department, Hospital Authority, Hong Kong, Hong Kong; 501 University of Washington, Seattle, WA USA; 502 University of Queensland, Brisbane, Australia; 503 Southwest College of Naturopathic Medicine, Tempe, AZ USA; 504 Management Policy, National Development Institute of Korean Medicine, Gyeongsan-si, 38540 South Korea; 505 Naturopathy, University of Medicine, Rostock, 18057 Germany; 506 Urology, University of Medicine, Rostock, 18057 Germany; 507 Pediatrics of Korean Medicine, Kyung Hee University Hospital at Gangdong, Seoul, South Korea; 508 Mental Health, CMI (Clinica Medicina Integrativa), Madrid, 28015 Spain; 509 Department of Medical Oncology, Kanazawa Medical University, Uchinada, Ishikawa 920-0293 Japan; 510 Phytomedicines Supply and Development Center, Steigerwald Arzneimittelwerk GmbH, Bayer Consumer Health, Darmstadt, 64295 Germany; 511 Department of Medical Cell Biology, Philipps-University Marburg, Marburg, Germany; 512 Center for Endoscopy Starnberg, Starnberg, Germany; 513 Großhadern Hospital, University of Munich, Munich, Germany; 514 Naturopathy, Center for Internal Medicine, University of Medicine, Rostock, Germany; 515 University of Applied Sciences, Leiden, 2333CK Netherlands; 516 Irvine School of Medicine, Irvine, University of California, Irvine, CA USA; 517 Internal Medicine and Integrative Medicine, University of Duisburg-Essen, Essen, Germany; 518 Institute for Social Medicine, Epidemiology and Health Economics, Charité University Hospital, Berlin, Germany; 519 National Research Center in Complementary and Alternative Medicine (NAFKAM), Department of Community Medicine, Faculty of Health Sciences, The Arctic University of Tromsø, Tromsø, 9037 Norway; 520 Department of Midwifery, Istanbul Medipol University, Istanbul, 34083 Turkey; 521 Delivery Room, Istanbul Bahcelievler Public Hospital, Istanbul, 34180 Turkey

## P44 Expectancy did not predict treatment response in a randomised sham-controlled trial of acupuncture for menopausal hot flushes

### Carolyn Ee^1^, Sharmala Thuraisingam^2^, Marie Pirotta^2^, Simon French^3^, Charlie Xue^4^, Helena Teede^5^

#### ^*1*^*National Institute of Complementary Medicine, Western Sydney University, Sydney, Australia;*^*2*^*Department of General Practice, University of Melbourne, Melbourne, Australia;*^*3*^*School of Rehabilitation Therapy, Queens University, Ontario, Canada;*^*4*^*School of Health and Biomedical Sciences, RMIT University, Melbourne, Australia;*^*5*^*School of Public Health and Preventive Medicine, Monash University, Melbourne, Australia*

##### **Correspondence:** Carolyn Ee

**Background**

Evidence on the impact of expectancy on acupuncture treatment response is conflicting. We conducted a secondary analysis of a randomised sham-controlled trial on acupuncture for menopausal hot flushes. We aimed to evaluate whether baseline expectancy is correlated with hot flush score at end-of-treatment, and determine factors associated with baseline treatment expectancy.

**Methods**

Women experiencing moderate-severe hot flushes were randomised to receive real or sham acupuncture for eight weeks. We measured expectancy using the Credibility and Expectancy Questionnaire immediately after the first treatment, and hot flush score using a seven-day hot flush diary. A complete mediation analysis using linear mixed-effects models with random intercepts was performed, adjusted by baseline hot flush score, to identify associations between independent variables, expectancy levels and hot flush score at end-of-treatment. Because there was no difference between real and sham acupuncture for the primary outcome of hot flush score, both arms were combined in the analysis (n = 285).

**Results**

Treatment credibility, perceived allocation to real acupuncture or uncertainty about treatment allocation, and previous positive response to acupuncture predicted higher baseline expectancy. There was no evidence for an association between expectancy and hot flush score at end-of-treatment. Hot flush scores at end-of-treatment were 8.1 (95%CI, 3.03 to 13.20; P = 0.002) points lower in regular smokers compared to those who had never smoked.

**Conclusion**

In our study of acupuncture for menopausal hot flushes, we did not find an association between expectancy levels and treatment outcome. The association between smoking and improvement in hot flushes warrants further exploration.

This trial was registered with the Australia New Zealand Clinical Trials

Registry ACTRN12611000393954 on 14/04/2011

## P45 Prevalence and associations for use of Complementary and Alternative Medicine (CAM) among people diagnosed with, or with a family risk for Coronary Heart Disease (CHD) in the 6th Tromsø Study

### Agnete E Kristoffersen^1^, Fuschia Sirois^2^, Trine Stub^3^

#### ^*1*^*NAFKAM, UiT, The arctic university of Norway, Tromsø, 9019, Norway;*^*2*^*Department of Psychology, University of Sheffield, Sheffield, S1 1HD, United Kingdom;*^*3*^*Department of community medicine, UiT The arctic university of Norway, Tromsø, 9019, Norway*

##### **Correspondence:** Agnete E Kristoffersen

**Aim**

The aim of this study was to examine prevalence and associations for use of CAM among People diagnosed with, or with a family risk for CHD in the 6th Tromsø Study.

**Methods**

A total of 12982 men and women (response rate 65.7%) filled in a self-administrated questionnaire with questions about life style and health issues. Eight hundred and thirty of those had been diagnosed with either heart attack and/or angina pectoris while 4830 had a family risk for such disease due to close family members diagnosed with such disease.

**Results**

CAM use (CAM provider, OTC CAM products and/or CAM techniques) was found in 30.2% of the participants diagnosed with CHD and 35.8% of the participants with family risk of CHD (p < 0.001). Self-rated health, expectations for future health, preventive health beliefs and health behaviour were significant predictors of CAM use for those at risk of CHD. In the CHD patient group only health behaviour and self-rated health were significantly associated with CAM use.

**Conclusion**

Risk for CHD disease seems to be a stronger predictor for CAM use than the diagnosis of CHD itself.

## P46 Addressing training needs of general practitioners working with cancer patients – trial protocol

### Jennifer Engler^1^, Stefanie Joos^2^, Corina Güthlin^1^

#### ^*1*^*Institue of General Practice, Goethe University Frankfurt, Frankfurt, 60590, Germany;*^*2*^*Department of General Practice, University of Tübingen, Tübingen, Germany*

##### **Correspondence:** Jennifer Engler

**Background**

During the years 2012–2015 we collected data on training needs of health care personnel working with cancer patients within the research network KOKON. 70% of general practitioners felt it was important for their work to stay informed about CAM, but only 31% felt safe when talking about CAM options. GPs wished to get more information on a variety of CAM options with homeopathy, mistletoe and mind-body-techniques being of highest interest. In a separate study within KOKON it was shown that not only information on CAM but also the issue of how to communicate CAM options mattered to physicians during their training on CAM. Therefore, we plan to offer blended learning training to GPs (e-learning courses on CAM options, i.e. benefits and potential problems AND communication training). We will show the training and the evaluation concept.

**Methods**

We will conduct an exploratory pilot study with wait-list controlled design in order to study feasibility of the training concept in GPs, but also effects of the training by analyzing real-life communication using vignettes. Roter Interaction Analysis System (RIAS) will be used to evaluate communication training. 15 GPS will be trained first and 15 GPs will be trained after finishing waitlist-control.

**Results**

The trial protocol will be presented in detail.

**Conclusion**

Training on CAM options will not suffice unless not incorporated into communication training. We will show an innovative blended-learning concept and an evaluation design explicitly addressing both aspects adequately.

## P47 Treatment of patients with digestive disorders with *Gentiana Magen Globuli velati* (*GMGv*) – results from a non-interventional study

### Jennifer Felenda^1^, Christiane Beckmann^1^, Florian Stintzing^2^

#### ^*1*^*Med.-pharm. Information / Klinische Forschung, WALA Heilmittel GmbH, Bad Boll/ Eckwälden, 73087, Germany;*^*2*^*Ressortleitung Wissenschaft, WALA Heilmittel GmbH, Bad Boll/ Eckwälden, 73087, Germany*

##### **Correspondence:** Florian Stintzing

**Background**

*GMGv* is an anthroposophical drug used to harmonize the motility and secretion of the gastrointestinal tract, containing *Artemisia absinthium*, *Gentiana lutea*, *Taraxacum officinale* and potentized *Strychnos nux-vomica*. In Europe the prevalence of unspecific digestive disorders reaches 14% to 25% [1]. According to physicians” expertise, *GMGv* is an effective medicine, especially for treatment of dyspepsia with the main symptoms digestive weakness, gastric pressure and anorexia [2].

**Methods**

A national non-interventional study observed 319 adult patients with digestive disorders treated with *GMGv*. 29 physicians assessed the individual treatment considering diagnostic findings and patients’ preference.

The main aim was to evaluate the application modalities of *GMGv*: diagnosis, reason of application, duration of therapy and dosage. Secondly, the course of therapy and the patient’s acceptance were monitored.

**Results**

The intention-to-treat analysis of 292 out of 319 patients (71% females, mean age 47.3 years) showed patients suffering from functional dyspepsia (34%), psychosomatic disorders (21%), colon irritable (13%) and gastroenteritis (9%). The physicians prescribed *GMGv* to 53% of the patients as single therapy and to 27% because of insufficient efficacy of other drugs, mainly 3×5-10 globules/day for over 22 days. 68% of the patients were cured after completion of the treatment.

Patients’ status of health, evaluated by development of symptoms, global general condition, severity level of the main diagnosis, improved statistically significant.

151 of 195 patients (78%) were satisfied with the treatment and 143 of 165 (87%) would use *GMGv* again.

**Conclusion**

*GMGv* reduce symptoms and the severity level of the main indication, e.g. digestive disorders and improve the global general condition of the patients.

**References**

1. Mahadeva S., Goh KL. Epidemiology of functional dyspepsia: a global perspective. World Journal of Gastroenterology, 2006. 12: 2661–2666

2. Felenda J., Beckmann C., Stintzing F., Meyer U. Gentiana Magen Globuli velati in der ärztlichen Praxis- Ergebnisse einer Umfrage. Der Merkurstab, 2012. 65: 465–470

## P48 Virtual reality environments for supporting mindfulness based healing of low back pain

### Roni Evans^1^, Gert Bronfort^1^, Daniel Keefe^1^, Anna Taberko^2^, Linda Hanson^1^, Alex Haley^1^, Haiwei Ma^1^, Joseph Jolton^2^, Lana Yarosh^1^, Francis Keefe^1,3^, Jung Nam^1^

#### ^*1*^*University of Minnnesota, Minneapolis, 55455, MN, United States;*^*2*^*Minneapolis College of Art and Design, Minneapolis, MN, United States;*^*3*^*Duke University, Durham, NC, United States*

##### **Correspondence:** Roni Evans

**Background**

Low back pain (LBP) is among the most common and burdensome pain conditions worldwide. Mindfulness based interventions (MBIs) are a promising form of self-management which can help LBP patients develop the skills to self-regulate pain responses. However, similar to other self-management interventions, it can be difficult to motivate participants to engage in MBIs. Virtual Reality (VR) can provide individualized, appealing, and immersive environments that motivate patients to practice mindfulness. Our group is conducting a pilot study. One of the questions it will answer is: Can a multi-sensory VR intervention be designed to facilitate engagement in mindfulness practices in patients with LBP?

**Methods**

A multidisciplinary group of individuals from the fields of mindfulness, psychology, computer science, clinical research, and art and design is using an adapted intervention mapping approach to develop a prototype VR-MBI intervention. A total of 10 participants with chronic, mechanical LBP will use the VR-MBI for 8 weeks. Self-report outcomes (e.g. pain, disability, fear-avoidance, mindfulness, medication use) will be measured pre- and post- intervention; engagement in VR-MBI will be measured via diary and satisfaction, barriers, and facilitators will be assessed through qualitative interviews.

**Results**

A series of iterative design prototypes including imagery, narration, 3D models, and user interface techniques has been created to explore options for the VR environment that can best support mindfulness practices around pain. Special attention has been given to color, forms, and visual texture to align with the pain narrative, mindfulness exercises, and target outcomes. Patient enrollment and data collection will be completed by 2017.

**Conclusions**

As VR becomes increasingly affordable and available, it could become a very practical and engaging home-based self-management intervention for patients with persistent pain and other chronic conditions.

## P49 Understanding facilitators and barriers to compassionate technology

### Roni Evans^1^, Liwanag Ojala^2^, Mary J Kreitzer^1^, Linda Hanson^1^

#### ^*1*^*University of Minnesota, Minneapolis, 55455, MN, United States;*^*2*^*Caring Bridge, Minneapolis, MN, United States*

##### **Correspondence:** Roni Evans

**Background**

Established in 1997, CaringBridge is a web-based social network serving over 500,000 people daily and over 43 million people annually in over 230 countries. By using personal, protected websites for communities to share, connect, and rally support for people navigating challenging health journeys, CaringBridge sites offer a space for compassion, the natural emotional response to suffering and authentic desire to help. Our overall objective is to use technology to enhance social connectedness, and bring people together to help overcome the isolation that comes with illness. One of our questions examines What are the key characteristics, facilitators, and barriers of compassionate technology that can inform future optimization of web based social networks?

**Methods**

As part of a larger multi-phase project addressing the range of stakeholders, we conducted an electronic qualitative survey using Qualtrics. This survey was sent to CaringBridge staff and leadership and queried individuals regarding their perceptions of why people use CaringBridge, barriers to engagement, and unique characteristics of the CaringBridge experience and platform. Content analysis of text responses was performed to identify major themes.

**Results**

A total of 38/62 (62%) individuals affiliated with CaringBridge responded to the survey. Eight individuals each identified themselves as having used CaringBridge themselves as a patient (21%) or a caregiver (21%); 84% also identified as active visitors. Perceived motivators to using CaringBridge included ease of communication and the opportunity to see and feel much needed social support. Potential barriers to using CaringBridge included discomfort with technology, privacy concerns, a lack of awareness or understanding the goals of the site, and not knowing how to participate or what to say. Among the unique features mentioned was the journal component, where patient authors share their thoughts and emotions, and be vulnerable in a safe space, cultivating a powerful sense of community and shared experience.

**Conclusions**

As expressed by key stakeholders, compassionate technology sites, like CaringBridge, can meet important social needs associated with serious illness. Through identification of facilitators and barriers, implementation and accessibility of this potentially impactful social networking resource can be optimized for greater reach.

## P50 Althaea officinalis cough preparations: Data from two independent consumer surveys underlining efficacy and tolerability for the treatment of irritative cough

### Careen Fink^1^, Karin Kraft^2^

#### ^*1*^*Phytomedicines Supply and Development Center, Innovation & Development, Bayer Consumer Health Steigerwald Arzneimittelwerk GmbH, Darmstadt, 68526, Germany;*^*2*^*University Medicine of Rostock, Rostock, 18055, Germany*

##### **Correspondence:** Careen Fink

**Background**

Cough preparations containing aqueous Marshmallow root extracts (Althaea officinalis) are well established as medicinal products in Germany. Measuring the consumer experience and satisfaction is the key factor to their understanding. Especially for non-prescribed over-the-counter (OTC) medication, the experiences and needs of consumers are the key resource for information

**Material and Methods**

Two independent prospective, non-interventional pharmacy surveys were performed in German pharmacies on consumers buying either lozenges (winter season 2014/15) or a syrup (winter 2015/2016) of the aqueous Marshmallow root extract STW42. They were requested to complete a questionnaire on the course of cough symptoms, onset of effect, global assessment of efficacy, and tolerability during a 7 days treatment.

**Results**

These two prospective, non-interventional pharmacy surveys focussed on creating a better understanding of the patient’s impression of the efficacy, tolerability and satisfaction. A total of 822 questionnaires (syrup: n = 516, lozenges n = 306) consumers were evaluated. The median duration for recovery from symptoms was five days. Relief of oral or pharyngeal irritation and associated dry cough started within 10 min after application in >58% of the consumer, efficacy was rated at least as good by >83%.

The studies confirmed a good efficacy for the symptomatic treatment of oral or pharyngeal irritation and associated dry cough with a very quick onset of action: in the majority of cases the effect was stated within 10 minutes or less.

**Conclusion**

The surveys justify the long-standing use of Marshmallow preparations in cough treatment, and confirm the excellent tolerability of corresponding preparations.

## P51 The RUTI trial: a multi-centred, 16 week, randomised, double blind, placebo controlled feasibility study of Chinese herbal medicine (CHM) for women with Recurrent Urinary Tract Infections (RUTIs)

### Andrew Flower^1^, George Lewith^1^, Kim Harman^1^, Beth Stuart^1^, Felicity L Bishop^2^

#### ^*1*^*Primary Care and Population Sciences, University of Southampton, Southampton, United Kingdom;*^*2*^*Psychology, University of Southampton, Southampton, United Kingdom*

##### **Correspondence:** Andrew Flower

**Background**

Recurrent urinary tract infections (RUTIs) are an unpleasant, disabling and expensive condition affecting an estimated 3–4% of adult women. Usual treatment involves long-term antibiotics. The growing threat of antimicrobial resistance has resulted in an interest in herbal alternatives to antibiotics. Chinese herbal medicine (CHM) may be a useful treatment for RUTIs. This trial explores the feasibility of CHM for RUTIs in UK primary care with particular reference to (1) obtaining UK regulatory approval for CHM as a Clinical Trial of an Investigational Medicinal Product (CTIMP); (2) comparing the effect sizes of individualised CHM, standardised CHM and placebo treatments; (3) investigating the feasibility and acceptability of administering CHM in UK GP practices; (4) exploring the impact of different therapeutic environments on contextual treatment effects.

**Methods**

16 week, multi-site, double blinded, randomized, placebo controlled, mixed methods, pilot/feasibility trial, involving up to 80 women aged between 18–65 designed to compare standardized CHM remedies administered via GP practices, with individualized CHM formulae administered by an experienced TCM practitioner. Feasibility outcomes include the viability of developing, implementing and evaluating a CHM intervention within UK primary care with respect to recruitment, referral patterns, patient compliance, the use of a herbal placebo, and drop out rates. Quantitative outcomes included changes in the duration, severity and recurrence of acute UTI. Qualitative outcomes will explore the experience of patients and practice nurses during the delivery of CHM.

**Results**

After a protracted process MHRA and NHS regulatory approval was obtained for the first Clinical Trial of an Investigational Medicinal Product (CTIMP) of CHM in the UK. In total so far 41 primary care sites have participated in the identification of recruits in the individualised arm recruiting 21 women; and 18 sites have been set up (with 6 active) in the standardised arm recruiting 15 women.

**Conclusion**

We have shown it is feasible to conduct a CTIMP trial in the UK and to recruit via NHS primary care sites. Important lessons have already been learned that could inform a more definitive future trial. A full quantitative and qualitative analysis of the final data will be available in March 2017.

## P52 Consultations with complementary medicine practitioners and childhood vaccine uptake: is there cause for concern

### Jane Frawley

#### *Faculty of Health, University of Technology Sydney, Sydney, 2007, Australia*

**Background**

Is there an association between complementary medicine (CM) use and vaccine uptake in Australian children? Data from the US and Canada demonstrates that parents who use CM are more likely to delay or forgo vaccination, however this association has not been examined in an Australian paediatric population.

**Methods**

An online survey (n = 149) was carried out to determine parent’s attitudes and decision-making regarding their children’s health care needs, including vaccination.

**Results**

A total of 73.8% of children had visited a complementary medicine (CM) practitioner (48.3%) or used a CM product (68.5%) in the previous 12 months. Many children had also consulted a general practitioner (89.9%), community health nurse (31.29%), paediatrician (30.3%), and other medical specialist (38.8%). Children had also visited a naturopath/herbalist (30.4%), chiropractor (18.4%), homeopath (11.6%), nutritionist (6.8%) or traditional Chinese medicine practitioner (8.2%) and 52% of parents did not disclose the use of CM to their primary health care physician. A high number of children (35.6%) were not vaccinated with safety concerns being the most common reason (76.6%). Visits to a CM practitioner (OR 0.16; CI 0.07, 0.36; p < 0.001) and the use of CM products (OR 0.25; CI 0.09, 0.64; p = 0.004) were more likely if childhood vaccinations were not up to date.

**Conclusions**

Poor vaccination uptake is associated with the use of CM in this sample and nationally representative studies are needed to confirm these findings. Research is also needed to determine if these associations are due to CM practitioner attitudes to vaccination or parental health beliefs.

## P53 The effects of modulated electro-hyperthermia on hepatocellular carcinoma in vitro and in vivo models

### Lilla Füleki, Eva Kiss, Tamas Vancsik, Tibor Krenacs

#### *1st Department of Pathology and Experimental Cancer Research, Semmelweis University, Budapest, 1085, Hungary*

##### **Correspondence:** Lilla Füleki

Modulated electro-hyperthermia (mEHT) is an integrative approach to chemo- and radiotherapy. Electromagnetic field and the concomitant heat (<42 °C) can target malignancies due to their elevated ion-concentration (Warburg-effect) and electric conductivity. We tested the effects of mEHT on a hepatocellular carcinoma cell line (HepG2) *in vitro* and *in vivo*.

HepG2 culture was treated *in vitro* three times for 60 minutes (42 °C) during two days. Heat-stress was measured by western blot for Hsp70. Cells were counted for viability in Bürker-chamber using resazurin assay. Tumor cell proliferation was assessed using Ki67 immunocytochemistry. Flow cytometry for annexin-V/propidium iodide (PI) was used to determine the apoptotic per necrotic rates. HepG2 xenografts in SCID mice were treated with mEHT for 30 minutes. Formalin-fixed, paraffin.embedded tumors were tested 24 and 96 hours after treatment using hematoxillin-eosin based morphometry and immunhistochemistry to identify proliferating (Ki67) and apoptotic (cleaved caspase-3) cell fractions.

mEHT treatment *in vitro* resulted in a significant destruction and loss of tumor cells. Elevated release of stress-associated Hsp70 was also observed. Annexin-V/PI measurements confirmed elevated apoptotic cell death rate. Directly after mEHT treatment there was a transitional elevation of resazurin uptake in tumor cells which later decreased to the control level. This phenomenon could be a residual effect on enzyme activity after the thermal impact. The proliferation of the tumor cells was reduced based on the Ki67 assessment. *In vivo*, mEHT treatment induced caspase-3-mediated tumor-destruction and reduced tumor cell proliferation. Further studies are under way to clarify the mechanism of action of mEHT on HEPG2 cells.

## P54 Preliminary findings from an active surveillance reporting system among spinal manipulative therapy providers

### Martha Funabashi^1^, Katherine A Pohlman^1,2^, Silvano Mior^3^, Haymo Thiel^4^, Michael D Hill^5^, David J Cassidy^6^, Michael Westaway^5^, Jerome Yager^1^, Eric Hurwitz^7^, Gregory N Kawchuk^1^, Maeve O’Beirne^5^, Sunita Vohra^1^

#### ^*1*^*University of Alberta, Edmonton, Canada;*^*2*^*Parker University, Dallas, TX, United States;*^*3*^*Canadian Memorial Chiropractic College, Toronto, Canada;*^*4*^*Anglo-European College of Chiropractic, Bournemouth, United Kingdom;*^*5*^*University of Calgary, Calgary, Canada, ;*^*6*^*University of Toronto, Toronto, Canada;*^*7*^*University of Hawaii at Manoa, Honolulu, Hawai’I, United States*

##### **Correspondence:** Martha Funabashi (funabash@ualberta.ca)

**Background**

Patient safety is one of the leading healthcare challenges globally. The aim of this study was to evaluate initial findings from an active surveillance reporting system to identify adverse events (AEs) following spinal manipulative therapy (SMT).

**Methods**

Chiropractors and physiotherapists were recruited for this study. Chiropractors were asked to collect data from 100 consecutive, unique patients and physiotherapists from 50. Data collected included relevant health history, medication list, treatment provided (including SMT), and symptoms before and immediately after SMT. Patients were also asked to describe symptom changes up to one week after the treatment. Any worsened or new symptom following SMT was considered an AE.

**Results**

To date, 4 chiropractors and 2 physiotherapists collected data from 500 patients with 299 patients providing post-treatment data 4.4 days after treatment (average, range: 0–29 days). The most common reason for care was low back pain (24.2%) then neck pain (21.2%). On an 11-point scale, patients reported an average decrease of 0.88 points after treatment. For pre-existing symptoms, 48.3% of patients reported symptom improvement, 47.4% no change in symptoms, and 4.3% reported worsened symptoms. Eighteen patients (3.6%) reported a total of 50 new symptoms after SMT of which 37 were rated as mild, 10 moderate and 3 severe.

**Conclusion**

Although a small percentage of patients reported new or aggravated symptoms, most reported symptom improvement after care. Further investigations are recommended, however, to determine if these new/aggravated symptoms are related to care or reflect the natural history of the presenting conditions.

## P55 Scientific production on the efficacy of osteopathy: a bibliometric analysis from 1980 to 2015

### Isabelle Gaboury, Chantal Morin

#### *Family Medicine, Universite de Sherbrooke, Sherbrooke, J1H 5N4, Canada*

##### **Correspondence:** Isabelle Gaboury

In the last two decades, osteopathy has gained interest among the general population as well as among the scientific community. To better understanding publishing on this topic, we conducted a bibliometric analysis to describe the scientific production of empirical studies on osteopathic interventions, analyze its trends, and identify research gaps.

Articles published between 1980 and 2015 were retrieved from Medline and CINAHL. Keywords used to identify the articles of interest were a combination of osteopath*, manipulation, and treatment or synonyms as well as a list of manual techniques used by osteopaths and terms referring to empirical study designs. Three rounds of reviews were conducted (reviewing titles, abstracts, and full articles) to determine eligibility for inclusion of articles. Bibliometric indicators, such as scientific productivity, research design, and treated conditions were extracted.

3103 articles were retrieved by the literature search, and 426 were considered in the analysis. Scientific production is following an increasing trend, with the number of publications doubling every 5 years. 111 journals published empirical results of osteopathic interventions, with four of the top five journals (representing almost 2/3 of the articles) dedicated to an osteopathic readership. Most popular research designs are randomized controlled trial (37%), followed by case study (26%). Neck, thoracic and low back pain (20%) and visceral-related issues (18%) were the most often treated conditions.

This study identifies trends and gaps that could contribute to the development of rigorous studies evaluating the efficacy of osteopathic interventions. Dissemination strategies are relatively limited to the community of osteopaths.

## P56 Meta-analysis of clinical trials in homeopathy: anything left to learn? – protocol for a new approach

### Katharina Gaertner^1^, Loredana Torchetti^1^, Martin Frei-Erb^1^, Michael Kundi^2^, Michael Frass^3^

#### ^*1*^*Institute for Complementary Medicine, University of Bern, Bern, 3008, Switzerland;*^*2*^*Institute of Environmental Health, Medical University of Vienna, Vienna, 1090, Austria;*^*3*^*Department of Medicine I, Clinical Division of Oncology, Medical University of Vienna, Vienna, 1090, Austria*

##### **Correspondence:** Katharina Gaertner

**Purpose**

A systematic review with meta-analyses of the various homeopathic interventions and their effects in specific pathologies is outstanding.

**Method**

The study protocol provides a new methodological bottom-up approach, including grey literature and observational studies, the assessment of study quality regarding internal, external and model validity as well as a refinement of the overall analysis by gradual detection of differences by means of subgroup and sensitivity analyses. Available studies are allocated into three meta-analyses that try to identify: Clinical effects of homeopathic medicines (HOM) in randomized controlled trials (RCT), in observational studies (OS) and in preventive use; each compared to placebo or to conventional treatment in 9 pathology-based subgroups. An initial set of 535 Studies has been reviewed and screened for inclusion. 1,216 master theses have been identified as suitable for further evaluation. A second literature search, especially for the years 2013–2015, is ongoing.

**Results**

380 studies were included for further data-extraction. 72.5% of the included studies use RCT, 27.5% OS techniques. The studies, which apply HOM for preventive purpose (19.6%) will be analyzed separately. In the study-set of therapeutic use, the investigations of individualized and complex homeopathy count around 35%, clinical homeopathy approximately 25%, and isopathy 5%. In one third of studies the comparator is standard therapy, in two thirds placebo.

**Conclusion**

Investigating clinical studies of HOM with meta-analytical means by subgrouping of homeopathic methods, study designs and pathologies may contribute to a better understanding of the clinical effects of HOM and may open new perspectives for homeopathic research.

## P57 SENeCA Study: observational study on the effect of medicinal cannabis on quality of life and nutritional outcomes

### Eugenia Gallo^1,2,3^, Valentina Maggini^1,2,3^, Mattia Comite^2,3^, Francesco Sofi^1^, Sonia Baccetti^4^, Alfredo Vannacci^1^, Mariella Di Stefano^4^, Maria V Monechi^4^, Luigi Gori^2,3^, Elio Rossi^4^, Fabio Firenzuoli^2,3,4^, Rocco D Mediati^5^, Giovanna Ballerini^5^

#### ^*1*^*Department of Experimental and Clinical Medicine, University of Florence, Florence, Italy;*^*2*^*Center for Integrative Medicine, Careggi Universtary Hospital, Florence, Italy;*^*3*^*Referring Centre for Phytoterapy, Region Tuscany, Florence, Italy;*^*4*^*Tuscan Network of Integrative Medicine, Region of Tuscany, Florence, Italy;*^*5*^*Cure Palliative e Terapia Del Dolore, Careggi Universitary Hospital, Florence, Italy*

##### **Correspondence:** Eugenia Gallo

**Background**

The Tuscany Health System offers the medicinal use of Cannabis especially for the treatment of oncologic or neuropathic chronic pain, resistant to conventional therapy. Our Centre has promoted this retrospective study to evaluate the efficacy and safety profile of Cannabis. In particular, the effects on pain, quality of life (QoL), anxiety, nausea, fatigue, and nutritional assessment of the patients will be taken into account.

**Methods**

Hospital Pharmacy has prepared Cannabis flos (19% THC) dispensed for decoction or as an oil extract. A total of 40 patients (32.5% male/ 67.5% females), age mean di 57,10 who had taken cannabis for 250 days was observed. Clinical data, QoL, lifestyle, drugs use and adverse effects were assessed by administering an appropriate questionnaire to the patients treated with cannabis.

**Results**

All 40 patients (mean age 57,1 ± 13,6 years) have been included in the study. Seventy percent of the oncologic and neurologic patients has received Cannabis for pain relief. Pain symptoms are improved in the 60% of subjects in 10–15 days. QoL is improved in the 50% of patients, in particular for mood and sleep; the abdominal swelling is reduced in the 41% of subjects. Seventy-one percent of patients with nausea is improved, with an increase in weight in 45% of the cases. Adverse effects are reported for the 27% of the patients.

**Conclusions**

Preliminary results of study suggest that Cannabis treatment in patients with conventional treatment-resistant chronic pain may result in improved pain, QoL and appetite as well as reduced opioids and NSAIDs use.

## P58 Lessons learned during the Integrative Medical Group Visits (IMGV): randomized controlled trial for recruiting low-income racial/ ethnic minority research study participants

### Paula Gardiner^1,2^, Anna S Lestoquoy^1^, Lily Negash^1^, Sarah Stillman^1^, Prachi Shah^1^, Jane Liebschutz^1,2^, Pamela Adelstein^3^, Christine Farrell-Riley^3^, Ivy Brackup^4^, Brian Penti^1^, Robert Saper^1,2^

#### ^*1*^*Family Medicine, Boston University Medical Center, Boston, 02118, MA, United States;*^*2*^*Boston University School of Medicine, Boston, MA, United States;*^*3*^*Codman Square Health Center, Dorchester, MA, United States;*^*4*^*Dothouse Health Center, Dorchester, MA, United States*

##### **Correspondence:** Anna S Lestoquoy

**Background**

Integrative Medical Group Visits (IMGV) are an innovative program for delivering chronic pain and depression care. This randomized control trial compares the IMGV model to primary care appointments across three inner city clinics in Boston. Our participants largely identify as racial/ ethnic minorities, a historically challenging population for research recruitment. This poster will share our methods and strategies in overcoming barriers related to recruitment of this patient population.

**Methods**

Participants were recruited through provider referral, warm handoffs (face to face encounters with a research assistant during a clinical session), targeted letters or self-referred after seeing flyers or other study recruitment related materials. Trained research assistants either contacted or were contacted by patients for screening and consenting procedures.

**Results**

A total of 331 patients were consented and screened for inclusion in the study, and 154 enrolled. Seventeen percent of those screened were male; fifty-nine percent identified as Black; with the site specific demographics similar to each site’s patient population. Over the course of the study, in order to ensure a representative sample we changed our recruitment methods to enroll a greater number of male participants. Different patients responded to different recruitment methods, with older patients responding largely to targeted letters and younger patients responding to self-referral and provider referral (p = 0.0003, α = 0.05). The most common reasons for declining participation was not being interested in groups or having scheduling conflicts with the group schedule.

**Conclusion**

Different populations of patients respond to different forms of recruitment. Our varied approaches resulted in successfully recruiting our target number of participants.

## P59 Acupuncture as a complementary technique in assisted reproduction patients in an integrative way: the results from 6 years’ experience

### Isabel Giralt Sampedro^1,2^, Gilda Carvajal^1^

#### ^*1*^*ENERGIMED, Barcelona, 08007, Spain;*^*2*^*Ginecology, Obstetrics and Reproduction, Hospital Universitari Quiron Dexeus, Barcelona, Spain*

##### **Correspondence:** Isabel Giralt Sampedro

We would like to present our work in the Clinic “**Salut de la Dona Dexeus**” in Barcelona over more than 6 years using Acupuncture as a complementary treatment during IVF. Our experience is an Integrative Medicine experience as we are working in a cooperation way in the Fertility Department of this Gynecology Clinic.

Treatments such as In vitro Fertilization (IVF) have always been associated with high monetary costs, anxiety and secondary effects. Because of this, there is a demand to increase success rates while lowering the overall costs. Previous studies on the application of acupuncture with IVF have showed positive effects on pregnancy rates. The combination of these techniques in an Integrative form could be a possible solution for increasing benefits and lowering costs.

In our study 135 women were undergoing IVF accepted Acupuncture treatment during the application. Acupuncture was applied twice before oocyte extraction and in an immediately after embryo transfer or at list within a 24 hour time frame. We compared the results of the Acupuncture group with the results of the IVF performances without Acupuncture.

In our Acupuncture treatments we use some standard points and some other individual points related to the energetic woman disease according to an individualized medicine.

We are currently waiting on the complete study results from our clinic. However there are some results from our preliminary study from which some suggestions can be made: Acupuncture is very useful especially when we apply in women who receive a frozen embryo transfer with a 57% positive response while the global clinical rates in our Clinic are39 % . Results in the Acupuncture group for direct IVF ( embryo fresh ) are not presenting so significant differences.

In our speech we will explain points and techniques in our Acupuncture treatments and how it was combined with the conventional treatments in an Integrative way. We will update the results with new cases because we will continuous working in this issue until the Congress.

## P60 Identifiability of components of complex interventions

### Andreas Gleiss

#### *Center for Medical Statistics, Informatics, and Intelligent Systems, Medical University of Vienna, Vienna, 1090, Austria*

**Background**

During the last years interventions in complementary and alternative medicine (CAM) were increasingly viewed as complex interventions. The ‘active ingredients’, i.e. the intervention’s multiple components, were classified as specific or non-specific, characteristic or incidental, and their possible interactions were discussed. Various trials were conducted in, e.g., homeopathy or acupuncture to shed some light on the importance of these components.

**Methods**

Many of these CAMstudies exhibit the structure of factorial designs. In this class of designs all possible combinations of the levels of two or more treatments occur together. In this talk, complex interventions are viewed as factorial combinations of their components.

**Results**

The application of well-known concepts from experimental design helps to recognize which components or sums or interactions of components are identifiable within a given study design. The question of identifiability, i.e. unique estimability of the components effects from the observed data, arises particularly if some combinations are not or cannot be observed (e.g. individualized homeopathic prescription without consultation). Furthermore, it will be demonstrated, using study designs from published CAM studies, that the factorial design view enables a clear and correct interpretation of the studied components.

**Conclusions**

In conclusion, some recommendations are given concerning the design and analysis of complex intervention studies. Multi-component intervention studies should be designed in such a way that the effects of interest are identifiable and can thus be uniquely estimated from empirical data once the study has been conducted.

## P61 Spicing up warming compresses – the influence of ginger on the perception of warmth and relaxation through warming compresses

### Marie M Gross, Dorothea Brendlin, Jonas Röttger, Wiebke Stritter, Georg Seifert

#### *AG für Integrative Medizin in der Pädiatrischen Onkologie, Charité Universitätsmedizin, Berlin, 13353, Germany*

**Background**

Though external ginger applications such as warming compresses are commonly used in anthroposophical care, practice and clinical application are mainly based on experience. There is little empirical data on their effect. The aim of this study was to separate factors such as quietness, attentiveness and external thermal application from the effectiveness of ginger powder itself.

**Methods**

In a controlled study design we applied different compresses to healthy adults, adding another factor with every application. Physiological and psychometric data was collected and we conducted phenomenological interviews, which were analyzed according to thematic analysis. Here we focused on reported sensation of warmth and relaxation.

**Results**

In comparison to the hot-water compress, the ginger compress had less influence on temperature perception, and this influence was more diverse. During the application warmth developed differently. A second thermal peak was described after moments of chillness, especially during the resting period with the ginger compress, giving a sensation of burning sun.

While participants felt more relaxed, balanced and reported a greater peace of mind after the ginger compress, they experienced an impulse to move, wandering thoughts and a feeling of agitation especially in the resting period during the hot-water compress.

**Conclusion**

The ginger powder seemed to have an impact on the participants perception of temperature, influencing especially the thermal development. Referring to relaxation the ginger powder might have an additional calming influence compared to the hot-water compress.

## P62 Relevance and acceptance of naturopathic and complementary medicine in women suffering from endometriosis

### Noelle Grzanna^1^, Rainer Stange^2^, Peter W Guendling^1^

#### ^*1*^*Complementary Medicine, Hochschule Fresenius, München, 80798, Germany;*^*2*^*Naturheilkunde, Immanuel-Krankenhaus, Berlin, 14109, Germany*

##### **Correspondence:** Noelle Grzanna

**Background**

Endometriosis describes a gynaecological disease that should be regarded as chronic relapsing. It occurs in women of childbearing age. Characteristic of this disease is the appearance of cell aggregates like the endometrium outside their eutopic localization in the uterine cavity. The current estimated prevalence is about 10–15%. In which about 40% of affected women have endometriosis requiring therapy.

The cardinal symptoms of endometriosis are secondary dysmenorrhea, pelvic pain and dyspareunia, the clinical picture is dominated by the localization of endometriosis lesions.

**Objectives**

Survey of the relevance and acceptance of naturopathic and complementary procedures (CAM) with women who are affected by endometriosis.

**Methods**

An anonymous cross-sectional survey with women who have had a secured endometriosis diagnosis. Patients answered a comprehensive questionnaire of 40 questions, either in paper format or as an online version. The questionnaires were distributed in paper format over 14 doctors’ offices and clinics across Germany to potential study participants. The link to the online version of the questionnaire was passed on to women in endometriosis support groups, patients organizations and women’s health centres. The data were subjected to descriptive statistical analysis.

**Results**

133 patients answered the questions. 86.2% of them had already applied one or more naturopathic and / or complementary procedures for the treatment of endometriosis. Where 77.2% applying subjectively as ‘very important’ rated. Dissatisfaction with the conventional endometriosis treatment moved 61.5% of women to apply naturopathic and / or complementary procedures. Next led the symptoms of endometriosis (53.8%) and infertility (46.9%) for this process selection. TCM (51.1%), acupuncture (60.3%) and SART (40.8%), which contains these therapies were used most frequently. Homeopathy (52.7%) and osteopathy (40.5%) were often used as well. 91.8% of women described an improvement in their symptoms and quality of life through the use of CAM.

**Conclusion**

Patients of endometriosis seem to benefit significantly from the use of naturopathic and complementary procedures. Randomised controlled studies to further investigate the effectiveness of naturopathic and complementary procedures are highly warranted.

## P63 Effects of the Qinlingye extraction on PGC-1a and related inflammatory factors in vitro

### Wen Gu^1^, Yan Lu^2^, Jie Wang^3^, Chengcheng Zhang^4^, Hua Bai^5^, Yuxi He^3^, Xiaoxu Zhang^3^, Zhengju Zhang^3^, Dali Wang^3^, Fengxian Meng^3^

#### ^*1*^*Beijing Hospital of Traditional Chinese Medicine , Rheumatism, Beijing, China;*^*2*^*Xiyuan Hospital, Emergency Department, Beijing, China;*^*3*^*Dongfang Hospital, Beijing University of Chinese Medicine, Rheumatism, Beijing, China;*^*4*^*Dongfang Hospital, Beijing University of Chinese Medicine, Nephrology, Beijing, China;*^*5*^*Xiluoyuan Community Health Service Centre, Rehabilitation Department, Beijing, China*

##### **Correspondence:** Fengxian Meng (0614064@163.com)

**Objective**

To study the effect of Qinlingye extraction (QLYE) on PGC-1a, IL-18 and IL-1β in vitro, and to explore the mechanism of inhibiting immune inflammatory injury from hyperuricemic nephropathy.

**Methods**

Human renal tubular epithelial cells (HKC) and macrophage cells in mice (RAW264.7) were cultured respectively. HKC cells were induced by uric acid (UA) in the model group. While stimulated by UA, the administered groups were intervened by high-, middle- and low-dose of QLYE. After 24, 36 and 48 hours intervention, the total RNA and protein were extracted, and the mRNA transcription and protein expression of PGC-1a were detected. RAW264.7 cells were induced by lipopolysaccharide (LPS) in the model group. While stimulated by LPS, the administered groups were intervened by high-, middle- and low-dose of QLYE. After 24, 36 and 48 hours intervention, the total RNA and protein were extracted, and the mRNA transcription and protein expression of IL-18 and IL-1β were detected.

**Results**

(1)HKC cell experiments: In the model groups, the PGC-1a gene expression level was down-regulated at 24th hour, and the significant reduction of PGC-1a protein content were observed at both the 36th and 48th hour. Compared with the model groups, the PGC-1a gene expression level were upregulated in the medium- and high- dosage group at the 36th and 48th hour respectively. At the 36th hour the PGC-1a protein content were elevated in all three herbal groups and at the 48th hour, the PGC-1a protein content were elevated in both medium- and lower- dosage groups respectively. (2)RAW264.7 cell experiments: In the model groups, the IL-1β gene expression level was up-regulated at the 6th hour, the IL-1β and IL-18 gene expression level were up-regulated at the 12th hour. The significant increase of PGC-1a protein content were observed at 6th, 12th and 24th hour. Compared with the model group, the IL-1β gene expression level were down-regulated in the high- dosage group at the 6th hour, the IL-18 gene expression level in high- dosage group and the IL-1β gene expression level in high- and low- dosage groups were all down- regulated at the 12th hour. The IL-18、IL-1β protein content were down-regulated in all three administered groups at the 6th, 12th and 24th hour.

**Conclusion**

The mechanism how Qinlingye extraction inhibit immune inflammatory injury from hyperuricemic nephropathy may be associated with the upregulation of PGC-1a and the inhibition of its related inflammatory factors——IL-18, IL-1β.

## P64 Plasma concentrations of ascorbic acid in a cross section of the German population

### Alexander Hagel^1^, Heinz Albrecht^1^, Claudia Vollbracht^2^, Wolfgang Dauth^3^, Wolfgang Hagel ^4^, Francesco Vitali^1^, Ingo Ganzleben^1^, Hans Schultis^5^, Peter Konturek^6^, Jürgen Stein^7^, Markus Neurath^1^, Martin Raithel^1^

#### ^*1*^*University of Erlangen, Erlangen, 91054, Germany;*^*2*^*Pascoe Naturmedizin, Gießen, 35394, Germany;*^*3*^*Institute of Employment Research, Nuremberg, Germany;*^*4*^*Martha Maria, Nuremberg, Germany;*^*5*^*Synlab, Weiden, Germany;*^*6*^*Thuringia Clinic, Saalfeld, Germany;*^*7*^*Department of Nutritional Medicine, Sachsenhausen , Germany*

##### **Correspondence:** Heinz Albrecht; Claudia Vollbracht

In modern industrialized countries, vitamin C deficiency is commonly conceived to be extremely rare. The aim of the present study was to assess vitamin C levels in the German population.

As part of a consultant-patient seminar on nutrition and food intolerances, patients were asked to participate in the study on a voluntary basis. Blood samples were taken for analysis of serum vitamin C and patients were asked to complete a questionnaire. Vitamin C levels were determined by high performance liquid chromatography.

Of approximately 300 patients attending the seminar, 188 (62.6%) consented to blood sample analysis for vitamin C and 178 (59.3%) answered the questionnaire. Mean vitamin C level was 7.98 mg/L (range 0.5–17.4; reference value 5–15 mg/L). Low plasma levels with risk of vitamin C deficiency (<5 mg/L) was found in 31 (17.4%), and a potential scorbutogenic deficiency (<1.5 mg/L) in 6 subjects (3.3%). Body mass index correlated inversely with vitamin C levels.

In accordance with older studies, potential vitamin C deficiency was found to be common. A high BMI was associated with reduced vitamin C levels, particularly in older females. It is therefore possible, even in modern developed populations, that certain individuals may require a higher intake of vitamin C.

## P65 Intravenous vitamin C in the treatment of allergies: an interim subgroup analysis of a long-term observational study

### Alexander Hagel^1^, Claudia Vollbracht^2^, Martin Raithel^1^, Peter Konturek^3^, Bianka Krick^2^

#### ^*1*^*University of Erlangen, Erlangen, 91054, Germany;*^*2*^*Pascoe Naturmedizin, Gießen, 35394, Germany;*^*3*^*Thuringia Clinic, Saalfeld, Germany*

##### **Correspondence:** Alexander Hagel

**Background**

Oxidative stress in allergic diseases appears to play not only a key factor in the pathogenesis of the disease but also represents a promising therapeutic target. Allergic diseases have been reported as being associated with reduced plasma ascorbate levels, which is a key physiological antioxidant. Hereby it prevents excessive inflammatory reactions without reducing the defensive function of the immune system.

**Method**

An interim analysis of a multicentre prospective observational study was conducted to investigate the change in disease-specific and non-specific symptoms (tiredness/fatigue, sleep disorders, depression, lack of concentration) during adjuvant treatment with vitamin C infusions (Pascorbin®) in 71 patients with allergy-related respiratory or cutaneous indications. Symptoms atbaseline/visit 1 vs.end of observation period were compared. Change in sum scores of the 3 most prominent disease-specific symptoms and the 4non-specific symptoms were calculated.

**Results**

The mean sum score (0–9) of the disease-specific symptoms decreased significantly by 4.71 points between start (5.9) and end (1.2) of treatment (p

**Conclusion**

Our descriptive statistical data suggests that a treatment with intravenous high-dose vitamin C reduces allergy-related symptoms. This observational study provides information on allergy-related vitamin C deficiency, effective vitamin C dosages and relevant outcomes for conducting a controlled clinical study with more definitive, clinically-relevant endpoints.

Trial registration: Clinical Trials NCT02422901

## P66 Mindfulness-based stress reduction for women with breast cancer: an updated systematic review and meta-analysis

### Heidemarie Haller^1^, Petra Klose^1^, Gustav Dobos^1^, Sherko Kümmel^2^, Holger Cramer^1^

#### ^*1*^*Department of Internal and Integrative Medicine, Faculty of Medicine, University of Duisburg-Essen, Essen, 45276, Germany;*^*2*^*Breast Unit, Kliniken Essen-Mitte, Essen, Germany*

##### **Correspondence:** Heidemarie Haller

**Aim**

The aim of this meta-analysis was to systematically update the effectiveness of mindfulness-based stress reduction (MBSR) and mindfulness-based cognitive therapy (MBCT) in women with breast cancer.

**Methods**

Medline/PubMed, Scopus and Central were searched through October 2016 for randomized controlled trials (RCTs) assessing the effects of MBSR/MBCT in adult women with breast cancer. Primary outcomes were quality of life, fatigue and sleep. Stress, depression, anxiety and safety were secondary outcomes. For each outcome, standardized mean differences (SMD) and 95% confidence intervals (CI) were calculated. Risk of bias was assessed using the Cochrane risk of bias tool.

**Results**

Literature search revealed 15 articles on 11 studies including 1772 participants. Overall risk of bias was at least unclear, except for low attrition and low other bias. Compared to usual care, significant short-term effects of MBSR / MBCT were found for quality of life (SMD = 0.21; 95%CI = [0.04│0.39]), fatigue (SMD = -0.28; 95%CI = [−0.43│−0.14]), sleep (SMD = −0.23; 95%CI = [−0.40│−0.05]), stress (SMD = −0.33; 95%CI = [−0.61│−0.05]), anxiety (SMD = −0.28; 95%CI = [−0.39│−0.16]), and depression (SMD = −0.34; 95%CI = [−0.46│−0.21]). Medium-term effects were significant for anxiety (SMD = −0.28; 95%CI = [−0.47│−0.09]) and depression (SMD = −0.26; 95%CI = [−0.47│−0.04]); long term-effects for anxiety (SMD = −0.21; 95%CI = [−0.40│−0.03]). Compared to other active interventions, significant effects were only found for short-term and only for anxiety (SMD = −0.45; 95%CI = [−0.71│−0.18]) and depression (SMD = −0.39; 95%CI = [−0.65│−0.14]). Subgroup analysis revealed no differences between MBSR and MBCT, cancer stages, or women during versus finishing adjuvant treatment. Effects were robust against potential methodological bias. Adverse events were reported insufficiently.

**Conclusions**

There is promising evidence for short-term effectiveness and safety of MBSR/MBCT in women with breast cancer. However, concerning the small number of available RCTs for medium- and long-term comparisons, more research is needed to draw further conclusions.

## P67 Physical and emotional release effects of Neural Therapy: a qualitative analysis of therapeutic mechanisms and health outcomes

### Heidemarie Haller^1^, Felix J. Saha^1^, Anna Kowoll^1^, Barbara Ebner^1^, Bettina Berger^2^, Gustav Dobos^1^, Kyung-Eun Choi^1^

#### ^*1*^*Department of Internal and Integrative Medicine, Faculty of Medicine, University of Duisburg-Essen, Essen, 45276, Germany;*^*2*^*Department of Health, Institute for Integrative Medicine, Witten/Herdecke University, Herdecke, Germany*

##### **Correspondence:** Heidemarie Haller

**Background**

Research on neural therapy is limited and mainly focused on physical outcomes. This study aimed at describing heterogeneous experiences of patients treated with procaine injections.

**Methods**

Maximum variation sampling was used to collect 22 inpatients aged 59.6 ± 14.9 years (81.8% female) undergoing integrative treatment including neural therapy. With 9.4 ± 6.9 diagnoses patients were mostly multi-morbid. Semi-structured interviews were analyzed in MAXQDA using qualitative content analysis.

**Results**

With injection, patients described anesthetic effects and increased local warmth; sometimes followed by vagotonic responses of weakness or dizziness as well as temporary aggravation of existing symptoms or appearance of new, concealed or phantom symptoms. Thereupon, pain and associated symptoms decreased. Treated areas were perceived as more vivid and reintegrated within the body image. In several cases, injections triggered memories with suppressed emotions. Emotional release was often accompanied by weeping and experienced as initially overwhelming. However, recalled images could be perceived from a distant observer perspective helping patients to reevaluate and reintegrate them into a new emotional context. This led to emotional relief, increased pain acceptance and empowerment. Other patients did not report distinct emotional events, but were surprised at the feelings of happiness, confidence and regained enjoyment of life after treatment. Adverse events included pain due to injection, vegetative complaints and emotional turmoil lasting minutes or hours with a maximum of two days.

**Conclusions**

Neural therapy suggested promising effects on various chronic symptoms, improved daily functioning and mental quality of life. Further studies on its specific efficacy and safety are needed.

## P68 Blinding, easier said than done: experiences from trials of Chinese Herbal Medicine

### Lisha He, Han Wang, X. He, C. Gu, Y. Zhang, Linhua Zhao, Xiaolin Tong

#### *Guang’anmen Hospital, China Acadamy of Chinese Medicine Sciences, Beijing, China*

##### **Correspondence:** Linhua Zhao (melonzhao@163.com)

**Background**

With the development of evidence-based medicine, a number of RCTs had been carried out to confirm the efficacy of Chinese Herbal Medicine (CHM). Blinding is a strategy to control measurement bias in clinical trials. But it has certain challenges in the implementation of blinding in placebo-controlled trial of CHM, especially the placebo presentation, so we had made some efforts on it in several studies.

**Methods**

Staffs who had been involved in the procedure of randomization code generation and concealment or drug blinding will work independent of the data collection and analysis. Both participants and investigators were kept in blinding status to the allocations until the trial was completed. For placebo preparation of CHM decoction, the placebo was prepared by 0.9% of the high-dose group in the trial of GQD treatment of diabetes. For placebo preparation of CHM granules, the placebo was prepared by adding bitters, caramel pigment and additives to ensure the same color, appearance and taste as the treatment drug in another trial of GQD. When the control group was treated with Western Medicine, we applied the double-blind double-dummy method. We prepared both simulation agents for CHM granules and western medicine in a trial of Shenzhuo formula (SZF) treatment of DN.

**Results**

Blinding had been strictly implemented and followed in several trials of CHM formula. Some strategies and standard operations were proposed.

**Conclusions**

There are many similarities between trials of CHM and other complementary and alternative medicines. The practical strategies could be shared and inspired by each other.

This study was funded by the Key Project of the National Natural Foundation of China (Grant No: 81430097), and The Innovation Funding for PhD Students at China Academy of Chinese Medical Sciences.

## P69 How to administrate placebo to patients with diabetes in RCTs of Chinese Herbal Medicine? Practicable and ethical issues

### Lisha He, Han Wang, Xinhui He, Chengjuan Gu, Ying Zhang, Linhua Zhao, Xiaolin Tong

#### *Guang’anmen Hospital, China Acadamy of Chinese Medicine Sciences, Beijing, China*

##### **Correspondence:** Lisha He

**Background**

Placebo-controlled design is necessary for evaluation efficacy of interventions. However, it will meet many challenges to administrate placebo in chronic diseases such as diabetes considering routine treatments. Challenges may be more serious in Chinese Herbal Medicine (CHM) researches because of placebo preparation for CHM is quiet difficult, which leads to the deficiency of the implementation of blinding. Maintain patients compliance and relative ethical issues also need to be carefully considered. In our ongoing trial, we made several efforts to make it practicable and ethical.

**Methods**

This study is a randomized, double-blinded, and placebo-controlled clinical trial. 120 diabetes patients were randomly allocated to receive either Gegen Qinlian Decoction (GQD) or placebo for 12 weeks. The HbA1c, FPG, 2hPG, blood lipids, HOMA-IR and HOMA-β were evaluated. The placebo was prepared by adding bitters, caramel pigment and additives to ensure the same color, appearance and taste as the treatment drug. In order to ensure patients compliance, they all received face-to-face lifestyle education, including dietary and exercise education every 4 weeks. A safety assessment will be performed at every 4 weeks to monitor patients’ safety.

**Results**

This study had been approved by the ethics committee of Guang’anmen hospital (Beijing) before initiation. According to the monitoring data, the overall dropout rate is less than 20%. Most of the adverse reactions are mild, including nausea, flatulence and diarrhea etc.

**Conclusions**

In order to evaluate efficacy and safety of GQD, good management strategies to patients often plays an important role in the placebo-controlled CHM clinical trials.

This study was funded by the Key Project of the National Natural Foundation of China (Grant No: 81430097), and The Innovation Funding for PhD Students at China Academy of Chinese Medical Sciences.

## P70 Sample size calculations: perspectives from investigators of Chinese Herbal Medicine

### Lisha He, Han Wang, Xinhui He, Chengjuan Gu, Ying Zhang, Linhua Zhao, Xiaolin Tong

#### *Guang’anmen Hospital, China Acadamy of Chinese Medicine Sciences, Beijing, China*

##### **Correspondence:** Lisha He

**Background**

The sample size calculation is to determine the minimum number of observed cases, which ensure the reliability of research conclusions with enough statistical power. The sample size is determined by several aspects including types of study design, central and dispersion tendency of main outcomes, group allocation ratio, dropout and other attrition rate etc. The clinical research of Chinese Herbal Medicine (CHM) has its own characteristics, we made several efforts in our clinical trial.

**Methods**

We conducted a randomized, double-blinded, and placebo-controlled clinical trial to determine the efficiency of Gegen Qinlian Decoction (GQD) on the treatment of diabetes. The sample size was estimated based on results from our preliminary trial. HbA1c was selected as the primary main point which was also the basis of null and alternative hypothesis for statistics. Preliminary study results showed that the mean difference of HbA1c between the GQD group and the placebo group was between 0.6% and 0.8%. According to the previous results, the standard deviation (SD) of the placebo group was calculated to be 1.02%.

**Results**

Finally, the sample size was estimated to be 50 in each group, with 90% power to detect the 0.66% difference of HbA1c between two groups at the 2-sided significance level of 0.05. Assuming the total attrition rate of 20%, sample size was planned to be 120 totally.

**Conclusions**

To achieve a well-designed trial, investigators should give more attention to sample size estimation. It may involve many efforts from different roles, including investigators, statisticians, monitors, staffs of data management etc.

This study was funded by the Key Project of the National Natural Foundation of China (Grant No: 81430097), and The Innovation Funding for PhD Students at China Academy of Chinese Medical Sciences.

## P71 Acupuncture and related therapies used as add-on or alternative to prokinetics for functional dyspepsia: overview of systematic reviews and network meta-analysis

### Robin ST Ho^1^, Vincent CH Chung^1,2^, Xinyin Wu^2^, Charlene HL Wong^2^, Justin CY Wu^2^, Samuel YS Wong^1^, Alexander YL Lau^2^, Regina WS Sit^1,2^, Wendy Wong^2^

#### ^*1*^*Jockey Club School of Public Health and Primary Care, The Chinese University of Hong Kong, Hong Kong, 852, Hong Kong;*^*2*^*Hong Kong Institute of Integrative Medicine, The Chinese University of Hong Kong, Hong Kong, 852, Hong Kong*

##### **Correspondence:** Robin ST Ho

**Background**

Prokinetics for functional dyspepsia (FD) have relatively higher number needed to treat values. Acupuncture and related therapies could be used as an add-on or alternative. An overview of systematic reviews (SRs) and network meta-analyses (NMA) were performed to evaluate the comparative effectiveness of different acupuncture and related therapies.

**Methods**

We conducted a comprehensive literature search for SRs of randomized controlled trials (RCTs) in eight international and Chinese databases from their inception till November 2015. Methodological quality of included SRs was assessed with the validated AMSTAR (Assessing the Methodological Quality of Systematic Reviews) Instrument. Data from eligible RCTs were extracted for random effect pairwise meta-analyses. NMA was used to explore the most effective form of treatment among acupuncture and related therapies used alone or as an add-on to prokinetics, compared to prokinetics alone.

**Results**

From five SRs, 22 RCTs (n = 1727) assessing various acupuncture and related therapies were included. Methodological quality of included SRs were mediocre. No serious adverse events were reported. Five pairwise meta-analyses showed that manual acupuncture has marginally stronger effect in alleviating global FD symptoms, as compared to domperidone (6 RCTs) or itopride (3 RCTs). There were no significant differences in the following comparisons: moxibustion versus domperidone (3 RCTs); manual acupuncture plus moxibustion versus domperidone (2 RCTs); and electroacupuncture versus itopride (2 RCTs). Results from NMA showed that the combination of manual acupuncture, moxibustion and clebopride has the highest probability (95.0%) of being the best option for alleviating patient reported global FD symptom. This is followed by the combination of manual acupuncture, moxibustion and domperidone (76.1%), clebopride alone (74.5%), manual acupuncture alone (62.6%) and moxibustion alone (62.3%).

**Conclusions**

Combination of manual acupuncture, moxibustion and clebopride might be the most effective treatment for FD symptoms. Patients who are contraindicated for prokinetics may use manual acupuncture and moxibustion as an alternative. Future confirmatory comparative effectiveness trials should compare clebopride with domperidone, when used as an add-on to manual acupuncture and moxibustion. The potential synergistic effect of proton pump inhibitor and acupuncture should also be explored.

## P72 Exploring breast cancer survivors’ use of the internet to find information on complementary medicine

### Michelle Holmes^1^, Felicity Bishop^1^, Lynn Calman^2^

#### ^*1*^*Psychology, University of Southampton, Southampton, SO17 1BJ, United Kingdom;*^*2*^*Faculty of Health Sciences, University of Southampton, Southampton, SO17 1BJ, United Kingdom*

##### **Correspondence:** Michelle Holmes

**Question**

Breast cancer survivors often turn to the internet as an information resource as part of the decision-making process to use complementary and alternative medicine (CAM). The objective was to explore breast cancer survivors’ use of the internet when making decisions about CAM use.

**Methods**

A purposive sample of 11 breast cancer survivors (mean age = 56) who had used the internet to find information on CAM completed a quantitative questionnaire and a qualitative telephone interview. The theory of planned behaviour (TPB) was used to guide interview questions. Framework analysis and descriptive statistics were used.

**Results**

All participants had used some form of CAM after their diagnosis. Themes from the interviews went beyond the standard definitions of the TPB areas. Participants’ cancer diagnosis transformed how they experienced the internet with their perceptions of internet use changing due to their needs as cancer survivors. Despite the lack of approval from their social network and healthcare team surrounding both CAM and internet use, participants used the internet to find information on CAM.

**Conclusions**

Participants’ use of the internet was more complex than can easily be explained by the TPB and was inherently connected to the experience of self-management for the consequences of cancer and its treatment. Healthcare professionals need to be aware that the information available on the internet plays a role in the decision-making process to use CAM, as breast cancer survivors may not disclose their use of the internet to their healthcare team.

## P73 The feasibility of conducting a cluster-randomised trial on patient-reported outcome measures in chiropractic care

### Michelle Holmes^1^, Felicity Bishop^1^, George Lewith^2^, Dave Newell^3^, Jonathan Field^4^

#### ^*1*^*Psychology, University of Southampton, Southampton, SO17 1BJ, United Kingdom;*^*2*^*Primary Medical Care, University of Southampton, Southampton, SO16 5ST, United Kingdom;*^*3*^*Anglo-European College of Chiropractic, Bournemouth, BH5 2DF, United Kingdom;*^*4*^*Back2Health, Southsea, PO4 0DW, United Kingdom*

##### **Correspondence:** Michelle Holmes

**Background**

Patient-reported outcome measures (PROMs) are being increasingly utilised in routine clinical practice. The literature indicates PROMs may impact clinically and psychologically on patients, however there is limited evidence to currently support this. The purpose of this study was to explore the feasibility of conducting a cluster-randomised controlled trial on PROMs in chiropractic clinics for low back pain.

**Methods**

This feasibility study used a mixed methods approach, combining a cluster-randomised controlled trial (n = 8 patients, n = 3 chiropractors), audio recordings of treatment sessions, and qualitative interviews with stakeholders who are involved with PROMs – patients, chiropractors and reception staff (n = 18). Thematic analysis and descriptive statistics were used.

**Results**

55 eligible patients were invited to take part; 46 declined to participate. Of the nine patients registered to take part in the trial, five were lost to follow up. Despite PROMs being routinely used in chiropractic settings, no participants completed the intervention. The qualitative interviews identified improvements for the development of PROMs as an intervention. This included: selecting relevant PROMs, improving patient engagement with PROMs, and educating clinicians over their use.

**Conclusions**

Patients’ and chiropractors’ views on participating in a trial identified several recommendations on the evaluation of PROMs. The study identified that patients and clinicians need to understand the value of PROMs within the process of patient care. Further development of PROMs as an intervention is also necessary, in the choice, application and timing of PROMs, to ensure PROMs are meaningful to patients and chiropractors and improve engagement.

## P74 Use of traditional, complementary and alternative medicine (TCAM) among stroke patients in Yangon, Myanmar

### Win L Htut, Dongwoon Han, Da I Choi, Soo J Choi, Ha Y Kim, Jung H Hwang

#### *Global Health and Development, Hanyang University, College of Medicine, Seoul, 133-791, South Korea*

##### **Correspondence:** Dongwoon Han

**Background**

Stroke is major public health concern worldwide due to its significant motility and morbidity. Lack of comprehensive rehabilitation care and chronic consequences of stroke, long-term care by using Traditional, Complementary and Alternative Medicine (TCAM), is very common in stroke patients. Stroke patients own sense of severity and functional limitation has been proven to be essential in effective rehabilitation care. However, association between patients self-reported severity and choice of TCAM has not been still documented.

**Methods**

A cross-sectional study was conducted among 310 stroke rehabilitation patients attending outpatient clinics of National Rehabilitation Hospital, Yangon, Myanmar. Data was collected by face-to-face interview with structured questionnaires, containing 35items. Descriptive analysis was done and both univariate and multivariable analysis were used to determine the TCAM use among stroke patients according to their different characteristics.

**Results**

70.3% of the patients used TCAM for their symptom management (55.96%). The main reason for using these treatments was their belief on integrated treatment (42.66%). Stroke patients self-reported severity as less severe used high TCAM (p < 0.01). Moreover, presence of high blood pressure (p < 0.01), having some problem in walking (p < 0.01), self-care (p < 0.05) and usual activities (p < 0.01), feeling moderate anxiety (p < 0.01) were significantly associated with higher TCAM usage. But patients without self-reported pain used more TCAM (p < 0.01).

**Conclusion**

Significant proportion of stroke patients is using TCAM because of their belief in the effectiveness of combined treatments. Hence, physicians should be aware of using TCAM and have to understand its potential risk. Furthermore, provide education among their patients.

Keywords: Traditional, complementary and alternative medicines (TCAM), stroke, Myanmar

## P75 Differences in the use of traditional medicine between South Korea and Taiwan: a preliminary study

### Ching W Huang^1,2^, Bo H Jang^1,2^, Fang P Chen^3^, Seong G Ko^1,2^

#### ^*1*^*Department of Preventive Medicine, College of Korean Medicine, Kyung Hee University, Seoul, 02447, South Korea;*^*2*^*Institute of Safety, Efficacy and Effectiveness Evaluation for Korean Medicine, Kyung Hee University, Seoul, South Korea;*^*3*^*Taipei Veterans General Hospital, Taipei, Taiwan*

##### **Correspondence:** Ching W Huang (sunnierlove@gmail.com)

**Background**

As more people realize the important of the traditional medicine, many people are turning to choose traditional medicine (TM) as a primary healthcare treatment. Herein, this study is the first nationwide study which is comparing two countries—South Korea and Taiwan—in terms of the using pattern of traditional medicine.

**Methods**

To compare the TM utilization patterns between South Korea and Taiwan, we used the million sampling data from national health insurance data of each country.

**Results**

There were 138,119 people in South Korea, and 259,897 in Taiwan were recorded to use traditional medicine service more than one time in 2011. In Korea, women, 40y-60y generation and high income level individuals with the highest frequency to use TM service. In Taiwan, women, 20y-40y generation and middle-high income level is the biggest group to use TM service. The two countries did not have significantly different in terms of either number or season of using TM service. But in South Korea, acupuncture is the most common TM treatment while in Taiwan powdered herbal preparations are most often used treatment in TM system.

**Conclusion**

Under the different health insurance system, there were some different patterns of TM use between South Korea and Taiwan. We surmise one of the important influences was the different coverage between the national health insurance systems of each country.

## P76 Investigation and Analysis on the intention of using traditional Chinese medicine in patients with diabetes

### Wenjing Huang, De Jin, Fengmei Lian

#### *Department of Endocrinology, Guang’anmen hospital China Academy of Chinese Medical Sciences, Beijing, 100053, China*

**Correspondence:** Fengmei Lian

**Purpose**

Investigate the treatment intention of diabetics, to learn the development of TCM therapy in the field of diabetes, to provide a basis for clinical medical decision-making.

**Method**

Juneto August 2016,600 questionnaires were sendedto diabetics in Beijing Chao-Yang Hospital (BCYH) and Guang’anmen Hospital of China Academy of Chinese Medical Sciences (CAMH),592 questionnaires were recovery (296 questionnaires each), investigate the intentions and reasons for the choose TCM.

**Results**

In the questionnaires, there were 279 (47.1%) patients had used TCM treatment, 255 (91.4%) patients of them will continue using, while 72.2% of 255 patients who thought TCM treatment have curative effect (including improving blood glucose and symptoms), 68.6% patients thought good safety (including no side effects and can reduce the side effects of Western medicine); there were 24 (8.6%) patients did not intend to continue using TCM treatment, while 29.2% patients of them thought that inconvenient to take TCM,12.5% patients did not like to drinking. 313 (52.9%) patients never had used TCM treatment, but there were 149 (47.6%) of 313 patients would use TCM treatment in the future, they believed that security of TCM is good and TCM could make them better, 164 (52.4%) of 313 patients stilled did not choose TCM treatment, they thought TCM is not curative effect and safety, they felt inconvenient to take TCM and did not like to drink . In CAMH, there were 213 (76.3%) of 279 patients of had used TCM treatment, while 203 (97.7%) of 213 patients prefered to continue using TCM. There were 172 (91.48%) patients of BCYH in 188 patients who did not intend to use TCM treatment.

**Conclusion**

Patients’ intention in hospital of TCM and western medicine hospital maybe related to physician’s medical decision-making and treatment-oriented. Through popularize TCM formula granule and improve the taste, to make more diabetics receive TCM treatment.

## P77 Attitudes and knowledge on herbal medicine among the Koreans: a cross-sectional survey

### Soobin Jang^1^, Kyeong H Kim^1^, Eun K Lee^1^, Seung H Sun^2^, Ho Y Go^3^, Youme Ko^1^, Sunju Park^4^, Bo H Jang^1^, Yong C Shin^1^, Seong G Ko^1^

#### ^*1*^*Kyung Hee University, Seoul, 02447, South Korea;*^*2*^*Sangji University Korean Medicine Hospital, Wonju, South Korea;*^*3*^*Semyung University, Jecheon, South Korea;*^*4*^*Daejeon University, Daejeon, South Korea*

##### **Correspondence:** Soobin Jang

**Background**

The global herbal medicines market is growing by 5–15% in every year. However, regulations on herbal medicines of safety are still insufficient and there are many cases that perceptions of general population about herbal medicines are wrong. This study aimed to investigate the opinions about herbal medicines.

**Methods**

The survey was conducted via Macromill Embrain (http://www.embrain.com), which is the online research company. The questionnaire was developed by five traditional Korean medicine experts, and the questions consisted of attitude toward herbal medicine, and knowledge status about herbal medicines. Statistical analyses were performed by IBM SPSS statistics program ver.18.0. The entire process of this survey was approved by Institutional Review Board of Kyung Hee University (IRB No. KHSIRB1-15-039).

**Results**

Among the total 1,134 participants, 726 (64.0%) responded that they thought herbal medicines is safe and the remaining 408 (36.0%) responded herbal medicines is not safe. The main reasons of their thoughts were uncertainty of origins (342, 82.8% of 408 those who thought that herbal medicines is not safe), anxiety for harmful substances (289, 70.8%), management insufficiency of herbs (235, 57.6%), and adverse events (190, 46.6%). Of the total respondents, only 308 (27.2%) correctly knew that the difference of herbs for medicine and herbs for food.

**Conclusions**

This survey suggests that proper education to correct misunderstanding on herbal medicines is needed. Also, the results of survey will be basis of establishing national policies on herbal medicines.

Keywords: herbal medicines, survey, safety, attitude, knowledge

## P78 Correlations between pulse wave velocity, age, anxiety and depression of cardiologic patients

### Hubert Janik^1^, Natalie Greiffenhagen^1^, Jürgen Bolte^2^, Karin Kraft^1^

#### ^*1*^*Complementary Medicine, University of Medicine Rostock, Rostock, 18057, Germany;*^*2*^*Strandklinik Boltenhagen, Boltenhagen, Germany*

##### **Correspondence:** Hubert Janik

**Background**

It’s known that cardiovascular events and mortality follow to advanced aortic stiffness. High pulse wave velocity (PWV) may provide evidence to aortic stiffness. The aim of this study was to examine correlations between PWV, age and psychometric scores.

**Methods**

PWV of N = 25 male patients (age: 59.2 ± 6.9 years, BMI: 28.2 ± 3.9 kg/m^2^; mean ± SD) was investigated in addition to the standard procedure in a cardiologic rehabilitation clinic. Absolute arrhythmia, pacemaker, treatment with insulin and acute inflammation are some exclusion criteria. The Mobil-O-Graph (IEM, Stolberg, Germany) is a portable 24 h blood pressure recorder with the ability to calculate PWV by use of curve analysis. The Hospital Anxiety and Depression Scale (HADS-D) comprises the dimensions anxiety (A) and depression (D) with a range from 0 to 21 for each dimension.

**Results**

Mean PWV was 8.1 ± 1.2 m/s. Mean outputs of the HADS-D were 7.2 ± 3.5 for A and 5.0 ± 3.4 for D. Spearman’s rank correlation coefficients were (A & D) = 0.76, (A & age) = 0.62, (A & PWV) = 0.58, (D & age) = 0.54, (D & PWV) = 0.55 and (age & PWV) = 0.90, p < 0.01 for all specified correlations.

**Conclusions**

High correlations between age, A, D and PWV indicate that these parameters are important for detection of patients with cardiovascular risk and are relevant for prevention and treatment of cardiovascular diseases.

Informed consent was signed.

## P79 Sense of responsibility for the health and anxiety in patients with Irritable Bowel Syndrome in context of selected dietary restrictions

### Mariusz Jaworski, Miroslawa Adamus, Aleksandra Dobrzynska

#### *Department of Medical Psychology, Medical University of Warsaw, Warsaw, 02-091, Poland*

##### **Correspondence:** Mariusz Jaworski

**Purpose**

Currently, there is a small percentage of studies which analyse the relationship between sense of responsibility for health, and healthy behaviour. Therefore, purpose of this study was to analyse the correlation between the level of sense of responsibility for the health and level of anxiety on the one hand, and selected dietary restrictions on the other hand, in patients with Irritable Bowel Syndrome (IBS). Currently, in the literature review, there is no study about this problem.

**Methods**

Cross-sectional study involving 60 patients with Irritable Bowel Syndrome was carried out. In this study, Information about sociodemographic variables, and standardised measure of psychological variables were collected. The level of sense of responsibility for the health was measured using The Sense of Responsibility for Health Scale (HSRS), but level of anxiety by using The State-Trait Anxiety Inventory (STAI). For calculations there was used the statistical package SPSS version 21.

**Results**

The level of anxiety has a negative correlation with the global level of Sense of Responsibility for Health (HSR), and its two dimensions: Active Involvement (HSR-AI), and Adequate Behaviour (HSR-AB). Moreover, Sense of Responsibility for Health has positive relationship with reduced consumption of vegetables, meat, fried foods and foods that may cause flatulence (e.g. bean, peas).

**Conclusions**

The level of anxiety, and sense of responsibility for health play a key role in human food behaviour in patients with IBS. This has important practical and clinical implications.

## P80 Mind-Body Medicine and Lifestyle modification in Supportive Cancer Care: a cohort study on a day care clinic program for cancer patients

### Michael Jeitler^1^, Jessica Jaspers^2^, Christel von Scheidt^1^, Barbara Koch^1^, Andreas Michalsen^1,2^, Nico Steckhan^2^, Christian Kessler^1,2^

#### ^*1*^*Department of Internal and Complementary Medicine, Immanuel Hospital Berlin, Berlin, 14109, Germany;*^*2*^*Charité - University Medical Center, Berlin, 10117, Germany*

##### **Correspondence:** Michael Jeitler

**Question**

We developed an integrative day care clinic program for cancer patients focusing on Mind-Body Medicine techniques (meditation, yoga, mindfulness), health-promoting lifestyle modification and self empowerment. The intervention program consisted of 7 h once-per-week group sessions over 12 weeks (84 h intervention time in total).

**Methods**

A cohort study design with a waiting group was implemented. Outcome parameters were assessed at the beginning (baseline), at the end of the active program (12 weeks), and at a 6 month follow-up. Patients waiting >4 and <12 weeks before treatment start were allocated to the waiting group and additionally assessed at the start of their day care program. Outcome measures included quality of life (FACT-G, FACT-B/C, WHO-5), fatigue (FACIT-F), depression and anxiety (HADS) and mood states (ASTS). A per protocol analysis using mixed linear models was performed.

**Results**

100 patients were screened on-site for eligibility, of which 86 patients were included into the study. 86 cancer survivors (83% female; mean age 53.7 ± 9.7 years; 49% breast cancer; 7% colon cancer; mean time since first diagnosis 55.06 ± 28.75 months) participated in the program. 62 patients were allocated to the intervention group, 24 patients were allocated to the waiting group (mean waiting time 5 ± 1 weeks). 14 patients in the intervention group discontinued the intervention. 6 data sets were not complete. 66 data sets were included in the final per protocol analysis.

Significant improvements were observed in favour of the intervention group after 12 weeks compared to the waiting group at the end of the waiting list period for quality of life (WHO-5: 3.94 (1.7, 6.1), p = .001; FACT-G: 6.4 (2.3, 10.5), p = .02, FACT-B: 13 (4.5, 21.5), p = .0004), decreased anxiety/depression (HADS -2.8 (−5.3, −0.4, p = .01) and decreased fatigue symptoms (FACIT-F: 6 (1.3, 10.8), p = .02). Results from the 6 month follow-up for the whole study population showed lasting improvement of quality of life. Most practiced Mind-Body techniques were yoga and meditation. The overall effect of the day care clinic program was rated positive from >90% of all participants.

**Conclusions**

This integrative day care clinic program can be considered as an effective means to improve quality of life, fatigue and mental health of cancer patients. Moreover, it appears to have a sustainable effect, which has to be proved in randomized trials.

Trial registration: DRKS00011027

## P81 Research on key techniques of clinical evaluation of qi deficiency and blood stasis syndrome based on literature research and expert questionnaire

### De Jin, Wen-jing Huang, Bing Pang, Feng-Mei Lian

#### *Guang An Men Hospital of China Academy of Chinese Medical Sciences, Beijing, 100053, China*

##### **Correspondence:** Feng-Mei Lian

**Objective**

To explore the essential factors of clinical evaluation in treating qi deficiency blood stasis syndrome (QDBSS) and find out the reasonable way and scientific method of diagnosis and effect appraisal of QDBSS through literature retrieval and expert questionnaire.

**Methods**

A search strategy was designed to select published literature focusing on QDBSS from CNKI database and Wanfang database according to certain. The frequency of key information in these search strategies were calculated, summarized and analyzed by SPSS 19.0. And related apartments physicians mentioned in the literature research results were incorporated into the questionnaire investigation, which physicians in Guang’anmen Hospital, Xiyuan Hospital, and Beijing traditional Chinese medicine hospital were selected to be investigated.

**Results**

The physicians were investigated in these three hospitals from January to March 2015. A total of 440 questionnaires were conducted to face-to-face interviews, and 439 questionnaires were returned (response rate 99.77%). Main representative diseases of QDBSS were coronary heart disease, stroke, lung cancer, and diabetic nephropathy etc. The characteristic signs and symptoms of QDBSS include tingling, fatigue, tongue with teeth marks, etc. The focus of symptoms were switched from qi deficiency syndrome to blood stasis syndrome, with the improvement of physician level. Average duration of QDBSS is 2.82 months. The multiple symptoms improved with four classification methods (42.79%) and all symptoms of the whole syndrome improved with four classification method (24.59%) were mainly selected as a curative effect valuation.

**Conclusion**

Combination with disease, symptom and syndrome is the precondition of curative effect evaluation of QDBSS, and the treatment course is the key to the clinical evaluation process of QDBBS. Meanwhile, symptom index makes up the fundamental elements of curative effect evaluation. The preliminary results reveal the evaluation factors of the key technology of QDBSS, to provide a new idea for the clinical syndrome efficacy evaluation of traditional Chinese medicine.

## P82 Development of monographs for anthroposophic medicinal products, the example of citrus/cydonia

### Miek Jong^1^, Erik Baars^1^, Anja Glockmann^2^, Harald Hamre^2^

#### ^*1*^*Louis Bolk Institute, Driebergen, 3972LA, Netherlands;*^*2*^*IFAEMM at the Witten/Herdecke University, Freiburg, Germany*

##### **Correspondence:** Miek Jong

**Background**

Monographs are condensed reviews of medicinal products that provide information on their pharmaceutical quality, safety and effectiveness. Monographs give guidance to regulators and healthcare professionals. Existing monographs for Anthroposophic Medicinal Products (AMPs) are not adequate according to current standards. The objective of this study is to develop monographs for AMPs, in order to support their scientific and regulatory assessment.

**Methods**

Standards for scientific assessment of safety and effectiveness of AMPs in monographs were developed and implemented, starting with the AMP Citrus/Cydonia. A systematic literature review of existing data on quality, prescription/use, safety and effectiveness were carried out.

**Results**

Searches in EMbase, Pubmed, CAMbase and Anthromedlit demonstrated that Citrus/Cydonia is manufactured and prescribed according to Anthroposophic Medicine (AM) principles, predominantly for the treatment or prophylaxis of hay fever. Citrus Cydonia is used in children (<18 years, n = 417 patients documented), adults (≥18 years, n = 461) and elderly (≥65 years, n = 25). The effectiveness of Citrus/Cydonia for hay fever was supported by three clinical studies and four immunological studies in cell systems. The overall frequency of Adverse Drug Reactions (ADRs) to Citrus/Cydonia in all studies was 1.4% of users (n = 12/879). No serious ADRs were reported.

**Conclusions**

It is possible to develop AMP monographs that reflect the specific characteristics of AM and adhere to contemporary scientific standards for quality, safety and effectiveness documentation. Standards presented are of interest to other whole medical systems such as Homeopathy and Traditional Chinese Medicine.

## P83 Heart rate variability as a predictor of the effectiveness of the *Bupleuri Radix* (saiko) contained in Kampo Medicines

### Mosaburo Kainuma^1^, Aya Murakami^1^, Toshio Kubota^1^, Daisuke Kobayashi^1^, Yasuhiro Sumoto^1^, Norihiro Furusyo^2^, Shin-Ichi Ando^3^, Takao Shimazoe^1^

#### ^*1*^*Community Medicine Education Unit, Graduate School Of Medical Science, Kyushu University, Fukuoka, 812-8582, Japan****/****Department of Clinical Pharmacy and Pharmaceutical Care, Graduate School of Pharmaceutical Sciences,kyushu University, , Fukuoka, Japan;*^*2*^*Department of General Internal Medicine, Kyushu University, , Fukuoka, Japan;*^*3*^*Sleep Apnea Center, Kyushu University Hospital, , Fukuoka, Japan*

**Background**

Kampo prescriptions that include Bupleuri Radix (saiko) are used for neurosis, insomnia, and symptoms that have a strong association with autonomic nervous system disorders, especially for female patients. However, little is known about the objective efficacy of saiko on the autonomic nervous system.

**Methods and Results**

We retrospectively analyzed the heart rate variability (HRV) data of 54 new female patients (≥20 years) who visited our Kampo Medicine Clinic from 2008 to 2013 to determine if the index of HRV as a surrogate marker of autonomic nervous system is related to the efficacy of saiko and thus can be used to determine for which patients saiko is most effective. The HRV data of patients prescribed medicines that included saiko at their initial visit were divided into effective and ineffective groups (27 patients each) based on the recorded changes of symptoms after two weeks. We found that Very Low Frequency (VLF) (0.003–0.04Hz)/Total Frequency (TF) was significantly higher (p = 0.009) and Ultra Low Frequency (ULF)-1 (0.0001–0.0003Hz)/TF was significantly lower (p = 0.006) in the effective group. The cut-off values for VLF/TF and for ULF-1/TF were 0.35 (Sensitivity, 63%; Specificity, 78%; AUC, 0.71) and 0.24 (Sensitivity, 78%; Specificity, 56%: AUC, 0.71).

**Conclusions**

Our data suggest that ULF-1/TF and VLF/TF have potential as predictors of the effectiveness of medicines containing saiko.

## P84 Ethanol exposure in children: food more relevant than phytomedicines

### Olaf Kelber^1^, S Verjee^2^, Eva Gorgus^2^, Dieter Schrenk^2^

#### ^*1*^*Innovation & Development, Phytomedicines Supply and development Center, Steigerwald Arzneimittelwerk GmbH, Bayer Consumer Health, Darmstadt, 64295, Germany;*^*2*^*Food Chemistry and Toxicology, University of Kaiserslautern, Kaiserslautern, 67663, Germany*

##### **Correspondence:** Olaf Kelber (olaf.kelber@bayer.com)

**Background**

Liquid dosage forms of medicinal products are well suitable for children, as they allow to adapt the dose to the age group. But as in many cases they contain ethanol, they have been repeatedly triggering critical questions.

**Aims**

The aim was therefore to assess to which extent medicinal products contribute to the ethanol exposition in this age group, in comparison to the normal uptake with usual food items.

**Methods**

By evaluation of data from the use of herbal medicinal products in liquid form and by generation of a scenario for the exposure by food items based on new analytical data, exposition values for a 6 years old child were estimated.

**Results**

When using herbal medicinal products, in a 6 years old child amounts of ethanol between 70 and 180 mg are applied with a single dose. With 3 times daily dosing this is 210–540 mg. Related to a body weight [b.w.] of 20 kg, this is 10.1 – 27.0 mg/kg b.w. [1].

An evaluation of side effects of these medicinal products, collected in non interventional studies in more than 50.000 children, and of the spontaneous reports from their use in about 3 Mio. children, did not reveal ethanol related side effects.

For evaluation of the uptake of ethanol with food items commonly used in children, the ethanol content of these items was determined by gas chromatography. E.g. in fruit juices, up to 770 mg/L were found, in bakery products up to 1200 mg/100 g [3]. Based on these data a scenario for the mean ethanol exposure was developed, using data on nutritional habits from USA and Germany.

The resulting mean ethanol exposure was 10.3 mg/kg b.w.. Assuming an ethanol exposure in the upper range of this scenario, it was 12.5 – 23.3 mg/kg b.w..

**Conclusion**

According to these data, the ethanol uptake with herbal medicinal products in children is in an order of magnitude comparable to everydays exposure with usual food items.

From this point of view, it is conclusive that no ethanol related side effects due to an exposure exceeding the average daily intake via food can be expected after the use of these medicinal products. The exposure to ethanol resulting from the use of these products therefore is no cause for toxicological concerns.

**References**

1. Kelber O et al. 2008, PharmInd; 70, 1124–1127

2. Kelber O et al. 2016, Wien Med Wochenschr, in press;

3. Gorgus E et al. 2016, J Analyt Toxicol, doi:10.1093/jat/bkw046

## P85 Training in integrative therapies increases self-efficacy in providing non-drug therapies and self-confidence in offering compassionate care

### Kathi Kemper, Ellie Hill

#### *OSU, Blacklick, 43004, OH, United States*

##### **Correspondence:** Kathi Kemper

**Purpose**

This proof of concept project evaluated the feasibility and preliminary impact of training nurses and other health professionals in introductory workshops about complementary therapies.

**Methods**

We conducted a prospective cohort study of training in acupressure, guided imagery, massage, and Reiki on clinicians sense of self-efficacy in providing non-drug therapies, self-confidence in providing compassionate care, and engagement with work. The training was voluntary as was completion of anonymous online pre- and post-trainng surveys.

**Results**

All topics except massage met minimum enrollment numbers; 22 of 24 participants completed follow-up as well as pre-training surveys. All would recommend the training to others and planned changes in personal and professional care. There were significant improvements in self-efficacy in using non-drug therapies, confidence in providing compassionate care, and unplanned absenteeism (P < 0.05 for each).

**Conclusion**

Training in integrative therapies is feasible and associated with significant improvements in clinicians sense of self-efficacy, confidence in providing compassionate care, and engagement with work. Additional studies are needed to determine the impact on quality of care and long-term workforce engagement.

## P86 Engaging in online training in Mind-Body practices has long-term benefits

### Kathi Kemper^1^, Nisha Rao^2^, Gregg Gascon^1^, John Mahan^1^

#### ^*1*^*OSU, Blacklick, 43004, OH, United States;*^*2*^*College of Medicine, OSU, Columbus, 43210, OH, United States*

**Purpose**

Assess the dose-response relationship between the number of hours of online mind-body skills training for health professionals and outcomes one year later.

**Methods**

Among 1438 registrants for online training (including up to 12 hours of training on mind-body practices) between December, 2013 and December, 2015, we analyzed responses from the first 10% who responded to an anonymous online survey by February 1, 2016. Questions included the type and frequency of mind-body practice in the past 30 days and whether the online training had any impact on personal life or professional practice. Standardized measures were used to assess stress, mindfulness, confidence in providing compassionate care, and burnout.

**Results**

The 149 respondents represented a variety of ages and health professions; 55% completed one or more mind-body training modules an average of 14 months previously. Most (78%) engaged in one or more mind-body practices in the 30 days before the survey; 79% reported changes in self-care and 71% reported changes in the care of others as a result of participating. Increasing doses of training were significantly associated with practicing mind-body skills more frequently; increasing practice frequency was associated with less stress and burnout, which were in turn associated with missing less work. Greater practice frequency was also associated with improvements in stress, mindfulness, and resilience, which in turn were associated with increased confidence in providing compassionate care.

**Conclusions**

Online training in mind-body therapies is associated with changes in self-reported behavior; increasing doses of training are associated with more frequent practice which is associated with less stress, burnout, and missing work, and higher levels of mindfulness, resilience and confidence in providing compassionate care. Additional studies are needed to compare mind-body skills training with other interventions designed to improve resilience and compassion while decreasing burnout in health professionals.

## P87 Phase II clinical trial on efficacy and safety of additive mistletoe extract therapy in combination with standard radiotherapy and chemotherapy in patients with newly diagnosed glioblastoma after surgical resection (GLIOMIS-trial) – a trial protocol

### Gunver Kienle^1^, Jörg Dietrich^2^, Claudia Schmoor^3^ , Roman Huber^1^

#### ^*1*^*Center for Complementary Medicine, Institute for Environmental Health Sciences and Hospital Infection Control, Medical Center – University of Freiburg, Freiburg, 79106, Germany;*^*2*^*Massachusetts General Hospital, Boston, MA, United States;*^*3*^*Clinical Trials Unit Freiburg, Medical Center – University of Freiburg, Freiburg, Germany*

##### **Correspondence:** Gunver Kienle

**Background**

Glioblastoma multiforme (GBM) is the most common primary malignant intracranial neoplasm. Despite surgery, radiotherapy and Temozolomide intervention, GBM cannot be cured and the median survival is 16 months. Treatments are desperately needed that improve the results of standard treatments without further impairing the quality of life. Mistletoe extracts (ME) are widely used in integrative cancer care and show promising preclinical effects in GBM cells but have hardly been investigated in GBM patients.

**Objective**

Do ME improve progression-free survival, quality of life, cognitive functioning and influence local immune response in GBM patients?

**Method**

Prospective randomised, double-blind, placebo-controlled, parallel-group, multicentre trial; stratified for center, 1:1 randomization. 150 patients with newly diagnosed, supratentorial glioblastoma, after surgical resection, starting radiotherapy and Temozolomid will be included in 5 US American and German centers. They will be randomized to receive either ME (Iscador Qu®, 0.01–10 mg, sc, 3/week in an individually adapted, dose-escalating scheme), or isotonic saline solution (identical scheme, sc, 3/week), until tumor progression. Primary outcome is progression-free survival, assessed by magnetic resonance imaging as per standard of care (every three months in Germany, every two months in the US) or earlier when clinically indicated, allowing for a one month surveillance period, to account for possible pseudo-progressions. Key secondary endpoints are health-related quality of life (EORTC QLQ-C30, EORTC QLQ BN20), neurocognitive assessment (IPCG battery, MMSE), overall survival, neutropenia due to chemotherapy, safety, immune parameters gene expression profiles in the tumor microenvironment before and after treatment (subgroup of patients).

Funding

Public and private.

## P88 Use of complementary and alternative medicine among patients attending spine specialty hospitals in South Korea

### Weon H Kim^1^, Dongwoon Han^2^, Mansoor Ahmed^2^, Luzhu He^2^, Jung Hye Hwang^2^

#### ^*1*^*Graduate School of Public Policy, Hanyang University, Seoul, 133-791, South Korea;*^*2*^*Global Health and Development, Hanyang University, College of Medicine, Seoul, 04763, South Korea*

##### **Correspondence:** Weon H Kim

**Purpose**

The purpose of the study is to describe the use of CAM among patients with chronic diseases, who visiting spine speciality hospitals in Seoul. This study also explored the characteristics of CAM user, the prevalence of CAM use, perceived effectiveness of CAM, and variables associated with CAM use and patients perception on the symptoms of chronic disease.

**Method**

The data were collected with a questionnaire for this study from 26 November, 2015 to 5 December, 2015. A cross sectional survey design was use to carry out face-to-face interviews and self administered questionnaire. Data of subject on general demographic and perception of health status and disease and the experiences of health service use and CAM modalities used. Data were analyzed using SPSS (Statistical Package for the Social Science) 21.0 program to compare.

**Result**

The total numbers of participants were 322 with chronic disease. The result shows that 47.8% were using some form of CAM. The most commonly used therapies were acupuncture (response rate = 52.4%). The result of the logistic regression analysis of the factors related to the use of CAM showed that variables associated with CAM use were: subjective health status, perceived level of pain and effectiveness of traditional Korean medicine.

**Conclusion**

Prevalence of CAM use among outpatients with chronic diseases, attending spinal speciality hospitals was comparable with previous studies in developing and developed countries. The predictors of CAM use among the patients helps explain why the patients attending the specialty hospital turn to use CAM. Health professionals may need to identify patients” history of CAM use more carefully so as to better screen for possible adverse clinical interactions.

## P89 Tumor destructive and immune response effect of modulated electro-hyperthermia

### Eva Kiss^1^, Tamas Vancsik^1^, Nora Meggyeshazi^1^, Csaba Kovago^2^, Tibor Krenacs^1^

#### ^*1*^*1st Department of Pathology and Experimental Cancer Research, Semmelweis University, Budapest, 1085, Hungary;*^*2*^*Department of Pharmacology and Toxicology, Szent Istvan University Faculty of Veterinary Science, Budapest, 1078, Hungary*

##### **Correspondence:** Eva Kiss

Modulated electro-hyperthermia (mEHT, oncothermia) generates electric field which can interfere with malignant tumors at 42 °C resulting in destruction of neoplastic cells. The tumor selectivity of mEHT is due to increased metabolite content and permittivity of tumors compared to normal tissues. mEHT treatment can provoke apoptosis and immune cell infiltration in HT29 colorectal cancer xenografts of immunocompromised mice.

In this study C26 mouse colorectal adeno-carcinoma cell line was injected to both femoral regions of BalbC mice. The treatment groups were 1) mEHT treatment for 30 minutes on the right side tumor, 2) application of Marsdenia tenacissima (MTE, Chinese Xiao-Aiping decoctum), 3) combination of MTE and mEHT, 4) sham control. Tumor samples were tested for mEHT related tumor destruction, stress and immune response (Ki67, cleaved caspase-3, cytochrome c, AIF, TRAIL, Hsp70, HMGB1, CD3, S100).

Significant caspase-3 dependent tumor destruction were observed both in the mEHT group and in the combination group. Expression of TRAIL receptor and mitochondrial release of cytochrome c could also be seen in the treated tumors. The absence of AIF and the positive TUNEL assay also indicated the extrinsic way of apoptosis. Significant elevation of S100 positive dendritic cells and CD3 positive cells in the treated tumors of mEHT group and in the combination group both sides may refer to immunogenic cell death.

mEHT in combination with MTE can induce caspase-dependent programmed cell death. Additionally the elevated dendritic cells and the appearance of T cells indicate immunogenic tumor cell death response in C26 colorectal tumor allograft model.

## P90 Internal coherence scale – cross sectional study to investigate reliability and validity in a geriatric population

### Anne K Klaus^1,2^, Roland Zerm^1,2^, Danilo Pranga^1^, Thomas Ostermann^3,4^, Marcus Reif^5^, Hans Broder von Laue^1^, Benno Brinkhaus^6^, Matthias Kröz^1^

#### ^*1*^*Research Institute Havelhöhe, Berlin, 14089, Germany;*^*2*^*Internal Medicine, Hospital Havelhöhe, Berlin, Germany;*^*3*^*Integrative Medicine, Witten/Herdecke University, Witten/Herdecke, Germany;*^*4*^*Methodology and Statistics in Psychology and Psychotherapy, Witten/Herdecke University, Witten/Herdecke, Germany;*^*5*^*Society for Clinical Research, Berlin, Germany;*^*6*^*Institute for Social Medicine, Epidemiology and Health Economics, Charité University, Berlin, 10117, Germany*

##### **Correspondence:** Anne K Klaus (anne-kathrin.klaus@charite.de)

**Background**

The Sense of Coherence Scale (SOC) has been increasingly used in epidemiological studies with promising results. Nevertheless, because of its retrospective focus the SOC is not applicable as a clinical questionnaire. Therefore, the compact 10-item Internal Coherence Scale (ICS) has been validated in patients with chronic conditions, and in healthy people between 30 and 83 years. The aim of this study was to evaluate the reliability and validity of the ICS in elderly people.

**Methods**

The cross sectional study was conducted in a German geriatric population from 2013–2015. Elderly people older than 70 years were retested up 2 until 4 weeks later in at least 50% of all participants. To apply for convergent validity SOC, Short Form Health Survey (SF-12), and Geriatric Depression Scale (GDS) have been administered beside the ICS.

**Results**

104 people (age 70–96 years; cancer patients (n = 32), diabetes mellitus type 2 patients (n = 22), and age-matched relative healthy controls (n = 51) have been included. The 10-item ICS showed sufficient reliability (Cronbach”s alpha: r = 0.72, test-retest reliability: r = 0.52, p < 0.01). Sufficient construct and convergent validity were shown with moderate correlations to SOC, SF-12, and GDS (0.34–0.45, p < 0.01). In the factor analysis the subscale structure (Inner Coherence and Thermo Coherence) as published in the German validation within a younger sample was confirmed.

**Conclusions**

In this study ICS is featured by sufficient reliability, robust validity with health-markers and stable subscale structure in elderly people confirming the results of a prior study in a younger population.

## P91 Validation of the trait autonomic regulation questionnaire in a geriatric population

### Anne K Klaus^1,2^, Roland Zerm^1,2^, Danilo Pranga^1^, Daniela Rodrigues Recchia^3^, Thomas Ostermann^3,4^, Marcus Reif^5^, Hans B von Laue^1^, Benno Brinkhaus^6^, Matthias Kröz^1,2,3,6^

#### ^*1*^*Research Institute Havelhöhe, Berlin, 14089, Germany;*^*2*^*Internal Medicine, Hospital Havelhöhe, Berlin, Germany;*^*3*^*Integrative Medicine, Witten/Herdecke University, Witten/Herdecke, Germany;*^*4*^*Methodology and Statistics in Psychology and Psychotherapy, Witten/Herdecke University, Witten/Herdecke, Germany;*^*5*^*Society for Clinical Research, Berlin, Germany;*^*6*^*Institute for Social Medicine, Epidemiology and Health Economics, Charité University, Berlin, Germany*

##### **Correspondence:** Anne K Klaus (anne-kathrin.klaus@charite.de)

**Background**

Geriatric assessment is an important issue to capture symptom burden, functional limitations, and rehabilitative need of old people. Nevertheless, because of its multidimensional approach and often described limitations of geriatric patients it needs important time resources. Hence, it is of interest to develop pre-screening instruments. The questionnaire of autonomic regulation (aR) captures different items on autonomic functions with sufficient validity in German language for people between 18 and 85 years and correlations to health, and in case of loss of regulation with chronic conditions.

**Methods**

We report the reliability and validity results of the Trait version of aR-scale in a German geriatric population with a test-retest time-span of 2–4 weeks. Cumulative illness rating scale (CIRS), physical self-maintenance scale (PSMS), and geriatric depression scale (GDS) have been applied for convergent validity as geriatric assessment standard scales.

**Results**

104 peoplebetween 70 and 96 years (32 cancer, 22 diabetes mellitus type 2 patients, and 51 age-matched relative healthy controls) have been included. For the 18-item aR questionnaire sufficient until good reliability (Cronbach”s alpha r = 0.70, test-retest reliability r = 0.83) can be documented with robust construct, convergent validity with correlations to CIRS, PSMS and GDS: r = 0.27–0.31, p

**Conclusions**

Autonomic regulation is a reliable and valid questionnaire for a geriatric group capturing correlations with health, chronic conditions and geriatric assessment, but subscales differences demands clarification. AR could be a useful geriatric pre-screening instrument.

## P92 An international pragmatic, randomised controlled pilot study comparing individualised homeopathic add-on treatment and usual care only in women with premenstrual disorders (PMD)

### Christien T Klein-Laansma^1^, Mats Jong^2^, Cornelia von Hagens^3^, Jean P Jansen^1^, Herman van Wietmarschen^1^, Miek C Jong^1^

#### ^*1*^*Health Care and Nutrition, Louis Bolk Institute, Driebergen, 3972LA, Netherlands;*^*2*^*Nursing, Mid-Sweden University, Sundsvall, Sweden;*^*3*^*Naturopathy and Integrative Medicine, Department of Gynaecological Endocrinology and Reproductive Medicine, University Women’s Hospital Heidelberg, Heidelberg, Germany*

##### **Correspondence:** Christien T Klein-Laansma

**Study design**

A multi-centre, international, randomised, controlled pragmatic study with two parallel groups.

**Study period**

October 2012–July 2016.

**Objective**

To investigate the feasibility of organizing an international multi-centre pragmatic trial on an individualised homeopathic add-on treatment (HT) in women with premenstrual disorders (PMS/PMDD), compared to usual care only (UC).

**Methods**

After a two months’ screening phase, women diagnosed with PMS or PMDD were randomized to UC or HT for a 4 months’ treatment.

**Results**

In the Netherlands, recruitment took 2 years, 38 women were included. In Sweden, ethical approval was difficult to obtain, recruitment was slow and stopped after 3 years, 22 women were randomized. In Germany, even non-randomized case series with individualized homeopathy were classified as drug trial by the authorities.

Of 244 interested women, 114 started screening, 60 were randomized (HT: 28; UC: 32), 47 completed the study. 83% women preferred homeopathy; 75% had objections to antidepressants, 82% to Oral Contraceptive Pills (OCPs). Women in the UC group were advised to take OCPs, Intra Uterine Device (IUD), antidepressants or were referred. In the HT group, Sepia officinalis was most prescribed.

After four months, the relative mean change of premenstrual symptom scores in the HT group was significantly better than in the UC group (Ancova; p = 0.0028). No confounders were identified.

**Conclusions**

With respect to recruitment and different legal status, it seems not feasible to perform a larger randomized controlled clinical trial on individual homeopathic treatment for PMS in Europe. Final results will be presented at the conference.

Trial registration nb NTR3560

## P93 Predicting characteristics of Korean female subjects with hands and feet coldness: Korea-based multi-center pilot registry

### Youme Ko^1^, Seung-Ho Sun^2^, Ho-Yeon Go^3^, Chan-Yong Jeon^4^, Yun-Kyung Song^5^, Seong-Gyu Ko^1^

#### ^*1*^*Preventive medicine, Kyung Hee university, Seoul, 02447, South Korea;*^*2*^*Korean Internal Medicine, Sangji University, Wonju, South Korea;*^*3*^*Korean Internal Medicine, Semyung University, Jecheon, South Korea;*^*4*^*Korean Internal Medicine, Gachon University, Seongnam, South Korea;*^*5*^*Rehabilitation Medicine, Gachon University, Seongnam, South Korea*

##### **Correspondence:** Youme Ko

**Purpose**

The aim of this pilot study is to analyze the characteristics of Korean women with symptom of cold hands and feet (CHF) through the patient registry.

**Methods**

This study is prospective observational study which was conducted in 6 different Korean medicine hospitals in Korea. Each institution received approval from their own institutionalreview board. Before enrolling, researchers obtained and gave consent for research purposes. We recruited 134 female healthy participants who aged over 19 years and less than 59 years. The patient”s demographics, symptom characteristics of CHF and degree of symptoms were collected from all participants at baseline.

**Results**

134 female participants were enrolled within 6 months recruitment period and no dropouts during trial. 70 participants (52.2%) had complaint of cold extremities, and 64 (47.8%) did not suffer from coldness. The visual analogue scale score for CHF and skin temperature in acupoint PC8 between 2 groups were statistically significant. Identifying symptom patterns of pattern identification questionnaire by frequency analysis, skin and mouth dryness and preference for warmth in any condition were most common symptoms in CHF participants. The result of pattern diagnosis by Korean medicine experts showed that deficiency pattern were the most frequent cause of CHF, especially blood and qi deficiency patterns.

**Conclusion**

Through this trial, we explored the characteristics of CHF patients in Korea. This data will be used to specify the target population and develop an appropriate design of the experimental trial that will be conducted in near future.

## P94 The role of yoga and self-esteem for menopausal symptoms and quality of life in breast cancer survivors - a mediation analysis

### Anna K Koch^1,2^, Sybille Rabsilber^3,4^, Romy Lauche^1,5^, Sherko Kümmel^4^, Gustav Dobos^1^, Jost Langhorst^1,2^, Holger Cramer^1,5^

#### ^*1*^*Department of Internal and Integrative Medicine, Kliniken Essen-Mitte, Essen, 45276, Germany;*^*2*^*Centre of Integrative Gastroenterology, Kliniken Essen-Mitte, Essen, Germany;*^*3*^*Department of Gynecology, Malteser Hospital St. Anna, Duisburg, Germany;*^*4*^*Interdisclipinary Breast Cancer Center, Kliniken Essen-Mitte, Essen, Germany;*^*5*^*Australian Research Center in Complementary and Integrative Medicine, Faculty of Health, Sydney, Australia*

##### **Correspondence:** Anna K Koch

**Purpose**

When undergoing menopausal transition, breast cancer survivors cannot take hormonal medicine used for treatment of menopausal symptoms. Yoga as a hormone free alternative has been shown to enhance quality of life and ease menopausal symptoms of breast cancer survivors. The present study tested the mediating effects of self-esteem in the relationships between yoga, quality of life, fatigue and menopausal symptoms.

**Methods**

Analyses were based on a previously published open-label, randomized controlled clinical trial assessing the longitudinal effect of yoga in comparison to usual care in 40 breast cancer survivors who suffered from menopausal symptoms. Self-esteem was assessed by the Rosenberg Self-Esteem Scale at week 12 after randomization. Outcomes included menopausal symptoms (Menopause Rating Scale), quality of life (Functional Assessment of Cancer Therapy-Breast), and fatigue (Functional Assessment of Chronic Illness Therapy-Fatigue) at week 24. Mediation analyses were performed using SPSS and applying bootstrapping.

**Results**

Self-esteem mediated the effect between yoga and quality of life (*B* = 8.04, 95% BCI [3.15 to 17.03]), social well-being (*B* = 1.80, 95% BCI [.54 to 4.21]), emotional well-being (*B* = 1.62, 95% BCI [.70 to 3.34]), functional well-being (*B* = 1.84, 95% BCI [.59 to 4.13]), fatigue (*B* = 4.34, 95% BCI [1.28 to 9.55]), total menopausal symptoms (*B* = -2.11, 95% BCI [-5.40 to -.37]), psychological menopausal symptoms (*B* = -.94, 95% BCI [−2.30 to−.01]), and urogenital menopausal symptoms (*B* = -.66, 95% BCI [−1.65 to −.15]). The effects on physical well-being (*B* = .79, 95% BCI n.s.), and somatovegetative menopausal symptoms (*B* = -.50, 95% BCI n.s.) were not mediated by self-esteem.

**Conclusions**

Findings support the assumption that self-esteem plays a vital role in the process of the beneficial effect of yoga. Yoga can have long-term benefits for women who suffered from former breast cancer undergoing menopausal transition.

Trial registration: clinicaltrials.gov (registration number NCT01908270)

## P95 Herbal medicine in the treatment of irritable bowel syndrome – a systematic review

### Anna K Koch^1,2^, Milena Trifunovic-Koenig^1,2^, Petra Klose^1^, Holger Cramer^1,3^, Gustav Dobos^1^, Jost Langhorst^1,2^

#### ^*1*^*Department of Internal and Integrative Medicine, Kliniken Essen-Mitte, Essen, 45276, Germany;*^*2*^*Centre of Integrative Gastroenterology, Kliniken Essen-Mitte, Essen, Germany;*^*3*^*Australian Research Center in Complementary and Integrative Medicine, Faculty of Health, Sydney, Australia*

##### **Correspondence:** Anna K Koch

**Background**

Herbal medicine is a promising alternative in the treatment of irritable bowel syndrome (IBS). We performed a systematic review for herbal treatments of IBS.

**Methods**

A computerized search of databases Cochrane Library, PubMed, Psychinfo, and Scopus through July 20th 2016 was performed. Randomized controlled trials (RCT) and controlled trials (CT) evaluating adults diagnosed with IBS were included. No language restriction was applied. Trials on traditional Chinese medicine were not included.

**Results**

A total of 26 trials with 20 different herbal treatments and a total of 1915 patients with IBS met the inclusion criteria. Herbal medications were compared with placebo or conventional pharmacologic therapy or tested combined with conventional therapy. Compared with placebo, red pepper, peppermint oil, ispaghula, caraway oil, STW 5, STW 5-II, and Dinggui Oil showed beneficial effects. Compared with placebo, also berberine hydrochloride was beneficial in releasing symptoms. However, even though berberine is an herbal medicine, an extract was used in the study. Ayurvedic therapy consisting of aegle marmelos correa plus bacopa monniere linn was particularly beneficial in diarrhoea predominant form. Compared with conventional therapy, an herbal preparation (mentha longifolia, cyperus rotundus and zingiber officinale) and supermint showed beneficial effects. Combined with conventional therapy, Gwakhyangjeonggisan (GJS), an herbal preparation (melissa officinalis, mentha spicata, and coriandrum sativum) showed additional benefit compared with conventional therapy alone. No evidence for the efficacy of ayurvedic herbs consisting of murraya koenigii, punica granatum and curcuma longa, bitter candytuft, St John’s wort, ginger, curcuma, furmitory, and aloe vera was found. A differentiation between IBS-subtypes was not possible due to inconsistent reporting within the trials. No serious adverse events regarding the herbal treatments were reported.

**Conclusions**

Various herbal preparations show promising effects in the treatment of IBS. Especially peppermint oil is well evaluated and effective. Further studies regarding the other herbal medicines and a more stringent attention regarding the different IBS subtypes are necessary.

## P96 Measuring patient-perceived quality of care in integrative medicine

### Evi Koster^1^, Erik Baars^1^, Diana Delnoij^2^

#### ^*1*^*University of Applied Sciences Leiden, Leiden, 2333CK, Netherlands;*^*2*^*Scientific Centre for Transformation in Care and Welfare (Tranzo), Tilburg University, Leiden, Netherlands*

##### **Correspondence:** Evi Koster

**Background**

This study aimed at measuring patient-perceived quality of care in Integrative Medicine (IM). Patient-perceived quality of care is becoming increasingly important in evaluating healthcare. Specific methodologies have been developed, such as Consumer Quality-Index (CQ-Index) and other PREMs (Patient Reported Experiences Measures) for measuring process aspects, and PROMs (Patient Reported Outcome Measures) for measuring outcomes. Because (IM) has a holistic and individual-oriented approach, and applies specific patient-relevant aspects, it is unknown whether current “conventional” methodologies are able to measure patient-perceived quality of IM adequately.

**Method**

IM is addressed by focusing on Anthroposophic Medicine (AM).

To measure patient-perceived quality of AM, methods used are: existing conventional methodologies, extended conventional methodologies with patient-relevant AM aspects, and newly developed methodologies to measure patient-relevant AM aspects.

To identify patient-relevant aspects, methods used are: focus groups, semi-structured interviews, surveys, qualitative triangulation and literature research. Comparative statistical analyses are performed to compare patient experiences in AM and conventional care.

**Results**

Patient-relevant aspects regarding quality of care of AM are identified.

Patient-relevant domains on quality of life (QOL) are constructed and prioritised. Contributions of AM to self-management from patients” perspectives are explored. In the AM children”s healthcare centre in Zeist parent-perceived additional values are evaluated. The standard CQ-Index General Practice is extended with AM-specific items.

**Conclusions**

Relevant quality aspects of AM partly overlap with and partly differ from aspects in conventional care. Patients particularly value aspects regarding individual tailored treatment and possibilities, natural healing and patient-provider relationship. Patient-perceived quality of AM is good.

## P97 Health competence as key to longterm disease prevention

### Lena Kroll, Kathrin Weiss

#### *Sport sciences, University of Augsburg, Augsburg, 86135, Germany*

##### **Correspondence:** Lena Kroll (lena.kroll@sport.uni-augsburg.de)

**Background**

Health literacy has become an important term for long-term disease prevention over the last 20 years. Still, almost 50% of Europeans show low rates in health literacy and new approaches for its promotion have to be developed.

At Augsburg University, a longitudinal survey examines from 2015–2017 the influences of an intervention on health literacy of University staff. The intervention focusing on Yoga consists of three standardized modules, “1 - the health-related basis” (Yoga classes), “2 - the transfer into working life” (individualized support at working place) and “3 - the integration in working and everyday life” (self-dependent) and is oriented at the theoretical concept of “health competence”.

**Methods**

The survey consists of a longitudinal quasi-experimental control trial (t0: Nov.2015; t1: Feb.2016, t2: July 2016, t3: Nov.2016) using validated scales on health (WHOQOL-Bref, WHO, 2000), on work-related behavior and experience patterns (AVEM, Schaarschmidt & Fischer, 2008), on health literacy / competence (Lenartz, 2012), and on the actual physical well-being (WKV-20, Kleinert, 2006). The sample at t2 included 92 individuals in the intervention group and 129 participants in the control group.

**Results**

There have been significant improvements within the intervention-group in almost all aspects measured in the survey. Out of 16 dimensions, 13 changed significantly or highly significantly in a positive way. The results for the control-group did not change significantly in any dimension.

**Conclusion**

Especially the long-term trend shows interesting results and allows an optimistic view on the promotion of health competence / literacy in University staff with Yoga.

## P98 Mobile app-based mindfulness intervention for cancer patients and their caregivers - a feasibility study within an integrated health care delivery system

### Ai Kubo^1^, Sarah Hendlish^1^, Andrea Altschuler^1^, Nancy Connolly^1^, Andy Avins^1,2^

#### ^*1*^*Division of Research, Kaiser Permanente, Oakland, CA 94612, United States;*^*2*^*University of California, Department of Medicine, San Francisco, CA, United States*

##### **Correspondence:** Ai Kubo

**Question**

Is a mobile-based mindfulness intervention feasible and accepted among cancer patients undergoing chemotherapy and their primary caregivers?

**Methods**

Eight-week single-arm pilot trial within Kaiser Permanente, Northern California oncology clinics. Participants were cancer patients with ≥8 weeks of remaining chemotherapy and their primary unpaid caregivers, where neither had a regular meditation practice. Participants were given access to a commercially available mindfulness program, HeadspaceTM, via smartphone application or home computer, and were asked to listen to meditation instruction for 10–20 minutes daily, for 8 weeks. Data on depression, anxiety, sleep, fatigue, quality of life, caregiver burden, and satisfaction with care were collected at baseline, at 4 weeks and following the intervention. Paired t-tests were used to assess before-after changes.

**Results**

28 patients (median age 65.5y; female 71%) and 15 caregivers (median age 61y; female 60%) were enrolled. Among them, 19 patients (68%) and 9 caregivers (60%) completed the study. Of these, 71% practiced meditation >50% of the days; 39% practiced >70% of the days. Participants experienced significant reduction in levels of depression (p = 0.009) and anxiety (p = 0.003), improvement in physical (p = 0.005) and mental domains of quality of life (p = 0.0001), sleep quality (p = 0.03), and fatigue (p = 0.02). In qualitative interviews, participants reported feeling more relaxed, positive, and resilient, sleeping better, and having less pain.

**Conclusions**

This pilot trial of a mobile-app/online mindfulness program shows promise in reducing anxiety and depression and improving quality of life among cancer patients and caregivers unable to attend traditional in-person classes. Larger, randomized studies could fully assess efficacy.

## P99 Using a cluster-analytic approach to identify profiles and predictors of healthcare utilization typical users across conventional, allied and complementary medicine and self-care

### Romy Lauche^1^, Daniela Rodrigues Recchia^2^, Holger Cramer^1^, Jon Wardle^1^, David Lee^3^, David Sibbritt^1^, Jon Adams^1^, Thomas Ostermann^2^

#### ^*1*^*Australian Research Centre in Complementary and Integrative Medicine (ARCCIM), University of Technology Sydney, Ultimo, 2007, Australia;*^*2*^*Department of Psychology and Psychotherapy, University of Witten/Herdecke, Witten, Germany;*^*3*^*Department of Public Health Sciences, University of Miami, Miami, FL, United States*

##### **Correspondence:** Holger Cramer

**Introduction**

Cluster analytic techniques can identify health care utilization patterns, and define typologies of consumers and their specific characteristics based on the similarity of their behaviour. This study aims to examine health care utilization patterns using a cluster analytic approach; and the associations of health care user types with sociodemographic, health-related and health-system related factors.

**Methods**

Cross-sectional data from the 2012 National Health Interview Survey were used (n = 32,017). Twelve-month self-reported health care utilization behaviours were assessed across a variety of medical, allied and complementary healthcare modalities including self-care interventions (exercise, diet, supplementation etc.). A model-based clustering based on finite normal mixture modelling, and several indices of cluster fit were determined. Health care utilization within the cluster was described descriptively, and independent predictors of belonging in the respective clusters were analysed using logistic regression models including sociodemographic, health- and health insurance-related factors.

**Results**

A 9-cluster solution describing 9 different health care user types, from nearly non-use of health care modalities, to over-utilization of medical, allied and complementary health care including self-care was found. Several sociodemographic and health-related characteristics were associated with cluster membership, including age and gender, health status, education, income, ethnic origin, and health care coverage.

**Conclusions**

Cluster analysis can be used to identify typical health care utilization patterns based on empirical data, and those typologies are related to a variety of sociodemographic and health-related characteristics. Those findings may provide information for future health research and policy.

## P100 Is the use of yoga and meditation associated with a healthy lifestyle? Results of a national cross-sectional survey of 28,695 Australian women

### Romy Lauche^1^, David Sibbritt^1^, Crystal Park^2^, Gita Mishra^3^, Jon Adams^1^, Holger Cramer^1,4^

#### ^*1*^*Australian Research Centre in Complementary and Integrative Medicine (ARCCIM), University of Technology Sydney, Ultimo, 2007, Australia;*^*2*^*Department of Psychology, University of Connecticut, Storrs CT, United States;*^*3*^*School of Public Health, University of Queensland, Herston QLD, Australia;*^*4*^*Department of Internal and Integrative Medicine, University of Duisburg-Essen, Essen, Germany*

##### **Correspondence:** Holger Cramer

**Background**

Rooted in Indian philosophical, spiritual, and health practice yoga has become a popular avenue to promote physical and mental well-being. Traditionally yoga not only consists of physical exercises, but incorporates advice for an ethical and healthy lifestyle. This study aimed to examine the relationship between yoga/meditation practice and health behavior in three age cohorts of Australian women.

**Methods**

Women aged 19–25 years, 31–36 years, and 62–67 years from the Australian Longitudinal Study on Womens Health (ALSWH) were surveyed regarding smoking, alcohol or drug use, physical activity and dietary behavior; and whether they practiced yoga/meditation on a regular basis. Associations of those health behaviors with yoga/meditation practice were analyzed using multiple logistic regression modelling.

**Results**

11344, 8200, and 9151 women aged 19–25 years, 31–36 years, and 62–67 years, respectively, were included in the analysis of which 29.0%, 21.7%, and 20.7%, respectively, practiced yoga/meditation. Women practicing yoga/meditation were less likely to smoke regularly (OR = 0.41–0.47), and more likely to be physically active (OR = 1.50–2.79) and to follow a vegetarian (OR = 1.72–3.22) or vegan (OR = 2.26–3.68) diet. Women practicing yoga/meditation were also more likely to use marijuana (OR = 1.28–1.89) and illicit drugs (OR = 1.23–1.98).

**Conclusions**

Yoga/meditation practice was associated with a higher likelihood of non-smoking, regular physical activity, and vegetarian/vegan diet. While health professionals need to keep the potential vulnerability of yoga/meditation practitioners to drug use in mind, the positive associations of yoga/meditation with a variety of positive health behaviors warrant its consideration in preventive medicine and healthcare.

## P101 Oncogenetic key signal RANTES/CCL5 - Cytokine cross talk in tumors and silent inflammation of jawbone

### Johann Lechner

#### *Praxisklinik München, Munich, 81547, Germany*

**Background**

Despite significant therapeutic advances most malignancies, as well as adenocarcinomas of the breast, remained incurable. At the same time, the importance of the microenvironment surrounding the tumor cells with “silent inflammation” increases.

**Objective**

To check the suspected tumor-relevant inflammatory cytokine sources in fatty-degenerative osteonecrotic jawbone (FDOJ), we analyze these conspicuously altered jawbone areas to assess the expression and quantification of cytokine expression.

**Material and Method**

In 38 tumor patients we determine the levels of cytokines by bead-based Luminex® analysis in samples of FDOJ.

**Results**

Striking is the high content of chemokine RANTES/CCL5 (R/C) in all 38 tissue samples. A single case is characterized by high R/C levels in FDOJ sample and simultaneously by metastasizing cells inside the FDOJ sample. The R/C expression in all 38 FDOJ samples is on average at 35 fold higher compared to healthy jawbone.

**Discussion**

R/C interacts on several levels in immune responses and is considered in scientific literature as pathogenetic key point in tumor growth. The study supports a potential mechanism where FDOJ is a mediating link specifically in breast cancer (MaCa) and its metastasis. R/C is thus involved intensively in oncogenic propulsion progress developments.

**Conclusion**

The authors conclude from the data of FDOJ analysis that these areas express hyperactivated signal transduction of the chemokine R/C, induce pathogenetic autoimmune processes in tumors, MaCa and its metastasis and serve as a possible cause. Combining the R/C signal induction of tumors and the information we collect illustrated, it may be suggested to involve FDOJ in an integrative therapy concept for tumor therapy.

## P102 Acupuncture for autism spectrum disorder: a systematic review and meta-analysis

### Boram Lee^1^, Jihong Lee^1^, Jinhong Cheon^2^, Hyun K Sung^3^, Seunghun Cho^4^, Gyu T Chang^1^

#### ^*1*^*Departments of Pediatrics, Kyung Hee University, College of Korean Medicine, Gangdong-gu Seoul, South Korea;*^*2*^*Departments of Pediatrics, Pusan National University, College of Korean Medicine, Pusan, South Korea;*^*3*^*Departments of Pediatrics, Semyung University, College of Korean Medicine, Chungju, South Korea;*^*4*^*Departments of Neuropsychiatry, Kyung Hee University, College of Korean Medicine, Seoul, South Korea*

##### **Correspondence:** Boram Lee

**Background**

Autism Spectrum Disorder (ASD) is characterized by persistent deficits in social communication and interaction, and restricted, repetitive patterns of behavior, interests or activities. Parents of children with ASD have been concerned about potential adverse effects of drug and are seeking for treatments which are more secure. Therefore, acupuncture, one of the forms of Complementary and Alternative Medicine, with fewer adverse effects has been increasingly looked out. There were systematic reviews about acupuncture for ASD, but they concluded that acupuncture had limited or no evidence until 2012. Since then, many researches about acupuncture for ASD have been published. So we aimed to summarize and evaluate the up-dated evidence of efficacy and safety on acupuncture for ASD.

**Methods**

We searched 13 electronic databases up to December 2016. Randomized Controlled Trials (RCTs) assessing the efficacy of acupuncture for ASD were included. The details on acupuncture procedure of the included studies were reported based on the revised Standards for Reporting Interventions in Clinical Trials of Acupuncture (STRICTA) guidelines. The risk of bias was assessed using the Cochrane risk of bias assessment tool. Data analysis was performed using RevMan software version 5.3.

**Results**

33 RCTs involving 2002 patients with ASD were included**.** Data from 23 RCTs was used for meta-analysis. When added to conventional treatment, acupuncture group had significantly low Childhood Autism Rating Scale (CARS) (MD = −8.48, 95% CI −12.29 to −4.68) and Aberrant Behavior Checklist (ABC) (MD = −7.98, 95% CI −10.25 to −5.72) scores after intervention compared with the conventional treatment group. Also acupuncture group lowered Autism Treatment Evaluation Checklist (ATEC) (MD = −10.71, 95% CI −15.57 to −5.84), improved Functional Independence Measure for Children (WeeFIM) (MD = 3.23, 95% CI 1.46 to 5.01) and had a high total effective rate (OR = 5.28, 95% CI 3.53 to 7.91) compared with the control group. Acupuncture lowered CARS more than conventional treatment and improved WeeFIM more than sham acupuncture. The group of conventional treatment during retention of acupuncture needles had low CARS score and high total effective rate compared with the group doing acupuncture and conventional treatment separately. Within the studies, there were no serious adverse events associated with acupuncture.

**Conclusions**

Evidence of efficacy on acupuncture for ASD is encouraging, but not conclusive in this review, because of the low methodological qualities and heterogeneities of the included studies. Further well-designed RCTs are needed to confirm these results. This trial is registered in PROSPERO CRD42017054544.

This study was supported by the Traditional Korean Medicine R&D program funded by the Ministry of Health & Welfare through the Korea Health Industry Development Institute (KHIDI) (Number: HB16C0075).

## P103 Neural network underlying the recovery from disowned bodily states induced by rubber hand illusion

### Inseon Lee^1^, Younbyoung Chae^2^

#### ^*1*^*Abt. Innere Medizin VI - Psychosomatische Medizin und Psychotherapie, Universitätsklinikum Tübingen, Tübingen, 72076, Germany;*^*2*^*Acupuncture & Meridian Science Research Center, Seoul, 130-701, South Korea*

##### **Correspondence:** Younbyoung Chae

**Background**

It has not been investigated that how brain could recover from the illusory self-attribution by the rubber hand illusion (RHI). Mechanical stimulation using acupuncture needle, on their own body would be a useful tool to re-instantiate their body ownership from the disowned bodily states. We used fMRI and investigated how the causal influence between brain regions during the RHI are modulated by the two different types of mechanical stimuli (tactile and visual stimuli).

**Methods**

We applied the needle rotations during RHI in two different ways: one is at the real hand (re-instantiation by tactile stimuli: R-TS) and the other is at the rubber hand (re-instantiation by visual stimuli: R-VS). Here we utilized dynamic causal modeling to investigate interaction among four relevant brain regions: the ventral premotor cortex (PMv), the intraparietal sulcus (IPS), the secondary somatosensory cortex (SII), and the lateral occipitotemporal cortex (LOC).

**Results**

Tactile aspect of needle rotations changed the effective connectivity by directly influencing on the activity in the SII, whereas visual aspect of needle rotations changed the effective connectivity by influencing on both the SII and the LOC. The intrinsic connectivity parameter of the IPS to the PMv were significantly reduced in the R-TS condition compared to the R-VS condition. On the other hand, the modulatory parameter of the IPS to the PMv were significantly enhanced in the R-TS condition compared to the R-VS condition.

**Conclusions**

We demonstrated that the connectivity patterns driven by the disowned bodily states could be differently modulated by tactile or visual afferent inputs. Our findings indicate that effective connectivity between parietal and frontal multimodal areas would play a crucial role in re-instantiation of the body ownership.

## P104 What are the optimum electroacupuncture parameters for relaxation or pain control?

### Jisu Lee^1^, Seung H Cho^1^, Yujin Choi^2^

#### ^*1*^*Kyung Hee University, College of Korean Medicine, Seoul, South Korea;*^*2*^*Kyung Hee University, Department of Clinical Korean Medicine, Graduate School, Seoul, South Korea*

##### **Correspondence:** Seung H Cho

**Background**

Earlier studies have shown electroacupuncture (EA) stimulations of both low frequency and high frequency induced analgesia through different mechanisms, either by decrease or increase of cortical excitation. This study aims to examine EEG status during different EA modes of frequency and intensity.

**Method**

We first measured baseline EEG of the participants, and then measured EEG during EA stimulation in two different modes; low frequency (2Hz)-high intensity or high frequency (120Hz)-low intensity in random order. Participants were required to close their eyes and each following sessions took 5 minutes. EA was stimulated on the same acupuncture point ST36 bilaterally. After filtering the artifact from raw EEG data, each set of EEG data was subjected to Fast-Fourier Transform (FFT) analysis to obtain the absolute and relative EEG band power at each electrode. Repeated-measure analysis of variance (ANOVA) was conducted for statistical analysis.

**Result**

15 volunteers participated for the study (aged 20–29 years). Compared to baseline EEG, absolute theta power significantly decreased during EA stimulation (p < 0.05). Moreover, relative alpha power in parietal area (P3, P4) increased during the low frequency-high intensity EA stimulation (p = 0.01). Relative beta power in frontal area (Fp1, F3) increased during the high frequency-low intensity EA stimulation (p < 0.05).

**Conclusion**

The frequency and the intensity variables resulted in the change on the EEG pattern. The heightened alpha power in parietal area during low frequency-high intensity EA stimulation potentially refers to relaxed wakefulness. Whereas increased beta power in frontal area during high frequency-low intensity EA stimulation suggests that it is probably more suitable for pain control.

## P105 Analysis of clinical characteristics and treatments in geriatric cancer patients hospitalized in Korean Medicine Hospital

### Jee Y Lee, Han S Ryu, Sung S Yoon, Hye K Oh, Lyun K Hyun, Jin O Kim, Seong W Yoon

#### *Kyung Hee University Hospital at Gangdong, Korean internal medicine, Seoul, South Korea*

##### **Correspondence:** Jee Y Lee

**Background**

As the overall health condition is improving, geriatric cancer patients are increasing. Still, there is limited information about treatments for this population. This study was aimed to analyze clinical characteristics and the factors influencing treatment decision in elderly cancer patients.

**Methods**

Data of the elderly cancer patients (≥65 years) who were admitted to Korean Medicine Hospital from March 2014 to February 2016 were collected. We compared the clinical characteristics and overall survival of the chemotherapy group and non-chemotherapy group.

**Results**

19 patients were included in this study. 9 people received chemotherapy concurrently and 10 people did not receive chemotherapy due to concerning about quality of life. Age, Eastern Cooperative Oncology Group (ECOG) performance status, Activities of Daily Living (ADL) score showed differences between two groups. And median survival time was not significantly different between two groups. Compared with chemotherapy alone, Traditional Korean Medicine combined with chemotherapy prolonged median survival time.

**Conclusions**

Old age, low performance status and ADL score are influential factors for receiving chemotherapy. Further studies are needed to confirm these factors influencing decision making of cancer treatments in geriatric cancer patients.

## P106 Pressure waveform study of radial artery with heart condition for traditional medical application: using a cardiovascular simulator

### Ju-Yeon Lee^1^, Sang-Hoon Shin^2^, Min Jang^2^, Indra Müller^1^, So-Hyun Janson Park^1^

#### ^*1*^*Cardiovascular Engineering, Helmholtz Institute of RWTH Aachen University & Hospital, Aachen, 52074, Germany;*^*2*^*Oriental biomedical engineering, Sangji University, Wonju, 26339, South Korea*

##### **Correspondence:** So-Hyun Janson Park

**Background**

Cardiovascular disease patients cannot ascertain a decline in their health themselves. Pulse diagnosis, a traditional diagnosis method, permits cardiovascular system monitoring by modern equipment through non-invasive and real-time measurement. The purpose of this paper is to study the effect of change in heart condition on radial artery pulse waveforms using a cardiovascular simulator.

**Methods**

The simulator comprised of two parts: The cardiac component was developed at RWTH Aachen University and the blood vessel component, at Sangji University. A pressure-volume loop measured with changes in heart rate and stroke volume. The radial arterial pressure was measured with an invasive sensor and a tonometry sensor. Heart rate represents the rhythm of heart and stroke volume represents the contractility of heart.

**Results**

The mean blood pressure increased with an increase in stroke volume and heart rate. Pulse pressure increased with an increase in stroke volume— a typical human body phenomenon, but decreased with an increase in heart rate. Additionally, oscillation of the blood pressure waveform increased with an increase in heart rate.

**Conclusions**

Experimental results indicate that the characteristics of the simulator correspond well with those of the heart, measured at the radial artery. And we could check out the effects of stroke volume and heart rate on radial artery separately. This study proves that the simulator developed will find widespread application in various future studies involving scientific pulse diagnosis. Also, we expect that pulse diagnosis can be used as a tool to self-assess heart condition based on these results.

## P107 A qualitative evaluation of the Integrative Medical Group Visits (IMGV) model of care for chronic pain and depression; lessons learned from clinician-patient interactions

### Anna S Lestoquoy^1^, Lance Laird^2^, Lily Negash^1^, Suzanne Mitchell^2^, Paula Gardiner^1^

#### ^*1*^*Family Medicine, Boston University Medical Center, Boston, 02118, United States;*^*2*^*Boston University School of Medicine, Boston, United States*

##### **Correspondence:** Anna S Lestoquoy

**Purpose**

Integrative Medical Group Visits (IMGV) are an innovative program for delivering chronic pain care. These visits combine principles of Mindfulness Based Stress Reduction (MBSR) and Evidence Based Integrative Medicine techniques with a group medical visit. In this qualitative analysis, the IMGV program is contrasted with usual care (visits to a primary care clinician).

**Methods**

Independent interviewers conducted 4 focus groups (N = 20) with patients. Four independent coders used thematic analysis to analyze the transcripts of these sessions.

**Results**

Participant demographics were as follows: Black (65%), Latino (35%); and the average age was 51. The aggregate pain level was 6 (scale 1–10, SD 2.29) and PHQ-9 depression score was 11 (scale 1–27, SD 5.6). Themes for usual care include: doctors using medication as a “default” solution for pain treatment, not being informed of alternative treatment options, not being told how side effects may affect functioning, and poor coordination of care among different providers. Many reported lack of options for chronic pain care as their motivation for being part of the IMGV. Reasons participants preferred the IMGV model included: it was customizable, the techniques could be used at any point in time, the model took into account emotional and social health and it allowed patients more access to providers.

**Conclusions**

Patients expressed a preference for the IMGV model over usual care for chronic pain. It is possible the IMGV model is able to better address the social challenges and health disparities this underserved population faces than individual provider appointments.

## P108 Yinlai decoction influences on mucosal immunity of mice with dyspesia combined with FM1 influenza virus infection

### Xiaofei Li^1,2^, Yunhui Wang^1^, Jianhua Zhen^1,3^, He Yu^1^, Tiegang Liu^1^, Xiaohong Gu^1^

#### ^*1*^*Beijing University of Chinese Medicine, Beijing, 100029, China;*^*2*^*Pediatrics, First People’s Hospital of Pingyuan County, Shangdong, China;*^*3*^*Department of Pulmonary Diseases, China-Japan Friendship Hospital, Beijing, China*

##### **Correspondence:** He Yu (yuhe221@126.com)

**Objective**

To explore the influence of Yinlai decoction on mucosal immunity in mice with dyspepsia combined with FM1 influenza virus infection, and identify the efficacy of Yinlai Decoction and its potential mechanism.

**Methods**

90 male mice were randomly allocated into normal group, infection group, infection and dyspepsia group, high, middle and low dose of Yinlai decoction groups, ShuanghuangLian group, XiaoerhuashiWan group, ribavirin group. A diet-induced mice model with high fat and high protein diet were built in infection and dyspepsia group and all the treating groups to build models of dyspepsia animal, and the mice were infected by FM1 influenza virus via nose in those groups. The level of secreted immunoglobulin A (sIgA), interleukin-10(IL-10), tumor necrosis factor-α(TNF-α) and interferon-γ(INF-γ) of lung and colon tissue homogenate by ELISA detection.

**Results**

Compared with the normal group, the levels of sIgA, IL-10 and TNF-α in colon tissue decreased, the levels of sIgA, INF-γ in lung tissue decreased significantly (P < 0.05), the levels of IL-10, TNF-α in lung tissue were increased in infection groups as well as infection and dyspepsia compound group. Compared with infection and dyspepsia compound group, the levels of sIgA, IL-10 in colon and lung tissue were increased significantly (P < 0.05) in middle dose of Yinlai decoction group, the levels of TNF-α in colon tissue increased significantly in each dose of Yinlai decoction group. Compared with the normal group, the levels of sIgA, INF-γ in lung tissue were decreased significantly in each dose of Yinlai decoction group (P < 0.05). Compared with the treating groups, the levels of IL-10 elevated significantly (P <0.05), the levels of TNF-α decreased significantly (P < 0.05) in lung and colon tissue, the lung tissue levels of INF-γ decreased significantly (P < 0.05) in each dose of Yinlai decoction group.

**Conclusion**

Yinlai decoction can promote the secretion of sIgA in lung and colon tissue, regulate the secretion of IL-10, TNF-α, INF-γ, thus regulate mice mucosal immune function and then achieve an anti-viral effect.

## P109 The regulatory effect of Qinlingye Extract on TLR4/NF-ĸB/iNOS pathway in kidney of uric acid renal injury rats

### Hui Liu, Weiguo Ma, Chengcheng Zhang, Xuezheng Shang, Yu Bai, Fengxian Meng

#### *Beijing University of Chinese Medicine Dong Fang Hospital, Chinese medicine, Beijing, China*

##### **Correspondence:** Hui Liu

**Objective**

To investigate the effects of Qinlingye extract (QLYE) on toll-like receptor (TLR)-4/NF-ĸB/iNos signaling pathway in kidney of uric acid renal injury rats.

**Methods**

Male SD rats aged 6 weeks with weight of 200 ± 10 g were randomly divided into control group, model group, positive group, and high-, medium-, low- dose groups of QLYE,12 in each group except 6 in normal group. The molding method was gavaging adenine and feeding yeast. Control group and model group were daily gavaged with distilled water (10 ml/kg · d), positive control group was daily gavaged with allopurinol by 23.33 mg/kg · d. High-, medium-, low-dose group of QLYE were daily gavaged with QLYE by 7.46 g/(kg · d), 3.73 g/(kg · d), 1.87 g/(kg · d), ig 8 weeks. Some rats of each group were sacrificed at the 6th and 8th week, and their kidneys were taken. mRNA transcription levels of TLR4 and iNOS in kidney were detected by RT-PCR. Protein levels of TLR4, NF-ĸBp65 and iNOS in kidney were examined by Western-blot and immunohistochemical staining.

**Results**

Compared with the normal group , mRNA and protein expression of TLR4, NF-ĸBp65 and iNOS in kidney increased significantly in the model group. Compared with the model group, mRNA expression of TLR4 was down-regulated at 6th week and that of iNOS was down-regulated at 8th week in 3 QLYE groups significantly (p < 0.01). Protein levels of TLR4, NF-ĸBp65 and iNOS ,examined by Western-blot, were significantly down-regulated in 3 QLYE groups both at 6th and 8th week (p < 0.01, p < 0.05). The results of immunohistochemical staining showed, protein levels of NF-ĸBp65 was significantly down-regulated in 3 QLYE groups at 6th week (p < 0.01) and in high-,medium-dose groups at 8th week (p < 0.05), TLR4 was down-regulated at 8th week , and iNos was down-regulated at 6th week significantly (p < 0.01, p < 0.05) in 3 QLYE groups.

**Conclusion**

The main molecular mechanisms of QLYE inhibiting immune inflammatory injury in high uric acid kidney tissue may be associated with the regulatory effect on TLR4/NF-ĸB/iNOS pathway.

## P110 The lower extremity contributions of the vertical support during Tai Chi

### Wei Liu^1^, Collin Rooney^2^, Amos Smith^3^

#### ^*1*^*Edward Via College of Osteopathic Medicine, Auburn, AL, United States;*^*2*^*Northwestern University, Evanston, IL, United States;*^*3*^*Auburn University, Auburn, AL, United States*

##### **Correspondence:** Wei Liu

**Background**

Tai Chi (TC) exercise is becoming an increasingly popular complementary and alternative approach for both healthy people and patients with a variety of medical conditions in the United States. TC is a continuous, slow rhythmic and bipedal movement, and this requires TC to control joints at ankle, knee and hip in a coordinate manner. The ground reaction force (GRF) is an important indicator of lower extremity synergy of vertical support during walking and running. Few studies have investigated the lower extremity contribution of GRF during TC. To better understand the biomechanics of TC, the purpose of study was to determine the lower extremity contribution of GRF during TC and compared to normal gait.

**Methods**

Ten healthy TC practitioners performed normal walking and TC while data were collected using high-speed motion capture system integrated with force plates. The lower extremity joint contribution of GRF were calculated by using induced acceleration analysis (IAA). A paired *t*-test (p < 0.05) was used to compare TC with normal walking.

**Results**

TC was significantly predominated by the contribution of the contralateral ankle (ankle: 32.44%) than normal waking (0.11%), whereas the knee was the primary contributor to support in normal walking (knee: 41.39%) than in TC (17.11%).

**Conclusion**

This study demonstrates that TC places a high mechanical demand on the contralateral ankle joint during vertical support. The lower stress the knee experiences during TC supports benefits of TC on decreasing knee joint load, which suggests TC as a potential therapy for people with joint disease.

## P111 Mindfulness for the management of pain in musculoskeletal disorders: a feasibility and preliminary efficacy study with nursing workers in a public university hospital in Brazil

### Shirlene Lopes^1^, Marcelo Demarzo^2^, Maria do Patrocínio Nunes^1^

#### ^*1*^*Internal Medicine-General Internal Medicine and Semiology, University of São Paulo-School of Medicine, São Paulo, 01218011, Brazil;*^*2*^*Public Health, Brazilian Center for Mindfulness and Health Promotion – UNIFESP, São Paulo, 04753-060, Brazil*

##### **Correspondence:** Shirlene Lopes

**Background**

Chronic pain in the nursing category has been the subject of several scientific studies pointing to the need to minimize this worldwide burden scenery. Mindfulness-based Interventions (MBI) have demonstrated promising results in clinical and non-clinical health conditions, including pain management.

**Objective**

To evaluate the feasibility and efficacy of a MBI as a complementary strategy for the management of musculoskeletal complaints in nursing assistants and technicians in a public hospital.

**Method**

We performed an open trial with pre- post- and 3-month follow-up measurements. Sixty-four workers with chronic musculoskeletal pain were recruited to participate in an eight-week one-hour sessions in an adapted MBI from the original programs. Scales were applied to anxiety, depression, attention, musculoskeletal complaints, catastrophic thoughts about pain, self-compassion and quality of life. All variables were evaluated by ANOVA and Students t- test.

**Results**

The adapted program contributed to significantly reduce levels of anxiety, depression, musculoskeletal symptoms and catastrophic thoughts about pain (p < 0.001); increased self-compassion and the perception of quality of life in physical, psychological and overall quality of life (p ≤ 0.001). The fields environment and social relationships presented relevant results only for test Students t-test post-intervention (p = 0.001, p = 0.043). The attention variable showed no statistical significance (p > 0.05) at any of the study time. The results remained in the three- month follow-up.

**Conclusion**

The program contributed to reduce painful symptoms and improve the quality of life of nursing providers with lasting effect, showing that the adapted intervention may be feasible and efficacious inside a public hospital in Brazil.

## P112 Oak bark tannins exhibit anti-allergic activity in basophils and mast cells *in vitro*

### Peter Lorenz^1^, Carsten Gründemann2, Miriam Heinrich^1^, Manuel Garcia-Käufer^2^, Franziska Grunewald^2^, Silke Messerschmidt^1^, Anja Herrick^3^, Kim Gruber^3^, Christiane Beckmann^3^, Matthias Knödler^1^, Roman Huber^2^, Carmen Steinborn^2^, Florian Stintzing^1,3^

#### ^*1*^*Department of Analytical Development and Research, Wala Heilmittel GmbH, Bad Boll/ Eckwaelden, 73087, Germany;*^*2*^*Center for Complementary Medicine, Institute for Environmental Health Sciences and Hospital Infection Control, University Medical Center, Freiburg, 79106, Germany;*^*3*^*Department of Pharmacology and Clinical Research, Wala Heilmittel GmbH, Bad Boll/ Eckwaelden, 73087, Germany*

##### **Correspondence:** Kim Gruber (kim.gruber@wala.de)

**Background**

Oak bark tannins are used for a long time as traditional treatment of dermatological disorders and eczemas because of their well-known adstringent and anti-inflammatory characteristics. Since there are indications for anti-allergic properties of tannin-containing plant extracts, we evaluated the *in vitro* capacity of constituents of an aqueous oak bark decoction (OBD) to attenuate allergic reactions.

**Methods**

To investigate active components of the OBD, fractions of low- and high-molecular weight tannins were obtained by chromatographic separation on Sephadex® LH-20 and subsequent HPLC(DAD)/LC-MSn analyses. The effect of the OBD and its fractions on degranulation and allergic mediator release were examined in basophilic cells in a photometric assay and in human mast cells by ELISA, respectively.

**Results**

In total, 29 phenolic constituents were identified from the OBD and its fractions by LC-MS analyses. The OBD and the high-molecular weight tannin fraction, which included castalagin, vescalagin, acutissimin and ellagicatechin, exhibited dose-dependent inhibitory effects on degranulation in basophilic cells and a dose-related inhibition of IL-8, IL-6 and TNF-α release in human mast cells. The *in vitro* inhibition activities were comparable to the synthetic drugs azelastine and dexamethasone.

**Conclusions**

The results obtained provide evidence that high-molecular weight tannins of oak bark inhibit degranulation and cytokine release, which are part of the early and late phase allergic reactions, respectively. Therefore, aqueous oak bark extracts might be effective in the treatment of allergic reactions and thus possibly offer new therapeutic strategies in the future.

## P113 Massage therapy for pediatric acute diarrhea: a systematic review and meta-analysis

### Taoying Lu^1^, Lixin Wang^2^, Darong Wu^1^

#### ^*1*^*The Second Affiliated Hospital of Guangzhou University of Chinese Medicine, Guangzhou, 510120, China;*^*2*^*The Affiliated Hospital of Changchun University of Chinese Medicine, Changchun, China*

##### **Correspondence:** Taoying Lu

**Purpose**

To assess the therapeutic effect of massage compared with western medication for acute diarrhea in children under the age of 6 years old.

**Methods**

All randomized controlled trials of massage therapy for pediatric acute diarrhea were searched from Chinese National Knowledge Infrastructure (CNKI) (1979–2014), Chinese Biomedicine (CBM) (1978–2014), Wan Fang Database (1982–2014), Chinese Scientific Journal Database VIP (1989–2014) and all the searches ended at June 2014. Two authors extracted data and assessed the trials quality independently. Disagreements between authors were resolved by consensus. Revman 5.2 software was used for data analysis with effect estimate presented as risk ratio (RR) with 95% confidence intervals (CI).

**Results**

A total of 17 eligible studies involving 1797 participants were included in this review. Most of the RCTs were of high risk of bias with flawed study design and poor methodological quality. Meta-analysis showed that the massage therapy had better efficacy compared with intestinal mucosa protector (RR 1.11, 95% CI 1.04–1.19) and intestinal mucosa protector plus microecological preparation (RR 1.23, 95% CI 1.13–1.34). Adverse events were not reported in any studies.

**Conclusion**

According to the above results, the efficacy of massage is superior to that of western medication for pediatric acute diarrhea. However, no confirmative conclusions can be drawn from this review due to the poor quality of the trials. More rigorous studies are needed to support clinical practice.

**Key words**

massage, acute diarrhea, randomized controlled trial, systematic review, meta-analysis

## P114 Assessing everyday resiliency: examination of the current experiences scale

### Christina M Luberto^1,2^, Daniel L Hall^1,2^, Emma Chad-Friedman^2^, Suzanne Lechner^3^, Elyse R Park^1,2^

#### ^*1*^*Harvard Medical School/ Massachusetts General Hospital, Boston, 02114, MA, United States;*^*2*^*Benson-Henry Institute for Mind-Body Medicine, Massachusetts General Hospital, Boston, MA, United States;*^*3*^*University of Miami, Miami, FL, United States*

##### **Correspondence:** Christina M Luberto (cluberto@mgh.harvard.edu)

**Background**

Resiliency is an important factor for positive health outcomes following mind-body interventions, but there is great variation in its conceptualization and assessment. Our perspective is that resiliency involves the ability to bounce back and maintain adaptive functioning in response to the chronic *everyday stressors* of daily living. To test this, we examined whether the Current Experience Scale (CES), a revised version of the Post-traumatic Growth Inventory, a well-validated measure of positive growth following trauma, could reliably and validly measure six factors of resiliency in response to everyday stress.

**Method**

Participants were 273 adults with stress-related problems who completed the CES (Mage = 45.13, SD = 13.37; 76% F, 79% white), 97 of whom (Mage = 47.99, SD = 14.58; 74% F, 95% white) completed the CES and validated measures of construct validity before and after an 8-week evidence-based mind-body resiliency intervention.

**Results**

The six-factor structure fit the data well (RMSEA = .08 [90% CI = .07–.08]; CFI = .97, TLI = .96), and internal consistency was good for the total score and all sub-factors (α = .81–.95). The total score and all but one sub-factor showed a significant increase from pre-post the resiliency intervention, demonstrating sensitivity to change (ps < .05, d = .30–.48). The CES was significantly correlated in the expected direction with all measures of convergent validity (ps < .001), and not significantly correlated with measures of discriminant validity (p = .11 to.76), demonstrating good construct validity.

**Conclusion**

The CES may be a valid and reliable measure of everyday resiliency that could be used in mind-body medicine research or clinical work. Further replication studies are warranted.

## P115 Postpartum outcomes and mindfulness practice in mindfulness-based cognitive therapy for perinatal women

### Christina M Luberto, Elyse Park, Janice Goodman

#### *Harvard Medical School/Massachusetts General Hospital, Boston, 02114, MA, United States*

##### **Correspondence:** Christina M Luberto (cluberto@mgh.harvard.edu)

**Background**

Anxiety is common during pregnancy and associated with poorer outcomes for mother and child. Our single-arm pilot study of Mindfulness-Based Cognitive Therapy (MBCT) for women with elevated anxiety during pregnancy showed significant improvements in anxiety, depression, worry, mindfulness, and self-compassion from before to after the 8-week group intervention. It remains unclear whether these improvements are maintained post-partum, and whether amount of formal mindfulness practice is correlated with outcomes. The current study examined whether 1) improvements in the outcomes above were maintained at 3-months post-partum, and 2) mindfulness practice during the intervention and post-partum was correlated with these improvements.

**Methods**

Twenty-three pregnant women (Mage = 33.5, SD = 4.40; 75% White; 71% with generalized anxiety disorder) completed validated self-report measures before and after the intervention, and 3-months post-partum.

**Results**

Post-intervention improvements in anxiety, worry, mindfulness, and self-compassion were maintained post-partum (*p’*s < .05), and post-intervention improvements in depression showed further improvements at post-partum (p < .001). Participants were generally adherent to mindfulness practice recommendations during the intervention (54%-80% weekly adherence; M = 17.31 total practice hours [SD = 7.45]), and continued practicing at one-week post-intervention (91%) and post-partum (55%). Mindfulness practice during the intervention was not significantly correlated with outcomes at post-intervention, and only significantly correlated with worry post-partum (*r* = .87, *p* < .001). Mindfulness practice post-partum was only marginally related to improved worry post-partum (p = .05).

**Conclusions**

MBCT may be associated with maintained improvements in mental health outcomes for women during pregnancy and post-partum, but the role of mindfulness practice is unclear. Research using larger samples and randomized controlled designs is needed.

## P116 Parents’ perceptions of an integrative approach to pediatric oncology treatments: a qualitative study at a University Hospital

### Sonja Luer^1^, Matthias Heri^2^, Klaus von Ammon^2^, Martin Frei-Erb^2^

#### ^*1*^*Division of Pediatric Hematology / Oncology, University Children’s Hospital, Bern, Switzerland;*^*2*^*Institute of Complementary Medicine, University, Bern, Switzerland*

##### **Correspondence:** Sonja Luer

**Background**

The Department of Pediatric Hematology / Oncology at the University Hospital Bern adopted an integrative treatment approach in addition to conventional standard-of-care oncology therapies. Based on the definition of the The Academic Consortium for Integrative Medicine & Health (2010), one part of the integrative approach is a collaboration with the Institute of Complementary Medicine at the University of Bern. The aim of this study was to investigate parents’ experiences and opinions about the integrative approach and the collaboration during cancer treatment of their children.

**Methods**

Ten volunteer parents (9 female; 1 male) of childhood cancer survivors treated at our institution were surveyed via semi-structured interviews. Questions to the following elements of an integrative approach were included in this study: (1) focus on the whole person, (2) communication and relationship, (3) inclusion of complementary medicine, and (4) the importance of empirical evidence for complementary methods. The interviews were recorded, transliterated and evaluated with qualitative context analysis (Mayring, 2000).

**Results**

Concerning the integrative approach, high satisfaction existed regarding implementation of the whole person focus, communication and relationship. The most frequent positive statements were: high satisfaction with treatment, good communication and relationship with medical professionals and the feeling of being well-informed and taken seriously. The most mentioned negative statements were about impersonal communication and not being enough responsive to the parents emotions. Regarding the integration of complementary medicine, parents asserted that openness to complementary methods depended on personal attitudes of medical professionals. Parents would have wished a higher level of inclusion of complementary medicine. Being able “to do something” and thus contributing to the child’s well-being was ranked more important by the parents than scientific evidence of complementary medicine approaches.

**Conclusions**

Statements about communication and relationship were the main part of answers suggesting the high importance for parents. Parent empowerment in the sense of contributing to the well-being of their child themselves was perceived as important. In general, evidence about complementary medicine approaches was less important to parents suggesting a need for guidance by trained medical personnel in order to avoid possibly harmful self-medication. Overall, the collaboration between the Department of Pediatric Hematology / Oncology and the Institute for Complementary Medicine was appreciated. Based on this small study, improvement would be needed in terms of communication as well as in offering a more standardized approach to optimize the integrative approach regarding complementary medicine in the future.

## P117 Effect of Chinese herbal decoction Qinlingye extract (QLYE) on the FOXO3α expression in mice with hyperuricemia renal impairment

### Weiguo Ma, Fengxian Meng

#### *Dongfang Hospital of Beijing University of Traditional Medicine, Clinical, Beijing, China*

##### **Correspondence:** Weiguo Ma

**Purpose**

To investigate the effect of Chinese herbal decoction(QLYE) on the IL-6, VCAM-1 and Foxo3a expression in mice with hyperuricemia renal impairment.

**Methods**

The rat model was induced by gavaging adenine and feeding yeast. The successful models of mice (n = 60) were randomly divided into model, positive drug and high-, medium-, low-dose of QLYE, and were administrated with distilled water (10 ml.kg-1.d-1/i.g), allopurinol (23.33 mg. kg-1. d-1/i.g) and QLYE (7.46 g.kg-1.d-1/i.g, 3.73 g.kg-1.d-1/i.g and 1.87 g.kg1.d-1/i.g) respectively. Model mice (n = 12) were used as the control group and given distilled water (10 ml. kg-1.d-1/i.g). After 8 weeks of intervention, all mice were sacrificed. RT-PCR was used to detect the mRNA transcription of Foxo3a, IL-6 and VCAM-1 in renal tissue. Western blot was used to detect the protein expression of Foxo3a in renal tissue. ELISA was used to detect the protein expression of IL-6 and VCAM-1 in serum.

**Results**

Compared with the control group, levels of IL-6 and VCAM-1 mRNA transcription and protein expression in the model group were significantly higher (P < 0.05). And levels of Foxo3a were significantly lower (P < 0.05). Compared with the model group, levels of IL-6 mRNA transcription in mediu- and low-dose groups were significantly lower (P < 0.01, P < 0.05). Levels of Foxo3a mRNA transcription in mediu-dose group were significantly higher (P < 0.05). Levels of IL-6 and VCAM-1 protein expression in all three herbal groups were remarkably lower (P < 0.01, P < 0.05). Levels of Foxo3a protein expression in all three herbal groups were remarkably higher (P < 0.01).

**Conclusion**

Kidney damage caused by high uric acid kidney tissues can appear when abnormal expression of IL-6, VCAM-1 and Foxo3a. QLYE can effectively improve the abnormal expression of both, with good renal protective effect.

## P118 A preliminary study for evaluation of cannabis - chemotherapy interactions on human colon cancer cells

### Valentina Maggini^1,3^, Eugenia Gallo^1,3^, Ida Landini^1^, Andrea Lapucci^1^, Stefania Nobili^2^, Enrico Mini^1^, Fabio Firenzuoli^3^

#### ^*1*^*Department of Experimental and Clinical Medicine, University of Florence, Florence, viale Pieraccini 6, 50139, Florence, Italy;*^*2*^*Department of Health Sciences, University of Florence, viale Pieraccini 6, 50139, Florence, Italy;*^*3*^*Referring Center for Phytotherapy, Centre for Integrative Medicine - Careggi, Universitary Hospital, 50139 Florence, Italy*

##### **Correspondence:** Valentina Maggini

**Background**

Cannabis may be used for cancer pain relief, also in concomitance with anticancer chemotherapy. However, herb-drug interactions can lead to potentially severe and even life-threatening adverse reactions. For instance, inhibition or induction of CYP enzymes by herbal compounds can alter the ADME process of co-administered drugs.

This study aims to investigate the potential effects of phytocannabinoids from Cannabis extracts (CE), on drug transporters, cannabinoid receptors and proteins involved in nociception in human colon cancer cell lines.

**Methods**

Ethanolic extracts of Cannabis flos were titrated in HPLC-MS e HPLC-MS/MS. Human colon carcinoma cells sensitive (LoVo) and resistant to doxorubicin (DOX) (LoVo/DOX) were used. Total RNA of study genes was isolated and reverse transcribed. MDR1, CNR1, CNR2 and TRPV1 gene expression levels were evaluated using the housekeeping gene, 18 s rRNA, as endogenous control to normalize data.

**Results**

THC was 0.14% in Cannabis ethanolic extract. In CE untreated cells, basal gene expression levels of CNR1, CNR2 and MDR1 were higher in Lovo/DX cells as compared to LoVo cells. No substantial differences between the two cell lines for TRPV1 expression was observed.

**Conclusions**

In vitro studies on the effects of CE and their combinations with selected anticancer drugs in human colon cancer cells are ongoing. The results of our studies upon completion will contribute to understanding in vitro interactions between cannabis extracts and anticancer agents.

## P119 Feasibility study for a community based intervention for adults with severe Chronic Fatigue Syndrome/ ME

### Clare McDermott^1^, George Lewith^1^, Selwyn Richards^2^, Diane Cox^2^, Sarah Frossell^3^, Geraldine Leydon^1^, Caroline Eyles^1^, Hilly Raphael^3^, Rachael Rogers^4^, Michelle Selby^2^, Charlotte Adler^2^, Jo Allam^2^

#### ^*1*^*Primary Care and Population Science, University of Southampton, Southampton, SO16 5ST, United Kingdom;*^*2*^*University of Cumbria, Carlisle, United Kingdom;*^*3*^*Rebuilding Your Life Project, Oxford, United Kingdom;*^*4*^*Oxfordshire CFS/ME Service, Oxford, United Kingdom*

##### **Correspondence:** Clare McDermott; George Lewith

**Background**

Chronic Fatigue Syndrome/ME (CFS/ME) is characterised by debilitating fatigue with many bedbound patients. The study aims were

- To determine whether a new intervention could be successfully delivered.
- To collect quantitative outcome data to guide the design of future studies.
- To explore qualitatively the experience of patients, carers and clinicians.

**Methods**

Mixed-methods feasibility study with qualitative and quantitative evaluation. Participants: 12 UK patients who were housebound with severe CFS/ME. Intervention: Based on recovery skills identified through a 2.5 year Patient and Public Involvement development process involving individuals with first-hand experience of recovery from CFS/ME, as well as current patients and clinicians. The resulting one year intervention, delivered by a multi-disciplinary team, included domiciliary therapy visits and optional peer support group. Quantitative outcome measures: Patient-reported and therapist-reported outcome measures (including fatigue, physical function, anxiety, depression and other variables) and electronic activity measurement.

**Results**

The study recruited and engaged twelve participants with no serious adverse events or dropouts. At end of intervention, 5/12 participants had improved in fatigue, physical function. Group mean scores improved overall for fatigue (Chalder fatigue scale), physical function (activity and physical function scale) and anxiety. Qualitative interviews suggested that the intervention was acceptable to patients, whilst also highlighting suggestions for improvement. Participants will be followed up for a further year to find out if improvements are sustained.

**Conclusion**

This is the largest study ever conducted in severe CFS/ME and shows significant recovery suggesting further studies are indicated. Treatment is uniquely based on a patient inspired intervention.

## P120 Effect of Qinlingye extract on the inflammatory signal pathway of NLRP3/TLR4/NF-κB in HKC cells

### Fengxian Meng^1^, Wen Gu^2^, Chengcheng Zhang^3^, Hua Bai^4^, Zhengju Zhang^1^, Dali Wang^1^, Xiangwei Bu^1^, Honghong Zhang^1^, Jianpeng Zhang^1^, Hui Liu^1^

#### ^*1*^*Dongfang Hospital, Beijing University of Chinese Medicine, Department of Rheumatology, Beijing, China;*^*2*^*Beijing Hospital of Traditional Chinese Medicine, Department of Rheumatology, Beijing, China;*^*3*^*Dongfang Hospital, Beijing University of Chinese Medicine, Department of Nephrology, Beijing, China;*^*4*^*Xiluoyuan community health service center of Fengtai distict, Department of Traditional Chinese Medicine, Beijing, China*

##### **Correspondence:** Fengxian Meng

**Objective**

The objective of this study was to investigate the effect of Qinlingye extract (QLYE) on the inflammatory signal pathway of NLRP3⁄ TLR4⁄ NF-κB in HKC cells.

**Methods**

HKC cells were cultured and induced by uric acid (UA) in model group. While stimulated by UA, the administered groups were intervened by high-, middle-, low-dose of QLYE (1000, 500, 250 umol⁄L). After 24, 36, 48 h of intervention, the total RNA and protein were extracted. RT-PCR was used to detect the mRNA transcription of NLRP3, TLR4 and NF-κB, Western blot was used to detect the protein expression of NLRP3, TLR4 and IκBα.

**Results**

1. Compared with the control group, the mRNA transcription of NLRP3 at 24 h, TLR4 at 36 h and NF-κB at 36, 48 h were higher in model group (P < 0.05); the protein expression of NLRP3 and TLR4 were higher, IκBα was lower at 24, 36, 48 h in model group (P < 0.05, P < 0.01).
2. Compared with the model group, the mRNA transcription of NLRP3 in three administered groups at 24 h, TLR4 in high-, middle-dose group at 36 h, and NF-κB in high-dose group at 36, 48 h were lower (P < 0.05, P < 0.01); the protein expression of NLRP3 in high-, middle-dose group and TLR4 in high-, low-dose group were lower, IκBα in high-, middle-dose group were higher at 24 h, the protein expression of NLRP3 in three administered groups and TLR4 in high-, middle-dose group were lower, IκBα in high-, middle-dose group were higher at 36 h, the protein expression of NLRP3 in middle-dose group was lower, IκBα in middle-dose group was higher at 48 h (P < 0.05, P < 0.01).

**Conclusion**

QLYE may inhibit the NLRP3⁄ TLR4⁄ NF-κB signal pathway to ameliorate the renal immune inflammatory injuries induced by UA.

## P121 Effectiveness of mindfulness- and relaxation-based eHealth interventions for patients with medical conditions: a systematic review

### Michael Mikolasek, Jonas Berg, Claudia Witt, Jürgen Barth

#### *Institute for Complementary and Integrative Medicine, UniversityHospital Zurich, Zurich, 8091, Switzerland*

##### **Correspondence:** Michael Mikolasek

**Purpose**

This systematic review aims to summarize eHealth studies with mindfulness- and relaxation-based interventions for medical conditions and to determine if eHealth interventions have positive effects on health.

**Methods**

A comprehensive search of five databases was conducted for all available studies from 1990 to 2015. Studies were included if the intervention was mainly technology delivered, included a mindfulness- or relaxation-based intervention strategy and if patients with a medical condition were treated. Treatment effects were summarized by vote counting for different outcomes.

**Results**

A total of 2383 records were identified whereof 17 studies with 1855 patients were included in this systematic review. These studies were conducted in patients with irritable bowel syndrome, chronic fatigue syndrome, cancer, chronic pain, surgery, and hypertension. All but one study were delivered online through a web-platform. One study delivered the intervention with iPods. The studies indicate that mindfulness- and relaxation-based eHealth interventions can have positive effects on patients’ general health and psychological well-being. No effects were found for stress or mindfulness. Only five studies reported economic analyses of eHealth interventions without any clear conclusion.

**Conclusion**

There is evidence that mindfulness- and relaxation-based eHealth interventions for medical conditions can have positive effects on health outcomes. No app studies were retrieved, even though a vast number of smartphone apps exist which aim at increasing users” health. Therefore, more studies investigating those health apps are needed since many of them are in use.

## P122 Efficacy of ointment containing comfrey and propolis in the treatment of mild acute sports injuries

### Ivan Miskulin^1^, Zdenka Lalic^2^, Maja Miskulin^1,3^, Albina Dumic^4^, Damir Sebo^1^, Aleksandar Vcev^4^

#### ^*1*^*Institute for Integrative Medicine, Faculty of Medicine Osijek, Josip Juraj Strossmayer University of Osijek, Osijek, 31000, Croatia;*^*2*^*APIPHARMA Ltd., Zagreb, Croatia;*^*3*^*Department of Public Health, Faculty of Medicine Osijek, Josip Juraj Strossmayer University of Osijek, Osijek, 31000, Croatia;*^*4*^*Department of Internal Medicine, Family Medicine and Medical History, Faculty of Medicine Osijek, Josip Juraj Strossmayer University of Osijek, Osijek, Croatia*

##### **Correspondence:** Ivan Miskulin

**Background**

Comfrey (*Symphytum officinale L*) is a medicinal plant with a long tradition in the treatment of painful muscular and joint complaints. On the other hand, propolis has been used since ancient times for the treatment of various conditions, wound healing treatment being one of them. The purpose of this study was to determine the efficacy of ointment containing comfrey and propolis in the treatment of mild acute sports injuries.

**Methods**

This prospective cohort epidemiological study that included 144 participants of both genders, mean age 36.9 ± 15.7 years, was conducted in the general practice setting in Osijek, Croatia, from April till September 2016. Participants were instructed to use tested ointment three times daily during three weeks. A specially designed questionnaire was used to collect demographic data and data concerning their sports injury symptoms and to evaluate the intensity of the latter data according to the scale defined in the research protocol.

**Results**

The study showed statistically significant improvements in the intensity of pain of the participants (Wilcoxon signed-rank test; p < 0.001) as well as statistically significant differences in proportions of the participants with swelling (McNemar’s test; p < 0.001) and bruise (McNemar’s test; p < 0.001).

**Conclusions**

The tested product - ointment with comfrey and propolis is proven clinically effective in eliminating pain and reducing swelling and bruising caused by various mild acute sports injuries and as such can be recommended as one of the first-line products in the treatment of the described health problems.

## P123 The use of traditional, complementary and alternative medicine (TCAM) among patients with type 2 diabetes mellitus in Yemen

### Nasr AA Mohammed^1^, Dongwoon Han^1^, Mansoor Ahmed^2^, Soo Jeung Choi^3^, Hyea Bin Im^3^, Jung Hye Hwang^3^

#### ^*1*^*Global Health and Development, Hanyang University, College of Medicine, Seoul, 04763, South Korea;*^*2*^*Hanyang University, College of Medicine, Seoul, 133-791, South Korea*; ^*3*^*Global Health and Development, Hanyang University, Seoul, South-Korea*

##### **Correspondence:** Nasr AA Mohammed

**Background**

During the last few decades, there has been a significant increase in the use of traditional, complementary and alternative medicine (TCAM) among patients with chronic illness. However, there is scarcity of evidence about the prevalence and patterns of TCAM use in patients with type 2 diabetes mellitus (DM) in Yemen.

**Methods**

A cross-sectional survey on 272 patients with type 2 DM was conducted after they were recruited from out-patient department (OPD) of four main hospitals in Sana’a. The survey asked questions on socio-demographics, diabetes characteristics and patterns of TCAM use. Descriptive statistics, chi square test and binary logistic regression was used to examine the association between dependent and independent variables.

**Results**

Use of TCAM was prevalent among 35% of the patients with type 2 DM in Yemen. Herbs were the most commonly used modality with ginger (22.4%), garlic (22.4%) and fenugreek (19.7%) the most popular. Most frequently reported reasons for using TCAM were cures the disease (34%), improvement in general health (14.2%), low cost (7.1%) and reduces side-effects of conventional medicine (6.4%). Independent predictors of TCAM use were presence of diabetes complications and perceived effectiveness of TCAM combined with western medicine.

**Conclusion**

Having strong trust in the effectiveness of TCAM, a considerable proportion of Yemini patients use these therapies for the management of type 2 DM. Healthcare providers should realize patients” choice of using TCAM and must fully understand its potential risks and benefits in order to appropriately advise their patients. There is a need to educate both the physicians and patients on the importance of discussing use of TCAM.

Keywords: Complementary and alternative medicine, chronic disease, type 2 diabetes mellitus, herbal medicine, folk medicine, Arab countries, Yemen

## P124 Elucidation of molecular mechanism of Pentahydroxy flavone isolated from Madhuca indica J. F. Gmel. Leaves against sodium arsenite-induced cardiotoxicity in rats

### Anwesha Mukherjee, Amit Kandhare, Subhash Bodhankar

#### *Poona College of Pharmacy, BVDU, Department of Pharmacology, Pune, India*

##### **Correspondence:** Anwesha Mukherjee

**Background**

Arsenic poisoning through drinking water is a major concern in the developing world and displaying various clinicopathological conditions including cardiovascular diseases.

**Aim**

To elucidate efficacy and possible mechanism of action of isolated moiety from *Madhuca indica* J. F. Gmel. Leaves against sodium arsenite (SA)-induced cardiotoxicity in rats.

**Materials and methods**

Flavone D3 was isolated from methanolic extract of *Madhuca indica* J. F. Gmel. Leaves (MI-ALC) and well characterized using HPTLC, FT-IR, 1H-NMR and LC-MS1. Cardiotoxicity was induced in Sprague-Dawley rats by SA (5 mg/ kg, p.o., 28 days) 2. Rats were either treated with vehicle (5 mL/kg, p.o.) or D3 (2.5, 5 and 10 mg/kg, p.o.) for 28 days. Various behavioral, biochemical, molecular and histopathological parameters were evaluated.

**Results**

D3 was characterized as 3,5,7,3′,4′-Pentahydroxy flavone with molecular weight as C15H10O7. Chronic administration of SA significantly altered myocardial functions (electrocardiographic, hemodynamic and left ventricle contractile functions) whereas administration of D3 (5 and 10 mg/kg) significantly (*p <* 0.05) attenuated these alteration. Elevated levels of cardiac markers (LDH, CK-MB, AST, ALT, and ALP) and altered lipid metabolism (total cholesterol, triglyceride, LDL, HDL, and VLDL) after SA administration were decreased by D3 (5 and 10 mg/kg). It also significantly decreased (*p <* 0.05) the elevated level of cardiac oxido-nitrosative stress and significantly increased (*p <* 0.05) myocardial mitochondrial enzymes (I-IV) activity. It significantly restored (*p <* 0.05) SA-induced alteration in cardiac Nrf-2, HO-1, Smad-3, and TGF-β mRNA expressions. Flow cytometric analysis revealed that SA-induced myocardial apoptosis was attenuated by D3 (5 and 10 mg/kg) treatment. D3 reduces histopathological aberrations (measured using hematoxylin-eosin stain, masson’s-trichrome stain and transmission electron microscopy) induced by SA.

**Conclusion**

Pentahydroxy flavone isolated from MI-ALC ameliorates SA-induced cardiotoxicity via modulation of TGF-β/Smad-3 and Nrf-2/HO-1 pathways along with a reduction in myocardial apoptosis.

Keywords: Apoptosis, Cardiotoxicity, Flow cytometric analysis, HO-1, Madhuca indica, Nrf-2, Pentahydroxy flavone, Smad-3, Sodium arsenite, TGF- β, Transmission electron microscopy

**References**

1. Mohod SM, Kandhare AD, and Bodhankar SL. J Ethnopharmacol. 2016;182:150–159.

2. Adil M, Kandhare AD, Ghosh P, and Bodhankar SL. Chem Biol Interact. 2016;253:66–77.

## P125 Chrysin ameliorates pressure overload-induced cardiac hypertrophy in rats via modulation of endogenous biomarkers and oxidative stress

### Anwesha Mukherjee^1^, Amit Kandhare^1^, Prasad Thakurdesai^2^, Subhash Bodhankar^1^

#### ^*1*^*Poona College of Pharmacy, BVDU, Department of Pharmacology, Pune, India;*^*2*^*Indus Biotech Private Limited,, Pune, India*

##### **Correspondence:** Anwesha Mukherjee

**Background**

Congestive heart failure is a chronic complex syndrome leading to both hemodynamic and electrographic abnormalities, along with alterations in metabolic and neurohormonal conditions. Chrysin (5, 7-dihydroxyflavone) is a flavonoid possesses antioxidant, antihypertensive and anti-inflammatory potential1.

**Aim**

To investigate the effect of chrysin (3, 10, 30 mg/kg) on pressure overload induced cardiac hypertrophy in rats.

**Method**

Cardiac hypertrophy was induced in male Wistar rats (150–200 g) by suprarenal abdominal aorta constriction2 and they were randomly divided into various groups namely, sham, aortic stenosis and aortic stenosis + chrysin (3, 10, and 30 mg/kg, p.o.). Chrysin was administered 4 weeks after surgery. The degree of cardiac hypertrophy was determined at the end of treatment by hemodynamic parameters (including ECG, blood pressure, left ventricular function), cardiac biomarkers, antioxidant levels and histopathological evaluation.

**Results**

The significant and dose-dependent restoration (*p* < 0.01 and *p* < 0.001) of the hemodynamic parameter along with left ventricular function was observed after 4 weeks of treatment of chrysin (10 and 30 mg/kg). Pressure overload induced decreased in levels of superoxide dismutase, reduced glutathione, and membrane-bound inorganic phosphate enzymes (Na + K + ATPase and Ca2 + ATPase) were significantly and dose-dependently (*p* < 0.01 and *p* < 0.001) elevated by chrysin treatment. Moreover, it also significantly and dose-dependently (*p* < 0.01 and *p* < 0.001) attenuated an elevated levels of cardiac biomarkers (creatine kinase-MB and lactate dehydrogenase enzymes), as well as the activities of malondialdehyde, aspartate aminotransferase and lactate dehydrogenase. Histological aberration induced after pressure overload was restored by chrysin treatment.

**Conclusion**

Chrysin ameliorates development of cardiac hypertrophy via mitigating the endogenous biomarkers, oxidative stress and decreasing hemodynamic alterations in pressure overload-induced rats.

Keywords*:* Cardiac hypertrophy, Chrysin, Hypertension, Oxidative stress, Reactive oxygen species

**References**

1. Kandhare AD, Shivakumar V, Rajmane A, Ghosh P, and Bodhankar SL. J Nat Med. 2014;68(3):586–603.

2. Visnagri A, Kandhare AD, Ghosh P, and Bodhankar SL. Cardiovasc Endocrinol. 2013;2(4):85–97.

## P126 Care-ally assisted massage training and application to address chronic neck pain in US veterans: a 4-year clinical trial

### Niki Munk^1^, Erica Evans^1^, Amanda Froman^2^, Matthew Kline^2^, Matthew J Bair^1^

#### ^*1*^*Health Sciences, Indiana University School of Health and Rehabilitation Sciences, Indianapolis, 46202, IN, United States;*^*2*^*Roudebush VA Medical Center, Indianapolis, IN, United States*

##### **Correspondence:** Niki Munk

**Background**

Evidence is robust supporting massage for chronic neck pain (CNP) with multiple, hour long sessions per week for multiple weeks showing the greatest effects. However, barriers such as out-of-pocket costs and logistics exist preventing Veterans from effective care access. Curative approaches for chronic conditions needing longitudinal management are only temporarily helpful. This presentation outlines the massage training and protocol developed for Veteran/Care-ally dyads to address chronic neck pain in a three-armed, randomized clinical trial.

**Methods**

Informed by prior research, clinical massage practice, and education experience, a care-ally and self-applied massage training approach and protocol was developed for Veterans with CNP. Veteran/Care-ally dyads (N = 156) will be randomized to the massage training arm and compared to therapist-delivered and wait-list control arms.

**Results**

The care-ally assisted massage training includes a 4.5 hour training session with three components: training workshop, instructional DVD, and training manual. Trainings are held 2–3 times per month for 8–10 participant dyads. The trainings include general massage and context instruction, technique demonstration, supervised practice, individualized self-care, care-ally applied trigger point strategies, protocol practice, and home application adaptation strategies. The 30-minute care-ally assisted massage protocol addresses head, neck, shoulders, arms, back, and chest; and includes basic Swedish techniques, lymph drainage, and myofascial trigger point treatment and stretching. Veteran/care-ally dyads are supported with follow-up and maintenance instruction as needed.

Recruitment is underway and the study is designed to determine the extent to which Veteran care-ally assisted massage training and application addresses chronic neck pain. Study completion is expected in 2020.

## P127 The “USB” (Use – Safety - Benefit) principle as a methodology to integrate patient experiences into the construction of an intervention program using survey data

### Frauke Musial^1^, Agnete E Kristoffersen^1^, Terje Alræk^1^, Harald J Hamre^2^, Trine Stub^1^, Lars Björkman^3^, Vinjar M Fønnebø^1^

#### ^*1*^*National Research Center in Complementary and Alternative Medicine, NAFKAM, Department of Community Medicine, The Arctic University of Norway, UiT, Tromsø, 9037, Norway;*^*2*^*Institute for Applied Epistemology and Medical Methodology, University of Witten-Herdecke, Herdecke, Germany;*^*3*^*Dental Biomaterials Adverse Reaction Unit, Uni Health, Uni Research, Bergen, Norway*

##### **Correspondence:** Frauke Musial

**Background**

For clinical trials, patient participation is now demanded from all Norwegian national funding sources and is often operationalized through councils. The Use – Safety - Benefit (USB) principle introduced here allows to carry user participation even further, by using a survey among the target group for the construction of an intervention. It is particularly useful in situations where there is no scientific evidence for the risk/benefit profile of an intervention available.

**Methods**

A survey of experiences with therapeutic interventions was distributed to all members of a Norwegian patient association (n = 999; response rate: 36.4%). Therapies were ranked according to I) the number of users; II) risk by using the percentage of all participants reporting worsening; III) perceived benefit. Statistical criteria were used for exclusion of therapies at each step. Moreover, a quantitative methodology for the measurement of patients’ experiences and preferences of a patient population the “Patient Experience-based Benefit/Risk Index” (PEBRI) was developed.

**Results**

Of n = 26 therapies included in the survey, n = 13 therapies remained after the stepwise selection and were in different ways included into the intervention program developed for the trial. The conduct of a survey among the target group was a probate and feasible methodology in order to operationalize patient participation in the development of a treatment program.

**Conclusion**

In a situation where there is no scientific evidence for the risk/benefit profile of the preferred interventions available, the USB (use-safety-benefit) approach provides a helpful guide for decision making and the implementation of patient participation.

## P128 Different intervention strategies for preventing type 2 diabetes mellitus: a systematic review and meta-analysis of controlled trials

### Bing Pang^1^, Feng-mei Lian^1^, Qing Ni^1^, Xiao-lin Tong^1^, Xin-long Li^2^, Wen-ke Liu^1^, Shuo Feng^3^, Xi-yan Zhao^1^, Yu-jiao Zheng^1^, Xue-min Zhao^1^, Yi-qun Lin^1^

#### ^*1*^*Department of Endocrinology, Guang anmen Hospital of China Academy of Chinese Medical Sciences, Beijing, 100053, China;*^*2*^*Institute of Basic Research in Clinical Medicine, China Academy of Chinese Medical Sciences, Beijing, China;*^*3*^*Centre for Evidence-Based Chinese Medicine, Beijing University of Chinese Medicine, Beijing, China*

##### **Correspondence:** Qing Ni; Xiao-lin Tong

**Background**

Different intervention strategies can prevent type 2 diabetes (T2DM). This systematic review was to assess the efficacy of different strategies on preventing T2DM among Chinese subjects with prediabetes.

**Methods**

Seven electronic databases were searched to identify eligible trials published beforeJune 1, 2016. Studies were grouped into 11 different strategies: 1: Traditional Chinese patent medicine (TCPM); 2: diet plus exercise intervention; 3: diet intervention; 4. exercise intervention; 5–8: anti-diabetic drugs [metformin, α-glucosidase inhibitors, glitazones, beta-cell stimulating drugs]; 9. lipid-affecting drugs;10: anti-hypertensive drugs; 11: Sitagliptin. Controlled trials on T2DM prevention in Chinese prediabetic subjects, with a minimum follow-up duration of 6 months and which provided outcomes on T2DM incidence were included for analysis. Primary outcome was the incidence of diabetes. Data extraction and quality assessment were performed according to the Cochrane review standards. A random effect model was used to analyze outcomes which were expressed as odds ratio (OR), with 95% confidence intervals (CI), I2 statistics were used to assess heterogeneity.

**Results**

Seventy-seven studies with a total of 10628 participants met the inclusion criteria. Ten kinds strategies, except for lipid-affecting drugs, were able to prevent T2DM, with different efficacy, from 0.19 (0.08–0.47) to 0.46 (0.24–0.88). Efficacy of TCPM [0.38(0.29–0.49)] was similar with that of α-glucosidase inhibitors. At meta-regression analysis, body mass index and sample sizewere associated with the efficacy of intervention.

**Conclusions**

TCPM is effective for reducing the incident diabetes, which may bring a new approach to prevent T2DM.

The PROSPERO registration no. CRD42016047935.

## P129 The efficacy and safety of Chinese herbal medicine Jiang Zhuo Formula in metabolic syndrome patients with dyslipidemia: a randomized, placebo-controlled trial

### Bing Pang^1^, Feng-mei Lian^1^, Xiao-lin Tong^1^, Tian-yu Zhao^2^, Xi-Yan Zhao^1^, Hui Che Phd^3^, Chen Zhang^1^

#### ^*1*^*Department of Endocrinology, Guang anmen Hospital of China Academy of Chinese Medical Sciences, Beijing, 100053, China;*^*2*^*Endocrinology, First Teaching Hospital of Tianjin University of Traditional Chinese Medicine, Tianjin, China;*^*3*^*Digestion, Xiyuan Hospital of China Academy of Chinese Medical Sciences, Beijing, China*

##### **Correspondence:** Feng-mei Lian; Xiao-lin Tong

**Aim**

To assess the efficacy and safety of Jiang Zhuo formula (JZF) in metabolic syndrome (MetS) patients with dyslipidemia.

**Methods**

Adult MetS patients with dyslipidemia [triglyceride (TG) 2.26–7.0 mmol/L] were enrolled. After a two-week run-in period, 159 subjects were randomly assigned to JZF group or placebo group. The period of treatment was 12 weeks. Primary endpoint was the change of TG. Secondary endpoints included changes of waist circumstance, body mass index (BMI), fasting plasma glucose (FPG), and adverse events. Per-protocol analyses were applied to data analysis. Data were analyzed using SPSS 22.0. A significant difference was considered when P < 0.05.

**Results**

154 patients were included for per-protocol analysis (77 for JZF and 77 for placebo) with baseline matched. After12 weeks of treatment, JZF reduced mean TG by 0.48 ± 1.40 from baseline and placebo reduced mean TG by 0.20 ± 1.38 (P = 0.619). For patients with dyslipidemia that untreated previously, JZF reduced mean TG by 0.63 ± 1.20 from baseline and placebo by 0.15 ± 1.32 (P = 0.004); for patients with hypertriglyceridemia, JZF reduced mean TG by 1.30 ± 1.06 from baseline and placebo by 0.34 ± 1.32 (P = 0.010). Compared to placebo, JZF could significantly decrease waist circumstance (-4.21 ± 4.66 vs. -2.16 ± 4.59 cm; P = 0.019) and BMI (-0.62 ± 1.25 vs. -0.19 ± 1.09 kg/m2; P = 0.036). JZF and placebo reduced mean FPG (-0.14 ± 0.66 vs. -0.04 ± 0.63 mmol/L; P = 0.094). Adverse events were reported for 5 casesin the JZF group and 3 casesin the placebo group (P > 0.05).

**Conclusion**

JZF could improve dyslipidemia and other cardiovascular risk factors, especially in patients with dyslipidemia that untreated previously or hypertriglyceridemia.

ClinicalTrials.gov; NCT01265160.

## P130 Research on a new physiotherapy device as an intervention for Chinese overweight and obese patients: before-after study in the same patients

### Bing Pang^1^, Feng Liu^2^, Xiao-lin Tong^1^, Lin-hua Zhao^1^, Xue-min Zhao^1^, Ru Ye^1^, Cheng-juan Gu^1^

#### ^*1*^*Department of Endocrinology, Guang anmen Hospital of China Academy of Chinese Medical Sciences, Beijing, 100053, China;*^*2*^*Obesity research center, Beijing Wuguanda Biological Technology co., LTD., Beijing, 100053, China*

##### **Correspondence:** Feng Liu; Xiao-lin Tong

**Aim**

To assess the effectiveness and safety of a new physiotherapy device on reducing weight and body mass index (BMI) in Chinese subjects.

**Methods**

Electronic records between Jan 1, 2012 and Oct 1, 2015 were used as database. Subjects with a BMI > 25 was eligible for inclusion. The primary outcomes were the changes in body weight and BMI. The secondary outcome were adverse events. Data were analyzed using Statistical Product and Service Solutions software (SPSSStatistics 22.0). A significant difference for statistical analysis was considered when P < 0.05.

**Results**

481 subjects were included. There was significant improvement in the difference of body weight values between every return visit and baseline (1–0 week: −2.73 ± 1.63 kg, P < 0.001; 2–0 week: −4.09 ± 2.05 kg, P < 0.001; 4–0 week: −6.19 ± 2.92 kg, P < 0.001; 6–0 week: −8.66 ± 3.56 kg, P < 0.001; 12–0 week: −10.11 ± 4.05 kg, P < 0.001; 16–0 week: −10.83 ± 3.36 kg, P < 0.001). BMI were also improved, there was significant difference between every return visit and baseline (1–0 week: −1.01 ± 0.59 kg/m2, P < 0.001; 2–0 week: −1.53 ± 0.73 kg/m2, P < 0.001; 4–0 week: −2.32 ± 1.07 kg/m2, P < 0.001; 6–0 week: −3.15 ± 1.22 kg/m2, P < 0.001; 12–0 week: −3.58 ± 1.40 kg/m2, P < 0.001; 16–0 week: −3.57 ± 1.43 kg/m2, P < 0.001). No severe adverse events occurred.

**Conclusions**

The device might be short-term effective in reducing body weight and BMI, which might be mediated through inducing lipolysis and increasing satiety. However, prospective, controlled, and longer follow-up duration studies are still required.

## P131 Prevention of type 2 diabetes mellitus with the traditional Chinese patent medicine: a systematic review and meta-analysis

### Bing Pang^1^, Qing Ni^2^, Xiao-lin Tong^2^, Feng-mei Lian^2^, Xi-yan Zhao^2^, De Jin^2^, Xue-min Zhao^2^, Yu-jiao Zheng^2^, Yi-qun Lin^2^

#### ^*1*^*Traditional Chinese medicine, Centre for Evidence-Based Chinese Medicine, Beijing University of Chinese Medicine, Beijing, Beijing, China;*^*2*^*Department of Endocrinology, Guang anmen Hospital of China Academy of Chinese Medical Sciences, Beijing, 100053, China*

##### **Correspondence:** Qing Ni; Xiao-lin Tong

**Aim**

To systematically assess the evidence on the effectiveness and safety of traditional Chinese patent medicine (TCPM) on prevention of type 2 diabetes (T2DM).

**Methods**

Seven electronic databases were searched from their inception to June 2016. Randomized controlled trials that used TCPM as an intervention or co-intervention with lifestyle modification (LM) versus LM alone, in patients with prediabetes were included for analysis. Primary outcome was the incidence of diabetes. Secondary outcomes were the normalization of blood glucose and adverse events (AEs). Data extraction and quality assessment were performed according to the Cochrane review standards. RevMan 5.2 software was applied for data analysis.

**Results**

Thirty trials with a total of 4239 participants were included. Eleven trials had a Jadad score ≥ 3. Results showed that subjects who received TCPM plus LM were less likely to progress to T2DM compared with LM alone (RR 0.49; 95% CI 0.42 to 0.58). There were also no significant differences in the adverse events in both groups (RR 0.94; 95% CI 0.65 to 1.35). Subjects who received TCPM plus LM were also more likely to have their blood glucose levels return to normal levels compared with LM alone (RR 0.74; 95% CI 0.70 to 0.78). The most frequently used five herbs were Huangqi, Dihuang, Huanglian, Shanyao and Tianhuafen.

**Conclusions**

In patients with prediabetes, TCPM reduced the risk of progression to T2DM, and increased the possibility of regression towards normoglycaemia, and TCPM was safe to use.

Trial registration number: The PROSPERO registration no. CRD42016046553

## P132 Predictors of arthritis management amongst older Chinese people in a nationally representative survey

### Wenbo Peng, Romy Lauche, David Sibbritt, Jon Adams

#### *Faculty of Health, University of Technology Sydney, Sydney, 2007, Australia*

##### **Correspondence:** Wenbo Peng

**Aim**

To examine the prevalence of arthritis amongst older Chinese population and the predictors of their managements of arthritis.

**Methods**

This study was conducted as part of the 2013 China Health and Retirement Longitudinal Study, a nationally representative survey focusing upon people over 45-year old. Predictors of each treatment of arthritis were evaluated by multivariable logistic regression, including demographic characteristics, health status, health service utilisation and common co-morbidities.

**Results**

There are 6404 participants (54.3%) with diagnosed/self-reported arthritis. Amongst these people, 58% are female, 90% are Han ethnic origin, 39% rated their health poor, 79% were covered by the New-Rural-(Residents)-Cooperative-Medical-Scheme and 8% were covered by the Urban-Employees-Medical-Insurance. Additionally, 67% and 92% of people with arthritis experienced hypertension and diabetes, respectively. Participants who did not treat their arthritis (41.5%) are more likely to be with Urban-Employees-Medical-Insurance (OR = 1.33), have hypertension (OR = 1.97) and/or diabetes (OR = 1.25). Participants who used Western-medicine-only treatment (22.5%) for their arthritis are less likely to be Tibetian ethnic origin (OR = 0.37) and/or with Urban-Employees-Medical-Insurance (OR = 0.64). Participants who used Chinese-herbal-medicine-only treatment (6.7%) for their arthritis are more likely to be Tibetian/Mongol/Miao/Yi ethnic origin (OR = 3.02–7.16) and with poor self-rated health status (OR = 1.44). Only female (OR = 1.43) is the predictor of the Chinese-Western integrative medicine treatment (5.9%) for arthritis.

**Conclusions**

The prevalence of arthritis is high amongst older Chinese population. However, 42% of these people did not treat their arthritis. Policy-makers should be aware of this important issue and further studies are warranted to determine the cause for older people’s choice of each treatment of arthritis.

## P133 Herbal medicines use and healthy lifestyle: a national survey of young Australian women

### Wenbo Peng^1^, Jon Wardle^1^, Holger Cramer^2^, Gita Mishra^3^, Romy Lauche^1^

#### ^*1*^*Faculty of Health, University of Technology Sydney, Sydney, 2007, Australia;*^*2*^*Faculty of Medicine, University of Duisburg-Essen, Essen, Germany;*^*3*^*School of Public Health, University of Queensland, Brisbane, Australia*

##### **Correspondence:** Wenbo Peng

**Background**

Women who use herbal medicines self-prescribed/prescribed (by naturopaths/herbalists) generally assume they have health lifestyle. Recommendations for a healthy lifestyle are also part of naturopathic treatment. This study aims to examine the associations of herbal medicines use with healthy lifestyle behaviours in young Australian women.

**Methods**

Women in 1973–1978 cohort (31–36 years, n = 8200) and 1989–1995 cohort (16–25 years, n = 11345) from the Australian Longitudinal Study on Women’s Health were asked about their smoking/alcohol status, Marijuana/illicit drug use, physical activity and dietary behaviour in the previous year. Multiple logistic regressions were used to analyse the associations between such health behaviours and their consultations with naturopaths/herbalists and herbal medicines utilisation.

**Results**

Approximately 9% (1989–1995 cohort) to 12% (1973–1978 cohort) of women consulted naturopaths/herbalists, and 15% (1989–1995 cohort) to 20% (1973–1978 cohort) used herbal medicines at least “sometimes”. Small overlaps were found between the consultation and utilisation in both cohorts. Women consulting with naturopaths/herbalists were only associated with non-smoker (OR = 0.61) and vegetarian diet (OR = 1.37) in the 1973–1978 cohort. Women using herbal medicines “often” were more likely to be physically active (1973–1978 cohort: OR = 1.47; 1989–1995 cohort: OR = 1.45), follow a vegetarian diet (1973–1978 cohort: OR = 1.69) and use Marijuana or illicit drugs (1989–1995 cohort: OR = 1.15–1.31).

**Conclusions**

Herbal medicines use is associated with some healthy lifestyle behaviours (i.e. exercise, vegetarian diet), while it is also associated with the Marijuana and illicit drugs use amongst young women. Further studies are required to investigate the reasons and impact on women’s health regarding the concurrent use of drugs and herbal medicines.

## P134 Preliminary findings from an evaluation of patient safety culture of chiropractors in three Canadian provinces

### Katherine A Pohlman^1,2^, Silvano Mior^3^, Martha Funabashi^1^, Diana De Carvalho^4^, Mohamed El-Bayoumi^5^, Bob Haig^6^, Kimbalin Kelly^6^, Darrell J Wade^7^, Maeve O’Beirne^8^, Sunita Vohra^1^

#### ^*1*^*University of Alberta, Edmonton, T6G 2C8, Canada;*^*2*^*Parker University, Dallas, United States;*^*3*^*Canadian Memorial chiropractic College, Toronto, Canada;*^*4*^*Memorial University of Newfoundland, St. John’s, Canada;*^*5*^*New Brunswick Chiropractors’ Association, Fredericton, Canada;*^*6*^*Ontario Chiropractic Association, Toronto, Canada;*^*7*^*Newfoundland & Labrador Chiropractic Association, St. John’s, Canada;*^*8*^*University of Calgary, Calgary, Canada*

##### **Correspondence:** Martha Funabashi (funabash@ualberta.ca)

**Background**

Healthcare in the 21st century is focusing on value within a safe and high quality system; yet little is known about such system measures in the chiropractic profession. A cross-sectional survey was conducted to evaluate patient safety attitudes and opinions and quality improvement dimensions among members of chiropractic associations in Ontario, New Brunswick, and Newfoundland and Labrador.

**Methods**

SafetyNETs Survey to Support Quality Improvement was emailed to a total of 3471 chiropractic association members in Ontario (n = 3332), New Brunswick (n = 75) and Newfoundland and Labrador (n = 64).

**Results**

Overall response rate was 6.8% (n = 236/3471): Ontario-5.7%; New Brunswick-20%; Newfoundland and Labrador-48.4%. Most respondents were male, in solo practices for a mean of 19.1 years, with 50–150 patient visits weekly. Respondents in all provinces had similar moderate-high patient safety dimension scores, except one province, that reported statistically different (p < 0.05) moderate scores in *staff training* and *communication about error*. While still modest, *patient care tracking* had the lowest overall score. Information exchange with insurance companies was reported problematic among all respondents. Access to care was a concern in Newfoundland and Labrador (13% regular problem), yet not in Ontario (3% regular problem) or New Brunswick (0% regular problem).

**Conclusion**

This is the first study to show the positive patient safety culture among Canadian chiropractors from those three provinces. Despite the low response rate, opportunities for collaboration across Canadian provinces to improve specific patient safety areas were identified. Further efforts are necessary to emphasize the necessity for the chiropractic profession to develop an evidence-supported patient safety culture.

## P135 The adaptation for Italy of the questionnaire on autonomic regulation: an innovative tool for conventional and CAM health professionals and patients’ self awareness. Preliminary results of a patients’ self-administered version

### Emanuela Portalupi^1,2^, Giampietro Gobo^3^, Luigi Bellavita^1,4^, Chiara Guglielmetti^5^

#### ^*1*^*ARESMA, Milano, Italy;*^*2*^*ARTOI, Roma, Italy;*^*3*^*University of Milano, Dept. of Philosophy, Milano, Italy;*^*4*^*Politecnico of Milano, School of Design, Milano, Italy;*^*5*^*University of Milano, DEMM, Dept. of Economics, Management and Quantitative Methods, Milano, Italy*

##### **Correspondence:** Emanuela Portalupi

**Background**

Questionnaires on autonomic regulation (aR-Q) were developed by anthroposophic medicine (AM) at FIH, Berlin. In particular, an 18 item trait scale has been studied both in healthy people and ill patients, to evaluate the constitutional background and to introduce in medicine a salutogenetic-based practical measure.

AR-Q focuses on the regulation and rhythms of autonomic functions to access individual complexity, according to AM, which depicts human beings as integrated systems of physical, living, psychic and spiritual levels.

The study aims to define the process of translation and cross-cultural adaptation of the aR-Q into Italian in order to obtain a patients’ self-administered version. The Italian version for health professionals will follow.

**Methods**

This study employed the guidelines proposed by the International Society for Pharmacoeconomics and Outcomes Research (ISPOR) Task Force for Translation and Cultural Adaptation, which define 10 steps for translation and cross-cultural adaptation of self-report instruments.

Additionally, semantic equivalence tools (cognitive interview and think aloud protocol) were employed to select the final versions of terms used. Twenty volunteers of different levels of health literacy were interviewed to assess their level of comprehension of the items.

**Results**

Analysis of the back-translation and of the cognitive interview showed that some of the symptoms terms and some of the items response sets needed to be adjusted to be understood by patients.

**Conclusion**

The patients’ self-administered version of aR-Q is an agile and sustainable tool for conventional and integrative doctors, nurses, psychologists, counselors and therapists interested in a whole person approach.

The questionnaire is designed for clinical practice and research.

Keywords: autonomic regulation, anthroposophic medicine, self-administrated patient-centered questionnaire, cognitive interview, questionnaire validation.

## P136 Acupoint stimulation in treating oculomotor palsy due sinusitis: a single case study

### Christa Raak^1^, Myriam Teuber^2,3^, Friedrich Molsberger^2,3,4^

#### ^*1*^*Institute for Integrative Medicine, Witten/Herdecke University, Herdecke, 58313, Germany;*^*2*^*H:G - Hochschule für Gesundheit und Sport GmbH, Berlin, 10367, Germany;*^*3*^*Acupuncture Research Group, Düsseldorf, Germany;*^*4*^*Naturopathy and Acupuncture, Medical Office, Berlin, 14197, Germany*

##### **Correspondence:** Christa Raak; Friedrich Molsberger

**Objective**

This case study reports the effect of acupoint stimulation and Yamamoto New Scalp Acupuncture (YNSA) in a 75-year old male retiree with oculomotor nerve palsy.

**Background**

Oculomotor nerve palsy is an ophthalmological disease resulting from damage to the cranial nerve which controls the eye movement muscles. Oculomotor palsy can arise from a number of different conditions.

**Case description**

The patient has a 7-year history of oculomotor palsy with unknown origin of the right eye. The applied therapy consisted of seven consultations in which YNSA, classic acupuncture and craniosacral therapy. After this treatment normal eye coordination without diplopia was archived. After having seen the patient again due to an acute oculomotor palsy of the left eye after a sinusitis in the year 2014, former therapy was applied again without success. Thus, periocular acupoint stimulation by vibration of the points BL2, GB1, ST2 and Yuyao was applied 3x1 d for three minutes for ten days with an electrical tooth brush.

**Results**

Within this treatment the patient showed complete recovery from oculomotor palsy without any impairment of eye movement and no eye deviation until today (two-year observation period).

**Discussion**

This case shows a multimodal approach to oculomotor palsy using periocular acupoint stimulation by vibration. Although this single case provides substantial evidence, less is known about the working principles of this treatment approach. Thus further single cases should be collected before carrying out clinical trials. This case may aid in defining the role of acupoint stimulation in the treatment of oculomotor palsy.

## P137 Cuprum aceticum as a new treatment in ascites therapy – a case report

### Ulrich von Rath

#### *Institute of Family Medicine, University Hospital Schleswig-Holstein, Campus Luebeck, Luebeck, 23570, Germany*

**Purpose**

Ascites leads to severe problems due to decompensation of liver cirrhosis. However, a conventional multi drug treatment has its limitations. Patients could suffer from drug side effects and are in serious medical condition. Therefore, the aim was to evaluate the potential value of Cuprum aceticum for patients with liver cirrhosis.

**Method**

Two patients with decompensated liver cirrhosis have reached the limit of conventional drug therapy. Ascites persisted and severe side effects occurred such as renal failure.

Cuprum aceticum D3 Dilution (Weleda) [1, 2] was found to be effective in diastolic hypertension and relaxation of splanchnic circulation. It was prescribed as an oral medication two times a day 0.5 ml (1 ml D3 = about 1 mg of Copper(II)-acetate monohydrate). Both patients were given Cuprum aceticum combined with conventional multi drug treatment and controlled over time.

**Results**

Within four-weeks-time liver syntheses measured as cholinesterase activity recovered from 3.296 to 4.649 U/l (3.930–10.300) in one of the patients. Ascites was reabsorbed completely in both patients within two month time. Signs of inflammation decreased, conventional treatment was reduced by 50% in the following month and medical condition of patients was improved.

**Conclusions**

Cuprum aceticum as an additional therapy could improve conventional multi drug treatment of decompensated liver cirrhosis [3]. Ascites was completely reabsorbed. The conventional therapy could be reduced within three month of treatment. No adverse effects were observed.

However, future research on a larger sample of patients with liver cirrhosis is needed to verify the effect of Cuprum aceticum.

**Consent to publish**

The patients reported declared their consent for the publication of their data.

**References**

1. Gesellschaft Anthroposophischer Ärzte (Ed). Vademecum Anthroposophischer Arzneimittel. 3. Auflage. Der Merkurstab. Supplement 2013; 66. p.264f.

2. Engel W. Kalium aceticum comp. – Die Vielfalt des Weines im pharmazeutischen Herstellungsprozess. Der Merkurstab 2010; 63(2):112–122.

3. Rath U v. Polyneuropathien: Zu ihrem Verständnis und zu erweiterten Behandlungsoptionen aus anthroposophisch-ärztlicher Sicht. Der Merkurstab 2016; 69 (4): 280–290.

## P138 Tolerance and effect of a prophylactic treatment with a cough medicine containing ivy leaves dry extract in children with recurrent wheezy bronchitis

### Ulrike Reichelt^1^, Uta Schwanebeck^2^, Sabine Zeil^3^, Christian Vogelberg^3^

#### ^*1*^*Technische Universität Dresden, Medizinische Fakultät Carl Gustav Carus, Dresden, Germany;*^*2*^*Koordinierungszentrum für klinische Studien, Dresden, Germany;*^*3*^*Universitätsklinikum Carl Gustav Carus, Klinik und Poliklinik für Kinder- und Jugendmedizin, Dresden, Germany*

##### **Correspondence:** Ulrike Reichelt

Ivy leaf dried extract (ILDE) is a well-studied, safe and tolerable therapy in patients with respiratory diseases associated with productive cough. Some ingredients of ILDE are known for anti-inflammatory effects which are related to the leukotriene pathway. This suggests a potential prophylactic effect of ILDE in the therapy of recurrent wheezy bronchitis (RWB). In this randomised, double-blind, placebo-controlled, monocentric clinical trial, 60 infants (aged 1–3) who had at least 3 documented episodes of wheezy bronchitis during the last 12 months were investigated. Patients were to take in 2,5 ml of the preparation (study drug/placebo) twice a day for 28 days. During this period and the follow-up time (56 days), caregivers recorded infants symptoms and additional medication in a diary card. Additionally, patients were monitored in the clinic three times (day 0, day 28, day 84). For the primary endpoint number of days between the patients inclusion and their first bronchitis during the time of the trial, no significant difference (p = 0,386) between both interventions could be shown. Also for the secondary endpoints, i.e. length and severity of bronchitis episodes, additional application of β-agonists and corticosteroids, number of days without bronchitis overall, number of days without bronchitis after the treatment period, as well as safety and tolerability of the preparation, no significant difference could be proved. This proof-of-concept study cannot prove a difference in the prophylactic application between ILDE and placebo for infants suffering from RWB. However, it would be interesting to investigate the potential prophylactic effect during long-term treatment.

Trial registration: Current Controlled Trials EudraCT-Number: 2013-001978-25

## P139 Total lymphocyte count in cancer patients with lymphopenia treated with intravenous vitamin C: results of an observational study

### Dolores Rodríguez Veintimilla^1^, Claudia Vollbracht^2^, Guerrero Tapia Mery^1^, Marisol Maldonado Villavicencio^1^, Sandra Herrera Moran^1^

#### ^*1*^*Clinical Nutrition and Dietetics, SOLCA Cancer Hospital, Guayaquil, 000, Ecuador;*^*2*^*Pascoe Naturmedizin, Gießen, 35394, Germany*

##### **Correspondence:** Dolores Rodríguez Veintimilla

**Background**

Lymphopenia commonly occurs in cancer patients and predicts poor prognosis. It is caused by radio- and chemotherapy, with malnutrition and treatment-related oxidative stress playing key roles in its pathogenesis. Tumour-related morbidity is reported to be associated with reduced plasma ascorbate, which is a key physiological antioxidant and essential factor in immune function.

**Method**

A prospective observational study was conducted on 48 cancer patients with lymphopenia (<1500/μL) to investigate the total lymphocyte count (TLC) during four weeks of elective adjuvant treatment with intravenous (iv) vitamin C 7.5 g (Pascorbin®7.5 g) once a week . TLC values at baseline (just prior to start of treatment) and after 4 weeks treatment were compared using descriptive statistics.

**Results**

After 4 weeks IV vitamin C 7.5 g, TLC increased by a mean of 211/μL (p = 0.0018). Subgroup analyses showed that, in patients (n = 25) with severe lymphopenia (TLC <1000/μL), the increase in TLC was greater with a mean rise of 368/μL (p = 0.0004), than in patients (n = 23) with an initial TLC above 1000 (mean rise of 40/μL) (p = 0.6105). TLC increased by at least 240/μL in half of the patients with severe lymphopenia and by more than 610/μL in 25% of patients.

**Conclusion**

Our data indicate that IV high-dose vitamin C treatment increases TLC, which strongly implies improvement of immune function, especially in patients with severe lymphopenia. Based on these observational findings, a randomised interventional study with more clinically relevant endpoints seems warranted.

## P140 Randomized controlled pilot study on the effectiveness and tolerance of mild whole body hyperthermia for patients with irritable bowel syndrome

### Christian Sachse^1^, Peter W Gündling^2^, Rainer Stange^1^

#### ^*1*^*Department of Natural Medicine, Immanuel Krankenhaus Berlin-Wannsee, Berlin, 14109, Germany;*^*2*^*Naturheilkunde und Komplementäre Medizin, Hochschule Fresenius, Idstein (Taunus), Germany*

##### **Correspondence:** Christian Sachse (c.sachse@immanuel.de)

**Background**

Mild Whole Body Hyperthermia is widely used for the treatment of muscular-skeletal disorders. Little is known about its use for internal diseases. Results of a case series seemed to justify a controlled trial for Irritable Bowel Syndrome***.***

**Methods**

Randomized controlled pilot study with waiting group design. Patients with moderate to severe Irritable Bowel Syndrome (Rom-III criteria) (aged between 18-65) were to be randomized into two groups. Six weekly water-filtered infrared irradiations (wIRA) as mild to moderate hyperthermia were to be applied. Main outcome parameter was the Irritable Bowel Syndrome—Severity Scoring System (IBS-SSS), secondary outcome a modified Nepaen Dyspepsia Index(NDI), both measured at baseline (V0), one week after the last therapy, resp. at week 6 of waiting (V1), and three months after V1 (V2). Patients of the waiting group were offered the same treatment after V1.

**Results**

41 patients were screened, 17 (16 f),(mean age 50,3y) randomized: 7 into treatment, 10 into waiting group. 6 of each group completed the trial per protocol. IBS-SSS sum-scores between V0 and V1 decreased significantly for treatment from 294 to 165 (p=0.031, Wilcoxon-Signed-Rank Test) but not for waiting (315 to 337, p=0.6). At V2, this effect was less pronounced. NDI showed similar courses. 4 patients (waiting group) discontinued treatment for an unrelated reason, 1 patient (treatment group) did not tolerate heat of hyperthermia.

**Conclusions**

Mild whole body hyperthermia may be an option for irritable bowel syndrome. However, conditions to secure long-term benefit have to be explored as well as possible mechanisms of action.

## P141 An evidence based review on integrative medicine in weight control

### Monirsadat Sahebkarkhorasani^1^, Hoda Azizi^2^

#### ^*1*^*Iranian traditional medicine, Mashhad Medical University, Mashhad, 9172314, Iran, Islamic Republic Of;*^*2*^*Department of Complementary Medicine, Mashhad University of Medical Sciences, Mashhad, 9135913556, Iran, Islamic Republic Of*

**Background**

Given the high prevalence of obesity in Iran and worldwide, its serious complications, difficult to treat and frequently refer of patients to Complementary Medicine approaches, awareness of the level of scientific evidence about the effectiveness of these therapies in obesity seems necessary.

**Objective**

The literature review about herbs, supplements and non-herbal complementary therapies for weight control and check the availability of those herbal medicine in Iran.

**Methods**

In this narrative review, complementary therapis for losing weight were searched via international databases including PubMed, SCOPUS and Scientific Iranian Database using obesity, overweight, herbal medicine, supplements, naturaceuticals, weight control, Integrative Medicine, Complementary Medicine and the names of herbs as keywords which resulted in 86 related articles. Among them, 79 were entered the review. The inclusion criteria were Randomized Controlled Trials, invitro and invivo studies, and reviews with acceptable level of evidence. Information regarding those herbs in herbal and Traditional Iranian Medicine references, their availability in Iran, and the available Pharmaceutical products in this country were investigated through English and Persian references. Also, the herbs proposed in Iranian traditional medicine for the treatment of obesity were listed.

**Results**

Among herbs and supplements used for weight control, blond psyllium, Alpha lipoic acid, conjugated linoleic acid, aloe, caffeine, calcium, bean pod and vitamin D have acceptable scientific evidence. They are safe also available in Iran. Agar has been produced as a food industry product and diacylglycerol for research purposes. Ephedra is not recommended due to unsafety. Among non-herbal complementary therapies, yoga and meditation show promising scientific evidence.

**Conclusion**

Regarding the evidence and availability of the mentioned safe herbs, supplements and integrative medicine approaches in Iran, they could be utilized as an adjunct to diet and physical activity also for research purposes

## P142 Characteristics of vegetarian and omnivorous yoga practitioners – a nationally representative survey of US adult yoga users

### Dania Schumann^1^, Romy Lauche^2^, Tobias Sundberg^2,3^, Matthew J Leach^2^, Holger Cramer^1,2^

#### ^*1*^*Department of Internal and Integrative Medicine, Kliniken Essen-Mitte, Faculty of Medicine, University of Duisburg-Essen, Essen, 45276, Germany;*^*2*^*Australian Research Centre in Complementary and Integrative Medicine (ARCCIM), Faculty of Health, University of Technology Sydney, Sydney, New South Wales, Australia;*^*3*^*Department of Neurobiology, Care Sciences and Society (NVS/OMV), Karolinska Institutet, Stockholm, Sweden*

##### **Correspondence:** Dania Schumann

**Purpose**

To examine the prevalence of vegetarianism among yoga users, as well as to explore potential differences and similarities between yoga users who chose a vegetarian diet and those who do not.

**Methods**

A secondary analysis of the 2012 National Health Interview Survey (NHIS) was performed. Twelve-month prevalence of vegetarian/vegan diet utilization, sociodemographic characteristics, practice patterns, reasons for and perceived benefits of using yoga were compared between vegetarian and non-vegetarian yoga users.

**Results**

A total of 42,366 households were eligible and 33402 (78.8%) adults responded to the questions on yoga use, with 2974 (8.9%) reporting to have used yoga within the past 12 months. A total of 1.7 million US American adult yoga users have used a vegetarian diet in the past 12 months (8.3%), compared to 2.7 million vegetarians among non-yoga users (1.3%). This equals 39.4% among vegetarians, compared to 8.6% among non-vegetarians who also practiced yoga. A high percentage indicated to have used yoga for general wellness or disease prevention (78.5–89.4%), to improve energy (65.5–83.7%) and athletic or sports performance (48.5–59.0%), with higher rates amongst vegetarian diet users. 31.0% of vegetarians had used yoga for a specific health problem compared to only 16.3% of non-vegetarians.

**Conclusions**

This analysis found that 39.4% of vegetarian diet users also have practiced yoga within the past 12 months, compared to 8.6% among non-vegetarian diet users. Despite a potential relationship of the use of a plant-based nutrition and yoga, more research is warranted on the actual synergetic use, its effects and its safety.

## P143 Evaluation of acupuncture-induced relief of hand pain in rheumatoid arthritis by a new device for pressure algometry

### Susana Seca^1,2^, Henry Greten^3^

#### ^*1*^*Chinese Medicine, Heidelberg School of Chinese Medicine, Heidelberg, Germany;*^*2*^*Faculty of Medicine, University of Coimbra, Coimbra, Portugal;*^*3*^*Traditional Chinese Medicine, Institute of Biomedical Sciences Abel Salazar, University of Porto, Porto, Portugal*

##### **Correspondence:** Susana Seca (susana.secca@gmail.com)

**Background**

Acupuncture is still controversially discussed, as methodologic problems associated to its research impair scientific progress. Besides effective allocation of acupoints and double blinding, quantification of its effects is still a problem that has to be addressed. In particular, for relieving pain and reducing inflammatory effects associated to Rheumatoid Arthritis (RA), the effects of acupuncture need to be quantified more clearly.

We aim to study the effects of acupuncture resorting to an algometry device specifically designed to assess the pressure tolerated by RA patients.

**Methods**

Fourteen RA patients with a Traditional Chinese Medicine (Heidelberg Model) diagnose of a so-called pivotal or *Turning Point Syndrome,* and meeting the criteria of the American College of Rheumatology, received three acupuncture treatments in one week with a standardized treatment in order to optimize potential therapeutic effects of acupuncture on pain.

A newly developed pressure algometry device, designed to assess the tolerance in hand pain in RA, was tested. We compared the outcome of clinical acupuncture, as measured by Pressure Algometry (PA) and Visual Analogue Scale (VAS).

**Results**

The patients tolerated higher pressure on the hands (p = 0.001) as well as improved VAS scores along with the treatment (p = 0.005). All 14 patients displayed improvements in PA, while 11 improved their VAS scores - out of the remaining, 2 patients had their VAS score unaltered and 1 (who also displayed the smallest improvement in PA) worsened slightly.

**Discussion**

The findings of this study suggest that acupuncture effects in pain relief may exist and can be measured by the PA Device. This opens the door to quantitative measurement of the improvement for the use in future double-blinded acupuncture studies on pain in RA patients.

## P144 Incense plants: the mystery behind the smoke

### Sugir Selliah

#### *Department of Systematic and Evolutionary Botany, University of Zurich, Zürich, 8008, Switzerland*

**Background**

Burning incense plants is an ancient practice in many cultures around the world. Incense is used for several purposes including in healing processes however, little is known about their local uses in Eurasia.

**Purpose**

This study is about understanding the biodiversity and ethnobotanical uses of incense plants across Eurasia.

**Methods**

An incense plants database was constructed by compiling data related to uses and botanical information from the existing scientific literature such as books and articles.

**Results**

Totally, 556 species (374 genera, 114 families) with 1033 use-reports have been recorded. The most commonly reported families are: Asteraceae, Fabaceae, Lamiaceae, Solanaceae, Apiaceae, Poaceae and Cupressaceae. Overall, medicinal and ritual uses are equally reported. The most used plant parts are the leaves, the stem and exudates, respectively. In medicinal and fumigant purposes, the administration of the smoke tend to be actively inhaled while in ritual use is passively inhaled. Moreover 56% of the total medicinal use (n = 451 use reports) is reported against respiratory system disorders, infections-infestations and pain.

**Conclusions**

The incense plants taxonomic distribution is similar to the one of medicinal plants with exception of Poaceae and Cupressaceae. Among the above-mentioned families, Solanaceae, Fabaceae and Poaceae revealed to be commonly used for medicinal purposes while Cupressaceae is typically used for ritual purposes. Our data suggest that the plants species are mainly used as mono-ingredient and a strong correlation between their means of administration and their use. This study supports the relevance of exploring the composition of incense plants smoke for bioactive compounds that potentially useful for treatments of specific disorders.

## P145 Use of complementary and alternative medicine among hypertension patients in Nepal

### Anu Shakya^1^, Dongwoon Han^1^, Ha Yun Kim^2^, Da I Choi^3^, Hyea B Im^3^, Soo J Choi^3^

#### ^*1*^*Global Health and Development, Hanyang University, College of Medicine, Seoul, 04763, South Korea;*^*2*^*Healthcare Administration, Hanyang University, Seoul, 133-791, South Korea*; ^*3*^*Global Health and Development, Hanyang University, Seoul, South-Korea*

##### **Correspondence:** Anu Shakya

**Background**

The use of complementary and alternative medicine is common in various ailments including hypertension since it is becoming a public health challenge. Positive outcomes can be achieved only through modification of life style and adoption of different modalities in order to control hypertension. However, there lacks evidences on behavioral risk factors on CAM use in Nepal. The purpose of the study was to determine the factors on the use of CAM among hypertension patients in Nepal.

**Methods**

A descriptive cross-sectional study was conducted using 246 purposively selected hypertension patients, who attended in free health camp conducted by *Chandranigahpur* Hospital from September to October. Information was gathered on the socio-demographic, clinical characteristics, risk behaviors, CAM use. Bivariate analysis by Chi-square test was used to make comparisons between CAM users and non-users. Multivariable analysis was performed by using logistic regression to identify independent predictor for CAM use where p-value equal to or less than 0.05 was considered as significant.

**Results**

Out of 242 participants, 94(38.8%) reported using CAM for management of hypertension; majority of them were using bitter gourd (71.2%), Ayurveda (68%), Neem (50%). There was significant difference among participants in income level, residence status, risk behaviors, SBP, DBP and BMI while comparing between CAM users and non-users.

**Conclusion**

Hypertension patients with behavioral risk factors and blood pressure above prehypertension level were using wide variety of CAM modalities concurrently with conventional medicines. Therefore, both patients and doctor should be educated about potential risks of herb drug interactions and adverse effects.

## P146 Combining plant extracts resulting in the elimination of undesired modes of action

### Anna Sherbakova^1,2^, Gudrun Ulrich-Merzenich^1^, Olaf Kelber^3^, Heba Abdel-Aziz^4^

#### ^*1*^*Medizinische Klinik III, UKB, Friedrich Wilhelms-Universität Bonn, Bonn, 53127, Germany;*^*2*^*Volga State Technical University, Department of Forestry, Yoshkar-Ola, Russian Federation;*^*3*^*Phytomeidines Supply and development Center, Steigerwald Arzneimittelwerk GmbH, Bayer Consumer Health, Darmstadt, 64295, Germany;*^*4*^*Bayer Consumer Health, Medicial Affairs, Phytomedicines Supply and Development Center, Steigerwald Arzneimittelwerk GmbH, Darmstadt, Germany*

##### **Correspondence:** Anna Sherbakova

**Background**

The rational of combining different plant extracts is often questioned. The common argument for combinations is the elimination of adverse effects by the chosen combination partners. We investigated the capability of STW 5, a combination of 9 different plant extracts (*Iberis amara (L.)*, *Menthae piperitae (L.) Chamomilla recutita (L.), Glycyrhiza glabra (L.), Angelica archangelica (L.), Carum Carvi (L.), Silybium marianum (L.) Gaertn. Melissa officinalis (L.)* and *Chelidonium majus (L.))* to modulate interleukin 8 (IL-8). Il-8 is a small heparin binding protein mediating the activation and migration of neutrophils from blood into tissues. We selected IL-8 since we showed earlier its major involvement in reflux esophagitis [1], an indication for STW 5.

**Methods**

Synchronized HET1A cells were treated (18 hrs), alone or in presence of the pro-inflammatory capsaicin (80 μM), with STW 5 or its single extracts (4 concentrations). The experimental conditions did not influence cell viability (resazurin assay).

**Results**

The spontaneous release of IL-8 was 46.3 ± 4.8 pg/ml. The highest release of IL-8 was induced by the *Iberis amara* extract *(IBE)* (441 ± 177 pg/ml), whereas STW5 lead to releases up to 49.3 ± 4.2 pg/ml. Other extracts like *Silybium marianum* reduced releases up to 20.1 ± 8 pg/ml. Capsaicin increased IL-8 release to 85.8 ± 14 pg/ml (p < 0.005). Under this condition *IBE* stimulated an IL-8 release up to 625 ± 121 pg/ml. STW 5 reduced IL-8 releases in the presence of capsaicin up to 33.7 ± 2 pg/ml. Thus Il-8 releases induced by the different plant extracts were apparently not simply additive.

**Conclusions**

The chosen plant combination lead to a downregulation of Il-8. Cytokines are organized in networks with a significant measure of redundancy. They are important signal transducing molecules in the intestinal immune system which support the maintenance of physiological functions. Studies how plant extracts influence cytokine networks will improve our ability to rationally compose effective and safe plant combinations.

**Acknowledgements**

A. Shcherbakova was supported by the German Academic Exchange Service (DAAD). The study received research funds from the Steigerwald Arzneimittelwerk GmbH.

**References**

1. Abdel-Aziz H, Schneider M , Neuhuber W, Meguid Kassem A, Khailah S, Jürgen Müller J, Gamaleldeen H, Khairy A, Khayyal FM, Thomas Efferth T, Ulrich-Merzenich G*.* GPR84 and TREM-1 signaling contribute to the pathogenesis of reflux esophagitis. Mol Med 2015, doi: 10.2119/molmed.2015.00098

## P147 Mindfulness instruction improves anger regulation in US urban male youth

### Erica Sibinga^1^, Lindsey Webb^2^, Jonathan Ellen^3^

#### ^*1*^*Pediatrics, Johns Hopkins School of Medicine, Baltimore, 21224, MD, United States;*^*2*^*Mental Health, Johns Hopkins School of Public Health, Baltimore, MD, United States;*^*3*^*Johns Hopkins School of Public Health, Baltimore, MD, United States*

**Background**

US urban male youth are exposed to significant and recurrent stress and trauma, related to community and interpersonal violence, failing schools, and lack of opportunities. Mindfulness can ameliorate the effects of stress and trauma in adult and urban youth populations. We aimed to test mindfulness for US urban male youth, especially related to anger and aggression.

**Methods**

This was a small randomized controlled trial of mindfulness instruction (MI) vs. health education (HE) implemented at a public middle school in Baltimore, Maryland, USA. The programs were 12 weekly 45-minute MI or HE classes during the school day, taught by well-trained, experienced instructors. De-identified data were analyzed using regression analysis in the aggregate by grade, which corresponded to group assignment.

**Results**

Data were provided by 61 predominantly African American male students (average 12.1 years) from the MI (n = 31) or HE (n = 30) programs and were comparable at baseline. Students described significant stressors, including family, loss, health, conflict, and school. After completion of the programs, the boys from the MI course had significant improvements in the following important outcomes: stress, sadness, anger, anger expressivity, aggression, paranoid thoughts, contempt, and mindfulness: acting with awareness (all p < 0.05).

**Conclusions**

This school-based MI program was feasible for male students in a US urban public school and led to improvements in psychological symptoms, emotion regulation, and mindfulness. These findings support the inclusion of high-quality MI programs in efforts aimed at reducing the negative effects of stress, toxic stress, and trauma.

## P148 Creating stress resilience with integrative health care interventions to IBD patients

### Kari Skrautvol, Dagfinn Nåden

#### *Faculty of Health Sciences, Oslo and Akershus University College of Applied Sciences, Oslo, 0130, Norway*

**Background**

Inflammatory Bowel Disease - IBD has become a global lifestyle disease in both developed and developing countries. The disease is increasing among young adults in Norway. The following research question is asked during in-depth interviews with patients: What is IBD patients’ experiences with integrative therapies?

**Methods**

A hermeneutic qualitative approach was used to analyze interviews with patients living with IBD outside hospital. Thirteen young adults between 18 to 45 years of age in calmer phases of IBD participated in the study.

**Results**

Three main themes emerged from the analysis of the interviews: (1) Understanding limits in embodied tolerance, (2) Restoring balance is creating a new equilibrium and (3) Creating resilience through integrative care. The use of integrative medicine was common among IBD patients attending outpatient clinics in Norway. Patients with IBD found it easier to communicate about integrative medicine with the IBD nurse and the dietitian rather than with the gastroenterologist. Dietary change was one important integrative treatment together with homeopathy, acupuncture and physical activity within their tolerance limits.

**Conclusions**

Anxiety, depression, stress, insomnia and fatigue are known consequences of IBD and create a lower degree of wellbeing for the patients. The digestive system is very important in establishing the interface between the body and the external world. Properly functioning digestion, psychosocial stress reduction and sleep quality are important to rebuild a balanced immune system. Stress resilience during a patients recovery from IBD requires self-understanding, self-recognition and psychosocial support from health care professions at hospital outpatient clinics. This research intends to contribute to the integrative health care curriculum in integrative medicine.

The Norwegian Regional Committee for medical and Health Research Ethics approved the study and a written consent was obtained from the participants.

## P149 Tai Chi’s impact on motor and non-motor outcomes in Parkinson disease: A systematic review and meta-analysis

### Rhayun Song^1^, Weronika Grabowska^2^, Kamila Osypiuk^2^, Gloria V Diaz^3^, Paolo Bonato^3^, Moonkyoung Park^4^, Jeffrey Hausdorff^5^, Michael Fox^6^, Lewis R Sudarsky^7^, Daniel Tarsy^6^, James Novakowski^2^, Eric A Macklin^8^, Peter M Wayne^2^

#### ^*1*^*Department of Nursing, Chungnam National University, Daejeon, 301747, South Korea;*^*2*^*Osher Center for Integrative Medicine, Harvard Medical School and Brigham and Women’s Hospital, Boston, MA 02215, United States;*^*3*^*Physical Medicine & Rehabilitation, Harvard Medical School, Boston, MA, United States;*^*4*^*Nursing, Woosong College, Daejeon, South Korea;*^*5*^*Sackler Faculty of Medicine, Tel Aviv University, Tel Aviv-Yafo, Israel;*^*6*^*Neurology, Harvard Medical School, Boston, MA, United States;*^*7*^*Neurology, Brigham and Women’s Hospital, Boston, MA, United States;*^*8*^*Massachusetts General Hospital, Boston, MA, United States*

##### **Correspondence:** Rhayun Song (songry@cnu.ac.kr)

**Purpose**

Using a meta-analytic approach, this study aimed to systematically evaluate and quantify the effects of Tai Chi on motor (UPDRS III, gait, balance, TUG, 6mw, and falls) and non-motor outcomes (quality of life, depression/mood) in patients with Parkinson’s disease (PD).

**Methods**

A PRISMA guided systematic search targeted randomized trials evaluating Tai Chi for individuals with PD published from January 1980 through August 2016. CMA V.3 was used to estimate effect sizes (Hedge”s g) and publication bias.

**Results**

Fifteen RCTs were included in quantitative synthesis. Comparison groups included no treatment or usual care group (n = 7, 47%) and exercise control group (n = 8, 53%). Duration of Tai Chi ranged from 2 to 6 months. Tai Chi was associated with significant improvement on all motor outcomes (UPDRS III [ES = 0.379, p < .001], balance [ES = 0.356, p < .001], 6mw [ES = 0.533, p < .001], TUG [ES = 0.341, p = .005], and falls [ES = .403, p = .004]) except maximum gait speed (ES = 0.128, p = .22) and both non-motor outcomes (quality of life [ES = 0.322, p = .007], depression/mood [ES = 0.436, p = .001]). Funnel plots suggested some degree of publication bias.

**Conclusion**

Evidence supports a potential benefit of Tai Chi for improving motor and non-motor outcomes for individuals with PD, but results must be interpreted cautiously due to methodological bias in many studies. Additional large rigorous trials are warranted to better characterize the effects of Tai Chi in PD and to guide selection of optimal doses and specific protocols for individuals with different constellations of symptoms.

## P150 A mixed-method feasibility study evaluating Tai Chi in stroke rehabilitation

### Rhayun Song^1^, Inok Hwang^2^, Sukhee Ahn^1^, Myung-Ah Lee^3^, Peter M Wayne^4^, Min K Sohn^2^

#### ^*1*^*Department of Nursing, Chungnam National University, Daejeon, 301747, South Korea;*^*2*^*Chungnam National University Hospital, Daejeon, South Korea;*^*3*^*Kinesiology, Recreation and Sport, Indiana State University, Terre Haute, IN, United States;*^*4*^*Harvard Medical School, Boston, MA 02215, United States*

##### **Correspondence:** Rhayun Song (songry@cnu.ac.kr)

**Purpose**

To employ a mixed method design including quantitative and qualitative assessments to examine the feasibility and effectiveness of a Tai Chi for stroke rehabilitation program.

**Methods**

A convenience sample of stroke survivors with hemiplegia was recruited for a Sun-style Tai Chi program adapted for stroke survivors, delivered twice weekly for 12 months. Physical function (strength, flexibility, balance, and mobility), self-efficacy, and activity of daily living were measured at baseline and at 3 month intervals for 12 months. Outcomes were analyzed by Friedman test followed by Wilcoxon test for posthoc comparisons. The experience of the participants was assessed by content analyses of in-depth interviews.

**Results**

Fourteen participants started the program; 10 patients with mean age of 68.5 years completed all 5 measurements with a class attendance rate of 72.12% and loss to follow-up of 28.5%. The participants significantly improved self-efficacy at 3 months (Χ^2^ = 21.84, *p* < .001), and both flexibility (Χ^2^ = 11.70, *p* = .020) and balance (Χ^2^ = 14.08, *p* = .007) at 6 months. No serious adverse events were reported. Qualitative results highlight the programs positive impact on self awareness of physical improvement, psychological wellbeing, social support, and improved confidence in performing daily activities.

**Conclusion**

A 12-month adapted Sun-style Tai Chi program was safe and feasible in community dwelling stroke survivors. Trends of improved physical function and self-efficacy support the value of clinical trial evaluating the effectiveness of this program in post-stroke rehabilitation.

## P151 Heart rate variability – next step in the development of pulse analysis in traditional medicine and naturopathy

### Oleg Sorokin

#### *National Ayurvedic Medical Association of Russia, Novosibirsk, 630108, Russian Federation*

We have developed and validated an algorithm for constitution evaluation in traditional medicines and Naturopathy, based on the algorithms of VedaPulse technology (Biokvant, Russia, www.vedapulse.com). Analysis of spectral values of heart rate variability allows a practicing specialist to objectively evaluate nine basic constitution types with further creation of personalized rehabilitation program for the patient. VedaPulse technology algorithms allow implementing common standards of evaluation of functional health state that can be accepted by western insurance medicine as part of naturopathic doctor’s practice.

1) Analysis of cardiointervalogram in typical cases allows drawing a parallel between constitutional types of the pulse and dynamics of heart rate variability. This trend manifests itself through the characteristics of spectral analysis of cardiointervalogram. The main diapason of regulating factors for Wind type of constitution falls into the very low frequency (VLF) area. In Fire type of constitution low frequency (LF) factors dominate. In Phlegm type of constitution – high frequency (HF) factors.

2) An opportunity to evaluate absolute values of the spectrum power allows quantitatively evaluating the current physiological constitution of the patient in terms of traditional medicine and Naturopathy, allowing a naturopathic doctor to determine necessary rehabilitation procedures based on individual (constitutional) characteristics of the body.

## P152 Fasting induces global changes in gene expression in patients with metabolic syndrome

### Nico Steckhan^1^, Dagmar Heydeck^2^, Astrid Borchert^2^, Christoph-Daniel Hohmann^1^, Harmut Kühn^2^, Andreas Michalsen^1^, Christian Kessler^1^

#### ^*1*^*Clinical Naturopathy, Charité Institute of social medicine, epidemiologiy and health economics, Berlin, 14109, Germany;*^*2*^*Charité Institute of Biochemistry, Berlin, 10117, Germany*

**Question**

Latest evidence has shown potential health benefits of different kinds of fasting. Data from model organisms have shown that caloric restriction can increase longevity and reduce incidence of many chronic diseases, the evidence from human studies is much more limited and equivocal. Fasting has system wide effects that contribute to the health benefits observed in animals, the precise molecular mechanisms responsible remain to be elucidated.

**Methods**

This is to our knowledge the first study that examined the effects of a five days fast on gene transcription profiles in white blood cells from subjects suffering from metabolic syndrome.

**Results**

Principal component analysis revealed strong separation between samples before and after fasting. We found over 5000 differential expressed genes. Gene ontology analysis revealed genes associated with different biological processes like apoptosis, decreased expression of pro- inflammatory genes and induction of autophagy.

**Conclusion**

These processes acting synergistically and indicate potential mechanisms that could contribute to the putative beneficial effects of fasting in humans suffering from metabolic syndrome.

## P153 Effects of a mind-body intervention on hair cortisol from patients with cardio-metabolic risk

### Nico Steckhan, Christoph-Daniel Hohmann, Holger Cramer, Andreas Michalsen, Gustav Dobos, Christel von Scheidt, Clemens Kirschbaum, Tobias Stalder

#### *Institute of Social medicine, epidemiologiy and health economics, Charité University Hospital, Berlin, 14109, Germany*

##### **Correspondence:** Nico Steckhan

**Background**

The demand for multimodal interventions for chronic noncommunicable diseases is emerging. Latest evidence has shown tremendous involvement of lifestyle on risk factors leading to metabolic syndrome.

**Methods**

For a six month clinical trial we recruited 142 subjects with cardio-metabolic syndrome. The most essential intervention was a 12 weeks mind-body therapy as day care clinic. We conducted this study to examine the biological and biopsychological effects of such a multimodal intervention.

**Results**

For the first time this study could show decreased hair steroids, e.g cortisol (-1.85 ± 7.55 pg/mg, F(2, 142) = 4.3 p = .03) after six month follow up. We also found positive correlations between Cohens Perceived Stress Scale as well as Profile of mood scales and hair steroids.

**Conclusion**

Together these effects underline the importance of multimodal interventions forpatients with metabolic syndrome. Lifestyle modifications seem to be an effective strategy regarding sustainable health benefits.

## P154 Intentional touch for elderly people with chronic pain – a qualitative study

### Barbara Stöckigt, Michael Teut, Ralf Suhr, Daniela Sulmann, Benno Brinkhaus

#### *Institute for Social Medicine, Epidemiology and Health Economics, Charité - Universitätsmedizin Berlin, Berlin, 10117, Germany*

##### **Correspondence:** Barbara Stöckigt (barbara.stoeckigt@charite.de)

**Purpose**

Physical touch is a basic part of medicine and clinical care. Intentional Touch (IT) refers to a soft physical touch with the aim to improve complaints and increase wellbeing. The aim of this qualitative study was to investigate the subjective perception and experiences of IT of nurses and patients with chronic pain.

**Methods**

The intervention was developed by a stakeholder-involvement including nurses, patients, experts in therapies using physical touch and scientists. Nurses of a nursing home for elderly people were trained on IT by three experts in physical touch therapies, complementary and integrative medicine (CIM) and qualitative research. Semi-structured interviews (week 1 and 4 of the intervention) and participatory observation with video recording were conducted with nurses applying and inhabitants receiving IT. All interviews were analysed using Qualitative Content Analysis, video recordings were analysed using Qualitative Visual Analysis.

**Results**

Six nurses and six patients were included in the study. During the treatment relaxation, well-being and the sensation of warmth were described by all participants. Patients said to feel no pain during the treatment and pain relief afterwards. Patients described better motivation, positive feelings, improved self-confidence and self-effectiveness after the treatment whereas nurses described a feeling of empowerment and revaluation of their nursing work. An empathetic attention and an attitude of openness for the relationship between nurses and patients was said to be important. However, limited time resources including problems to finding time, continuity and reliability were challenges for IT integration in daily work.

**Conclusion**

Intentional Touch might be helpful to increase relaxation, well-being and pain relief for the elderly people suffering from chronic pain and to improve the patient-nurse relationships. IT needs more research to define its value in medical and clinical care.

## P155 In major depressive disorder, yoga and coherent breathing reduced depressive symptoms, increased respiratory sinus arrhythmia, and increased gama-aminobutyric acid

### Chris Streeter^1,2^, Patrica Gerbarg^3^, Marisa Silveri^1,2^, Richard Brown^4^, John Jensen^2^

#### ^*1*^*Psychiatry, Boston University School of Mediicine, Boston, 02118, MA, United States;*^*2*^*Psychiatry, Harvard University, Belmont, CA, United States;*^*3*^*Psychiatry, New York Medical College, Valhalla, 10955, NY, United States;*^*4*^*Psychiatry, Columbia College of Surgeon, New York, NY, United States*

##### **Correspondence:** Chris Streeter

**Purpose**

To test The Vagal-GABA Theory in subjects with Major Depressive Disorder (MDD), by measuring the effects of a yoga plus coherent breathing intervention on vagal activity, thalamic gamma-aminobutyric acid (GABA)/Creatine ratios (GABA/Cr), and depressive symptoms.

**Methods**

Subjects with MDD (n = 16) participated in a 12-week intervention, including 90-minutes of Iyengar yoga plus coherent breathing (at 5 breaths per minute) 2–3 times per week and a 20-minute daily home practice. Baseline measures were: Beck Depression Inventory II (BDI-II); respiratory sinus arrhythmia (RSA) as an indicator of vagal activity; and thalamic GABA/Cr using magnetic resonance spectroscopy (MRS). RSA and GABA levels were also obtained at the end of week 12 pre- and post- a 90-minute yoga plus coherent breathing intervention.

**Results**

From baseline to week 12, BDI-II scores decreased from 27.31 ± 8.45 to 8.88 ± 7.70 (F(1,15) = 91.47, *p* <0.001). After 12 weeks, acute increases in recovery RSA (post: 6.71 ± 0.61 – pre: 8.06 ± 0.84) correlated with acute (post: 0.31 ± 0.07 – pre: 0.26 ± 0.07) thalamic GABA/Cr (Spearman”s *rho*(7) = 0.821, *p* = 0.023). Decreased BDI-II scores correlated with the total number of minutes of yoga plus CB per week.

**Conclusions**

This study supports the Vagal-GABA Theory by showing that in patients with MDD, a 12-week yoga plus coherent breathing intervention was associated with significant reduction in depressive symptoms (BDI-II), increased vagal activity (RSA), and increased thalamic GABA levels (MRS). A randomized controlled trial is underway to further validate these findings.

## P156 Establishing integrative care for children with cancer – challenges in evaluation

### Wiebke Stritter^1^, Britta Rutert^1,2^, Angelika Eggert^1^, Alfred Längler^3^, Georg Seifert^1^, Christine Holmberg^2^

#### ^*1*^*Department of Pediatrics, Division of Oncology and Hematology, Charité Universitätsmedizin Berlin, Berlin, 13353, Germany;*^*2*^*Institute of Public Health, Charité Universitätsmedizin Berlin, Berlin, Germany;*^*3*^*Institute for Integrative Medicine, Gemeinschaftskrankenhaus Herdecke, Witten/Herdecke University, Witten/Herdecke, Germany*

##### **Correspondence:** Wiebke Stritter

**Background**

Treatment in pediatric oncology is associated with multiple side effects, such as nausea, pain and sleeping problems. To ease the associated suffering external applications as used in anthroposophic care have shown to be useful. A holistic concept of care can open up new therapeutic resources for the nurses, which may lead to more self-efficiency and satisfactory work for the nurses. Furthermore, the involvement of parents in the treatment can be beneficial in helping them to cope with the life-threatening situation of their child.

Goal of a project at a German university hospital is to implement anthroposophic care into standard care in pediatric oncology by developing, implementing and evaluating an integrative care concept.

Leading question of this contribution is how an evaluation concept ought to be designed to master the complexity of implementing an integrative care concept and how mixed method data can be merged to a coherent and comprehensive evaluations concept.

**Methods**

Following the framework of the UK Medical Research Council on process evaluation, concept development runs through multiple phases. Different methods, such as participant observation, qualitative interviews, psychometric measurements and analysis of care documentation and hospital billing were chosen to equally reflect on the different aspects of the intervention under evaluation.

**Results**

A complex evaluation concept was developed parallel to the integrative care concept, addressing the following levels:

- Process evaluation of implementation
- Evaluation of the clinical outcomes and effects of the interventions
- Evaluation of satisfaction and change in psychosocial stress in patients, parents and nurses
- Evaluation of cost effectiveness.

**Conclusion**

First results show that evaluation starts much earlier than implementation itself. In addition, contextual factors such as shortage of nursing staff, realities of health care system and politics are to be considered in concept development.

## P157 Chinese herbal medicine for patients living with HIV in Guangxi province, China: registered database analysis

### Jin Sun^1^, Xin Deng^2^, Wen-Yuan Li^1^, Bin Wen^2^, Nicola Robinson^3^, Jian-Ping Liu^1^

#### ^*1*^*Center for Evidence-Based Chinese Medicine, Beijing University of Chinese Medicine, Beijing, 100029, China;*^*2*^*Guangxi university of Chinese medicine affiliated hospital, Guangxi, China;*^*3*^*Faculty of Health and Social Care, London South Bank University, London, United Kingdom*

##### **Correspondence:** Jin Sun

**Background**

Chinese herbal medicine (CHM) has been used for treating people living with HIV in China for over twenty years. The National Free Traditional Chinese Medicine HIV/AIDS Treatment Program (NFCHMP) provided free CHM for HIV/AIDS patients from 2004. We aimed to explore the effectiveness of CHM for HIV patients by analyzing registered database established by NFCHMP in Guangxi province.

**Methods**

Data from patients living with HIV was extracted from registered databases in Guangxi province, China. Patients were divided into 3 cohorts according to their treatment regimens which included CHM alone, CHM combined highly active antiviral retroviral therapy (HAART) and HAART alone. Characteristics and clinical outcomes were described and compared among the 3 cohorts.

**Results**

2963 patients recorded in two registry were analyzed, 1522 individuals used CHM, 1061 individuals used CHM combined HAART, 380 individuals used HAART. Mean age was 48.1 ± 14.3 years, and 63.2% were male. CHM users had significant higher baseline CD4 cell counts (368.8 ± 211.6 cell/μL), approximately 100 cell/μL higher than the patients with CHM combined HAART regimens and HAART regimens. The three regimens improved patients” CD4 cell counts after treatment. All three regimens demonstrated improvements in patients” CD4 cell counts. Compared to the initial sharp improvement in the HAART group, the CD4 cell counts of patients in CHM group and CHM combined HAART group rose steadily and smoothly.

**Conclusion**

CHM alone appears to improve CD4 cell counts of patients. It is noteworthy that compared to patients who only took HAART regimen, the patients took CHM were more likely to have a steady growth rise in CD4 cell counts.

Key words: HIV, AIDS, Chinese herbal medicine, HAART, database analysis

## P158 The analysis of a satisfaction survey on the Korean Medicine Doctors’ Visiting School Program(KMDVSP) - Cohort study: Middle & High school participator of Seong-nam

### Hyun K Sung^1^, Narae Yang^1^, Ho Y Go ^2^, Seon M Shin^3^, Hee Jung^4^, Young J Kim^2^, Woo S Jung^5^, Tae Y Park^1^

#### ^*1*^*Institute for Integrative Medicine, Catholic Kwandong University International St. Mary’s Hospital, Incheon, South Korea;*^*2*^*Internal medicine, Semyung Univ, Chungju, South Korea;*^*3*^*Internal medicine, Semyung Univ, Jecheon, South Korea;*^*4*^*Korean medicine, Korea Health Industry Development Institute, Osong, South Korea;*^*5*^*Internal Medicine, Kyunghee Univ, Seoul, South Korea*

##### **Correspondence:** Hyun K Sung

**Question**

This study aimed to build base-line data for school health care satisfaction questionnaires on the Korean Medicine Doctors’ Visiting School Program(KMDVSP).

**Methods**

Korean medical doctors were assigned to 20 middle and high schools located in Seong-nam. Each school was visited by a doctor eight times over a five-month to conduct three programs : health consultations, Korean medical treatments and health education classes.

**Results**

85 teachers and school administrators responded to the survey, and the following conclusions were drawn about the program: Overall satisfaction of the program (very satisfied 69%, satisfied 28%, adequate 2%), the three programs : health counseling (very satisfied 69%, satisfied24%, adequate 2%), treatment of the Korean medicine doctor (very satisfied 69%, satisfied25%, adequate 2%), health education class (very satisfied 58%, satisfied31%, adequate 2%, very unsatisfied 1%).

Responses to questions regarding the advantages of the KMDVSP included: Provide students with useful information about their health’ (65%), Tell students what the usual health condition was (62%), Tell students how to manage their health and cure them (61%), Treat a sick student immediately (52%). As to the final question of whether the DVSP should continue, 93% answered Yes.

**Conclusion**

The proper health management of students is an important part of the foundation of the national health policy. We had developed the pilot project and completed it with success. Responses from teachers were overwhelmingly positive. Further studies based on this trial are needed for further development of project models and extended enforcement of the program.

## P159 Preliminary international survey on the image of integrative medicine

### Kiyoshi Suzuki^1^, Toshinori Ito^2^, Seiya Uchida^3^, Seika Kamohara^4^, Naoya Ono^4^

#### ^*1*^*MOA Health Science Foundation, President, Tokyo, Japan;*^*2*^*Osaka University School of Medicine, Integrative medicine, Osaka, Japan;*^*3*^*MOA Health Science Foundation, Tokyo, Japan;*^*4*^*Japan Society of Integrative Medicine, Tokyo, Japan*

##### **Correspondence:** Kiyoshi Suzuki

**Background**

Integrative medicine (IM) is commonly understood as the combination of Western medicine (WM) and other treatment methods (CAM). However its image may differ across countries. In Japan, IM is discussed as encompassing a medical and a social model. Medical model aims to enhance the effect of treatment and QOL. Social model aims to enhance QOL through community development. These models complement each other to create healthy aging society and optimize healthcare costs.

**Method**

In 2015, Japan Society of IM conducted a survey on the image of IM, and whether people agree towards the aforementioned concept in 14 different countries; 643 individuals (Japan 33%, South America 22%, Asia 20%, Europe 15%, USA 10%; medical personnel 60%, politicians 12%) answered the questionnaire.

**Results**

Seventy-three percent of total participants understood what IM is, 56% perceived IM to be understood as a combination of WM and CAM within their respective countries, 78% agreed towards the aforementioned concept, and 75% fully agreed towards the promotion of IM. Forty-five percent in Asia, 50% in Japan, 59% in Europe, 63% in South America, and 75% in US understood IM as the combination of WM and CAM. However, 72% in Europe, 74% in US, 77% in South America, 80% in Asia, and 82% in Japan agreed towards the aforementioned concept; 62% in Asia, 73% in Europe, 76% in Japan, 77% in US, and 82% in South America agreed towards the promotion of IM.

**Conclusion**

Further research is necessary to clarify the concept and goal of IM.

## P160 Studying relations between acupuncture source (Yuan) points reaction and subjective conditions of chronic pain patients at Kampo medicine outpatient clinic by multiple logistic regression analysis

### Mitsuyuki Takamura^1^, Ayumu Yokochi^2^, Kazuo Maruyama^1,2^

#### ^*1*^*Kampo medicine outpatient clinic, Mie University Hospital, Tsu, 24201, Japan;*^*2*^*Pain Clinic, Mie University Hospital, Tsu, 24201, Japan*

##### **Correspondence:** Mitsuyuki Takamura

**Purpose**

In an acupuncture theory, Source (Yuan) points are the points where the “Source Qi” may be accessed and these are indicated for diseases related to the five viscera. Also in Kampo medicine, the Japanese traditional herbal medicine, physicians must know the condition of the five organs of patients to treat them. Especially for controlling chronic pain, management for Qi status is very important. However, few Kampo physicians examine acupoints. We investigate relations between Source points and general conditions of chronic pain patients by statistical study as comprehending their status.

**Methods**

Retrospectively we checked the medical records of chronic pain patients who had visited our department from September 2013 to September 2016. We made a list including presence or absence of common 17 subjective conditions and of tenderness in five Source points (LU9, HT7, KI3, LR3, SP3). Relations between subjective conditions and acupoints reaction were calculated by chi-square test and multiple logistic regression analysis (MLRA).

**Results**

A total of 55 patients were listed. Average age was 54.6 ± 17.1, male to female ratio was 23:32. Most patients (67.3%) had tenderness in LR3. By MLRA, “heat sensation” was most associated with LR3 tenderness (Odds Ratio = 11.59), “sleep disturbance” with KI3 (OR = 8.11), “thirst” with HT7 (OR = 8.00), and “tinnitus” and “lump in throat” with SP3 (OR = 7.75, 5.57, respectively).

**Conclusions**

More than half of patients with chronic pain had tenderness on LR3. Because it was most associated with that tenderness, it might be important to care for Liver system by Kampo if the patients complained “heat sensation”.

## P161 Sham-controlled randomized clinical trial on the effectiveness of co-assisting acupuncture in the treatment of mild and moderate depression in a primary health care (study to begin in September 2016)

### Patricio Tapia

#### *Department of Family Medicine, School of Medicine, Pontifica Cniversidad Católica de Chile, Santiago, 8330077, Chile*

**Purpose**

To determine if co-assisting acupuncture helps to reduce symptoms in the standard treatment of mild and moderate depression in a primary care urban center in Chile.

**Methods**

A single-blind study will be conducted with 18 to 65 years old patients diagnosed with mild and moderate depression, assigned randomly in two groups. One group will be treated with true acupuncture. The control group will receive sham (false) acupuncture. Both groups will receive 10 sessions of 30 min, mostly once a week or more often. The points used in each session will be the same: Du20; Ex-HN3; H7; and Sp6 (the last two on both sides of the body). The control group will receive the sham treatment, with the needles inserted shallowly, avoiding *de-qi*.Expected

**Results**

The severity of depressive symptoms, as measured by the Beck Depression Inventory (50% reduction of the baseline score), and a remission of symptoms less than or equal to the 10 score, at the end of the 10 treatment sessions, and 3 months after completing the treatment. The results should indicate an improvement in the application of a Health Questionaire (EQ-5D).

**Conclusions**

At the end of this study, a significant effect of co-assisting acupuncture will be evident, as an Integrative Medicine Therapy for patients diagnosed with mild and moderate depression at the Family Health Center Juan Pablo II, La Pintana, in Santiago, Chile, with a 3 month lasting effect.

## P162 Understanding decision making in Complementary and Integrative Medicine – results of a qualitative study with patients in a university outpatient clinic

### Katarzyna Thabaut, Benno Brinkhaus, Barbara Stöckigt

#### *Institute for Social Medicine, Epidemiology and Health Economics, Charité - Universitätsmedizin Berlin, Berlin, 10117, Germany*

##### **Correspondence:** Katarzyna Thabaut (katarzyna.thabaut@charite.de)

**Purpose**

Little is known about how and whether Complementary and Integrative Medicine (CIM) works on the adherence. The aim of this qualitative research project was to examine the social reality of patients, their views, attitudes and decisions during CIM medical treatment including naturopathic.

**Methods**

Semi-structured interviews were conducted in a University outpatient Clinic specialised in CIM with eight patients suffering from various chronic diseases. The interviews were digitally recorded, transcribed and analysed using content analysis according to Mayring (2010) and the software program MAXQDA®.

**Results**

The results of the interviews revealed the importance of the role of physicians in the context of the implementation of CIM therapies. Essential aspects for the patients in the decision making process during the treatment were the involvement in the treatment process, the patient-physician communication, the provision of relevant information about the treatment and therapy as well as education of the patient on self-management strategies. In addition biographical background seemed to influence the patients decisions resulting in individual attitudes, motivations and expectations. In the course of the implementation of CIM therapies structural and organizational factors, like the time management during the medical treatment, seemed to be relevant. Furthermore therapy-related factors, such as the practicability seemed to challenge patients during the implementation of CIM therapies in their daily routine.

**Conclusions**

Decisions about the implementation of CIM therapies are influenced by biographical history, motivation and various decision-making processes. The communication between physicians and patients and involvement in the treatment process play also a central role.

## P163 Health related quality of life of breast cancer patients receiving non-pharmacological interventions within a multimodal integrative concept of a certified breast cancer centre

### Anja Thronicke^1^, Matthias Kröz^1,2,3^, Megan Steele^1^, Harald Matthes^1,2^, Cornelia Herbstreit^2^, Friedemann Schad^1,2^

#### ^*1*^*Research Institute Havelhöhe, Berlin, 14089, Germany;*^*2*^*Hospital Havelhöhe, Berlin, Germany;*^*3*^*Institute for Social Medicine, Epidemiology and Health Economics, Charité University Hospital, Berlin, Germany*

##### **Correspondence:** Anja Thronicke

**Background**

Health-related quality of life (HRQL) has become increasingly important in the comprehensive treatment of breast cancer. This pivotal study explored HRQL in breast cancer patients who received non-pharmacological interventions (NPI) within a multimodal integrative concept of a certified German breast cancer centre (BCC).

**Methods**

During a six-month’s observation baseline period, data of 48 breast cancer patients were analysed that consented to this study and received NPI and guideline-orientated standard care. Patient’s self-reported health and HRQL status were collected using the EORTC QLQ-C30 questionnaire at first diagnosis, 6 and 12 months follow-up. Clinical data were retrieved from the Network Oncology registry.

**Results**

Complete self-reported HRQL and health-related data on all measured time points were collected for 100% of patients. Median age at diagnosis was 59.6 years; all patients were female. All patients received psycho-oncological support and nursing interventions, 97% received movement therapies, 88% art therapies and 81% massages. Patient’s self-reported health status (p = 0.02) and HRQL (p = 0.03) improved significantly over 12 months. Daily living was reported to be limited to a greater extent by psychosocial conditions and fatigue than physical and cognitive conditions (p < 0.0001). After 12 months patients reported improvement compared to six months follow-up, most significantly in emotional (p = 0,0001) and social fields (p = 0.02) that may be explained by full psycho-oncological and nursing support at the BCC.

**Conclusions**

Patient health status and HRQL significantly improved within 12 months after first diagnosis of breast cancer. Our results give a first picture of HRQL of breast cancer patients treated with NPI in a certified BCC.

## P164 Evaluation of Chinese Herbal Medicine Jinlida in Type 2 Diabetes patients based on stratification: results of subgroup analysis from 12-week trial

### Jiaxing Tian^1^, Fengmei Lian^1^, Libo Yang^2^, Xiaolin Tong^1^

#### ^*1*^*Guang’anmen Hospital, China Academy of Chinese Medical Sciences, Beijing, 100053, China;*^*2*^*Hebei Medical University Pharmaceutical Research Institute, Shijiazhuang, 050017, China*

**Background**

Previous study found that Chinese Herbal Medicine Jinlida could significantly enhance the hypoglycemic action of metformin compared to metformin alone. However, the advantage group of Jinlida has not been clarified yet. We aimed to compare the efficacy of Jinlida in patients with T2DM based on stratification in this study.

**Methods**

Data were analyzed from 12-week, randomized, double-blind, multicenter study of Jinlida. The efficacy evaluation included HbA1c, FPG and 2hPG levels stratified by baseline HbA1c, sex, age, BMI and duration of DM. HOMA-IR and HOMA-β were also evaluated stratified by baseline insulin.

**Results**

192 adult type 2 diabetes patients whose diabetes was poorly managed with metformin monotherapy were enrolled. Subjects were randomly allocated to receive either Jinlida (9 g) or the placebo TID for 12 consecutive weeks. After 12 weeks of treatment, HbA1c was significantly reduced (p < 0.05) compared with baseline and placebo in patients taking Jinlida. Greater reductions were observed in patients with baseline HbA1c >8.5%, male, age > 60 yr, BMI ≤24 kg/m2 or duration of DM >5 yr (p < 0.05). Jinlida significantly alleviated insulin resistance (p < 0.05) in patients with baseline insulin >20 mU/L and improved β-cell function (p < 0.05) compared with baseline and placebo in patients with baseline insulin ≤ 20 mU/L (p < 0.05).

**Conclusions**

Jinlida significantly improved glycemic control in a wide range of T2DM patients ineffectively managed by metformin monotherapy, with greater improvements in patients with poor glycemic control, male, elderly, normal weight or long disease course. However, the results above need to be further confirmed by evidence-based medicine.

## P165 Effect of Lignum Sappan compatibility with Radix Aconiti Lateralis Praeparata on growth and metastasis of Lewis lung cancer

### Tian Tian, Hewei Zhang

#### *Beijing University of Chinese medicine, The Preclinical medicine Institute, Beijing, China*

##### **Correspondence:** Tian Tian

**Objective**

To investigate the effect of Lignum Sappan compatibility with Radix Aconiti Lateralis Praeparata on growth and metastasis of Lewis lung cancer(LLC) and detect p-selectin expressed in mice serum.

**Methods**

Lung carcinoma model was established by subcutaneously inoculating Lewis lung carcinoma cells in C57BL/6 mice.Randomly divided into four groups:control group (n = 13), low dose group (n = 13), mid-dosage group (n = 13) and high-dosage group (n = 13), continuously gavage for 21 days, once daily. Inhibitory rate, metastasis rate and inhibition rate of metastasis were observed.The differential expression p-selectin were detected between the treatment group and the control group.

**Results**

A statistical difference was found in tumor weight between treatment groups and control grou (p < 0.05).The results reveal that Lignum Sappan compatibility with Radix Aconiti Lateralis Praeparata in intermediate dose and low-dose groups can inhibit the expression of sP-selectin (p < 0.05).

**Conclusions**

Lignum Sappan compatibility with Radix Aconiti Lateralis Praeparata can inhibit the growth and metastasis of Lewis lung cancer (LLC). The mechanism may be related to inhibiting the expression of sP-selectin.

## P166 Different clinical therapeutic effects due to different practicing: a randomized crossover acupuncture trial

### Xia Tian^1^, CongCong Wang^1^, Qian Yun Chai^1^, Lijuan Zhang^2^, Ruyu Xia^1^, Na Huang^1^, Yutong Fei^1^, Jianpin Liu^1^

#### ^*1*^*Beijing University of Chinese Medicine, Beijing, 100029, China;*^*2*^*Acupuncture, Xiyuan hospital CACMS, Beijing , China*

##### **Correspondence:** Xia Tian

**Background**

Bias could be introduced to a trial investigating non-pharmaceutical intervention, such as acupuncture, if the real-world practicing is different among the recruited practitioners.

**Objective**

To evaluate the impact of different needle manipulations of two acupuncturists on effectiveness of acupuncture for chemotherapy induced nausea and vomiting (CINV) when following the same needling regimen.

**Methods**

A randomized cross-over trial. Two acupuncturists (senior-A, junior-B) provided acupuncture treatments 5 times per cycle to CINV cancer patients. Every patient was treated by A (or B) first and B (or A) second in two adjacent chemotherapy cycles. Acupuncture regimens were all decided by senior. The primary outcome was nausea and vomiting symptoms evaluated by National Cancer Institute nausea and vomiting rating scale (NCI) on the third day of chemotherapy. Rhodes Scale was also adopted. Other prognostic factors, including patient-doctor communication, acupuncture expectancy and treatment satisfaction was recorded.*Paired-Sample T test* and *Wilcoxon test* were used.

**Results**

39 patients were randomized (A, 20; B, 19) and analyzed.Symptoms of nausea and vomiting aggravated gradually and reached peak on day 3 during chemotherapy. NCI scores showed a significant difference between groups in vomiting (Z = −0.264, P = 0.017). Rhode scores (both nausea and vomiting domain) of patients treated by senior were significantly lower than those treated by junior. There was no significant difference on the other days. There was no significant difference for other prognostic factors.

**Conclusions and implications**

When possible prognostic factors were controlled to the possibly largest extent, senior acupuncturist controlled vomiting in CINV patients better than junior acupuncturist on the third day of chemotherapy when symptoms were most severe. This could be largely attributed to the needle manipulation differences between acupuncturists. Needling skills should be sufficiently considered in the phase of designing the trial, analyzing data and reporting of results.

## P167 Immediate and sustained improvements in psychological health and well-being following a 5-day residential yoga-based program for frontline professionals

### Natalie Trent^1,2^, Mindy Miraglia^3^, Jeffrey Dusek^4^, Edi Pasalis^3^, Sat B Khalsa^1,2^

#### ^*1*^*Brigham and Women’s Hospital, Medicine, Boston, MA, United States;*^*2*^*Harvard Medical School, Boston, MA, United States;*^*3*^*Kripalu Center for Yoga & Health, Stockbridge, MA, United States;*^*4*^*Allina Health, Minneapolis, MN, United States*

##### **Correspondence:** Natalie Trent

**Objective**

The purpose of this single-arm effectiveness study was to examine the impact of a pre-existing residential yoga-based program on measures of psychological well-being and health behaviors.

**Methods**

We evaluated the 5-day Kripalu RISE (resilience, integration, self-awareness, engagement) program conducted at the Kripalu Center for Yoga & Health, a non-profit educational organization in western Massachusetts focused on yoga, health, and holistic living that sees over 50,000 participants per year in its facility’s programs. The RISE program includes approximately 5 hours per day of yoga classes, meditation training, lectures, and didactic/experiential activities to promote physical and psychological health. Forty adult frontline professionals participated in the program and completed questionnaires immediately before (pre-RISE), after (post-RISE), and two months following the program (follow-up). Self-report measures included the Perceived Stress Scale, Positive and Negative Affect Schedule, Subjective Vitality Scale, Psychological Empowerment Scale, Resilience Scale, Self-Compassion Scale, and the Five Facet Mindfulness Questionnaire. Health behaviors including amount of exercise, vegetable and fruit intake, and sleep quality were assessed with the Lifestyle Questionnaire.

**Results**

Paired samples t-tests revealed that empowerment, mindfulness, resilience, stress, affect, and vitality improved from baseline to post-program (all p < .05) and that these improvements were sustained at the two month follow-up (all p < .05). Further, a significant increase in self-compassion was observed at the 2 month follow-up compared to post-program (p < .05). Participants also reported significant increases in daily exercise, fruit and vegetable consumption, and sleep quality, all of which persisted at the two-month follow-up (all p < .05) with the exception of exercise.

**Conclusion**

These findings suggest that the RISE program improves many measures of psychological well-being and health behaviors, and that these improvements persist two months after the program.

## P168 Acupuncture for osteoarthritis of the knee: a systematic overview

### Milena Trifunovic-König ^1,2^, Petra Klose^2^, Holger Cramer^2,3^ , Romy Lauche^2,3^ , Anna Koch^1,2^ , Gustav Dobos^2^, Jost Langhorst^1,2^

#### ^*1*^*Department of Internal and Integrative Medicine, Centre of Integrative Gastroenterology, Kliniken Essen-Mitte, Faculty of Medicine, University of Duisburg-Essen, Essen, Germany;*^*2*^*Department of Internal and Integrative Medicine, Kliniken Essen-Mitte, Faculty of Medicine, University of Duisburg-Essen, Essen, Germany;*^*3*^*Australian Research Centre in Complementary and Integrative Medicine (ARCCIM), University of Technology Sydney, Sydney, Australia*

##### **Correspondence:** Milena Trifunovic-König (m.trifunovic-koenig@kliniken-essen-mitte.de)

**Purpose**

Systematic overview on acupuncture for Osteoarthritis (OA) of the knee was conducted in order to summarize findings derived from published systematic reviews.

**Methods**

Systematic search of databases [Cochrane Library, PubMed, and Scopus] was performed up to June 2016. A Measurement Tool to Assess Systematic Reviews (AMSTAR) was used for quality assessment of the reviews [1–2].

**Results**

14 systematic reviews (6 conducted meta-analyses) satisfied our eligibility criteria with overall acceptable quality [3–16]. Results published in the reviews rely partly on identical and to some extent on different studies. Reviews generally supported a superiority of acupuncture over standard care (Cao et al. 2012: SMD = −0.43; 95% CI [−0.63, −0.23]) [4], and waiting list (Manheimer et al.2010: SMD = -.96; 95% CI [−1.21, −0.70]) control groups [10]. In comparison to sham findings were rather heterogeneous. White et al.2007 showed positive significant effects of acupuncture (electro and needle) over sham on pain relief (WMSD = 0.87, 95% CI [0.40, 1.34]) [15]. In contrast, Manheimer et al. 2010 opposed the superiority of acupuncture (only needle) over sham treatment (SMD = −0.29; 95% CI [−0.48, −0.10]) [10]. Involvement of different acupunctural interventions (electro and/or needle) across the reviews might be responsible for heterogeneity of the findings. Electro acupuncture seems to be more effective than traditional needle acupuncture in comparison to placebo (Bjordal et al. 2007 (WMD electro = 21.9; 95% CI [17.3, 25.3]; WMD needle = 1.3; 95% CI [−2.7, 4.7]) [3].

**Conclusions**

According to evidence derived from published meta-analyses and systematic reviews acupuncture can be implemented as a complementary treatment for OA of the knee.

**References**

1. Shea BJ, Grimshaw JM, Wells GA, Boers M, Andersson N, Hamel C, et al. Development of AMSTAR: a measurement tool to assess the methodological quality of systematic reviews. BMC medical research methodology. 2007;7(1):1.

2. Shea BJ, Hamel C, Wells GA, Bouter LM, Kristjansson E, Grimshaw J, et al. AMSTAR is a reliable and valid measurement tool to assess the methodological quality of systematic reviews. Journal of clinical epidemiology. 2009;62(10):1013–20.

3. Bjordal JM, Johnson MI, Lopes Martins RA, Bogen B, Chow R, Ljunggren AE. Short-term efficacy of physical interventions in osteoarthritic knee pain. A systematic review and meta-analysis of randomised placebo-controlled trials. BMC musculoskeletal disorders [Internet]. 2007; 8(51).

4. Cao L, Zhang XL, Gao YS, Jiang Y. Needle acupuncture for osteoarthritis of the knee. A systematic review and updated meta-analysis. Saudi medical journal. 2012;33(5):526–32.

5. Corbett MS, Rice SJC, Madurasinghe V, Slack R, Fayter DA, Harden M, et al. Acupuncture and other physical treatments for the relief of pain due to osteoarthritis of the knee: Network meta-analysis. Osteoarthritis and Cartilage. 2013;21(9):1290–8.

6. Ezzo J, Hadhazy V, Birch S, Lao L, Kaplan G, Hochberg M, et al. Acupuncture for osteoarthritis of the knee: A systematic review. Arthritis and rheumatism. 2001;44(4):819–25.

7. Galantino ML, Sowers K, Kelly M, Mao J, LaRiccia P, Farrar J. Acupuncture as an adjuvant modality with physical therapy for patients with knee osteoarthritis. Medical Acupuncture. 2009;21(3):157–66.

8. Hou PW, Fu PK, Hsu HC, Hsieh CL. Traditional Chinese medicine in patients with osteoarthritis of the knee. Journal of traditional and complementary medicine. 2015;5(4):182–96.

9. Manheimer E, Linde K, Lao L, Bouter LM, Berman BM. Meta-analysis: Acupuncture for osteoarthritis of the knee. Annals of internal medicine. 2007;146(12):868–77.

10. Manheimer E, Cheng K, Linde K, Lao L, Yoo J, Wieland S, et al. Acupuncture for peripheral joint osteoarthritis. Cochrane Database of Systematic Reviews [Internet]. 2010; (1). Available from: http://onlinelibrary.wiley.com/doi/10.1002/14651858.CD001977.pub2/abstract.

11. Markow MJ, Secor ER. Acupuncture for the pain management of osteoarthritis of the knee. Techniques in Orthopaedics.2003;18(1):33–6.

12. Puett DW, Griffin MR. Published trials of nonmedicinal and noninvasive therapies for hip and knee osteoarthritis. Annals of internal medicine.1994;121(2):133–40.

13. Scott D, Kowalczyk A. Osteoarthritis of the knee. BMJ clinical evidence. 2007; 2007.

14. Selfe Terry K, Taylor Ann G. Acupuncture and osteoarthritis of the knee: A review of randomized controlled trials. Family & community health [Internet]. 2008; Vol.31(3):[247–54 pp.]. Available from: http://onlinelibrary.wiley.com/o/cochrane/clcentral/articles/303/CN-00790303/frame.html.

15. White A, Foster NE, Cummings M, Barlas P. Acupuncture treatment for chronic knee pain: A systematic review. Rheumatology.2007;46(3):384–90.

16. Yamashita H, Masuyama S, Otsuki K, Tsukayama H. Safety of acupuncture for osteoarthritis of the knee - A review of randomised controlled trials, focusing on specific reactions to acupuncture. Acupuncture in Medicine.2006;24(SUPPL.):S49–S52.

## P169 Adjunctive hatha yoga vs. health education for persistent major depression: a randomized controlled trial

### Lisa Uebelacker^1,2^, Geoffrey Tremont^2,3^, Lee Gillette^4^, Gary Epstein-Lubow^1,2,5^, David Strong^5^, Ana Abrantes^1,2^, Audrey Tyrka^1,2^, Tanya Tran^3^, Brandon Gaudiano^1,2^, Ivan Miller^1,2^

#### ^*1*^*Psychosocial Research, Butler Hospital, Providence, 02906, RL, United States;*^*2*^*Brown University, Providence, RL, United States;*^*3*^*Rhode Island Hospital, Providence, RL, United States;*^*4*^*Independent Yoga Consultant, Providence, RL, United States;*^*5*^*UCSD, San Diego, CA, United States*

##### **Correspondence:** Lisa Uebelacker

**Question**

Can hatha yoga improve depression symptoms in people with continued depressive symptoms despite antidepressant treatment?

**Methods**

We conducted a randomized controlled trial of weekly group hatha yoga classes (n = 63) vs. a weekly health education control group (called Healthy Living Workshop, or HLW; n = 59) in a sample of these individuals. The intervention period lasted 10 weeks. We conducted follow-up assessments 3 and 6 months after the intervention period. Hatha yoga classes were manualized and included breathing exercises, postures, and brief meditation. Classes were designed to be accessible to people who may not have been physically fit. The primary outcome was depression symptom severity assessed by a blind rater using the Quick Inventory of Depression Symptoms. Secondary outcomes included depression response and remission, social and role functioning, general health perceptions, physical pain, and physical functioning.

**Results**

We found that, compared to HLW participants, participants assigned to yoga showed significantly lower levels of depression symptom severity over non-baseline timepoints while controlling for baseline levels of depression (parameter estimate = −1.38, SE = 0.57, p = 0.02). Fifty-one percent of yoga participants demonstrated at least 50% reduction in depression symptoms (i.e., response) at 6 month-follow-up, compared to 31% in the HLW group (OR = 2.31). This difference was statistically significant. Yoga participants showed significantly better social functioning and general health perceptions over time.

**Conclusion**

Hatha yoga may be a useful adjunctive intervention for depressed individuals with insufficient response to antidepressant medications.

Trial Registration: clinicaltrials.gov Identifier: NCT01384916

## P170 Health-related outcomes of the Feldenkrais method in children and adults: state of the art and future perspectives

### Gerhild Ullmann

#### *Social and Behavioral Sciences, School of Public Health, University of Memphis, Memphis, 38152, TN, United States*

**Background**

The Feldenkrais (FK) Method is a contemporary mindful exercise with two techniques: the group method called Awareness Through Movement (ATM) and the one-to-one learning process called Functional Integration (FI). The FK teacher guides students through a sequence of movements either verbally (ATM) or with a gentle, and non-invasive touch (FI). FK has been widely used in the performing arts and in various clinical settings. This review answers questions such as: Who can benefit from FK? What health conditions can be address with FK? And what are the costs?

**Methods**

Databases were searched for articles published between January 1976 and September 2016. Additionally, reference lists and conference proceedings were scanned. A systematic review of the retrieved studies was performed and included health condition, study design, type and duration of the intervention, outcome measures and findings.

**Results**

The majority of studies applied ATMs to musculoskeletal disorders such as back pain, neck and shoulder complaints in adults or focused neurological diseases (e.g., multiple sclerosis, Parkinsons disease). Based on RCTs there is evidence that FK improves balance and mobility in older adults. Some findings suggest positive effects of FK on cognitive function, anxiety, symptoms of depression, body image and self-confidence. FK interventions with children frequently combined the two techniques FI and ATM to address special needs.

**Conclusions**

Future studies should examine whether the beneficial effects of FK on balance and mobility can be replicated in clinical populations with movement limitations. The promising findings on cognitive function, anxiety, symptoms of depression and child development warrant further investigations with improved study designs.

## P171 Health benefits of seated Tai Chi

### Gerhild Ullmann^1^, Yuhua Li^2^

#### ^*1*^*Social and Behavioral Sciences, University of Memphis, School of Public Health, Memphis, 38152, TN, United States;*^*2*^*Exercise, Sport and Movement Science, University of Memphis, School of Health Studies, Memphis, TN, United States*

##### **Correspondence:** Gerhild Ullmann

**Background**

Tai Chi, a martial art form developed in 17th century China, has been practiced by millions of Chinese over centuries and gained popularity in Western cultures. This mind-body activity is typically performed in a standing position and has documented beneficial effects on both psychological and physical well-being. In recent years, research with seated tai chi, sitting tai chi and wheelchair tai chi programs have increasingly gained attention, in particular due to the large rates in mobility disability. The question is whether practicing seated tai chi produces similar health effects as standing tai chi.

**Methods**

PubMed and Google Scholar were searched for articles published between January 2000 and September 2016 using seated tai chi, sitting tai chi and wheelchair tai chi as keywords. A systematic review of the retrieved studies was performed and included health condition, study design, type and duration of the intervention, outcome measures and findings.

**Results**

Six studies were identified (two randomized controlled trials, three single-arm trials, and one case series). The populations studied included residents in long-term care facilities and nursing homes as well as people with Parkinsons disease, stroke survivors and people with spinal cord injuries. These studies examined cognitive function, depressive symptoms, quality of life, pain, fatigue, heart rate variability and functional changes. The reported findings on functional changes, pain and depressive symptoms were inconclusive. One randomized controlled trial found that quality of life improved after practicing seated tai chi for 26 weeks (40 minutes, 3 days weekly).

**Conclusions**

More randomized controlled trials are needed to examine functional changes, pain, depressive symptoms and quality of life. These studies should include measures of upper extremity muscle strength.

## P172 Integrative treatment of Vascular Blockages – CAD, CVA, DVT with SHARP – (Sanjeevan Heart Attack Rehabilitation Program)

### Sujata Vaidya^1^, Vinod Marathe^1,2^

#### ^*1*^*Health Solutions, Pune, India;*^*2*^*Sharp Health Care, HealthCare, Pune, India*

##### **Correspondence:** Sujata Vaidya

Research: to find a better, more effective, sustained option of treatment for the global threat of Vascular Disease. Clinical care that is not Technology dependant, effective in its ease of administration to the patient and follow up; early symptomatic relief, backed by clinical investigations. Traditional healing System as it respects and has the foundation of AYURVEDA that is a scientific natural, traditional health science encompassing aahar (food, nutrition and dietics based on the personal constitution) Vihar (that defines lifestyle modifications that are follow-able), Vichar (as stress, competition and a fast paced life is the adage to disease, disorders); Vyaama (yogasana and more that make supple the functionality of the being) Aaushadhi (Ayurvedic capsules SUVED, REIMMUGEN colostrums) because chronic disorders require medicinal care, support natural recovery, rejuvenation and repair, beyode symptomatic relief and drug based side effects.

SHARP – (Sanjeevan Heart Attack Rehabilitation Program) includes the principles of these and the results have been seen in a double blind, placebo controlled clinical trial (registered with CTRI, Bio Med and WHO). The Pilot study on 60 subjects over 3 months results: 100% functional recovery including increased stamina, reduced to stopped symptoms of chest pain, head ache, body pains, throbbing in head (CVA), breathlessness. Clinical changes in 75% showed significant improvement in IMT taken as a marker of atherosclerosis: EF. Control subjects did not have significant improvements.

SHARP is a viable integrative treatment option to the growing threat of vascular disorder.

## P173 Ketogenic diet for dog with Cavernous Hemangioma

### Ana C Vale, Jacquelyne Motta, Fabíola Donadão

#### *Clinic, Vila Velha, 29102010, Brazil*

##### **Correspondence:** Ana C Vale

**Objective**

The objective of this study is to verify the effectiveness of ketogenic diet as adjunctive therapy in the treatment of abdominal cavernous hemangioma in a dog.

**Methods**

Abdominal ultrasound results from a four-year old Shih-Tzu showed the formation of hyperechoic nodules in the umbilical region.

The dog went through three surgeries, including surgical biopsy with histopathological diagnosis of cavernous hemangioma. After all unsuccessful attempts, the patient took chemotherapy as an option to stop tumor growth, also without success. As an alternative therapy, a nutritional program with ketogenic diet was adopted.

The ketogenic diet consists in the replacement of carbohydrates by healthy fats, which are converted to ketones, and in the intake of moderate amounts of high quality protein to metabolize ketone bodies as an alternative fuel, when glucose availability is low.

**Results**

During dietary therapy, the patient was fed ketogenic diet (healthy fat protein very few carbohydrates) for a monitoring period of 30 days, while further evaluations were made. The results showed reduction of about 10% of the body weight, and no tumor growth detected by ultrasonography. This case study is still in progress.

**Conclusion**

The ketogenic diet can provide anti-angiogenic and pro-apoptotic mechanisms, suggesting that tumor cells have reduced ability to metabolize these ketone bodies for energy. It can offer hope against cancer, both for prevention and treatment.

## P174 The impact of euthmy therapy in heart rate variability: a systematic review

### Angela C Valente, Luana C Carvalho Valente, Ricardo Ghelman (ric.ghelman@gmail.com)

#### *Instituto do Tratamento do Câncer Infantil – Department of Pediatric Oncology, Faculdade de Medicina da Universidade de São Paulo, Sao Paulo, 05410-03, Brazil*

##### **Correspondence:** Angela C Valente

**Background**

Eurythmy (EUR) is a therapy that comes from the Anthroposophical Medicine and consists of body movements combined with music and poetry in order to exercise therapeutic functions to the individual. The impact of the eurythmy in the autonomic nervous system can be accessed through the heart rate variability (HRV), which is a non-invasive and objective method to evaluate this impact.

**Objective**

To evaluate scientific findings of the influence of eurythmy in heart rate variability.

**Materials and Methods**

This study searched articles from electronic data platforms such as PubMed, The Cochrane Library, Bireme/LILACS and MEDLINE for randomized and non randomized clinical trials, clinical trials and controlled clinical trials that demonstrated the impact of eurythmy in heart rate variability. Cochrane and TREND statement were used for articles analisis.

**Results**

Of the 27 articles founded by the search strategy, only four were selected for full reading. The total number of patients were 63. They showed the direct connection between intervention and HRV. Two studies assessed the HRV during the practice of EUR compared to conventional ergometer exercise and both noted significant fluctuations in the HRV during eurythmy practice when compared to conventional ergometer (CE). One found that very low frequency (VLF) and high frequency (AF) increased significantly (p <0.001) in the practice of EUR compared to CE. Another study noted a significant increase in VLF and ultra low frequency (ULF) wave pattern after EUR (p <0.001). The EUR also led to deep states of relaxation and a better quality of sleep in another study as a result of a reduced ULF.

**Conclusion**

The articles included in this review were not randomized and two of them did not have control groups in their study design. Small population was evaluated (n = 63). Despite this, eurythmy showed impact on HRV towards parasympathetic tone. Concomitant to this, EUR can be performed in non-strenuous way, broadening the spectrum of its activity. Conducting randomized controlled studies is strongly recommended.

## P175 The use of natural oils may help reduction of blood sugar level both in diabetes mellitus type 1 and type 2 patients

### Dusan Vesovic^1^, Dragan Jevdic^2^, Aleksandar Jevdic^3^, Katarina Jevdic^4^, Mihael Djacic^4^, Dragica Letic^5^, Drago Bozic^5^, Marija Markovic^5^, Slobodan Dunjic^6^

#### ^*1*^*VISAN - Sanitary Medical School of Applied Sciences, Belgrade, 11000, Serbia;*^*2*^*Private practice “Astrocit”, Vrsac, Serbia;*^*3*^*Private practice “Dr Jevdic”, Vrsac, Serbia;*^*4*^*Special neuropsychiatric hospital “Dr S. Bakalovic”, Vrsac, Serbia;*^*5*^*Health care Center “1. Oktobar”, Plandiste, Serbia;*^*6*^*Center for Integrative procedures and Supplements “Dr Dunjic”, Belgrade, Serbia*

##### **Correspondence:** Dusan Vesovic

**Background**

Prevalence of diabetes mellitus is on the rise worldwide. The aim of this paper was to point out the importance of the use of integrative approach in treating diabetes mellitus type1 and type2 (DM1; DM2) patients.

**Materials and methods**

76 patients of both gender were included in the study. 40 of them were DM1 patients, while other 36 were DM2 patients. There was no statistically significant difference between mean age of DM1 and DM2 patients (58.55–14.97 yrs vs. 63.94–9.27 yrs; p = 0,066). The same was true for body weight - 86.00–19.5 kg vs. 81.94–16.82 kg; p = 0,337. They were examined by ANESA apparatus at the beginning of the intervention, after one and, also, after two months. Patients changed their diet, and, additionally to their insulin and/or oral drug therapy, they were prescribed natural oils (“Ulje1”, “Ulje2”, “Ulje10”, “Ulje14”, “Ulje54” “Ulje80”, and “Ulje89”) made by “Planet zdravja” (Slovenia). Statistical analysis was done by using SPSS v. 17.0.

**Results**

Examinees were distributed in four age ranks (till the age of 30; from 31 to 50; from 51 to 70; above 71). At the beginning of the study, the highest prevalence of patients was in group from 31 to 50 – 56 examinees (73,7%). Of those patients, 34 patients (60,7%) had elevated blood sugar level (BSL). After one month of the study, 14 patients (25%) had elevated BSL, while after two months, the number of patients having elevated BSL dropped to 8 (14,3%).

**Conclusions**

Concomitant use of classic drug therapy with natural oils and diet change can help restoring balance in human body leading to improvement of BSL.

## P176 An integrative approach achieved by concomitant use of classic therapy with natural oils and diet change can help to improve blood glucose level in diabetes mellitus patients

### Dusan Vesovic^1^, Dragan Jevdic^2^, Aleksandar Jevdic^3^, Katarina Jevdic^4^, Mihael Djacic^4^, Dragica Letic^5^, Drago Bozic^5^, Marija Markovic^5^, Gordana Ruscuklic^6^, Dezire Baksa^6^, Slobodan Dunjic^7^

#### ^*1*^*VISAN - Sanitary Medical School of Applied Sciences, Belgrade, 11000, Serbia;*^*2*^*Private practice “Astrocit”, Vrsac, Serbia;*^*3*^*Private practice “Dr Jevdic”, Vrsac, Serbia;*^*4*^*Special neuropsychiatric hospital “Dr S. Bakalovic”, Vrsac, Serbia;*^*5*^*Health care Center “1. Oktobar”, Plandiste, Serbia;*^*6*^*“Planet Zdravja”, , Ljubljana, Slovenia;*^*7*^*Center for Integrative procedures and Supplements “Dr Dunjic”, Belgrade, Serbia*

##### **Correspondence:** Dusan Vesovic

**Purpose**

Diabetes mellitus IS chronic non-communicable diseases and is on the rise worldwide. The aim of this paper was to reveal whether the use of natural oils additionally to standard therapy of diabetes mellitus type1 and type2 (DM1; DM2) may help regulation of blood glucose level (BGL) in patients.

**Materials and methods**

76 patients of both gender were included in the study. 40 of them were DM1 patients, while other 36 were DM2 patients. There was no statistically significant difference between mean age of DM1 and DM2 patients (58.55–14.97 yrs vs. 63.94–9.27 yrs; p = 0,066). The same was true for body weight - 86.00–19.5 kg vs. 81.94–16.82 kg; p = 0,337. They were examined by ANESA apparatus at the beginning of the intervention, after one and, also, after two months. Patients changed their diet, and, additionally to their insulin and/or oral drug therapy, they were prescribed natural oils (“Ulje1”, “Ulje2”, “Ulje10”, “Ulje14”, “Ulje54” “Ulje80”, and “Ulje89”) made by “Planet zdravja” (Slovenia). Statistical analysis was done by using SPSS v. 17.0.

**Results**

Examinees were distributed in two groups based on BGL – patients having BGL within normal range – N, and increased BGL - I. At the beginning of the study, 43,2% of patients were in N group, while 56,8% were in I group; after one month of the study, 67,6% of all examinees were in N group, while two months after the intervention was commenced, 78,6% of patients had their BGL within normal range.

**Conclusions**

This is a good example how concomitant use of classic (insulin or oral) therapy with natural oils and diet change can help to improve BGL regulation.

## P177 Concomitant use of classic therapy with natural oils and diet change can help to improve cholesterol level in diabetes mellitus patients

### Dusan Vesovic^1^, Dragan Jevdic^2^, Aleksandar Jevdic^3^, Katarina Jevdic^4^, Mihael Djacic^4^, Dragica Letic^5^, Drago Bozic^5^, Marija Markovic^5^, Gordana Ruscuklic^6^, Dezire Baksa^6^, Slobodan Dunjic^7^

#### ^*1*^*VISAN - Sanitary Medical School of Applied Sciences, Belgrade, 11000, Serbia;*^*2*^*Private practice “Astrocit”, Vrsac, Serbia;*^*3*^*Private practice “Dr Jevdic”, Vrsac, Serbia;*^*4*^*Special neuropsychiatric hospital “Dr S. Bakalovic”, Vrsac, Serbia;*^*5*^*Health care Center “1. Oktobar”, Plandiste, Serbia;*^*6*^*“Planet Zdravja”, , Ljubljana, Slovenia;*^*7*^*Center for Integrative procedures and Supplements “Dr Dunjic”, Belgrade, Serbia*

##### **Correspondence:** Dusan Vesovic

**Objective**

The aim of this paper was to reveal whether the use of natural oils additionally to standard therapy may help regulation of cholesterol level (CHl) in diabetes mellitus type1 and type2 (DM1; DM2) patients.

**Materials and methods**

76 patients of both genders were included in the study. 40 of them were DM1 patients, while other 36 were DM2 patients. There was no statistically significant difference between mean age of DM1 and DM2 patients (58.55–14.97 yrs vs. 63.94–9.27 yrs; p = 0,066). The same was true for body weight - 86.00–19.5 kg vs. 81.94–16.82 kg; p = 0,337. They were examined by ANESA apparatus at the beginning of the intervention, after one and, also, after two months. Patients changed their diet, and, additionally to their insulin and/or oral drug therapy, they were prescribed natural oils (Ulje1, Ulje2, Ulje10, Ulje14, Ulje54 Ulje80, and Ulje89) made by Planet zdravja (Slovenia). Statistical analysis was done by using SPSS v. 17.0.

**Results**

Examinees were distributed in two groups based on their cholesterol level – patients having cholesterol level within normal range, and patients having an increased level of cholesterol. At the beginning of the study, 81,6% of patients had their CHl within normal range, while 18,4% had an increased; after two months of the study, 92,9% of all examinees had their CHl within normal range, and 7,1% of examinees remained at increased CHl.

**Conclusions**

This is a good example how concomitant use of classic therapy with natural oils and diet change can help to improve regulation of cholesterol level.

## P178 Quality of life in diabetes mellitus patients of both sexes before and after two months of intervention using natural oils and diet change additional to standard therapy

### Dusan Vesovic^1^, Dragan Jevdic^2^, Aleksandar Jevdic^3^, Katarina Jevdic^4^, Mihael Djacic^4^, Dragica Letic^5^, Drago Bozic^5^, Marija Markovic^5^, Gordana Ruscuklic^6^, Dezire Baksa^6^, Slobodan Dunjic^7^

#### ^*1*^*VISAN - Sanitary Medical School of Applied Sciences, Belgrade, 11000, Serbia;*^*2*^*Private practice “Astrocit”, Vrsac, Serbia;*^*3*^*Private practice “Dr Jevdic”, Vrsac, Serbia;*^*4*^*Special neuropsychiatric hospital “Dr S. Bakalovic”, Vrsac, Serbia;*^*5*^*Health care Center “1. Oktobar”, Plandiste, Serbia;*^*6*^*“Planet Zdravja”, , Ljubljana, Slovenia;*^*7*^*Center for Integrative procedures and Supplements “Dr Dunjic”, Belgrade, Serbia*

##### **Correspondence:** Dusan Vesovic

**Purpose**

The aim of this paper was to reveal if there was any difference in quality of life (QoL) in diabetes mellitus type1 and type2 (DM1; DM2) patients of both sexes before and after the intervention.

**Materials and methods**

76 patients of both gender were included in the study. 40 of them were DM1 patients, while other 36 were DM2 patients. There was no statistically significant difference between mean age of DM1 and DM2 patients (58.55–14.97 yrs vs. 63.94–9.27 yrs; p = 0,066). The same was true for body weight - 86.00–19.5 kg vs. 81.94–16.82 kg; p = 0,337. Patients changed their diet, and, additionally to their insulin and/or oral drug therapy, they were prescribed natural oils (“Ulje1”, “Ulje2”, “Ulje10”, “Ulje14”, “Ulje54” “Ulje80”, and “Ulje89”) made by “Planet zdravja” (Slovenia). Assessment of QoL was done before and two months after the study commencing. World Health Organization questionnaire – WHOQOL-BREF was used for quality of life assessment. Statistical analysis was done by using SPSS v. 17.0.

**Results**

Mean value of physical domain in males (M) before the study was 52,7 -8,7, while in females (F) was 42,9 -9,3; p = 0,000. Similar trend was also seen in mean value of psychological health domain before the study - M 56,8 -11,1 vs. F 43,2 -9,2; p = 0,000. This trend remained the same also after two months of the study: M 67,6 -10,9 vs. F 55,8 -12,1; p = 0,000. In other quality of life domains, no significant difference was found between sexes before and two months after the study commenced.

**Conclusions**

This study showed that QoL in segment of physical and psychological domain differs between male and female diabetic patients in favor of males. Further studies are needed to clarify this issue.

## P179 Cholesterol level in diabetes mellitus patients following two months of treatment by using classic therapy accompanied with natural oils and diet change

### Dusan Vesovic^1^, Dragan Jevdic^2^, Aleksandar Jevdic^3^, Katarina Jevdic^4^, Mihael Djacic^4^, Dragica Letic^5^, Drago Bozic^5^, Marija Markovic^5^, Kenan Vrca^6^, Slobodan Dunjic^7^

#### ^*1*^*VISAN - Sanitary Medical School of Applied Sciences, Belgrade, 11000, Serbia;*^*2*^*Private practice “Astrocit”, Vrsac, Serbia;*^*3*^*Private practice “Dr Jevdic”, Vrsac, Serbia;*^*4*^*Special neuropsychiatric hospital “Dr S. Bakalovic”, Vrsac, Serbia;*^*5*^*Health care Center “1. Oktobar”, Plandiste, Serbia;*^*6*^*“Planet Zdravja”, Sarajevo, Bosnia and Herzegovina;*^*7*^*Center for Integrative procedures and Supplements “Dr Dunjic”, Belgrade, Serbia*

##### **Correspondence:** Dusan Vesovic

**Purpose**

Impaired blood glucose regulation in diabetes mellitus patients is often accompanied with the rise of cholesterol level. The aim of this paper was to reveal whether the use of natural oils additionally to standard therapy may help regulation of cholesterol level (CHl) in diabetes mellitus type1 and type2 (DM1; DM2) patients of both sexes.

**Materials and methods**

76 patients of both genders were included in the study. 40 of them were DM1 patients, while other 36 were DM2 patients. There was no statistically significant difference between mean age of DM1 and DM2 patients (58.55–14.97 yrs vs. 63.94–9.27 yrs; p = 0,066). The same was true for body weight - 86.00–19.5 kg vs. 81.94–16.82 kg; p = 0,337. They were examined by ANESA apparatus at the beginning of the intervention, after one and, also, after two months. Patients changed their diet, and, additionally to their insulin and/or oral drug therapy, they were prescribed natural oils (“Ulje1”, “Ulje2”, “Ulje10”, “Ulje14”, “Ulje54” “Ulje80”, and “Ulje89”) made by “Planet zdravja” (Slovenia). Statistical analysis was done by using SPSS v. 17.0.

**Results**

At the beginning of the study, 75% of female patients (FP) had their CHl within normal range; the same was true for 100% of male patients (MP). This difference was statistically significant – p = 0,013. Following two months of the intervention, 90,5% of FP had their CHl within normal range, and 100% of MP; however, this difference was not statistically significant – p = 0,231.

**Conclusions**

This is an example how concomitant use of classic therapy with natural oils and diet change can help to improve regulation of cholesterol level in diabetes mellitus patients, if altered.

## P180 An exploratory study of scrambler therapy for fibromyalgia pain

### Ann Vincent, Dietlind Wahner-Roedler, Mary Whipple

#### *Mayo Clinic, Rochester, 55905, NY, United States*

##### **Correspondence:** Ann Vincent

**Background**

Chronic widespread pain, the cardinal symptom of fibromyalgia, is often debilitating and lacks effective treatment. Despite various pharmacological and nonpharmacological options, pain in fibromyalgia remains a significant problem. The purpose of this exploratory study was to evaluate the effects of Scrambler therapy, a novel non-invasive cutaneous electrostimulation method, on pain in fibromyalgia.

**Methods**

Patients with fibromyalgia received up to 10 treatments with Scrambler therapy over the course of two weeks. The primary measure was a self-report numeric analogue of pain intensity (daily, prior to treatment) and the Fibromyalgia Impact Questionnaire – Revised (FIQ-R) score (weekly), both of which are validated tools used in the assessment of fibromyalgia pain.

**Results**

7 female, Caucasian patients with fibromyalgia, mean age 54 (SD 11.4), were enrolled. Mean average daily pain scores decreased by 38% from baseline to day 10 of treatment and FIQ-R scores decreased by 31%. Reduced FIQ scores (15% below baseline) were maintained at 10 weeks following treatment. While some patients reported discomfort during treatment, treatment was well-tolerated by most patients.

**Conclusions**

The study results suggest that Scrambler therapy may be useful for pain in patients with fibromyalgia without troublesome side effects. The clinically meaningful reduction in pain observed at day 10 is comparable to other non-pharmacological therapies used in fibromyalgia. Although the effect of Scrambler therapy diminished over time, clinically significant reductions in FIQ-R scores were maintained at 10 weeks following treatment. Our data suggest that further study of Scrambler therapy for the amelioration of pain in fibromyalgia is warranted.

Trial Registration: NCT01347723

## P181 Danish cancer patients’ experiences with alternative treatment - can patient experiences challenge biomedical measurements of treatment effects?

### Maria M Vogelius

#### *Institute of Public Health, University of Copenhagen, Copenhagen, 1353, Denmark*

**Purpose**

The purpose of this project was to obtain more knowledge about Danish cancer patients’ experiences with alternative treatment and to explore how patient experiences may challenge the understanding of a linear causality within biomedical measurements of effect.

**Method**

The dataset consisted of 22 Danish cancer patients’ exceptional courses of disease (5 men and 17 women) from the Registry of Exceptional Courses of Disease at the National Research Center in Complementary and Alternative Medicine, Norway. The data was collected using a questionnaire with both open and closed questions.

A qualitative content analysis of patient descriptions was performed based on answers to the open questions from the questionnaire. Furthermore the patient descriptions were divided according to themes that illustrate patient experiences with alternative treatment.

**Results**

The analysis shows that patients experience other effects than symptom relief or cure – e.g. higher quality of life and changed beliefs about health, healing and disease. It also shows that patients become active in the treatment process and consider their own effort as extremely important.

**Conclusion**

The idea of linear causality is considered problematic in the evaluation of complex interventions such as alternative treatments. Therefore it is necessary to perform more analysis based on patients’ experiences with alternative treatment to obtain more knowledge of the different dimensions of effect.

## P182 Evaluation of vitamin blood levels in patients in palliative care

### Claudia Vollbracht^1^, Iris Friesecke^1,2^, Peter W Gündling^1^

#### ^*1*^*Naturopathy & Complementary Medicine, Carl Remigius Medical School, Idstein, 65510, Germany;*^*2*^*Palliativstation, Warnow-Klinik Bützow, Bützow, 18246, Germany*

##### **Correspondence:** Iris Friesecke; Peter W Gündling

**Purpose**

Main purpose of palliative care is amelioration of reduced quality of life and relief of symptoms. Frequently, the symptoms of patients requiring palliative care are coincident with symptoms of vitamin deficiency. Particularly, symptoms such as weakness, pain, shortness of breath, fatigue and loss of appetite are well-known symptoms of deficiency of specific vitamins. The aim of this thesis was to investigate whether patients in palliative are deficient in these vitamins.

**Method**

Blood levels of vitamins, homocysteine and C-reactive protein were measured in 31 palliative patients. Statistical analysis of the data included univariate analysis (data distribution, dispersion and measure of spread) and bivariate correlation analysis. An ethical approval of the university of RostockA2015-0107 exists.

**Results**

Vitamin D3 deficiency (< 62,5 nmol/L) affected 93.5% of patients, vitamin B6 deficiency (< 4,1 ng/ml) 48.4%, vitamin C deficiency (< 4.5 mg/L) 45.2%, vitamin B1 deficiency (< 35 μg/ml) 25.8% und vitamin B12 deficiency (< 193 pg/ml) 12.9% of patients. Folate levels were always in the normal range (>480 ng/ml). Homocysteine levels were elevated (> 12 μmol/L) in 93.5% of patients. A significant negative correlation was apparent for vitamin B1 and pain symptoms (r = −0.384; p = 0.033) and depression (r = −0.439; p = 0.014).

**Conclusion**

Patients in palliative care are severely vitamin deficient. Targeted vitamin supplementation of such patients is warranted and is likely to be therapeutically beneficial. A follow-up study should investigate the effects of targeted vitamin substitution on quality of life and symptom burden.

## P183 Caring hand massage: an intervention for cancer patients undergoing chemotherapy – a pilot study

### Dietlind Wahner-Roedler, Saswati Mahapatra, Rebecca Hynes, Kimberly Van Rooy, Sherry Looker, Aditya Ghosh, Brent Bauer, Susanne Cutshall

#### *Mayo Clinic, General Internal Medicine, Rochester, NY, United States*

##### **Correspondence:** Dietlind Wahner-Roedler

**Purpose**

The purpose of this study was to evaluate whether hand massage has beneficial effects on a cluster of symptoms experienced by cancer patients receiving chemotherapy in an outpatient chemotherapy unit.

**Methods**

*Population:* 40 patients undergoing chemotherapy in the outpatient setting were approached by a volunteer caring hand massage team. Informed consent was obtained. *Interventio*n: A 20 minute hand massage provided by “Mayo Clinic’s Caring Hands” volunteer team trained in hand massage therapy. The sessions were individualized according to patient preference and expressed needs. *Outcome measures*: Visual Analog Scales (VAS) for pain, fatigue, anxiety, muscular discomfort, nervousness, stress, happiness, energy, relaxation, calmness and emotional wellbeing (scale 0–10) prior to and after the intervention and a post therapy satisfaction survey. *Statistical Analysis*:Patients’ demographics were summarized using descriptive statistics.VAS total scores were compared between groups at each time point using two-group t-test.

**Result**

Of 40 participants 19 were males. Median age: 59.5 years. Significant improvement after hand massage was observed in VAS scores of fatigue, anxiety, muscular discomfort, nervousness, stress, happiness, energy, relaxation, calmness and emotional wellbeing (p < 0.05). Pain score also improved, however not statistically significant (p = 0.057). All patients indicated that they would suggest Hand Massage to other patients and 37 were interested in a hand massage during their next chemotherapy treatment.

**Conclusion**

Hand massage provided by a trained volunteer team to patients receiving chemotherapy in a busy outpatient chemotherapy unit is of benefit to patients and well received.

Trial registration number: # NCT02742415 (clinicaltrials.gov)

## P184 An emerging new paradigm for complementary medicine – generalised entanglement and new experimental support

### Harald Walach^1^, Ana Borges Flores^2^

#### ^*1*^*Pediatric Gastroenterology, Poznan Medical University, Poznan, 60-572, Poland; 2 Department of Psychology, University of Edinburgh, Edinburgh, United Kingdom*

##### **Correspondence:** Harald Walach (walach@europa-uni.de)

**Background**

Some years ago a new paradigmatic model was proposed that might be able to unite various experiences that are common in complementary and alternative medicine (Walach 2005). It is based on the new concept of a Generalised Quantum Theory (GQT) (Atmanspacher, Römer & Walach 2000) that also predicts Generalised Entanglement (GET) correlations. Meanwhile there is experimental support for the model.

**Method**

A meta-experiment has been developed by Walter von Lucadou with three positive experimental realizations that has now been replicated independently two times. It uses a random number generator that drives a fractal display. Voluntary participants are seated in front of the computer with the instruction to “influence the direction the fractal grows or shrinks using their intention or will only”. An experiment consists of 9 runs with 3 different instructions to shrink or grow the fractal, or keep it stable. The experiment is moved forward by key-presses. Out of this set-up 5*9 (45) physical variables are created and 5*9 (45) psychological variables. These 45*45 variables are correlated across all experiments, yielding a correlation matrix with 2025 cells. The theoretical prediction is that there will be more correlations visible than expected by chance or in the control matrix, and that in replications the correlations will swap places across cells, but will, overall, stay significantly visible. A control matrix is also constructed out of an empty run of the system and the psychological variables of a predecessor experiment. The statistical evaluation follows a classical approach using the differences in the number of correlations in both matrices and calculating a z-score, and a Monte-Carlo-simulation using 10.000 simulated runs.

**Results**

Two independent experiments with 100 to 200 participants each have been conducted up to now, and a large multi-center experiment is under way. The two experiments confirm independently, with z-scores between 5 and 7 that in the experimental matrix there are more correlations than should be expected by chance or than there are in the control matrix. A more conservative simulation analysis confirms the results with z = 2.

**Conclusion**

Generalised Entanglement seems to be indeed a feature of reality, if suitable rituals for a closure of a system are chosen. This lines up with attempts by Beauvais to understand results of high dilution experiments.

## P185 RegentK and physiotherapy improve knee function after anterior cruciate ligament rupture without surgery after 1 year – a randomised controlled trial

### Harald Walach^1^, Michael Ofner^2^, Andreas Kastner^3^, Gerhard Schwarzl^4^, Hermann Schwameder^5^, Nathalie Alexander^5^, Gerda Strutzenberger^5^

#### ^*1*^*Pediatric Gastroenterology, Poznan Medical University, Poznan, 60-572, Poland;*^*2*^*Institute of Pathophysiology, Medical University Graz, Graz, Austria;*^*3*^*Department of Traumatology, University of Linz, Linz, Austria;*^*4*^*Department of Radiology, University of Linz, Linz, Austria;*^*5*^*Sports Science, University of Salzburg, Salzburg, Austria*

##### **Correspondence:** Harald Walach

**Background**

Recent data have opened the debate whether conservative treatment of anterior cruciate ligament (ACL) rupture might be an alternative treatment option to surgery. In a previous study such a conservative treatment, Regeneration Treatment according to Mohammed Khalifa (RegentK) had good effects over physiotherapy. This trial was designed to answer the question whether RegentK would be able to heal ACL rupture.

**Methods**

This was a randomised controlled trial of one treatment of RegentK against myofascial mobilisation technique (MMT), another type of intensive physiotherapy in 20 patients with fresh ACL rupture during the previous 4 weeks.

**Results**

Both groups were comparable at baseline in all parameters, and specifically in the main outcome criterion IKDC (2000) score that was measured before, immediately and 3 months after treatment, and 1 year later; MRI data were taken before treatment and 1 year after treatment. A repeated measures analysis of variance showed a strong effect of time (p < 0.0001; partial eta2 = 0.81) and no significant interaction or group effect. Both groups reached near full function after one year. IKDC was 90,9 (SD 6,7) for the RegentK group and 93.3 (SD 3,1) for MMT.

**Conclusion**

One treatment of enhanced MMT physiotherapy or RegentK can induce a self-healing response and lead to nearly full recovery of a ruptured anterior cruciate ligament after one year.

Trial Registration: NCT01762371

## P186 Effect of Qinlingye Extract on PPARγ in RAW264.7 Cells and HKC Cells

### Jie Wang^1^, Yan Lu^2^, Wen Gu^3^, Chengcheng Zhang^4^, Xianwei Bu^1^, Honghong Zhang^1^, Jianping Zhang^1^, Yuxi He^1^, Xiaoxu Zhang^1^, Fengxian Meng^1^

#### ^*1*^*Dongfang Hospital, Beijing University of Chinese Medicine, Department of Rheumatology, Beijing, China;*^*2*^*Xiyuan Hospital, China Academy of Chinese Medical Science, Emergency Department, Beijing, China;*^*3*^*Beijing Hospital of Traditional Chinese Medicine, Department of Rheumatology, Beijing, China;*^*4*^*Dongfang Hospital, Beijing University of Chinese Medicine, Department of Nephrology, Beijing, China*

##### **Correspondence:** Fengxian Meng

**Objective**

To investigate the effect of Qinlingye extract(QLYE) on PPARγ in RAW264.7 cells and HKC cells, and to explore its mechanism of inhibiting uric acid(UA)-induced renal immune inflammatory injuries.

**Methods**

RAW264.7 cells were induced by LPS in model group. While stimulated by LPS, the administered groups were intervened by high-,middle-,low-dose of QLYE (2000,1000,500umol/L). After 6,12,24 h of intervention, cell media, total RNA and protein were extracted, and the mRNA transcription and protein expression of PPARγ, MCP-1 and IL-6 were detected. HKC cells were induced by UA in model group. While stimulated by UA, the administered groups were intervened by high-,middle-,low-dose of QLYE(1000,500,250umol/L).After 24,36,48 h of intervention, the total RNA and protein were extracted, and the mRNA transcription and protein expression of PPARγ were detected.

**Results**

1. In RAW264.7 cells: Compared with control group, the mRNA transcription and protein expression of PPARγ were lower, MCP-1 and IL-6 were higher at 6,12,24 h in model group (P < 0.05, P < 0.01). Compared with model group, the mRNA transcription and protein expression of PPARγ were higher, MCP-1 and IL-6 were lower at 6,12,24 h in QLYE groups (P < 0.05, P < 0.01).
2. In HKC cells: Compared with control group, the mRNA transcription and protein expression of PPARγ at 24,36,48 h were lower (P < 0.05, P < 0.01) in model group; Compared with model group, the mRNA transcription of PPARγ in QLYE groups at 24 h and in low-dose group at 48 h were higher (P < 0.05, P < 0.01), the protein expression of PPARγ in QLYE groups at 36 h were higher (P < 0.05, P < 0.01).

**Conclusion**

QLYE may inhibit the expression of inflammatory factors via up-regulating the expression of PPARγ to ameliorate UA-induced renal immune inflammatory injuries.

## P187 Exploring the mechanisms of Yinlai Decoction intervening the mice model of FM1 virus infected compound with high-fat and protein-diet

### Shang Wang, He Yu, Jinfeng Shi, Yu Hao, Tiegang Liu, Jun Wu, Zeji Qiu, Xiaohong Gu

#### *Beijing University of Chinese Medicine, Beijing, 100029, China*

##### **Correspondence:** Shang Wang

**Objective**

To explore mechanism of Yinlai decoction intervening the mice model of FM1 virus infected compound with high-fat and protein-diet.

**Methods**

A diet-induced mice model with high-fat and protein-diet were built, and the EGG was recorded after four days high-fat and protein-diet, and the serum levels of Gas and VIP were observed. In addition, the mice from other seven groups were randomly allocated into blank control group, normal diet group, high-fat and protein-diet group, Rivavirin group, Shuanghuanglian group, Xiaoerhuashiwan group and Yinlai decoction group. mice model of virus infected compound with high-fat and protein-diet were involved in all the treating groups and the mice were infected by FM1 influenza virus via nose. The life protection and mean survival days were calculated. The lung injuries were observed by H&E method. The expression of gastrin and VIP were detected by using radioimmunoassay kits method. The serum level of IFN-γ, IL-4, IL-6, IL-10 and TNF-α were measured by ELISA method.

**Results**

1. Compared with normal diet group, EGG dominant frequency, EGG swing, instability coefficient were increased in high-fat and protein-diet group (*P* <0.05, *P* <0.01, *P* <0.05). 2. Compared with high-fat and protein-diet group, Rivavirin group and Yinlai decoction group showed significantly higher survival rate (*P* <0.01, *P* <0.05). 3. Compared with high-fat and protein-diet group, the serum level of IL-10 and IFN-γhas been significantly increased (*P* <0.05, *P* <0.05) in Ribavirin and Yinlai decoction groups. While the serum level of IL-6 and TNF-α has been significantly decreased (*P* <0.05, *P* <0.05). 4. Compared with blank control group, severe lung cirrhosis injuries were found in high-fat and protein-diet group, and mild injuries were found in Yinlai decoction group and Ribavirin group.

**Conclusions**

The Yin lai decoction can regulate the level of cytokine, and improve the pathological damages of lung tissue, thus lead to protect the mice with high-fat and protein-diet from being injuried from the FM1 influenza virus.

## P188 The effects of craniosacral therapy on agitation in elderly adults with dementia

### Yuh-Hai Wang, Chi-Jung Lou

#### *Natural Biotechnology, Nanhua University, Dalin, 622, Taiwan*

**Purpose**

The purpose of this study was to obtain preliminary efficacy and to evaluate feasibility of craniosacral therapy (CST) on agitation behaviors of dementia patients.

**Methods**

Nineteen patients with minor to moderate dementia were recruited from a veterans home in southern Taiwan. The attorney-in-fact of the patients were informed and agreed the proceeding of the study. Caregivers of the institution monitor the behaviorsof the participants on a daily basis independently. Recorded irritable behaviors using Chinese translated Cohen-Mansfield Agitation Inventory (CMAI) were evaluated starting 2 weeks before CST intervention and were followed until 2 weeks after the conclusion of therapy. CST intervention was provided once a week, continuous for 6 weeks, with roughly 20 minutes each time. Physically aggressive, physically non-aggressive and verbally agitated behaviors before, during, and after CST were analysized.

**Results**

While the overall scores decreased continuously and significantly during the treatment, it clearly shows that the major contributor of the decrease comes from the decrease in verbally agitated behaviors. The drop in verbally agitated behaviors was more significant for those with few or no visitors. Comparing with minor dementia people, those with moderate dementia seem to dropped more in verbal agitated behaviors as well. It was also noticed that CST might be more effective on patients with 3 or fewer combined chronic diseases.

**Conclusions**

Our results show that CST might be a useful alternative treatment for verbally agitated behaviors on dementia elders. The effectiveness of CST might also depend on individual profiles, including frequency of visits, severity of dementia, and health conditions (number of chronic diseases).

## P189 The MOVA Study: Mindfulness meditation for recurrent ovarian cancer

### Sam Watts

#### *Primary Care, University of Southampton, Southhampton, SO16 5ST, United Kingdom*

**Background**

Women with ovarian cancer (OvCa) who experience disease recurrence experience a high prevalence of depression, anxiety and psychological distress. This lowers patient quality of life and impacts upon important clinical factors. Mindfulness interventions have been shown to help to manage depression and anxiety in other cancer sites but have never been specifically tested in recurrent ovarian cancer. There is also a growing foundation of evidence to show that mindfulness intervention may positively modulate key bio-markers relevant to ovarian cancer biology, including CA125 tumour markers and diurnal cortisol. The aim of this study is to address how a 6-week mindfulness intervention impacts upon 1) depression, anxiety and psychological distress and 2) diurnal cortisol and CA125 tumour markers in recurrent OvCa patients.

**Method**

We plan to recruit 30 recurrent OvCa patients from the Queen Alexandra Hospital in Portsmouth, England. All participants will be invited to complete the Hospital Anxiety and Depression Scale, the EORTC-QLQ-OV28 and the Freiburg Mindfulness Inventory at baseline, post intervention (week 6) and at 12-weeks follow-up. We will also collect CA125 tumour markers and salivary cortisol levels at the same time points.

**Results**

Preliminary findings will be available by January 2017. The primary outcome measure for this investigation will be changes in the percentage incidence scores of depression, anxiety and quality of life. Secondary outcomes will assess changes in diurnal cortisol levels and CA125 tumour markers.

**Conclusions**

It is hoped that this investigation will test the initial feasibility and clinical impact of delivering a OvCa specific mindfulness intervention to a cohort of recurrent OvCa patients.

## P190 Tai Chi for reducing dual task gait variability, a potential mediator of fall risk, in individuals with Parkinson’s disease: A pilot randomized controlled trial

### Peter Wayne^1^, Kamila Osypiuk^1^, Gloria Vergara-Diaz^2^, Paolo Bonato^2^, Brian Gow^1^, Jeffrey Hausdorff^3^, Jose Miranda^4^, Lewis Sudarsky^5^, Daniel Tarsy^6^, Michael Fox^7^, Eric Macklin^8^

#### ^*1*^*Osher Center for Integrative Medicine, BWH/HMS, Boston, MA, United States;*^*2*^*Physical Medicine & Rehabilitation, Spaulding Rehabilitation Hospital, Harvard Medical School, Boston, MA, United States;*^*3*^*Sackler Faculty of Medicine, Tel Aviv University, Tel Aviv, Israel;*^*4*^*Spaulding Rehabilitation Hospital, Physical Medicine & Rehabilitation, Boston, MA, United States;*^*5*^*Neurology, Brigham and Women’s Hospital, Boston, MA, United States;*^*6*^*Beth Israel Deaconess Medical Center, Boston, MA, United States;*^*7*^*Laboratory of Brain Network Imaging and Modulation, Beth Israel Deaconess Medical Center, Boston, MA, United States;*^*8*^*Massachusetts General Hospital, Boston, MA, United States*

##### **Correspondence:** Peter Wayne

**Purpose**

Higher gait stride-time variability while performing a cognitive task (i.e., dual task, DT) reflects poorer cognitive-motor coordination and is predictive of falls in Parkinson’s disease (PD). Tai Chi (TC) reduces falls in PD and DT gait variability in healthy adults. This pilot randomized controlled trial (RCT) evaluated the effect of TC on DT gait variability in individuals with early PD.

**Methods**

We conducted a two-arm waitlist-controlled RCT comparing 6-months of PD-tailored TC plus usual care (UC) to UC alone. Subjects were TC naive, age 40–75 years, and diagnosed with PD within 10 years. TC participants attended classes 2×/week supplemented with DVD-guided home practice. DT stride-time variability was assessed off-medication at baseline, 3 and 6 months.

**Results**

Thirty-two subjects were randomized to TC (n = 16) or UC (n = 16). Twenty-five subjects (78%) completed both follow-up visits, 50% of TC participants attended at least 70% of classes and 75% completed at least 70% of home practice. Six-month reductions in DT gait variability were larger in TC (14.1%) vs. UC (1.0%) (effect size 0.29; p = 0.47). If the effect of TC could be increased to yield a 25% improvement in DT gait variability (a magnitude associated with a clinically meaningful reduction in falls) through improved TC compliance or longer exposure period, a sample size of 114 (57 per group) would provide 80% power for a future trial.

**Conclusion**

An adequately powered clinical trial to test the efficacy of TC in improving cognitive-motor interactions in PD is warranted and feasible.

## P191 Withdrawn

## P192 Evaluation of cardiovascular risk associated with SKI306X use in patients with Osteoarthritis and Rheumatoid Arthritis

### Yeonju Woo^1^, Min K Hyun^2^

#### ^*1*^*Preventive Medicine, College of Korean Medicine, Dongguk University Graduate School, Seoul, South Korea;*^*2*^*Preventive medicine, College of Korean Medicine, Dongguk University, Gyeongju-si, South Korea*

##### **Correspondence:** Min K Hyun

**Purpose**

SKI306X (Joins®) was anti-inflammatory analgesic herbal medicine extract and clinically approved for treatment of osteoarthritis.

This retrospective cohort study investigated cardiovascular safety of SKI306X, celecoxib and naproxen.

**Methods**

We used the National Health Insurance Service–National Sample Cohort (NHIS-NSC). This study investigated patients over 20 years old with osteoarthritis and rheumatoid arthritis who received a single prescription of SKI306X, celecoxib or naproxen at least once from January 1, 2011 until December 31, 2012. Patients who received SKI306X, celecoxib or naproxen from January 1, 2009 to December 31, 2010 were excluded. Data pertaining to the included patients were investigated to determine the risk of major cardiovascular events associated with the aforementioned prescription using the Cox proportional hazards model after being adjusted for sex, age and comorbidity.

**Results**

A total of 27,253 patients were selected, among which 10,983 were prescribed SKI306X, 12,311 celecoxib and 3,959 naproxen. The incidence of major cardiovascular events was highest for celecoxib (15.4%), followed by SKI306X (8.6%) and naproxen (8.0%). Celecoxib had a higher cardiovascular risk than naproxen, while SKI306X did not have a higher risk of cardiovascular events than naproxen. In the sensitivity analysis, SKI306X was associated with 1.36 times (95%CI: 1.08–1.72) more cardiovascular events than naproxen during 15 to 60 days of prescription, but not at less than 14 days or more than 61 days.

**Conclusions**

Evaluation of the cardiovascular safety of the anti-inflammatory analgesic herbal medicine SKI306X revealed that it was not differ to that of naproxen, which is known to have low cardiovascular risk while celecoxib was higher risk than naproxen.

## P193 Analysis of the luteal phase on PMS patients’ anger feeling based on an emotion-inducing experiment: An EEG study

### Hao Wu, Tian-Fang Wang, Yan Zhao, Yu Wei, Lei Tian, Lei He, Xue Wang

#### *Beijing University of Chinese Medicine, TCM Diagnostics, Beijing, China*

##### **Correspondence:** Tian-Fang Wang (tianfangwang2000@163.com)

**Objective**

To investigate the anger feeling and electrical activity of brain induced by an emotional film experiment among PMS and health female college students during the luteal phase.

**Methods**

All the participants had a regular menstrual cycle (21–35 days), according to the diagnosis standard of PMS provided by the American Congress of Obstetricians and Gynecologists, 31 PMS subjects and 26 health subjects are included. The participants were shown an anger-inducing experimental film during the luteal phase along with a neutral film for comparison. We simultaneously evaluated the anger feeling and recorded electrical activity of brain.

**Results**

By means of the Self-Assessment Manikin (SAM) in a paper-and-pencil procedure, both the groups showed the valence declined, the arousal, the dominance and the intensity of anger rising compare to the baseline data (P < 0.05). By means of EEG data (Delta, Theta, Beta, Alpha, Gamma) in PMS group showed a statistic difference compare to the neutral film stage (P < 0.05), while the health group showed negative results.

**Conclusion**

PMS group experience stronger anger feelings induced by an emotional film during the luteal phase than health group, and show the extension of electrical activity of brain.

The work was funded by the National Science Foundation of China (No.81373771)

Keywords: PMS; anger; the luteal phase

## P194 Chinese herbal medicine Yinzhihuang combined with phototherapy for treating neonatal jaundice: a systematic review of randomized trials

### Ruohan Wu^1^, Shuo Feng^1^, Mei Han^1^, Patrina H Y Caldwell^2^, Shigang Liu^3^, Jing Zhang^4^, Jianping Liu^1^

#### ^*1*^*Beijing University of Chinese Medicine, Beijing, 100029, China;*^*2*^*The Children’s Hospital at Westmead and Discipline of Paediatrics and Child Health, University of Sydney, Sydney, Australia;*^*3*^*Guang’anmen Hospital, China Academy of Chinese Medical Sciences, Beijing, China;*^*4*^*Department of Biostatistics and Epidemiology, University of Pennsylvania, Philadelphia, United States*

##### **Correspondence:** Ruohan Wu

**Objective**

To systematically evaluate the effectiveness and safety of Chinese herbal medicine Yinzhihuang combined with phototherapy for neonatal jaundice.

**Method**

A comprehensive search was performed to identify randomised trials in four Chinese databases, two English databases and two trial registries from inception to June 2016. Methodological quality of the trials was assessed according to the risk of bias tool from Cochrane Collaboration. Binary or continuous data were analyzed with relative risk (RR) or mean difference (MD) and their confidence interval (CI) using Review Manager 5.3.

**Result**

A total of 17 trials (involving 2561 neonates) were included. Fourteen of the 17 included trials had unclear or high risk of bias. Significant differences were detected between yinzhihuang with phototherapy and phototherapy alone for serum bilirubin level (MD -50.25 μmol/L, 95% CI −64.01 to −36.50, I2 = 98%; 7 trials, random-effect model), failure of jaundice resolution (RR 0.24, 95% CI 0.16 to 0.37; 11 trials), and time of jaundice resolution (MD -2.17 days, 95% CI −2.96 to −1.38, I2 = 98%; 6 trials; random-effect model). Adverse events were reported in eight trials but none were serious. Trial sequential analysis for serum bilirubin suggested that the cumulative Z-curve (representing 1478 neonates) reached the required information size (n = 1301).

**Conclusion**

Based on trials with low methodological quality, Yinzhihuang oral liquid combined with phototherapy seemed to be safe and superior to phototherapy alone for reducing serum bilirubin and improving jaundice resolution in neonatal jaundice. This benefit needs to be confirmed in future rigorous trials.

## P195 Manipulation discrepancies between senior and junior acupuncturists for Chemotherapy Induced Nausea and Vomiting (CINV)

### Ruyu Xia^1^, Qianyun Chai^2^, Yutong Fei^3^, Zhongning Guo^4^, Congcong Wang^5^, Zhijun Liu^6^, Xun Li^1^, Ying Zhang^1^, Jianping Liu^1^

#### ^*1*^*Centre for Evidence-Based Chinese Medicine, Beijing University of Chinese Medicine, Beijing, China;*^*2*^*Journal of Traditional Chinese Medicine, Beijing, China;*^*3*^*Beijing University of Chinese Medicine, Beijing, China;*^*4*^*Department of Oncology, Xiyuan Hospital, China Academy of Chinese Medical Sciences, Beijing, China;*^*5*^*China Association of Traditional Chinese Medicine, Beijing, China;*^*6*^*Beijing Kawin Technology Share-holding CO., Ltd, , Beijing, China*

##### **Correspondence:** Ruyu Xia

**Purpose**

To compare the manipulation parameters of senior and junior acupuncturists in detail based on experimental data.

**Methods**

Cancer patient receiving cisplatin-based chemotherapy and 5-HT3 as antiemetic treatment were included. Patients were randomly allocated into senior group and junior group. Senior group manual acupuncture by senior acupuncturists (clinical experience > 15 years), junior group manual acupuncture by junior acupuncturists (< 5 years). Analysis firstly three days acupuncture prescription, compare details of acupuncture, flexibility of syndrome differentiation and points selection between groups.

**Results**

45 patients were included, 24 patients in senior group, 21 patients in junior group. senior group totally select 26 points, acupuncture 423 times, junior group select 17 points, acupuncture 253 times, the number and flexibility of points selection have no significant difference (P = 0.34). The flexibility of syndrome differentiation also have no significant difference (P = 0.731).We select the most used three points ST36 (Zusanli), RN12 (Zhongwan) and P6 (Neiguan) to analyze acupuncture detail. As to needling depth of ST36 (Zusanli) senior acupuncturists reported deeper distance than juniors (P = 0.022). For retaintion time of RN12 (Zhongwan) needling, senior acupuncturists mostly preferred 20 minutes, juniors usually chose 20 to 30 minutes (P = 0.029). As needling P6 (Neiguan) senior acupuncturists, besides using even method, inclined to use reinforcing method than juniors (P = 0.026).

**Conclusion**

The expertise difference of acupuncturists might impact needle manipulation, insertion depth and retaining duration more than symptom differentiation and points selection for the treatment of CINV. We need to consider clinical experience and operating habits of acupuncturists in protocols of the trials.

## P196 Nootkatol suppressed atopic dermatitis-like skin lesions in NC/Nga mice by inhibiting helper T cell immune responses

### I. J. Yang^1^, V. Ruberio Lincha^1^, S. H. Ahn^2^, D. U. Lee^3^, H. M. Shin^1,4^

#### ^*1*^*Dongguk University College of Korean Medicine, Physiology, Gyeongju-si, South Korea;*^*2*^*Dongguk University College of Korean Medicine, Anatomy, Gyeongju-si, South Korea;*^*3*^*Dongguk University, Division of Bioscience, Gyeongju-si, South Korea;*^*4*^*National Development Institute of Korean Medicine, Gyeongsan-si, South Korea*

##### **Correspondence:** I. J. Yang

**Question**

Sesquiterpenes, secondary metabolites produced mainly in higher plants derivatives possess many biological activities such as anti-inflammatory and antiparasitic activities.

**Methods**

Nootkatol, a new sesquiterpene was evaluated for its therapeutic effects on atopic dermatitis both in vivo and in vitro.

**Results**

The topical application of nootkatol suppressed the development of atopic dermatitis (AD)-like symptoms in an NC/Nga mouse model, including increased infiltration of mast cells, distribution of cutaneous vessel, expression of inducible nitric oxide synthase (iNOS), and production of helper T cell-related cytokines such as IL-13 and IL-22. In vitro studies using tumor necrosis factor (TNF)-$\alpha$ and interferon (IFN)-$\gamma$ treated human keratinocytes (HaCaT) revealed that nootkatol inhibited the aberrant production of Th2 chemokines including thymus and activation regulated chemokine (TARC)/CCL17 and macrophage-derived chemokine (MDC)/CCL22 through blockade of the NF-$\kappa$B pathway. In addition, nootkatol inhibited the production of proinflammatory chemokines such as C-X-C motif chemokine ligand (CXCL) 8 in IL-22-stimulated HaCaT cells.

**Conclusions**

These observations indicated that inhibition of Th2, Th22 cytokine production and their pro-inflammatory functions in skin lesions have been associated with the mechanisms of action of nootkatol in AD.

This study was supported by a grant from the Korean Health Technology R&D Project, Korean Ministry of Health & Welfare (no. HN12C0057), and the Dongguk University Research Fund (2017).

## P197 Traditional Chinese Medicine use amongst people with arthritis: Reports from nationally representative sample of Australian women

### Lu Yang, David Sibbritt, Wenbo Peng, Jon Adams

#### *University of Technology Sydney, Ultimo, 2007, Australia*

##### **Correspondence:** Lu Yang (lu.yang-1@student.uts.edu.au)

Traditional Chinese Medicine (TCM) attracts considerable public support in Australia and elsewhere around the world. TCM is an essential part of the growing field of complementary and alternative medicine (CAM) and TCM use has emerged as a topic of research interest during recent years. While literature suggests CAM (including TCM) use is popular for chronic illness (including arthritis) and a large proportion of arthritis sufferers using CAM consider these medicines to be somewhat or very effective, there remains a lack of detailed empirical investigation of TCM use amongst people with arthritis.

**Purpose**

The aim of this paper is to examine the profile, predictors, and determinants of TCM use amongst people with arthritis.

**Method**

Drawing upon data from the Australian Longitudinal Study on Women’s Health (ALSWH), a population-based cohort study, we use both cross-sectional and longitudinal analyses. Statistical analyses included bivariate chi-square and backward stepwise multiple logistic regression modelling.

**Results**

The result shows a substantial prevalence of TCM use amongst people with arthritis and women with arthritis who use TCM appear to gain significant mental and physical health benefits.

**Conclusions**

Further understanding and analysis of the issues around TCM use are significant for health service provision and organization who provide and manage safe, effective and quality care for people with arthritis. Therefore, the results from this paper will provide important insight of interest and benefits to patients, practitioners and policy makers.

## P198 The analysis of questionnaires about the health condition of students involved in the Korean Medicine Doctors’ Visiting School Program (KMDVSP) - Cohort study: Middle & High school participator of Seong-Nam-

### N Yang^1^, H Sung^2^, S M Shin^3^, H Y Go^2^, H Jung^4^, Y Kim^2^, T Y Park^5^

#### ^*1*^*Catholic Kwandong Univ. Int St.Mary’s hospital, integrative medicine, Incheon, South Korea;*^*2*^*College of Korean Medicine, Semyung University, Korean internal medicine, Chungju, South Korea;*^*3*^*College of Korean Medicine, Semyung University, Korean internal medicine, Jecheon, South Korea;*^*4*^*Korea Health Industry Development Institute, Korean medicine , Osong, South Korea;*^*5*^*Catholic Kwandong Univ. Int St.Mary’s hospital, Incheon, South Korea*

##### **Correspondence:** N Yang

**Question**

The aim of this study was to build base-line data for the Korean Medicine Doctors’ Visiting School Program (KMDVSP) by analyzing a student health survey filled out by the students.

**Methods**

Korean medicine doctors assigned to 20 middle and high schools in Seong-nam visited these schools eight times in five months. During each visit, the assigned doctors performed health consultations and Korean medicine treatment, and taught health education classes. 12115 students answered self-reported questionnaires about their own physical condition at the beginning of the program.

**Results**

In a question about pain, 7080 (58%) reported having a headache, while 4048 (33%) said they had a backache, nuchal pain/shoulder pain was reported by 5993 (49%), dyspepsia was present in 2736 (23%), rhinitis/sinusitis was reported by 4176 (34%), coughing/dyspnea by 7102 (59%), itching/skin rash by 2840 (23%), and constipation was reported by 1091 (9%), while 2264 (18%) said they had diarrhea. Increased urinary frequency/feeling of residual urine was reported by 569 students (5%), and 3324 (27%) said they had insomnia/fitful sleep/morning fatigue. When asked about menstruation, 4450 (83%) of the female students reported irregular menstruation or said they experienced menstrual pain.

**Conclusions**

Understanding the health condition of adolescent students is the starting point to determining national health policy in the future. We have developed the pilot project of KMDVSP and collected research about students health. Based on this data, further studies should be performed in order to develop a cooperative program between schools and the Korean medical center.

## P199 Rhythm-centred music making in community living elderly: a randomized pilot study

### Angela Yap^1^, Yu H Kwan^1^, Chuen S Tan^2^, Syed Ibrahim^3^, Seng B Ang^4^

#### ^*1*^*Duke-NUS Medical School, Singapore, Singapore;*^*2*^*National University of Singapore, Singapore, Singapore;*^*3*^*OneHeartBeat Percussions, Singapore, Singapore;*^*4*^*KK Women’s and Children’s Hospital, Singapore, Singapore*

**Aim**

Quality of life has become an important aspect in the measurement of the health of an individual as the population ages. Rhythm-centred music making (RMM) has been shown to improve physical, psychological and social health. The purpose of this study was to explore the effects of RMM on quality of life, depressive mood, sleep quality and social isolation in the elderly.

**Methods**

A randomised controlled trial with cross over was conducted. The intervention involved 10 weekly RMM sessions. Patient related outcome data which included European Quality of Life-5 Dimensions (EQ5D), Geriatrics Depression Scale (GDS), Pittsburg Sleep Quality Index (PSQI) and Lubben Social Network Scale (LSNS) scores were collected before intervention, at 11th and 22nd week.

**Results**

54 participants were recruited and 31 that remained were analysed. The mean age was 74.65 ± 6.40 years. In analysing the change in patient related outcome variables as a continuous measure, participation in RMM resulted in a non-significant reduction in EQ5D by 0.004 (95%CI: −0.097,0.105), GDS score by 0.479 (95%CI:−0.329,1.287), PSQI score by 0.929 (95%CI:- 0.523,2.381) and an improvement in LSNS by 1.125 (95%CI:−2.381,0.523). In binary analysis, participation in RMM resulted in a 37% (OR = 1.370, 95%CI: 0.355,5.290), 55.3% (OR = 1.553, 95%CI: 0.438,5.501), 124.1% (OR = 2.241, 95% CI = 0.677,7.419) and 14.5% (OR = 1.145, 95% CI = 0.331,3.963) non-significant increase in odds of improvement in EQ5D, GDS, PSQI and LSNS scores respectively.

**Conclusion**

Participation in RMM did not have any significant effect in the quality of life of community living elderly. A larger study is needed to validate the results of this pilot study.

## P200 Knowledge, attitudes and practices toward traditional and complementary medicines among nurses and midwives in North Western Uganda

### Alfred Yayi, Dongwoon Han, Hyea Bin Im, Jung Hye Hwang, Soo Jeung Choi

#### *Global Health and Development, Hanyang University, College of Medicine, Seoul, South Korea*

##### **Correspondence:** Alfred Yayi

**Purpose**

Previous studies show varying levels of knowledge, attitudes and practices of healthcare professionals on Traditional and Complementary Medicine. Few literature is available on low-income settings like Africa, where TCM is widely used without proper regulation. This study aimed to assess knowledge, attitudes and practices of Nurses and Midwives in North Western Uganda towards TCM to gain helpful information for policy-making and to promote rational use of TCM.

**Methods**

A descriptive cross sectional study using quantitative methods of data collection and analysis was conducted. Self-administered structured questionnaires were administered to 300 Nurses and Midwives in six selected public and private non-profit hospitals in North Western Uganda. Data was processed and analyzed using Microsoft Excel and SPSS version 21.

**Results**

49.0% of respondents had never heard of TCM modalities, 40.6% had limited knowledge, and only 10.4% knew pharmacodynamics. Most (81.7%) had no TCM training experience. The main information source was mass-media and oral tradition. Respondents personal use was common (54.9%). Having TCM provider as family, experiences on TCM training, greater than 5 years of service as health professional, working in rural hospitals were associated with increased self-practice and recommendation. Nurse/midwife–patient communication on TCM was irregular due to their limited knowledge.

**Conclusion**

Improving TCM knowledge among health professionals could help to promote safer TCM use. Training them on key aspects of TCM and integrating TCM use assessment as a key part of clinical evaluations can improve TCM knowledge. These public efforts will help to streamline the regulation and standardize its practice.

Keywords: Traditional, complementary and alternative medicines (TCAM), Knowledge, Nurses, Midwives, Uganda

## P201 Effects of Hyeolbuchugeo-tang on osteoclast diffrentiation and bone resorption

### Jeong E Yoo^1^, Ho R Yoo^2^, Sae B Jang^1^, Hye L Lee^3^

#### ^*1*^*Daejeon University, Obstetrics and Gynecology, Daejeon, South Korea;*^*2*^*Daejeon University, Daejeon, South Korea;*^*3*^*Gachon University, College of Korean Medicine, Seongnam, South Korea*

##### **Correspondence:** Jeong E Yoo

**Question**

Osteoclasts are multinucleated bone-resorbing cells that differentiate in response to receptor activator of nuclear factor-κB (NF-κB) ligand (RANKL). Enhanced osteoclastogenesis contributes to bone diseases, such as osteoporosis and rheumatoid arthritis. *Hyeolbuchugeo-tang* (HBC), is traditionally used as an herbal medicine for blood circulation by removing stasis and generating new blood; however, its biological actions on osteoclast diffrentiation and bone resorption have not been elucidated. This study was conducted to investigate the effects of *Hyeolbuchugeo-tang* (HBC) on osteoporosis.

**Methods**

We induced RAW 264.7 cells to differentiate to osteoclasts by RANKL and treated RANKL-induced RAW 264.7 cells with HBC (0, 150, 350, 700 μg/*ml*). To measure osteoclast differentiation and activation, we counted TRAP(+) MNCs and measured mRNA expressions of its related genes (TRAP, MMP-9, cathepsin K, NFATc1, c-Fos, MITF, iNOS, COX-2, TNF-α) by RT-PCR. To assess bone resorption, the bone pit formation were examined under a microscope.

**Results**

HBC inhibited RANKL-induced osteoclast differentiation and bone resorption. HBC decreased TRAP(+) MNCs and inhibited mRNA expressions of TRAP, MMP-9. HBC also decreased the protein levels of nuclear factor of activated T cells cytoplasmic-1 (NFATc1) and cathepsin K. In addition, HBC inhibited the nuclear translocation of NFATc1, c-Fos, and MIFT. Moreover, HBC inhibited bone pit formation.

**Conclusions**

Our data revealed that HBC has anti-bone resorption activity and suggest that HBC potentially be used for the prevention and treatment of osteoporosis.

## P202 Nutritious edible fruits of Solanum indicum act as natural antihypertensive

### Ala’a Youssef^1^, Shahira Ezzat^2^, Amira Abdel Motaal^2^, Hesham El-Askary^2^

#### ^*1*^*Heliopolis University for sustainable development, Medicinal Plants, Nasr City, Egypt;*^*2*^*Cairo University , Medicinal Plants, Cairo, Egypt*

##### **Correspondence:** Ala’a Youssef

Fruits are vital sources of nutritional components and phytochemicals and some of them have folk medicinal uses. *Solanum indichum* ssp. *distichum,* an edible fruit belonging to family Solanaceae, is known in African folk medicine as an antihypertensive agent. This raised our interest to identify its phytochemical constituents, to estimate its nutritional value as well as to investigate its inhibitory effect on Angiotensin Converting Enzyme (ACE) as a possible mechanism of action.

A preliminary phytochemical screening showed that the ethyl acetate and *n*-butanol fractions of the ethanolic extract, were rich in saponins, steroids and flavonoids. The total phenolic and flavonoid contents of the ethanolic extract were estimated as 40.43 ± 0. 68 gallic acid equivalent/g and 50.30 ± 0.42 quercetin equivalent/g, respectively. The nutritional analysis of the fruit revealed the presence of 13.19% of crude protein, 1.62% of fats, and 15.2, 10.2, 3.4 mg/g of vitamin A, calcium, and iron, respectively. Free radical scavenging activities of the mother ethanolic extract, hexane, chloroform, ethyl acetate, butanol fractions were measured by 1, 1-Diphenyl-2-Picryl-Hydrazil radical (DPPH) against gallic acid standard, where the mother ethanolic extract showed the highest antioxidant activity (IC_50_ 4.5 μg/ml). Biological investigation of the mother ethanolic extract and its fractions against Angiotensin Conversion Enzyme (ACE), showed that the chloroform fraction possessed the highest ACE inhibition activity (40%) compared to Captopril® positive standard (80%).

Thus, *Solanum indichum* is considered a promising natural antihypertensive drug.

## P203 Research on “symptom-based strategy of dosage selection” of traditional Chinese medicine: an integrated analysis of seven randomized, controlled, multicenter clinical trials about type 2 diabetes

### Xiaotong Yu^1,2^, Yashan Cui^1^, Ying Zhang^2^, Fengmei Lian^1^

#### ^*1*^*Guang’anmen Hospital, China Academy of Chinese Medical Sciences, Beijing, China;*^*2*^*Beijing University of Chinese Medicine, Beijing, China*

##### **Correspondence:** Xiaotong Yu

**Purpose**

“symptom-based strategy of dosage selection” means adjusting the drugs dosage according to patients’ conditions, which is a treatment strategy used by traditional Chinese medicine (TCM) doctors. Herein, we analyze the data of seven clinical trials about type 2 diabetes (T2D), attempting to demonstrate the strategy’s general applicability.

**Methods**

Clinical data were normalized, and its database was then established by the SAS software using the CDISC standard. Statistical methods such as paired *t* test, multiple regression analysis, ROC curve, chi square test, and youden’s index were adopted to obtain the “indicator” and “cut-off value/timing” of dosage adjusting related with treatment efficacy.

**Results**

1809 subjects were enrolled aggregately. The changes of glycosylated hemoglobin (HbA1c) from baseline to week 12 represented the treatment efficacy. Statistical analysis showed that, in the “indicators” having significant differences compared with the baseline (*P* < 0.05), diastolic BP (DBP) (OR = 1.034) and fasting plasma glucose (FPG) (OR = 0.783) at week 4; FPG (OR = 0.783) at week 6, DBP (OR = 1.037) and FPG (OR = 0.730) at week 8; FPG (OR = 0.741) at week 10; weight (OR = 0.705), body mass index (OR = 2.385), waist-hip ratio (OR < 0.001), DBP (OR = 1.016), FPG (OR = 0.872), and 2 h-PBG (OR = 0.943) at week 12were relevant to the changes of HbA1c respectively (*P* < 0.05). In addition, the five largest areas beneath ROC curve to HbA1c were the FPG of week 4, 6, 8, 10, and 12 (S = 0.601, 0.606, 0.630, 0.634, 0.633 respectively). Furthermore, with decreased 0.1–1.1 mmol/l (0.1 mmol/l interval) as the boundary value, FPG decreased 0.5 mmol/l at week 4 was elected to the final “indicator” and “cut-off value/timing” of dosage adjusting related with efficacy in 12-week treatment (youden’s index = 12.08%).

**Conclusions**

By increasing the sample size, our research revealed that the “symptom-based strategy of dosage selection” of TCM had a general applicability, which provided effective basis for the dosage adjustment to doctors.

## P204 Efficacy, safety and dose finding trial of topical Jaungo application in atopic dermatitis patients: study protocol for a randomized, double-blind, placebo controlled trial

### Younghee Yun^1^, Youme Ko^2^, Jin-Hyang Ahn^1^, Bo-Hyung Jang^2^, Kyu-Seok Kim^3^, Seong-Gyu Ko^2^, Inhwa Choi^1^

#### ^*1*^*Department of Korean Medicine Dermatology, Kyung Hee University Hospital at Gangdong, Seoul, South Korea;*^*2*^*Department of Preventive Medicine, Graduate School, Kyung Hee University, Seoul, South Korea;*^*3*^*Department of Dermatology of Korean Medicine, Kyung Hee University, Seoul, South Korea*

##### **Correspondence:** Inhwa Choi

**Purpose**

Atopic dermatitis (AD) is a common pruritic inflammatory skin disease with an increasing prevalence. AD has many different clinical phenotypes, in choric stage, hyperpigmentation, excoriation, lichenification and dryness are main symptoms. Jaungo is an approved herbal ointment for xerosis cutis in Korea. It is composed of two herbs and three carrier oils. In our previous *in-vitro* and *in-vivo* studies, Jaungo showed anti-inflamatory and anti-allergic effects in AD models.

**Methods/Design**

The study is designed as a randomized, double-blind, placebo-controlled, single center Phase IIa clinical trial to investigate the safety, preliminary efficacy and dose response of Jaungo on AD. The study aims to enroll 34 AD patients. Enrolled participants will be randomly allocated to three parallel groups: the treatment 1, treatment 2 and placebo groups. The treatment 1 group applies Jungo twice a day, the treatment 2 group applies Jaungo and placebo once a day separately, and the placebo group applies placebo twice a day for three weeks. Each participant will be examined Eczema Area and Severity Index, SCORring of Atopic Dermatitis index, transepidermal water loss, the Dermatology Life Quality Index, total IgE, eosinophil count, IL-17, IL-22, IFN-γ before and after applying ointment.

**Discussion**

The trial is currently Ongoing. Enrollment of subjects initiated. There is a need to develop a drug for the treatment of dry, hyperpigmented, scaly and ticked skin in chronic stage AD. There is a case series about the effect Jaungo on AD in 1999, and two recent studies reported the effects of Jaungo on the radiation dermatitis in breast cancer patients and Cutaneous Leishmaniasis. However there is few relavant randomized, controlled clinical trials of Jaungo on AD.This study may provide evidence of effecacy and safety of Jaungo, or provide specific AD symptoms associated with Jaungo.

Trial registration: Clinical Trials.gov Identifier: NCT01486303

Keywords: Dermatitis, Atopic; Jaungo; Randomized controlled trial; Phytoteraphy; Administraion, Topical

## P205 Impact of a multimodal treatment on the actigraphically measured activity pattern in breast cancer patients with cancer related fatigue

### Roland Zerm^1,2^, Augustina Glinz^1^, Danilo Pranga^1^, Bettina Berger^3^, Fadime ten Brink^4^, Marcus Reif^5^, Arnd Büssing^2^, Christoph Gutenbrunner^5^, Matthias Kröz^1,2,3,6^

#### ^*1*^*FIH-Berlin, Berlin, Germany;*^*2*^*Internal Medicine, Gemeinschaftskrankenhaus Havelhöne, Berlin, Germany;*^*3*^*Institute for Integrative Medicine, University Witten/Herdecke, Witten, Germany;*^*4*^*Hannover Medical School, Clinic for Rehabilitative Medicine, Hannover, Germany;*^*5*^*Society for Clinical Research, Berlin, Germany;*^*6*^*Institute for Social Medicine, Epidemiology and Health Economics, Charité University Hospital, Berlin, Germany*

##### **Correspondence:** Roland Zerm

**Purpose**

Breast cancer patients (BC) with cancer-related fatigue (CRF) compared to controls show in some studies a decreased physical activity although data are conflicting. We investigated a 10-week multimodal program (MT) consisting of sleep-education, psycho-education, eurythmy and painting therapy separately or in combination (CT) with aerobic training (AT) and compared MT and CT each with AT regarding improvement of CRF and actigraphically measured activity.

**Methods**

In a comprehensive cohort study BC with chronic CRF were randomized equally or allocated by patients preference. Primary endpoint was a composite outcome of the Pittsburgh Sleep Quality Index (PSQI) and the Cancer Fatigue Scale (CFS-D) after 10 weeks (T1); a second endpoint included physical activity, measured over 72 hours by actigraphy (Actiwatch®). We present the results of the intention-to-treat analysis by a linear model.

**Results**

65 BC were randomized and 61 allocated by preference to the three treatment arms. 105 started the intervention and from 82 patients (AT: n = 14, MT: n = 30, CT: n = 38) actigraphy measures are available. At Baseline (T0) all actigraphy parameters were comparable. After ten week intervention (T1) daytime activity and total 24 hour activity has increased in MT compared to CT (both, p < 0.05) and to AT in the daytime activity (p < 0.05) by tendency compared to AT (p = .0.062). All other actigraphical parameters showed no significant group differences at T1 (sleep latency, sleep efficiency, wake-after- sleep-onset and activity during sleep).

**Conclusions**

A multimodal therapy increased total activity and was superior to combined therapy and aerobic training.

## P206 A multimodal therapy concept for lifestyle optimization in Type 2 Diabetes – design and methodology of the AIM DIABETES study

### Roland Zerm^1,2^, Bert Helbrecht^1^, Danilo Pranga^1^, Benno Brinkhaus^3^, Andreas Michalsen^3^, Matthias Kröz^1,2,3,4^

#### ^*1*^*FIH-Berlin, Berlin, Germany;*^*2*^*Internal Medicine, Gemeinschaftskrankenhaus Havelhöne, Berlin, Germany;*^*3*^*Institute for Social Medicine, Epidemiology and Health Economics, Charité University Hospital, Berlin, Germany ;*^*4*^*Institute for Integrative Medicine, University Witten/Herdecke, Witten, Germany*

##### **Correspondence:** Roland Zerm

**Purpose**

The prevalence of Diabetes mellitus type 2 (DM2) increases tremendously with approximately 7.4% in Germany. Lifestyle modifications (LM) have preventive effect on DM2. Since lifestyle interventions aiming solely on diet and increased exercise often failed to demonstrate sustainable effects, multimodal interventions becoming increasingly important. The Aim of AIM (Anthroposophic/Naturopathic Integrative Medicine – Diabetes Intervention with Art-, Behavioral-, Exercise-Therapy and Education) study is to investigate the effects of such a multimodal LM.

**Methods**

We perform a randomized controlled exploratory trial in 50 non-insulin-depended DM 2 patients (disease duration <8 years, HbA1c ≥ 6,5%). Patients will be randomized in a study intervention group or in a waiting list control group (CG). A multimodal therapy concept (MT) consisting of 15 weekly sessions of 3.5 hours including physiotherapy, psycho-education, eurythmy -a mindfulness-oriented movement therapy- art therapy, and cooking including nutrition training was developed in interdisciplinary conferences of experts. MT aims not only to increase physical activity and to optimize nutrition, but also to improve personal competence and self-efficacy. CG receives twice an education on LM in addition to the routine treatment by general practitioners. Outcome parameter (baseline, after 2 and 4 months) are metabolic parameters (S-insulin, S-glucose, HbA1c, cholesterols, adiponectin) and various questionnaires (HRQL, autonomic regulation, self-regulation, internal coherence, and Pittsburgh Sleep Quality Index).

**Conclusions**

The results of the AIM-DIABETES study will clarify, if this multimodal treatment is a feasible preventive and therapeutic approach in DM2 patents and if benefits for the patients could be documented.

## P207 Effects of Chinese herbal decoction Qinlingye extract on the expression of NLRP3,Caspase-1,Caspase-3 in renal tissue of rats with uric acid kidney injury

### Honghong Zhang^1^, Tiesheng Fang^2^, Jie Wang^1^, Chengcheng Zhang^3^, Yuxi He^1^, Xiaoxu Zhang^1^, Zhengju Zhang^1^, Dali Wang^1^, Fengxion Meng^1^

#### ^*1*^*Dongfang Hospital, Beijing University of Chinese Medicine, Rheumatology, Beijing, China;*^*2*^*Community Health Service Center of Yangsong Town, Traditional Chinese medicine, Beijing, China;*^*3*^*Dongfang Hospital, Beijing University of Chinese Medicine, Nephrology, Beijing, China*

##### **Correspondence:** Honghong Zhang

**Purpose**

To investigate the effects of Chinese herbal decoction Qinlingye extract (QLYE) on the expression of NLRP3, Caspase-1, Caspase-3 in renal tissue of rats with uric acid kidney injury.

**Methods**

69 6-week SD male rats (200 ± 10 g) were fed adaptively for 1 week.6 rats were selected randomly as the control group, others were gavaged with adenine and fed with yeast. The successful models (n = 60) were randomly divided into model, positive drug and high-,medium-,low-dose of QLYE group, and administrated with distilled water (10 ml.kg-1.d-1/i.g), allopurinol (23.33 mg.kg-1.d-1/i.g) and QLYE (7.46 g.kg-1.d-1/i.g,3.73 g.kg-1.d-1/i.g,1.87 g.kg-1.d-1/i.g) respectively. Half the rats of each group were sacrificed at the 6th and 8th week. The mRNA transcriptions of NLRP3, Caspase-1, Caspase-3 in renal tissue were detected by RT-PCR. The protein expression of NLRP3, Caspase-3 in renal tissue were detected by Western-blot and immunohistochemical stainning .

**Results**

Compared with the control group, levels of NLRP3, Caspase-3 mRNA transcription and protein expression in the model group were higher at the 6th and 8th week (P < 0.01, P < 0.05), levels of Caspase-1 mRNA transcription was higher at the 8th week (P < 0.01). Compared with the model group, levels of NLRP3, Caspase-3 mRNA transcription and protein expression in the three herbal groups were lower at the 6th week (P < 0.01, P < 0.05) and at the 8th week, levels of NLRP3 mRNA transcription in high-,low-dose groups were lower (P < 0.01, P < 0.05), levels of Caspase-3 mRNA transcription and NLRP3, Caspase-3 protein expression in the three herbal groups were lower (P < 0.01, P < 0.05), levels of Caspase-1 mRNA transcription were lower in the high-,low-dose group.

**Conclusion**

QLYE may ameliorate the renal immune inflammatory injuries of rats with uric acid kidney injury by down-regulating the expression of NLRP3 Caspase-1 and Caspase-3.

## P208 Effect of Qinlingye extract on the PPARϒ/IL-18 signaling pathway expression in rats with uric acid kidney injury

### Jianping Zhang^1,2^, Chengcheng Zhang^1,2,3^, Hua Bai^3^, Zhiming Shen^4,5^, Weiguo Ma^4,5^, Hui Liu^4,5^, Yu Bai^4,5^, Xuezheng Shang^4,5^, Fengxian Meng^4,5^

#### ^*1*^*Dongfang Hospital, Beijing University of Chinese Medicine, Beijing China, Rheumatology, Beijing, China;*^*2*^*Dongfang Hospital, Beijing University of Chinese Medicine, Rheumatology, Beijing, China;*^*3*^*Xiluoyuan Community Health Service Center, Fengtai District, Beijing, China, Traditional Chinese Medicin, Beijing, China;*^*4*^*Jingzhou Central Hospital of Hubei Province, China, Hubei, China;*^*5*^*Dongfang Hospital, Beijing University of Chinese Medicine, Beijing China, Endocrinology, Beijing, China*

##### **Correspondence:** Jianping Zhang

**Purpose**

To investigate the effect of Chinese herbal decoction Qinglingye extract(QLYE) on the levels of PPARγ, MCP-1 and IL-18 gene transcription and protein expression in rats with uric acid kidney injury.

**Methods**

6 rats of 69 6-week SD male rats were selected randomly as control group. Others were gavaged with adenine and fed with yeast. The 60 successful models were randomly divided into model, positive drug and high-, medium-, low-dose of QLYE, and were administrated with distilled water (10 ml · kg^-1^ · d^-1^/i.g), allopurinol (23.33 mg · kg^-1^ · d^-1^/i.g) and QLYE (7.46 g · kg^-1^ · d^-1^/i.g, 3.73 g · kg^-1^ · d^-1^/i.g and 1.87 g · kg^-1^ · d^-1^/i.g) respectively. Half of the rats of each group were sacrificed at 6th week and 8th week. The mRNA transcription of PPARγ, IL-18 and MCP-1 in renal tissue were detected by RT-PCR. The protein expression of PPARγ in renal tissue was detected by Western blot, IL-18 and MCP-1 in serum were detected by and ELISA.

**Results**

Compared with the control group, the mRNA transcription and protein expression of PPARγ were lower,IL-18 and MCP-1 were higher at 6th and 8th week in model group (P < 0.05, P < 0.01). At 6th week, compared with the model group, the mRNA transcription and protein expression of PPARγ were higher, and the mRNA transcription of IL-18 were lower in all three herbal groups (P < 0.05, P < 0.01). The protein expression of IL-18 were lower in medium-dose group, MCP-1 were lower in medium- and low-dose group (P < 0.01, P < 0.05). At 8th week, the mRNA transcription of PPARγ were higher in high-and low-dose group, protein expression of PPARγ in all three herbal groups were higher (P < 0.01, P < 0.05), the mRNA transcription of IL-18 and MCP-1 were lower in medium-and low-dose group (P < 0.01, P < 0.05), The protein expression of IL-18 and MCP-1 were lower in all three herbal groups (P < 0.01, P < 0.05).

**Conclusion**

QLYE ameliorated kidney immune inflammatory injuries of UK rats by down-regulating the expression of inflammatory factors via promoting the PPARγ signal pathway.

## P209 Social interactions and support attenuate pain-induced affective responses

### Ruixin Zhang^1^, Fan Wu^2^, Ming Li^2^, Xinyun Xuan^1^, Xueyong Shen^2^, Ke Ren^3^, Brian Berman^1^

#### ^*1*^*Center for Integrative Medicine, University of Maryland, Baltimore, MD, United States;*^*2*^*Shanghai University of Traditional Chinese Medicine, Shanghai, China;*^*3*^*Department of Neural and Pain Sciences, Dental School, University of Maryland, Baltimore, MD, United States*

##### **Correspondence:** Ruixin Zhang

**Purpose**

Pain contains sensory-discriminative, affective-motivational, and cognitive-evaluative dimensions. Social interactions and support modulate cognitive activity and inhibit sensory pain. Whether social interactions and support modulate pain affect has not been determined. We utilized behavioral and optogenetic approaches to evaluate the effects and mechanisms of social interactions and support on pain-induced affective responses.

**Methods**

Adult Male and female Sprague-Dawley rats were housed respectively in pair for three weeks. The dominant/subordinate relationship of each pair was determined using a food competition test. Complete Freund adjuvant (CFA) was injected into one hind paw to induce pain. Affective responses were assessed with a conditioned place avoidance (CPA) test. Social interactions and support consist of visual and smell interactions only, or full interactions in which the CFA-injected rat interacted freely with its same-sex cage mate during pain-paired conditioning. AAV5-CaMKIIa-eNpHR3.0-EYFP at 0.75 μL was injected into the rostral anterior cingulate cortex (rACC). Optogenetic approaches were employed to inhibit the rACC neuronal activities to study their effect on CPA.

**Results**

Full interactions significantly (P < 0.05) abolished pain-induced CPA in dominant male and female rats and partially alleviated it in submissive male and female rats. Visual and smell interactions alleviated the CPA in dominant male rats but not in submissive male as well as dominant and submissive female rats. Rats providing support showed no CPA. Yellow light stimulation [200 mA (1000 mv), 0.8 Hz, 30 min] of AAV5-CaMKIIa-eNpHR3.0-EYFP-bearing rACC during pain-paired condition inhibited neuronal spikes and the CPA behavior, suggesting that social support-induced inhibition of CPA may be underpinned by the decrease of rACC neuronal activities.

**Conclusion**

Behavioral therapy may be used to relieve pain-induced affective responses and improve the quality of life. Supported by NIH R21 AT008467.

## P210 The effects of high calorie and protein diet on the intestinal microbiome of Kunming mice

### Jianhua Zhen, Xiaofei Li, Xiaohong Gu, He Yu, Zian Zheng, Yuxian g Wan, Yunhui Wang, Xueyan Ma, Fei Dong, Tiegang Liu

#### *School of Basic Medical, Beijing University of Chinese Medicine, Beijing, 100029, China*

##### **Correspondence:** Jianhua Zhen

**Objective**

To explore the impacts of high calorie and protein diet on the intestinal microbiome of Kunming mice at the molecular level by detecting the colorectal content samples by using High-throughput sequencing method.

**Methods**

The high calorie and protein diet model mice were built by feeding special fodder and the colorectal content samples in a germ-free environment were collected. The intestinal microbiome diversity and absolute/relative abundance were detected by using High-Throughputing Sequencing method, and the structure characteristics of the mice intestinal microflora were counted. In addition, the differences between groups were excavated, and the special gut microecological index initiated high calorie and protein diet was recorded.

**Results**

The intestinal microbiome of Kunming mice shows diverse structural characteristics and there are significant differences on the intestinal microbiome between high calorie and protein diet group and normal control group, with dramatically decreasing at diversity and relative abundance of Lachnospiraceae, while the characteristic index of high calorie and protein diet is OTU_1 which is annotated as: p__Firmicutes, c__Clostridia, o__Clostridiales, f__Lachnospiraceae.

**Conclusion**

High calorie and protein diet can make Kunming mice with keeping a intestinal microbiome disorder condition, with a dramatically declining at diversity and relative abundance of the dominant bacteria.

## P211 The structure of the intestinal microbiome in different gender Kunming mice

### Jianhua Zhen, Xiaofei Li, Xiaohong Gu, He Yu, Zian Zheng, Yuxiang Wan, Yunhui Wang, Xueyan Ma, Fei Dong, Tiegang Liu

#### *School of Basic Medical, Beijing University of Chinese Medicine, Beijing, 100029, China*

##### **Correspondence:** Jianhua Zhen

**Objective**

To explore the impacts of gender on the intestinal microbiome of Kunming mice at the molecular level by detecting the colorectal content samples by using High-throughput sequencing method.

**Methods**

The colorectal content samples were collected in a germ-free environment and the diversity and absolute/relative abundance of the intestinal microbiome in the samples were detected by High-Throughputing Sequencing method. The structure of the intestinal microflora on mice was described, and the differences between genders were distinguished.

**Results**

The intestinal microbiome of Kunming mice shows diverse structural characteristics and there are significant differences between male and female mice, for female mice are with a higher expression of diversity and relative abundance of Bacteroidaceae.

**Conclusion**

Gender may be one of the important factors which can impact the intestinal microbiome of Kunming mice.

## P212 Predictors of response to self-administered acupressure for persistent cancer-related fatigue in breast cancer survivors

### Suzie Zick, Richard Harris

#### *Family Medicine, University of Michigan, Ann Arbor, MI, 48105, United States*

##### **Correspondence:** Suzie Zick

**Introduction**

Persistent fatigue is a prevalent and burdensome late-term effect of breast cancer and its treatments. Our data from a phase III clinical trial (n = 288 of which 192 were randomized to receive acupressure) demonstrated that self-administered acupressure was able to significantly reduce fatigue in breast cancer survivors compared to usual care. However, 39.5% of women did not achieve clinically significant reduction in fatigue severity, and were thus non-responders to acupressure treatment. We analyzed participants baseline sociodemographic, clinical and patient centered outcomes to predict non-response to acupressure treatment.

**Method**

Correlations were conducted between change in fatigue (measured by the Brief Fatigue Inventory {BFI} baseline to 6-week visit) and continuous variables stratified by fatigue responders (defined as BFI score <4 at end of acupressure treatment) and non-responders (BFI ≥ 4). Chi-square tests were conducted examining the association between fatigue responders and non-responders. Binary logistic regression (fatigue responders vs. non-responders) was used to conduct multivariate analysis of those variables found to be significant (p ≤ 0.05) in bivariate analysis as well as being adjusted for age, and treatment group (relaxing acupressure, stimulating acupressure, usual care).

**Results**

Unadjusted analyses found a significant association (p < 0.05) between response to acupressure and baseline: pain interference (from the Brief Pain Inventory,), Long Term Quality of Life (LTQL)-somatic subscale, age at time of study entry, yearly household income, menopausal status at time of study entry, Hamilton Anxiety and Depression Scale-depression subscale, maximum activity count-from actigraphy, and average time in bed (from 14-day sleep diary). However, in adjusted analysis only yearly household income (lowest {≤$25,000 USD} vs. highest {≥$75,000 USD} income category; odds ratio {OR} = 3.8, 95% confidence interval {CI} 1.1–13.8, p = 0.04), menopausal status at time of study entry (OR = 11.0, 95% CI 1.0–124.2, p = 0.05), and LTQL-somatic subscale (OR = 0.9, 95%CI = 0.9–1.0, p = 0.03) continued to significantly predict response to acupressure treatment.

**Conclusions**

Easy to obtain measures can be employed by healthcare providers to help determine who may be the most likely women to benefit from self-administered acupressure for treating persistent cancer-related fatigue.

Trial Registration: clinicaltrials.gov Identifier: NCT01281904

## CLINICAL CARE

### P213 Moxibustion as an adjuvant for benign prostatic hyperplasia with lower urinary tract symptoms: a parallel group, randomized, controlled pilot trial

#### Go E Bae^1^, Jung N Kwon^1^, Hye Y Lee^1^, Jong K Nam^2^, Sang D Lee^2^, Dong H Lee^2^, Ji Y Han^2^, Young J Yun^1^, Ji H Lee^1^, Hye L Park^1^, Seong H Park^1^

##### ^*1*^*Pusan National University Korean Medicine Hospital, Internal Medicine, Yangsan-si, South Korea;*^*2*^*Pusan National University Yangsan Hospital,, Department of Urology, Yangsan, South Korea*

###### **Correspondence:** Jung N Kwon

**Purpose**

This study aims to explore feasibility of using moxibustion as a supplementary intervention and to assess the sample size for verifying the effectiveness and safety.

**Methods**

This study is a randomized controlled trial with a parallel-group and pragmatic design. A total 29 patients diagnosed benign prostatic hyperplasia (BPH) randomized to either conventional group (C.G, n = 14) or integrative group (I.G, n = 15). The conventional treatment set as the optimum treatment based on the urological doctor (UD)’s opinion. And the integrative group treated additional moxibustion therapy twice a week for 4 weeks. The moxibustion therapy was conducted on the SP6, LR3 and CV4 by a Korean medical doctor. The primary outcome was International Prostate Symptom Score (IPSS) after 4 weeks. The secondary outcome was Patient’s global impression of changes (PGIC) after 4 weeks, IPSS, PGIC and post-void residual urine volume after 12 weeks.

**Results**

Total 29 patients (C.G:14, I.G:15) were included in intention-to-treat (ITT) analysis and 25 patients (C.G:11, I.G:14) were included in per-protocol(PP) analysis. The primary outcome was statistically significant difference (p = 0.0439) in PP analysis (I.G;-2.57 ± 4.29, C.G; 1.00 ± 3.97) and no significant difference (p = 0.0693) in ITT analysis. Among the IPSS contents ‘incomplete emptying’, ‘straining’ were statistically significant difference (p ≤ 0.05) in ITT analysis. In the secondary outcomes, PGIC after 4 weeks was significant difference (p = 0.0023) in ITT analysis. There were no adverse events in both groups.

**Conclusions**

We can assess the effectiveness of the moxibustion therapy for BPH patients. And we can calculate a powered appropriate sample size according to results of IPSS, the sample size considered a 20% dropout rate would be 29 participants per group.

### P214 Integrated approach: Complementary medicine, exercise and nutritional program at Fondazione Salvatore Maugeri (FSM) - Pavia

#### Chiara Bocci, Giovanni B Ivaldi, Ilaria Vietti, Ilaria Meaglia, Marta Guffi, Rubina Ruggiero, Marita Gualea, Emanuela Longa

##### *S. Maugeri Fondation, Pavia, 27100, Italy*

###### **Correspondence:** Chiara Bocci 

**Background**

As recommended by EUSOMA (European Society of MAstology), in 2012 we introduced the use CAM, such as Homeopathy/Phytotherapy, Exercise Therapy and Nutritional Therapy, in our Breast Unit (BU) to identify the needs, hopes and to improve the quality of life of patients(pts).

**Materials and Methods**

The Breast Unit of Maugeri Foundation of Pavia in collaboration with the Interdepartmental Center of Biology and Sport Medicine of the University of Pavia started in March 2012 an integrated approach to analyze the different effects of complementary medicine, exercise, and nutritional program on the prognosis of breast cancer patients.

In our approach, the breast nurse welcomes the patient, provide her with a description of the project. Each patient has the possibility to choose one or more among complementary medicine, nutritional, and exercise program.

We carried out 460 visits of Homeopathy and Phytotherapy (mean age 57 aa.),70 pts required exercise therapy (average age 54 aa.) and 140 pts had a consultation with the dietitian (mean age 55 aa.).A form regarding quality of life (MUS) was distributed to patients who choose exercise program. Patients were medically evaluated by staff (anthropometrical analysis, maximal oxygen consumption and spirometric evaluation, blood sample with attention to specific parameters such as lipid profile and glycemic profile,). A personal training program which defines frequency, intensity, time and workloads in agreement with ACSM guidelines was given. Re-evaluation was done every 3 months to check progress. The dietary consultation investigated any inconvenience that followed oncological therapies (disgust, vomiting, loss of appetite, greed, predilections) and also evaluated knowledge about food, the awareness of the role of nutrition in health and disease and how much the patients were willing to change their dietary habits, according to nutritional guidelines.

**Results**

CAM is usually required for the treatment of vasomotor syndrome, RT-skin toxicity, CRF, anxiety, insomnia, gastrointestinal disorders and to improve QoL. Pts who followed the training program had an improvement of maximum oxygen consumption (VO2 max), ventilation (VE), respiratory parameters (FVC, FEV1, Tiffenau Index) but not a decrease in BMI. Pts who followed the nutritional program had a reduction of BMI.

**Conclusion**

The treatment with CAM, nutritional, and exercise program for the effects of cancer therapies was safe, well accepted and able to meet the unmet needs of patients with BC.

### P215 Benefits of integrated therapy for patients operated for breast cancer

#### Massimo Bonucci

##### *S. Feliciano Hospital, Outpatient Oncology, Rome, Italy*

**Introduction**

Survival in breast cancer has improved in the last decade. A specific instrumental diagnosis was added with the result of the detection of very small lesions (even just a few millimeters in diameter). All this thanks to long lasting pharmacological therapies such as that hormonal therapy. Relapse within 5 years are increasingly rare, with percentage of absence of disease of 60/80%, though after the discovery and surgery patients undergoing radio / chemotherapy and / or followed by hormone therapy. The integrated treatment involves, in addition to adequate diet during and after therapy, the use of natural substances and methods designed primarily to reduce the side-effects of traditional therapies (both radio and chemotherapy of new immunological therapies) and then further reduce the rates of medium and long-term recurrence. By recognizing the natural substances immediate benefit on nausea, vomiting, fatigue, dermatitis, mucositis, arthralgia, neuropathy or even stimulation of hematopoiesis, we can consider the usefulness of the medium and long time integration, also about evaluation of the reduction of cancer recurrence in a group of patients who have taken an integrated treatment for more than 5 years.

**Materials and methods**

In the period 2010–2016 225 patients with breast cancer in the absence of secondary lesions at baseline were examined.

Of these, 187 patients underwent surgical treatment with subsequent startup to chemo / radiotherapy and / or hormonal therapy. The remaining 38 patients after surgery, by their own choice, did not received any classical treatment (chemo-radiotherapy and / or hormonal therapy) but only nutrition and integrated therapy. 225 patients had no relapses during the study period.

**Discussion**

Breast cancer is undoubtedly the most frequent cancer in women. Fortunately, it is also the most treatable, especially in case of small size lesions and in absence of metastatic spread. It’s still a disease which may recur in the first 5 years in a percentage that varies from 20 to 30% even if the patients are subjected, after surgery, to classic anticancer therapies. In this retrospective study, all the patients followed adequate nutrition, plus an integrated treatment (herbal medicine, anthroposophic medicine, micotherapy, TCM) either without any further treatment or after the classic anticancer protocol, and in all of them no case of recurrence, both locally and at a distance, has been found until now.

Further studies are necessary to assess how the integrated therapy can play a role in the prevention of medium and long-term relapses.

### P216 Results from a study of CAM centres across the UK and Europe

#### Sarah Croke (dr.sarah.croke@hotmail.com)

##### *School of Heathcare, University of Leeds Alumnus, Rochdale, OL12 7RX, United Kingdom*

**Purpose**

This study sought to capture the models of delivery enacted by practitioners of Complementary and Alternative Medicine (CAM) in different contexts, to illuminate any impact of these on clinical care delivered and outcomes experienced.

**Methods**

The research employed Ethnography augmented by Theatricality to engage in-depth with the people (characters), processes (plots) and places (settings) of therapy in 5 clinical sites across the UK, Holland, Cyprus and Denmark. Embodied engagement and ‘performative criticism’ allowed the researcher to be drawn into these performances and individual stories, while also being critical of the complexities that created them.

**Results**

Despite some overlap in therapies provided, there were stark differences in these performances of CAM. The UK site presented an almost ‘biomedical’ model; clinical and white coats, that limited expectations and potential outcomes to physical ones. The Holland sites presented a typical anthroposophical model; cosy houses, lots of wood, artwork, flowers and crystals, where expectations and outcomes were often surpassed and everyone felt cared for and ‘carried’. The Cyprus site presented ‘an oasis in the desert’; this remote location gave a sense of escape from the limits of biomedicine, creating a space for miracles. The Denmark sites were functional yet welcoming; each provided comfortable ‘homes’ for therapists to express their identity and connect with clients on a ‘human’ level.

**Conclusions**

CAM presented in different places, by different people, performed different intentions impacting the limits and potential of clinical care and outcomes experienced. This suggests a reality that all practitioners and researchers may learn from.

### P217 Massage therapy improves psychological state and cognitive function in breast cancer patients undergoing chemotherapy treatment

#### Lourdes Diaz Rodriguez^1^, Juan C Caracuel-Martínez^2^, Manuel F Fajardo-Rodríguez^2^, Angélica Ariza-García^2^, Francisca García-De la Fuente^2^, Manuel Arroyo-Morales^1^

##### ^*1*^*Nursing, Faculty of Health Sciences/University of Granada, Granada, 18971, Spain;*^*2*^*Physiotherapy Unit, University Hospital San Cecilio/Health Andalusian Service, Granada, Spain*

###### **Correspondence:** Lourdes Diaz Rodriguez

**Purpose**

The main aim of this study was to compare the immediate effects of a Miofascial therapy session versus nutrition education intervention (placebo) during 30 minutes in breast cancer patients undergoing chemotherapy treatment.

**Methods**

A randomized, single-blind, controlled, crossover study was carried on 22 female patients with a mean (SD) age of 49.32 (9.95) years. Study inclusion criterias were age between 18 and 65 years, a primary diagnosis of breast cancer (grades I–IIIa) undergoing chemotherapy treatment. Exclusion criteria were: presence of metastasis and/or active cancer. Written informed consent was obtained from all participants, and the local ethics committee approved the study. The outcomes measured before and after Miofascial and Nutrition interventions were anxiety, stress, relaxation and neuropsychological state.

**Results**

The study included 18 Caucasian and 1 sudamerican female patients. No significant between-group differences were found in descriptive statistics or in the pre-intervention value of any outcome measure. Patients reported after 30 minutes of miofascial therapy, lower levels of anxiety (F = 11.48, p = 0.00) and stress (F = 9.2, p = 0.01), and higher levels in relaxation (F = 12.69, p = 0.00) and in attention and mental flexibility (TMT-B) (F = 5.56, p = 0.02) compared to the control group. There were no significant differences in pain, in affected (F = 3.30, p = 0.07) and in non-affected side (F = 2.49, p = 0.12) and in visual search and motor speed (F = 1.14, p = 0.29) between interventions.

**Conclusions**

30 minutes of miofascial treatment improves psychological state and cognitive impairment by enhancing attention, mental flexibility and relaxation and reducing stress, and anxiety levels in breast cancer patients undergo chemotherapy treatment.

### P218 Tetanus case report

#### Maria S Estrems^1^, Vicente G Gómez^2^

##### ^*1*^*Fundació Sànitaria de Mollet, Surgery, Barcelona, Spain;*^*2*^*Hospital de Viladecans, Barcelona, Spain*

###### **Correspondence:** Maria S Estrems (marisolestrems@hotmail.com)

A healthy 65-year-old caucasian woman sustained a stab wound above her left ankle from a piece of cane. The patient was admitted into hospital with a presumed diagnosis of a wound infection. Treatment with amoxicillin-clavulanic acid was initiated. The wound was debrided under local anesthesia two days after admission. Cramps became increasingly severe over the next 36 hours with spastic equino deformity of foot. The patient worsened with hyperexcitability to touch when wound was cleaned and dressed, bilateral spasms in both lower extremities to tactile stimulus, dysphagia and some desaturation episodes that required traqueostomy for mechanical ventilation on day 8. *Clostridium tetani* was isolated from the wound and a new surgery was required for wide debridement or amputation. At this point the patient was under treatment with midazolam, cisatracurio and bolus of atracurium during the change of dressings, then homeopathic treatment with Hypericum 30 ch and Nux Vomica 9 ch and Ledum Palustre 200 ch was given prior and after the surgery. Decompression fasciotomy and exeresis of peroneus longus, peroneus brevis and tibialis anterior muscles was performed on day 15. Cisatracurio was stopped the next day. She needed only the bolus of atracurium during the change of dressings. The patient exhibited clinical improvement the next postop 48 h with a small aggravation (day 19) when wound was cleaned. On day 18 atracurium was not needed. On day 19 atracurium was given and then stopped definitely. The patient exhibited clinical improvement and she was extubated on day 30 and discharged to home on day 45 in good health.

Written informed consent from the patient was obtained for publication.

### P219 The impact of Integrative Medicine on the postoperative course of acute peritonitis

#### Maria S Estrems^1^, Mónica Valero Sabater^2^

##### ^*1*^*Fundació Sànitaria de Mollet, Surgery, Barcelona, Spain;*^*2*^*Hospital Royo Villanova, Zaragoza, Spain*

###### **Correspondence:** Maria S Estrems (marisolestrems@hotmail.com)

**Aims**

To asses the effects of Integrative Medicine on postoperative complications in patients operated upon for secondary acute peritonitis.

**Methods**

A non-randomized, retrospective, unicentric clinical study of 517 consecutive patients operated on for secondary community peritonitis admitted as surgical emergencies from January 1994 to December 2004. Integrative Medicine (Homeopathy and Bach Flowers Remedies plus Allopathic Therapy) was given to 84 patients (Group IM) whereas Allopathic Therapy alone was given to the remaining 433 (Group AT). Allopathic Therapy included surgical source control, broad spectrum antibiotherapy and metabolic support measures as needed. Major and minor postoperative complications (Clavien-Dindo Classification), postoperative hospital stay and postoperative mortality were compared between both groups.

**Results**

The distribution of aetiologies were comparable in both groups, and included Acute Appendicitis (43.83%), Acute Cholecystitis (14.8%), Acute Left Colon Diverticulitis (6.07%), Perforated Peptic Ulcer (15.74%), Small Intestine perforation (3.79%), Colon perforation (5.5%) and Others (0.75%). There were no significant differences between IM and AT in Major complications (1.17% vs 1.66%; P = 0.3) or postoperative hospital mortality (3.5% vs 4.2%; P = 0.3). There was a trend towards fewer minor postoperative complications (Clavien I-II) in the IM group. The postoperative hospital stay was significantly shorter for patients of the IM group (8.92 ± 1.0 vs 11.98 ± 0.6 days; P < 0.001). No complications potentially related to IM were observed.

**Conclusions**

Patients receiving IM had a significantly shorter postoperative stay probably linked to less postoperative ileus and other non severe complications.

**Acknowledgements**

The author wishes to thank nurses Maribel Sánchez and Maribel Corral for their unconditional support, Dr. Assumpta Mestre, friend and professor, for advice onhomeopathy and Dr Sergio Abanades for believing in this study.

### P220 The role of comorbidities in the challenge of integrated cares in chronic diseases at the Hospital Centre of Integrated Medicine- Pitigliano, Italy

#### Rosaria Ferreri, Simonetta Bernardini, Roberto Pulcri, Franco Cracolici, Massimo Rinaldi, Claudio Porciani

##### *Hospital Centre of Integrated Medicine, Hospital of Pitigliano ASL Sudest Toscana, Grosseto, 58100, Italy*

###### **Correspondence:** Rosaria Ferreri

**Question**

Patient-centered care’s approach should not only be considered as a goal, but as a precondition of our health care system in the future. For that, integrated medicine has emerged as good to realize this approach, providing cares that are patient-centered, healing oriented, emphasizing the therapeutic relationship. We have investigated the potential role of integrated medicine in relation with comorbidities, that are those chronic conditions associated to a principal disease, often responsible for the worsening of the chronic patient’s status.

**Methods**

We report the incidence of comorbidities in chronic diseases (arthrorheumatic, allergic, oncological, neurologic) and the effectiveness of our integrated model of cares including homeopathy, acupuncture and life-style advices in the management of these patients; evaluations have performed through following parameters: QoL, global health and pharmacological load at 1, 2, 6 and 1 year –follow up.

**Results and conclusions**

We’ll report our results and considerations about:

I comorbidities that are combined with the examined chronic diseases and why they areimportant in the evaluation of integrated protocols;
II the integrated approaches that have been able to reduce the impact of symptom related to comorbidities in almost 45% to 90% of the patients;
III the use of conventional drugs for the comorbidities that have been reduced up to 100% in the follow up period;

We also show some examples of integrated approach through explicative clinical cases

### P221 Adverse reactions and interactions of herbal medicine with anticancer therapy: a review of the literature and recommendations

#### Fabio Firenzuoli^1,2,^ Sonia Baccetti^2^, Mariella Di Stefano^2^, Maria V Monechi^2^, Eugenia Gallo^1^, Valentina Maggini^1^, Luigi Gori^1^, Elio Rossi^2^

##### ^*1*^*Referring Centre for Phytotherapy, Center for Integrative Medicine, Careggi Universitary Hospital, Florence, 50139, Italy;*^*2*^*Tuscan Network of Integrative Medicine, Florence, Italy*

###### **Correspondence:** Fabio Firenzuoli

**Background**

Medicinal herbs are often used in cancer patients for the prevention and side effect treatment of chemo- and radiotherapy, and for the improvement of patients’ quality of life.

**Purpose**

To evaluate the available literature aiming to identify the adverse reactions and interactions of herbal medicines with chemotherapy and to find a tool, useful in the prevention and risk reduction, for oncologists and CAM operators.

**Methods**

A ten-year extensive search was performed up to 2014, concerning the possibility of negative interaction (interference) or positive (synergy) between herbal products and conventional therapy (chemotherapy and radiotherapy).

**Results**

This work shows the drugs that have been seen or could potentially interact with investigated medicinal plants: *St. John’s Wort, Grapefruit, Liquorice, Ginkgo, Ginseng, Soy, Echinacea, Rhodiola, Boswellia, Aloe, Ginger, Saffron, Astragalus, Lavandula, Green tea, Curcuma* and *Silymarin*.

We provide a summary table on the known *in vitro* and/or *in vivo* interactions or sinergy (with or without clinical evidence) between medicinal plants and chemo or radiotherapy taking into account the Reversed *grading* proposed by the Tuscan Network for Integrated Medicine. The grading is defined Reversed because the main level of evidence corresponds to the main level of negative recommendation. Expert opinions (*e.g.* EMA) on the uses and/or interactions are also reported, when present.

**Conclusions**

Literature analysis has allowed the classification of the medicinal plants used for cancer patients, relying on the clinical risk, even potential, of interaction or synergy with anticancer therapy, in particular using the *Reversed grading*, a useful tool in clinical practice for all oncologists of our Public Regional Healthcare System.

### P222 A feasibility study of Acupuncture to improve quality of life and fatigue in cancer patients undergoing Radiotherapy Treatment (ART)

#### Peter Fisher^1^, John Hughes^1^, Ariadna Mendoza^2^, Hugh MacPherson^3^, Claudia Witt^4^, Jacqueline Filshie^5^, George Lewith^6^

##### ^*1*^*Royal London Hospital for Integrated Medicine, UCLH NHS Trust, London, WC1N 3HR, United Kingdom;*^*2*^*UCH Cancer Clinical Trials Unit, London, United Kingdom;*^*3*^*University of York, York, United Kingdom;*^*4*^*University Hospital Zurich, Zurich, Switzerland;*^*5*^*Royal Marsden Hospital, London, United Kingdom;*^*6*^*University of Southampton, Southampton, United Kingdom*

###### **Correspondence:** Peter Fisher (peter.fisher@uclh.nhs.uk)

**Purpose**

To determine the feasibility of a clinical trial to evaluate whether acupuncture, administered by trained radiotherapists, helps symptoms of patients receiving radiotherapy for cancer.

**Methods**

A pragmatic randomised pilot feasibility study comparing standard care versus standard care plus acupuncture, with nested qualitative study to investigate the feasibility of possible future full-scale randomised controlled clinical trial of acupuncture delivered by radiotherapists with customized training in acupuncture, for cancer patients undergoing radiotherapy. Aspects investigated included processes, resources, interventions, procedures, and outcomes in the University College Hospital radiotherapy department. Qualitative telephone interviews were conducted with patients and stakeholders. Clinical outcomes include Quality of Life (EORTC QLQC-30, EQ-5D-5 L), Fatigue (MFI), symptoms (MSAS).

**Results**

The recruitment phase is complete. The recruitment target of 100 was met. Collaboration and dedicated staff in the UCH Cancer Clinical Trials Unit were crucial to success. Adjustments to processes during the study enabled it to meet its target. It is feasible to train radiotherapists to deliver acupuncture in a busy radiotherapy department. Preliminary analysis of qualitative data from patients and treating radiotherapists showed favourable attitudes. Some stakeholders expressed misgivings, but these referred to issues subsequently resolved. Final outcome data are due in November 2016.

**Conclusions**

This design is feasible for a full scale randomised controlled trial. We will present preliminary results at the conference. A decision to proceed to a definitive trial depends on identifying the primary outcome, clinically relevant effect size and power calculation.

### P223 Treatment of tinnitus by way of photostimulation via visible/IR wavelength on acupuncture points

#### Antonia Di Francesco, Alberto Bernardini, Monica Messe, Vincenzo Primitivo, Piera A Iasella

##### *IOMED, Lecce, Italy*

###### **Correspondence:** Antonia Di Francesco (antoniadf@iomed.it)

**Objective**

The goal of this study was to evaluate the reduction of tinnitus in three patients aged between 61 and 65 years, undergoing treatment of photostimulation via visible/IR-wavelength on acupuncture points.

**Method**

Data were collected from 3 patients suffer from tinnitus. Patients were subjected weekly to 8 photostimulation treatments. In the three cases we worked on the general point of lymphatic drainage on the face in front of the tragus of the two ears with the visible light wavelenght of yellow at 578 nm, kidney/bladder functional circle starting from GV 26 with red at 650 nm and VG 25 with orange at 590 nm nape point periauricular with IR at 950 nm according to the colorpuncture protocols by P. Mandel. Kidney/bladder functional circle was choosen because the ear is the kidney window to Traditional Chinese Medicine. The frequencies of visible light are emitted by incandescent lamp and selected by a filter. The disorder tinnitus assessment is made on the patients’s subjective perception, through the Tinnitus Handicap Inventory test (THI). Beside that, was carried out the evaluation of the frequency of the tinnitus occur (days per month) and the evaluation of intensity of the disorder on a scale from 0 to 10, where 0 indicates the absence of the phenomenon, and 10 is the maximum of the disorder manifested in terms of patient volume. THI, frequency and intensity tests were carried out before the start of cycle of eight treatments and after the 8th treatment.

**Results**

The tinnitus group exhibited decrease of disorder. The three patients have shown 68% of average noise reduction of THI test:

- Patient A, female, 61 years: the disorder is reduced by 96% (from 72 to 4 THI test)
- Patient B Male 64 years: the disorder is reduced by 65% (80 to 28 THI test)
- Patient C male patient 65 years: the disorder is reduced by 44% (from 82 to 46 THI test)

The frequency of tinnitus was reduced from an average value of 28 To 12 days per month. The intensity of the disorder was reduced from an average value of 10 to 4.

**Conclusion**

Photostimulation on specific points from Tradicional Chinese Medicine or French auricolotherapy with specific bioresonance frequencies from visible ed IR light provides interesting answers to reduction of a functional tinnitus disorder.

**Consent**

Informed consent for publication has been given by all patients.

### P224 Partnership for implementing Integrative Health and Medicine on pediatric oncology: development of a nursing care program based on Anthroposophic therapy and support for the pediatric hospital in Berlin and São Paulo

#### Ricardo Ghelman^1^, Monica Taminato^1^, Jaqueline Do Carmo Alcantara^1^, Katia R De Oliveira^1^, Debora C De Azevedo Rodrigues^1^, Juliana R Campana Mumme^1^, Olga K Matsumoto Sunakozawa^1^, Vicente Odone Filho^1^, Georg Seifert^2^

##### ^*1*^*Instituto do Tratamento do Câncer Infantil – Department of Pediatric Oncology, Faculdade de Medicina da Universidade de São Paulo, Sao Paulo, 05410-030, Brazil;*^*2*^*Department of Pediatric Oncology, Charité University Medicine, Berlin, Germany*

###### **Correspondence:** Ricardo Ghelman (ric.ghelman@gmail.com)

**Introduction**

The nurse is the more available professional for identification of individual needs and for interventions with supportive integrative care plans. The central components in the performance of anthroposophical nursing procedures are direct physical touch, promotion of adequate rhythm, thermal regulation, mindful empathy and compassion, and a calm, safe atmosphere for patients and their parents. The procedures provide an opportunity to intensify the therapeutic results through an innovative and holistic model into the university hospital.

**Objective**

To develop and implement a program of anthroposophical nursing and supportive therapies for use as part of standard patient care in a pediatric oncology ward. Implement a non-pharmacological integrative care protocols to promote the wellbeing to patients, families and care team in the different settings of the Pediatric Oncology Hospital based on education program and evaluation.

**Results**

The development of activities and nursing staff training in the Department of Pediatric Oncology of University of Sao Paulo and Charité University are being carried out in cooperation with the Integrative Hospital Havelhöhe and Witten-Herdecke. Nursing groups at the Sao Paulo University Hospital involve the four settings of the Pediatric Oncology: Bone Marrow Transplant Unit, Intensive Care Unit, Out-patients Clinic and In-Patients Ward. We achieve the approval of clinical protocol of external therapies for nursing care from the Ethic Committee and Academic Board of the Hospital. In addiction we have introduce the concept of Integrative Health and Medicine in the scientific meetings and we are developing the training program to be implemented in standard care in pediatric oncology accompanied with appropriate scientific support.

### P225 Establishing naturopathic dietary interventions for irritable bowel syndrome: an e-Delphi protocol

#### Joshua Goldenberg^1,2^, Andrew Day^1^, Masa Sasagawa^1^, Lesley Ward^2,3^, Kieran Cooley^2,4^

##### ^*1*^*Bastyr University, Kenmore WA 98028-4966, United States;*^*2*^*Australian Research Centre in Complementary and Integrative Medicine, University of Technology, Sydney, Australia;*^*3*^*Nuffield Dept of Orthopaedics, Rheumatology & Musculoskeletal Sciences, University of Oxford, Oxford, United Kingdom;*^*4*^*The Canadian College of Naturopathic Medicine, Toronto, Candada*

**Purpose**

Although patients with irritable bowel syndrome (IBS) often seek dietary advice from naturopathic doctors (NDs), there are no guidelines to inform optimal provision of care. To address this gap, we sought consensus amongst expert NDs on their dietary strategies to assess and treat IBS.

**Methods**

Purposive and snowball sampling identified 15 North American NDs with expertise in dietary approaches in IBS. Participation was limited to North America due to the relatively homogenous training and scope of practice within jurisdictions. All potential participants were invited to participate using an introductory email. Following informed consent, these panelists will participate in an anonymous online- e-Delphi comprised of three rounds of questions. Items are rated on a 100-point scale (0 = completely disagree to 100 = completely agree) visual analogue scale. First round items were generated by a steering committee using existing literature on naturopathic dietary approaches. Items are then rated for inclusion or exclusion based on *a priori* cut-offs, with consensus defined as median score of ≥75% on an item, and exclusion defined as a median score of <40%. Items not reaching consensus (median scores of ≥40% - <75%) will be forwarded to the next round for re-rating. In Round 1, panelists have an opportunity to provide open-ended suggestions to include in subsequent rounds.

**Results**

A steering committee will oversee all survey development and analysis of results. Following each round, the group of panelists will receive feedback in the form of a summary of aggregate findings. Questionnaire items will be analyzed quantitatively; median and inter-quartile ranges for each item will be reported.

**Conclusion**

Using Delphi methods, NDs with expertise in treating IBS will identify a core set of key dietary interventions to assess and treat IBS. This will address current heterogeneity within the profession and provide a reference tool for best practices for clinical care, education and future research. This tool will provide guidance while remaining flexible and open to clinician autonomy and patient preferences.

### P226 Use of recreational activities and complementary therapies in Icelandic Nursing Homes

#### Thora Gunnarsdottir, Ingibjorg Hjaltadottir

##### *Faculty of Nursing, University of Iceland, Reykjavik, 105, Iceland*

###### **Correspondence:** Thora Gunnarsdottir (thoraj@hi.is)

**Question**

Research has indicated that over half of nursing home residents spend little or no time in recreational activities and depression and behavioral symptoms are considerable. Complementary therapies have been used additionally with recreational activities to enhance positive wellbeing among older people. The aim of this study was to investigate what kind of recreational activities and complementary therapies (RAC) are provided in Icelandic nursing homes, who provide such therapies and what assistance the nursing homes need to provide such services.

**Methods**

Two questionnaires one with 19 questions about the use of recreational activities and one with 14 questions about the use of CT were developed and send to all nursing homes or total of 59.

**Results**

The findings show that all of the nursing homes offer recreational therapies and 43 (90%) of those nursing homes who replied offer some kind of CT. This is mostly planned by nurses and license practical nurse. The most common RAC are reading, singing, physical activity and heating paths, but many other therapies were used. About 90% of the nursing homes would like to have some more support to implement further RAC, especially complementary therapies, in the form of lectures, courses and visit to other nursing homes.

**Conclusion**

It is important to know what is being used and what assistance nursing homes need to enhance the use of RAC with the aim to offer implementation and education to increase the use of such service and measure if changes in symptoms can be detected.

### P227 Cinnamon supplementation in patients with polycystic ovary syndrome, a double blind randomized clinical trial

#### Mahdie Hajimonfarednejad (hajimonfared@yahoo.com)

##### *Shiraz University of Medical Sciences, Shiraz, 71747637, Iran, Islamic Republic Of*

**Objective**

To assess the efficacy and safety of cinnamon powder capsules in patients with Polycystic Ovarian Syndrome (PCOS), as a complementary therapy to standard treatment.

**Method**

A randomized double-blind placebo-controlled trial was conducted. Sixty six patients with PCOS were randomly allocated to be treated with cinnamon powder or placebo (1.5 gram/day in 3 divided dose for 12 weeks) in two groups. Insulin resistance (HOMA-IR index, the homeostatic model assessment), anthropometric factors, metabolic parameters and serum androgen were considered as the outcome measures.

**Results**

Cinnamon powder significantly reduced fasting insulin (p = 0.024), HOMA-IR (p = 0.014) and LDL (p = 0.049) of the participants after the intervention in comparison with the placebo group.

**Conclusion**

Complementary supplementation of cinnamon may improve insulin resistance and LDL in patients with PCOS.

### P228 Practical clinical management of eosinophilic oesophagitis (EoE) using CAM: a focus on increasing awareness and understanding of this rapidly emerging inflammatory gastrointestinal disease

#### Nicole Hannan (info@alchemyhealth.com.au)

##### *Alchemy Health & Wellbeing, Miami, 4220, Australia*

Eosinophilic oesophagitis (EoE) is a chronic antigen-driven inflammatory disease characterised histologically by high levels of eosinophils in the oesophagus and clinically by oesophageal dysfunction and gastrointestinal (GI) symptoms. Untreated EoE can lead to considerable GI complications. Although the prevalence of EoE has increased dramatically in the past decade to approximately 1 in 2,000 in Westernized countries (as high as 1 in 10 in patients with Crohns disease), under diagnosis due to poor practitioner awareness and invasive diagnostic techniques indicates that actual numbers are likely to be even greater. There are currently no pharmaceutical or complementary and alternative medicine (CAM) therapies specifically indicated for the management of EoE. Within conventional medicine, swallowed topical steroids, elimination diets and elemental formula are the main currently recognised methods of management. Severe allergy/intolerance reactions to foods, herbs, nutritional supplements and environmental allergens are common in EoE, requiring adequate understanding of this disease for integrative medicine (IM) and CAM practitioners. This presentation will focus on deepening practitioners understanding of elemental formula, pharmaceutical treatments and elimination diet recommendations as it relates to EoE; and explore practical management of EoE for CAM and IM practitioners. Given the allergic nature of EoE the value added by a CAM practitioner can be in the ability to identify possible current triggers and ways to safely modify existing treatment protocols for optimal patient outcomes.

### P229 Difficulties and possibilities in the dialogue about alternative cancer treatments: patients’ and oncologists’ views

#### Rut Hellsing^1^, Kathrin Wode^1^, Johanna Hök Nordberg^2^

##### ^*1*^*Regional Cancer Center Stockholm/Gotland, Stockholm, SE-102 39, Sweden;*^*2*^*Department of Neurobiology, Caring Sciences & Society, Karolinska Institutet, Huddinge, SE-14183, Sweden*

**Background & Aim**

Studies show that a small but significant part of cancer patients decline one or several conventional treatments and instead turn to alternative treatments (ATs). These situations have been shown to be difficult both for patients and oncologists. With the long-term objective to improve communication between oncologists and patients, this study aims to explore views on the dialogue about ATs.

**Material and Methods**

A qualitative design with a hermeneutic approach was used. Seven patients and six oncologists were selected for face-to-face interviews. The selection of patients was based on principles of extreme sampling and represented those who had declined any conventional cancer treatment. Oncologists were chosen based on their interest in the topic. Interviews were transcribed verbatim and analysed by all three researchers.

**Results**

Our preliminary analysis shows that the dialogue between patients and oncologists about ATs involves difficulties as well as possibilities. Difficulties include: Diverging and sometimes incompatible views on ATs, the patients’ exposed role as people with a life-threatening diagnosis and, the oncologists’ role as representatives of conventional medicine. The analysis also reveals an opportunity for reconciliation between patients and oncologists when knowledge, time and humility permeate the dialogue about ATs.

**Conclusions**

While there are inherent difficulties in the dialogue about ATs, this study highlights areas where the tension between patients and oncologists may be unnecessarily high. With improved understanding of potential conflicting views regarding ATs in cancer care, health care professionals are better prepared to engage in respectful dialogue with patients about treatment choices.

### P230 Glimpses of a good life: A qualitative study of patients’ experiences of anthroposophic pain rehabilitation

#### Johanna Hök Nordberg, Susanne Andermo, Maria Arman

##### *Department of Neurobiology, Caring Sciences & Society, Karolinska Institute, Huddinge, SE-14183, Sweden*

###### **Correspondence:** Johanna Hök Nordberg

**Question**

Little is known about how to best design rehabilitation programs for persons with chronic widespread pain (CWP) and its overlapping illnesses. With the long-term objective to improve CWP care, the aim of this study was to explore patients’ lived experiences of an anthroposophic rehabilitation program for CWP.

**Methods**

A hermeneutic phenomenological design was used. Ten women undergoing a three-week in-patient rehabilitation program for CWP at an anthroposophic clinic were purposively selected for face-to-face interviews. Interviews were transcribed verbatim and analysed by all three researchers.

**Results**

The analysis resulted in three themes: *A permissive caring atmosphere* where the every-day life is put on hold, the women are allowed to rest and to be vulnerable in a caring community with others. The second theme— *Therapies for change and inspiration*—include descriptions on how the women embark on an inner journey where therapies such as massage, art- and bath-therapy work as catalysts for getting new perspectives on themselves and their pain. Finally, the theme *Glimpses of a good life* presents experiences of a renewed sense of wonder, joy and creativity in life; existential openings that give promise for a future in better health.

**Conclusion**

This study underscores the importance of an existential approach to CWP rehabilitation and highlights the potential influence of the overall caring culture on such rehabilitation. Given the emphasis on experiences of both suffering and health, the findings also raise the question: Is the interdependent relationship between suffering and health sufficiently addressed in current rehabilitation programs?

### P231 Complementary Medicine inputs in a patient with non-small cell lung cancer stage IV

#### Iris von Hörsten^1^, Patricia Vásquez Torrielo^1^, Carmen L Andrade Vilaró^1^, Francisco Cerda Cabrera^2^

##### ^*1*^*Hospital San Juan de Dios, Internal Medicine, Santiago de Chile, Chile;*^*2*^*Centro de Estudios e Investigación Terapéutica, Santiago de Chile, Chile*

**Introduction**

Female, 72, with arterial hypertension, diabetes mellitus 2, non-smoker. Diagnosed 03/2012 of mucinous pulmonary adenocarcinoma*,* right lobe + pleural effusion, stage IV. Treatment with chemotherapy (Cisplatin) + radiotherapy (66gy). Patient initial weight, 58 kg – BMI 22.9 (07/2012), post chemo, 49 kg – BMI 19,4 (09/2012). Pain treatment with paracetamol and tramadol. CT scan control (11/2012): Multi-focal lung impacts at right side, signs of carcinomatous lymphangitis. Pleural effusion right. Mediastinal lymphadenopathy. Referred to Homeopathic Medicine Unit, while maintaining conventional treatments.

**Description**

29.01.2013 First homeopathic consultation to establish personal motivation and bio-pathology. Patient wishes to gain weight, and I want to learn to live a bit better. I do things from the past, I want to live to recover (my) time. She comes from a rural background, oldest of 10 siblings, three died from malnutrition. At seven she moved to the city for schooling; she was sad and lonely. As an adult, she was politically active against the dictatorship. She became pregnant at 36, not able to rest, miscarrying, on 1 May 1976. She thinks frequently of this child what would have happened to him?. Her partner disappeared, six days later. She continues to pursue his case. Years later she married, had a child. She continued to be politically active, neglecting her child, with guilty Evidentially she was held in the grip of past emotions which prevented her from connecting with the present, from feeling existential security, protection, and confidence in affective relations. Through psiconeuroimmunology mechanisms she developed pathologies described.

Treatment following the Healing Path method was begun, with homeopathic medicine ultrahigh diluted dinamizations of C10.000. After one month, it was possible to approach the subject of reconciliation. The process deepened: By 02/2014 she said: Im very close to my husband, we can agree on things, we can laugh. Speaking of the past she says There is PEACE. Weight stable and pain is minimum. 05/2015: The cancer has progressed, weighs 50 kg, requires Paracetamol 1 gr/d, and oxygen. 07/2016: She died in her home peacefully; without advanced pain treatment.

**Discussion**

Complementary medicine helped the patient become conscious of her present existential process and provided tools for a life-change. Her life-expectancy in 01/2013 were 12 months which she excided by 30 months.

Informed consent was obtained from the patient to publish this information.

### P232 Treating irritable bowel syndrome – science meets art

#### Roman Huber

##### *Center for Complementary Medicine, University Medical Center Freiburg, Freiburg, 79106, Germany*

**Purpose**

About 30% of patients visiting a gastroenterologist and about each third patient presenting at his general practitioner with abdominal complaints suffers from a functional disorder, dyspepsia or irritable bowel syndrome (IBS). Patients are often regarded as difficult from conventional medicine.

**Methods**

At University Hospital Freiburg, Germany, we have developed an evidence based integrative IBS diagnostic- and treatment program. Official IBS guidelines were reviewed for complementary therapies. Diagnostic and therapeutic approaches as well as communication strategies were reflected in case conferences.

**Results**

Official guidelines mention multiple evidence based IBS treatment modalities but prioritization and approaches to adapt them to the individual needs of the patients are lacking. Most important in diagnostics are anamnestic features of the exact symptom modalities to exclude or confirm relevant differential diagnoses. Most important therapy is the right way of talking with the patient in order to explain the functional nature of the problem in a way that the patient understands what he has. For successful communication the concept of an activated brain – gut-brain axis and affirmation by the patient have been helpful. The message that everything is alright organically is not sufficient. Efficacy of the right way of talking is supported by the differential symptomatic use of life style changes, external applications and (mostly herbal) medications. Regarding the benign nature of the problem medications should be as harmless as possible.

**Conclusions**

Integrative multimodal treatment concepts in patients with IBS should be further evaluated. The right way of talking and adjustment of possible treatment options to the individual needs is more than an operational procedure, it remains art of healing.

### P233 Establishing an Integrated Chinese-Western Medicine care model for in-patient through a pilot program in Hong Kong – practical experience for quality assurance

#### Henny Hui, Eric Ziea, Dora Tsui, Joyce Hsieh, Christine Lam, Edith Chan

##### *Chinese Medicine Department, Hospital Authority, Hong Kong, Hong Kong*

**Purpose**

In line with the Hong Kong Governments 2013 Policy Address, the Hospital Authority (HA) of Hong Kong has been tasked to test out the logistical model for providing ICWM care to inpatients via the Integrated Chinese-Western Medicine (ICWM) Pilot Programme in public hospitals for the first time. To facilitate smooth operational logistics, a series of guidelines have been developed. Besides, programme audit exercises have been carried out to ensure the compliance with established guidance.

**Methods**

The Pilot Programme was launched in September 2014 and after one-year of implementation, programme audit exercises were carried out on the administration of oral Chinese Medicines by nurses; the documentation of ICWM related medical records; and the documentation of consultation notes by CM Practitioners.

**Results**

100% compliance was observed on administration of oral Chinese Medicines and the filing and completion of medical forms and records. 96% compliance was achieved for documentation of consultation notes by CMPs. The non-compliance area included incompleteness of CM treatment record. Recommendations included sharing of good practice, further training for CMPs on the medical record documentation, and using templates to facilitate the documentation process. A re-audit was subsequently conducted in May 2015 to review the implementation and full achievement was obtained.

**Conclusion**

ICWM care is new and challenging care model introduced in public hospitals. The audit exercise serves as a tool to identify difficulties within the model. Through finding out the non-compliance areas, it helps to enhance the mechanisms, continuously refine the model and pave the way for future development.

### P234 Use of neurofeedback and mindfulness to enhance response to hypnosis treatment in individuals with multiple sclerosis

#### Mark P Jensen^1^, Samuel L Battalio^1^, Joy Chan^1^, Karlyn A Edwards^1^, Kevin J Gertz^1^, Melissa A Day^2^, Leslie H Sherlin^3^, Dawn M Ehde^1^

##### ^*1*^*University of Washington, Seattle, WA, USA;*^*2*^*University of Queensland, Brisbane, Australia;*^*3*^*Southwest College of Naturopathic Medicine, Tempe, AZ, USA*

The purpose of this pilot study was to determine if neurofeedback (NF) or mindfulness meditation (MM) training might enhance the efficacy of hypnosis (HYP) for decreasing chronic pain and fatigue in people with multiple sclerosis (MS). Based on the previous findings that (1) response to hypnosis treatment may be facilitated by more slow wave (especially theta) brain oscillation activity, and (2) NF and MM can increase slow wave activity, we hypothesized that study participants with MS and either pain or fatigue would who received NF or MM training prior to hypnosis treatment would report greater treatment-related improvements than participants who received hypnosis alone. Thirty-two individuals with MS were randomly assigned to one of three treatment conditions: (1) NF to increase theta oscillations (10 sessions) + HYP (5 sessions); (2) MM (10 sessions) + HYP or (3) HYP-ONLY. The hypnosis treatment targeted reductions in pain and fatigue. Large between-group effects were found for improvements in pain intensity (favoring NF + HYP) and small effects were found for improvements in fatigue (favoring both NF + HYP and MM + HYP). In exploratory analyses, large between-group differences were also found for improvements in sleep quality (favoring NF-HYP). The findings indicate that the beneficial effects of hypnosis may be enhanced by providing training in mindfulness or neurofeedback prior to the initiation of hypnosis treatment. Further research is needed to evaluate the reliability of these preliminary findings.

Keywords: Hypnosis, Neurofeedback, Mindfulness, Pain, Fatigue

### **P235 Analysis on Medical assistance during international sports event** - **Focus on traditional Korean medicine**

#### Kyeong H Kim, Soobin Jang, Bo-Hyoung Jang, Ho-Yeon Go, Sunju Park, Seong-Gyu Ko

##### *Management Policy, National Development Institute of Korean Medicine, Gyeongsan-si, 38540, South Korea*

###### **Correspondence:** Kyeong H Kim

**Question**

Traditional Korean Medicine (TKM) including acupuncture, taping, chuna, and herbal medicines have been actively utilized in many sports games. This study aimed to analysis result of medical assistance using TKM during international sports event held in Korea since 2000.

**Methods**

A search was conducted 5 databases using the keywords: “medical assistance”, “traditional medicine” and so on. And report collected from Association of Korean Medicine (AKOM) officially. Data without medical record was excluded.

**Results**

Three medical assistance using TKM during international sports events; Busan Asian Games (2002), Daegu Universiade (2003), Incheon Asian Games (2014) identified. A total of 1,829 patients (male:female = 1,990:639) was treated. Most of patients were athlete (51.9%), followed by official (27.0%). Only one treated patients (58.4%) is more than treated over twice. Patients have low back pain (27.0%), shoulder joint pain (18.1%), knee joint pain (10.7%), neck pain (10.4%). TKM doctors use acupuncture (36.4%), physical therapy (35.5%), taping (13.7%), chuna (7.4%), cupping (6.9%) for treatment.

**Conclusions**

It was conducted steadily that medical assistance using TKM during international sports event. It is necessary to prepare systemic assistance reflecting the result of medical record analysis.

### P236 Complementary medicine used for treatment of children during a mother-child health retreat severely influences their mothers’ preference of treatment methods for their children

#### Karin Kraft^1^, Hubert Janik^1^, Anja Börner^2^

##### ^*1*^*Naturopathy, University of Medicine, Rostock, 18057, Germany;*^*2*^*Urology, University of Medicine, Rostock, 18057, Germany*

###### **Correspondence:** Karin Kraft

**Question**

In Germany, rehabilitation clinics offer mother-child health retreats e.g. for the treatment of recurrent viral infections of the respiratory tract (RVIRT) in children. As it is well-known that these infections are often treated with complementary medicine (CM), it was of interest to evaluate the spectrum of methods used before and during the retreat and the possible change of preferred treatment methods.

**Methods**

In an epidemiological study mothers of children with RVIRT received questionnaires at the first and the prefinal day of their stay in a rehabilitation clinic at the seaside. Important items of the questionnaire were CM methods used before and during the retreat, and CM methods which the mothers would prefer for the treatment of their children thereafter.

**Results**

310 mothers with 388 children with RVIRT completed the questionnaire. Before rehabilitation 77.1% of the children were treated with home remedies, 47.4% with homeopathy. Any other CM method had been used in less than 20% of the children. During rehabilitation 65.2% received inhalations at the seaside, and 32.5% balneotherapy with natural brine. Both methods are evidence-based in RVIRT. However, also scientifically not proven methods were applied: Schuessler salts (31.4% of the children), Chinese massage (19.6%), and Bach flowers (11.3%). Mothers then wanted their children to be treated with Schuessler salts (36.2% of the mothers), Chinese massage (32.7%) and Bach flowers (23.6%) in the future, but showed much less interest in evidence-based methods.

**Conclusions**

The mothers’preference of scientifically unproven methods for the further treatment of their children gives cause of concern. These methods therefore should not be used during mother-child retreats, and the mothers should preferably be trained on the use of evidence-based CM methods.

### P237 A survey of Korea Medicine doctors’ clinical patterns for autism spectrum disorder: Preliminary research for clinical practice guidelines

#### Jihong Lee, Boram Lee, Gyu T Chang

##### *Pediatrics of Korean Medicine, Kyung Hee University Hospital at Gangdong, Seoul, South Korea*

###### **Correspondence:** Boram Lee (qhfka9357@naver.com)

**Background**

The aim of this study was to investigate Autism Spectrum Disorder (ASD) clinical practice patterns of Korean Medicine doctors (KMDs) through questionnaire survey.

**Methods**

Questionnaires on Korean medicine (KM) treatment for ASD were distributed to 255 KMDs on December 5th, 2016. The KMDs were KM psychiatrists, pediatricians, or general practitioners who took care of ASD patients. The questionnaire covered items on treatment methods, aims of treatment, KM syndrome differentiations, diagnostic tools and sociodemographic characteristics.

**Results**

A total 21.18% KMDs (n = 54/255) completed the questionnaires and matched the inclusion criteria. The KMDs utilized herbal medicine (90.74%), acupuncture (20.37%), scalp acupuncture (16.67%), Chuna manual therapy (11.11%) and psychotherapy (9.26%) to ASD patients. The most commonly prescribed herbal medicine was Yookmijihwang-tang. Forty eight (88.89%) KMDs responded that they used KM syndrome differentiations. ‘Organ system diagnosis’, ‘Qi and Blood diagnosis’, ‘Yin and Yang diagnosis’ and ‘Fluid and humor diagnosis’ were frequently used for syndrome differentiations. The diagnosis of ASD was commonly made on the basis of the fourth edition of the Diagnostic and Statistical Manual for Mental Disorders (DSM-4) and DSM-5.

**Conclusions**

This study is the first expert opinion investigation on KM treatment of ASD. Future clinical practice guidelines will be based on diagnostic and treatment patterns of KM.

This study was supported by the Traditional Korean Medicine R&D program funded by the Ministry of Health & Welfare through the Korea Health Industry Development Institute (KHIDI). (HB16C0075)

### P238 Psychoanalysis in Integrative Medicine

#### Alejandra Menassa (alejandramenassa@live.com)

##### *Mental Health, CMI (Clinica Medicina Integrativa), Madrid, 28015, Spain*

**Purpose**

Integrative Medicine considers the patient holistically, taking into account both its physical aspects as psychics. The mind-body techniques are one of the instruments of this approach. There are some international initiatives including psychoanalysis as one of these mind-body techniques. The purpose of this paper is to show how psychoanalysis can be a very useful tool in the treatment of various organic diseases. Sometimes, consider the unconscious psychic factors is key to solving cases. The psychoanalytic method takes into account these factors, which are usually overlooked in other techniques of psychological approach.

**Methods**

common theoretical bases that allow the alliance between integrative medicine and psychoanalysis and three cases gathered in Madrid, Spain, in the last two years in CMI (Integrative Medicine Clinic) shown are analyzed. These are cases addressed in an integrative manner, including instruments such as orthomolecular medicine, high doses of vitamin C intravenously, biorresonancia med-tronic, anti-inflammatory diet and psychoanalysis.

**Results and conclusions**

Psychoanalysis, along with other instruments of Integrative Medicine, may increase treatment success rates.

### P239 Chemotherapy Support Team (CST) and Kampo

#### Yoshiharu Motoo (motoo@kanazawa-med.ac.jp)

##### *Department of Medical Oncology, Kanazawa Medical University, Uchinada, Ishikawa, 920-0293, Japan*

Chemotherapy Support Team (CST) is a comprehensive, multidisciplinary team to support cancer patients who receive chemotherapy. Patients experience various problems during the period of chemotherapy including molecular targeting agents. They include physical symptoms as adverse drug reactions in addition to mental, psycho-social, and spiritual problems. Kampo can be applied tomany kinds ofsymptoms such as oral mucositis, diarrhea, anorexia, general malaise, or muscle pain. In order to use Kampo drugsfor cancer patients, we need evidence to validate their efficacy and safety. If there are some evidence, CST members would confidently accept Kampo drugs as integrative medicine. EBM Committee of Japan Society for Oriental Medicine (JSOM) has published Evidence Reports of Kampo Treatment (EKAT) since 2007. We analyzed structured abstracts (SAs) of EKAT in the field of cancer. Systematic review (SR) revealed that most SAsneed more detailed descriptions in necessary items recommended in theConsolidated Standards of Reporting Trials (CONSORT) statement 2010.

### P240 Evidence based medicine in functional gastrointestinal diseases: metaanalysis of clinical trials with STW 5

#### Jürgen Müller^1^, Sabine Rabini^2^, Bettina Vinson^1^, Olaf Kelber^1^, Martin Storr^3,4^, Karin Kraft^5^

##### ^*1*^*Phytomedicines Supply and Development Center, Steigerwald Arzneimittelwerk GmbH, Bayer Consumer Health, Darmstadt, 64295, Germany;*^*2*^*Department of Medical Cell Biology, Philipps-University Marburg, Marburg, Germany;*^*3*^*Center for Endoscopy Starnberg, Starnberg, Germany;*^*4*^*Großhadern Hospital, University of Munich, Munich, Germany;*^*5*^*Naturopathy, Center for Internal Medicine, University of Medicine, Rostock, Germany*

###### **Correspondence:** Olaf Kelber (olaf.kelber@bayer.com)

**Introduction**

The gastro-prokinetics, such as metoclopramide, domperidone and cisapride, had been introduced to the therapy of functional gastrointestinal diseases in the 70s and 80s, and for a long time they seemed to be standard therapeutics in this indication. Since they are no longer available for the use in this indication due to restrictions of their marketing authorizations based on rare but severe side effects, other well proven therapeutic options gain increased attention. One of these is STW 5 (Iberogast), for which more than 5 decades of therapeutic experience in more than 60 Mio patients are available.

**Aims**

Whether also the available clinical data comply to modern standards for a proof of efficacy was now tested by a meta-analysis of the randomized placebo-controlled double blind trials available in the therapeutic indication functional dyspepsia.

**Methods**

The individual datasets from all trials were entered and demografic data and primary endpoints were evaluated (ANCOVA).

**Results**

The primary outcome variable, the validated gastrointestinal symptom score (GIS) [1], as well as the therapeutic dose (3 × 20 drops/day) were identical in all trials, so allowing a uniform evaluation. The full analysis set (FAS) included 557 patients (272 resp. 285 for placebo resp. verum). The mean age (48 resp. 49 years), the mean body size (in both groups 168.7 cm), the mean body weight (72.0 resp. 72.2 kg), the BMI (25.35 resp. 25.54), the gender disitribution (67.3 resp. 69.5% females), the duration of the disease at the time of inclusion and the baseline of the GIS (11.6 resp. 11.5 points) were very well comparable between both groups. For the primary variable GIS the difference between placebo and verum after 28 days of treatment showed a highly significant (p < 0.0001) difference between placebo and verum (6.7 resp. 4.7 points).

**Conclusions**

This meta analysis therefore clearly shows the efficacy of STW 5 (Iberogast) according to present standards of evidence-based medicine (EBM). In addition it emphasizes the high quality of the trials, which is indicated e.g. by the very good balance between both patient groups. Additional insights can be expected from further analyses, as e.g. the evaluation of different subgroups with specific predominant symptoms or demographic properties.

**Reference**

1. Adam et al. Aliment Pharmacol Ther 2005; 22: 357–363

### P241 Determination of ICC Patterns in children with developmental 1disorders

#### Martin Niemeijer, Erik Baars, Joop Hoekman, Wied Ruijssenaaars

##### *University of Applied Sciences, Leiden, 2333CK, Netherlands*

###### **Correspondence:** Martin Niemeijer

**Question**

The problems of children with a developmental disorder show considerable variability. In addition to diagnosing as conventional classification we developed for an appropriate individual diagnostics and treatment the Instrument for diagnostics of a Childs Constitution (ICC). Question in this study is to determine or there are in the series of individual constitution profiles recognizable specific patterns for several developmental disorders.

**Methods**

The psychometric properties of the ICC were assessed in a study with 535 children with developmental disorders and 148 children without established developmental disorder. For this study we explored by means of determination of the average scores in the outcomes of the ICC patterns for several developmental disorders.

**Results**

Children with Autism Spectrum Disorders, ASD (N = 133), Attention Deficit Hyperactivity Disorders, ADHD (N = 74), Multiple Complex Developmental Disorders, MCDD (N = 29), Attachment disorders (N = 62), Down-syndrome (N = 33) demonstrate specific ICC patterns. This also applies to the ICC pattern in children without developmental disorder.

**Conclusions**

Specific ICC patterns are associated with specific developmental disorders (ASD, ADHD, MCDD, Attachment disorders, Down-syndrome). They can be distinguished from the ICC pattern of children without established developmental disorder. For treatment the classified disorder may provide general direction that will be individualized by the childs profile of the constitution that is established with the ICC.

### P242 Yoga for Chronic Neck Pain- A Systematic Review and Meta-Analysis

#### Faith C Njoku^1^, Petra Klose^2^, Benno Brinkhaus^3^, Andreas Michalsen^3^, Gustav Dobos^2^, Holger Cramer^2^

##### ^*1*^*Irvine School of Medicine, Irvine, University of California, Irvine, CA, United States;*^*2*^*Internal Medicine and Integrative Medicine, University of Duisburg-Essen, Essen, Germany;*^*3*^*Institute for Social Medicine, Epidemiology and Health Economics, Charité University Hospital, Berlin, Germany*

###### **Correspondence:** Faith C Njoku (fnjoku@uci.edu)

**Objective**

The purpose of this review is to systematically review and perform a meta-analysis on the effectiveness of yoga for relieving chronic neck pain.

**Methods**

The methods performed in this study utilized the PubMed/MEDLINE, the Cochrane Library, Scopus, and IndMED as a screening assessment through the month of December in 2015. These engines were evaluated for previous randomized controlled trials (RCTs) that assessed a patients neck pain intensity and/or related disabilities resulting from chronic neck pain. Secondary outcome measures included a patients quality of life, mood, and safety. The risk of bias was assessed using the Cochrane tool.

**Results**

Four RCTs on 288 patients with chronic non-specific neck pain (3 RCTs) or cervical spondylosis (1 RCT) comparing yoga to usual care were included. Two RCTs had overall low risk of bias; and two had high or unclear risk of bias for several domains. Evidence for short-term effects was found for neck pain intensity (standardized mean difference (SMD) = −1.37; 95% confidence interval (CI) = −1.78, −0.97; P < 0.001), neck pain-related disability (SMD = −0.97; 95%CI = −1.44, −0.50; P < 0.001), quality of life (SMD = 0.57; 95%CI = 0.17, 0.197; P = 0.005), and mood (SMD = −1.02; 95%CI = - 1.38, −0.65; P < 0.001). Effects were robust against potential methodological bias and did not differ between different intervention or patient subgroups. In the two RCTs which included safety data, no serious adverse events occurred.

**Discussion**

The short-term effects Yoga has on chronic neck pain regarding the quality of life, mood and its related disability, suggest that yoga may be a viable treatment option. However this study quality was heterogeneous and the long-term effects of yoga were not investigated. Future large-scale and high quality trials on yoga in chronic neck pain including a long-term follow-up are highly warranted.

Keywords: Neck Pain, Yoga, Complementary Therapies, Meta-analysis, Review

### P243 Acupuncture for frostbite sequel

#### Arne J Norheim, Terje Alræk

##### *National Research Center in Complementary and Alternative Medicine (NAFKAM), Department of Community Medicine, Faculty of Health Sciences, The Arctic University of Tromsø, Tromsø, 9037, Norway*

###### **Correspondence:** Arne J Norheim (arne@avital.no)

**Question**

Frostbites have for decades been a relevant problem in the military, and continue to be so. Newer therapies aimed at prevention of extensive tissue injuries have shown promising results in experimental studies and case reports. The pathophysiology in cold injuries and sequelae are still poorly understood although peripheral neurovascular dysfunction is impaired. Acupuncture has previously been described to influence the local circulatory mechanisms. Dynamic Infrared Thermography (DIRT) has been used to document microvascular effects following acupuncture stimulation.

**Methods**

The patient was a 19 years old previously healthy, non-smoking female soldier. She had no previous history of cold injuries. During outdoor military training in January 2015 in the harsh North-Norwegian climate, she noticed that she began to loose feeling in her fingers that during the exercise turned into dark discoloration with blisters. She was diagnosed with a second degree frostbite on the fingertips of both hands. Spontaneous recovery was followed the first year after she was injured, but she still got complaints with sensory-motor disturbances and hypersensitivity to cold. During the follow-up, she was examined by DIRT and offered off-label treatment with acupuncture. Acupuncture points were used to enhance peripheral blood circulation. Traditional Chinese Acupuncture was added to these points according to the patients’ symptom of feeling cold in general. Acupuncture treatment were given once a week for 12 weeks.

**Results**

After treatment, the patient reported less cold sensitivity, and there was an objective improvement shown in the thermographic examinations.

**Conclusions**

Acupuncture therapy might be a new and promising treatment for frostbite sequelae.

### P244 Acupressure on the Sanyinjiao (SP6) versus synthetic oxytocin during labour

#### Filiz Okumus^1^, Halime Oncu-Celik^2^

##### ^*1*^*Department of Midwifery, Istanbul Medipol University, Istanbul, 34083, Turkey;*^*2*^*Delivery room, Istanbul Bahcelievler Public Hospital, Istanbul, 34180, Turkey*

###### **Correspondence:** Filiz Okumus (fokumus@medipol.edu.tr)

**Purpose**

The aim of this study was to evaluate the effects of SP6 acupressure versus synthetic oxytocin on duration of delivery, pain and anxiety during labour.

**Methods**

The study was conducted in a private hospital in Istanbul, Turkey between July and December 2014. Primipara women (n = 100) with singleton pregnancies in the active phase of spontaneous labour (SP6 group: 50; synthetic oxytocin group: 50), were enrolled in this single-blinded, randomized, clinical trial. The labour pain level was measured using the visual analog scale, and the anxiety level was measured using the STAI form. The delivery process was monitered using a Partogram. Ethical approval for the study was obtained from the local ethical committee of the Istanbul Medipol University. The data were analyzed using SPSS software.

**Results**

There was significant progress in the acupressure groups number of contractions and cervical dilatation, effacement and fetal head levels. A shorter delivery duration **(** ***p < 0.001)*** and less labour pain **(** ***p < 0.001)*** were observed in the acupressure group. The anxiety level significantly decreased in the acupressure group and increased in the synthetic oxytocin group **(** ***p < 0.001).***

**Conclusions**

These findings showed that acupressure at SP6 was more effective than synthetic oxytocin for shortening the duration of delivery. In addition, SP6 was reduced labour pain and anxiety level.

**Kery words**

Delivery, Birth Pain, Oxytocin, Sanyinjiao, Acupressure

**About this supplement**

These abstracts have been published as part of *BMC Complementary and Alternative Medicine* Volume 17 Supplement 1, 2017. The full contents of the supplement are available online at https://bmccomplementalternmed.biomedcentral.com/articles/supplements/volume-17-supplement-1. Please note that this is part 2 of 3.

